# Insights into the Design of MYC-Targeting Proteolysis Targeting Chimeras (PROTACs)

**DOI:** 10.3390/molecules31061011

**Published:** 2026-03-17

**Authors:** Abdallah M. Alfayomy, Sven Hagemann, Matthias Schmidt, Ali Fouad, Mohamed Ayman El-Zahabi, Stefan Hüttelmaier, Wolfgang Sippl

**Affiliations:** 1 Department of Medicinal Chemistry, Institute of Pharmacy, Martin-Luther-University of Halle-Wittenberg, 06120 Halle (Saale), Germany; abdallah.alfayomy@pharmazie.uni-halle.de (A.M.A.); matthias.schmidt@pharmazie.uni-halle.de (M.S.); 2 Department of Pharmaceutical Chemistry, Faculty of Pharmacy, Al-Azhar University, Assiut 71524, Egypt; alifouad247@gmail.com; 3 Department of Molecular Medicine, Faculty of Medicine, Martin-Luther University Halle-Wittenberg, 06120 Halle (Saale), Germany; sven.hagemann@medizin.uni-halle.de (S.H.); stefan.huettelmaier@medizin.uni-halle.de (S.H.); 4 Pharmaceutical Medicinal Chemistry & Drug Design Department, Faculty of Pharmacy (Boys), Al-Azhar University, Cairo 11884, Egypt; malzahaby@azhar.edu.eg

**Keywords:** proteolysis-targeting chimeras (PROTACs), protein degradation, MYC

## Abstract

The oncogenic transcription factor MYC is a key driver of the development and progression of various types of cancer, but its intrinsically disordered structure and dependence on protein–protein interactions make it a difficult therapeutic target. Proteolysis-targeting chimeras (PROTACs) are bifunctional molecules that can induce the selective degradation of disease-relevant proteins. In this study, we report the synthesis and biological testing of a series of novel MYC-targeted PROTACs derived from the MYC inhibitor EN4. These ligands were conjugated to cereblon (CRBN) or von Hippel–Lindau (VHL) E3 ligase recruiters using different linker architectures and connection sites. The resulting PROTACs were synthesized in high purity and characterized analytically. Cellular evaluation in HEK293T, Panc-1 and HCT-116 cancer cells revealed only moderate reductions in cell viability. Unfortunately, none of the synthesized PROTACs showed detectable MYC degradation at biologically relevant concentrations. Testing the stability of the PROTACs in microsomes showed rapid degradation, which may be a reason for the observed inactivity in cells. These results underscore the significant challenges associated with the targeted protein degradation of intrinsically disordered transcription factors such as MYC. Further studies are necessary to identify additional causes for the lack of MYC degradation and to optimize the chemical structures accordingly.

## 1. Introduction

The MYC transcription factor regulates the expression of numerous target genes involved in essential cellular processes, including cell growth and proliferation, cell-cycle progression, differentiation, apoptosis, and angiogenesis. To exert its transcriptional activity, the MYC protein must heterodimerize with its obligate partner MAX through their shared basic helix–loop–helix leucine zipper (bHLH-LZ) domains. This dimerization is required for DNA binding and for the activation of MYC-dependent gene networks [1,2,3]. Under normal conditions, MYC expression is tightly regulated and increases only in response to specific biological cues, such as mitogen-stimulated proliferation. In cancer, however, MYC expression and activity are frequently co-opted by tumor cells, resulting in markedly elevated MYC RNA and protein levels. Given its widespread overexpression and central role in tumor initiation, maintenance, and progression, MYC represents a highly compelling target for therapeutic intervention [4,5,6].

**Figure 1:**
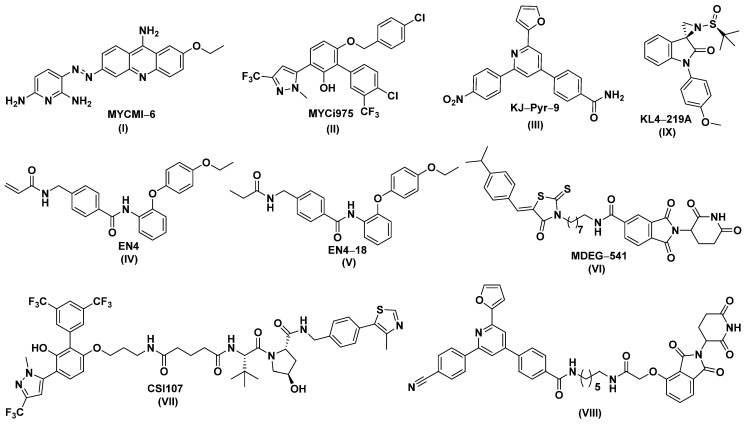
Example of reported MYC inhibitors and degraders.

Despite its critical involvement in malignancy, MYC has long been considered an undruggable target due to its intrinsically disordered structure, lack of enzymatic activity, and reliance on protein–protein interactions for function [3]. Consequently, early therapeutic strategies predominantly focused on indirect MYC suppression by targeting upstream signaling pathways, transcriptional regulation, or MYC protein stability [7]. More recent efforts have focused on developing direct MYC inhibitors that block formation of the MYC-MAX heterodimer (Figure 1). Among the most promising small-molecule inhibitors are MYCMI-6 (**I**), MYCi975 (**II**), and KJ-Pyr-9 (**III**), all of which have been shown to disrupt MYC-MAX interaction and reduce proliferation in several cancer cell lines [8,9,10]. In addition, EN4 (**IV**) was identified as a covalent ligand that targets Cys171 of MYC. EN4 has been reported to reduce MYC and MAX thermal stability, inhibit MYC/MAX DNA binding in vitro, impair MYC transcriptional activity in cells, and inhibit proliferation and tumorigenesis in breast cancer cells [11]. Interestingly, its saturated analogue EN4-18 (**V**) was also able to inhibit MYC and MYC/MAX DNA binding in vitro [11].

Proteolysis-targeting chimeras (PROTACs) have emerged as a promising modality for addressing previously undruggable proteins by harnessing the ubiquitin–proteasome system to induce targeted protein degradation. Unlike conventional inhibitors, PROTACs do not require high-affinity binding to active sites; instead, they promote the formation of a ternary complex between the target protein and an E3 ubiquitin ligase, thereby facilitating ubiquitination and subsequent proteasomal degradation of the target protein [12,13]. In recent years, several studies have reported the development of PROTACs designed to induce MYC degradation. However, most of these molecules showed limited degradation potency or insufficient selectivity [14,15,16]. For example, the thalidomide-based PROTAC MDEG-541 (**VI**) was shown to induce MYC degradation in gastrointestinal cancer cells. Nevertheless, this compound displayed poor selectivity, as it also triggered the degradation of CRBN neosubstrates, including G1-to-S phase transition proteins 1 and 2 (GSPT1/2) and Polo-like kinase 1 (PLK1) [14]. The VHL-based PROTACs CSI107 (**VII**) induced MYC degradation in PC3 cells and displayed anti-proliferative activity against PC3 and SKBR3 cancer cells (IC_50_ values of 13 and 19 μM, respectively) [15]. In addition, the CRBN-based PROTAC MTP3 (**VIII**) was reported to deplete MYC protein levels. However, it also led to the formation of a stable N-terminal deletion product that retains MYC-driven tumorigenic cell states [16]. More recently, the covalent small-molecule degrader KL4-219A (**IX**) was identified as a MYC degrader that binds covalently to Cys203, leading to MYC destabilization and proteasome-dependent degradation. Despite this activity, KL4-219A exhibits multiple off-target effects, underscoring the continued need for new molecular scaffolds capable of more effectively and selectively modulating MYC oncogenic activity [17].

**Figure 2:**
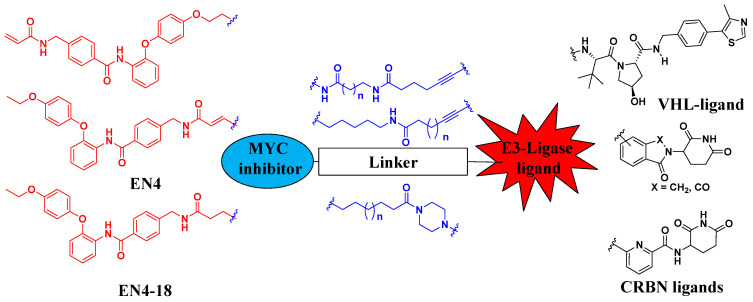
Strategy for design of MYC PROTACs based on EN4 and its analogue.

In the present work, we describe the design and synthesis of a new series of MYC-targeting PROTACs generated by conjugating the reported MYC inhibitors (EN4 and EN4-18) to VHL and CRBN E3 ligase ligands through different attachment points, as illustrated in Figure 2. The synthetic strategy was optimized to enable systematic variation of both linker length and E3 ligase recruitment.

## 2. Results and Discussion

### 2.1. Chemistry

**Scheme 1:**
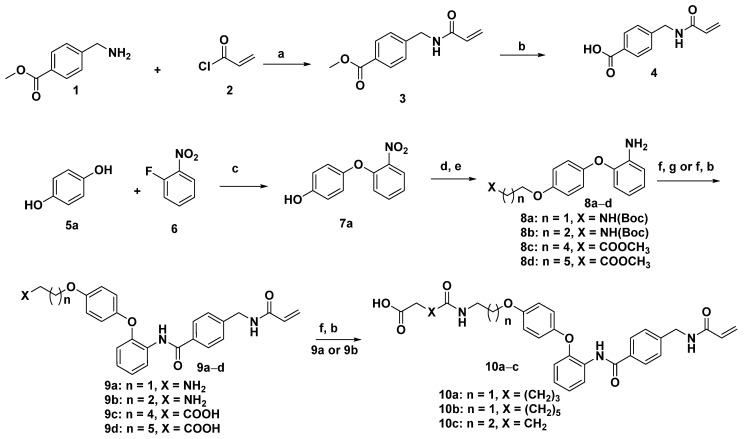
Reagents and conditions: (a) TEA, acetonitrile; (b) LiOH, THF, H_2_O; (c) K_2_CO_3_, acetonitrile; (d) alkyl bromide, Cs_2_CO_3,_ DMF, 80 °C; (e) iron, NH_4_Cl, 96% EtOH; (f) appropriate acid, HATU, DIPEA, DMF; (g) DCM, TFA.

**Scheme 2:**
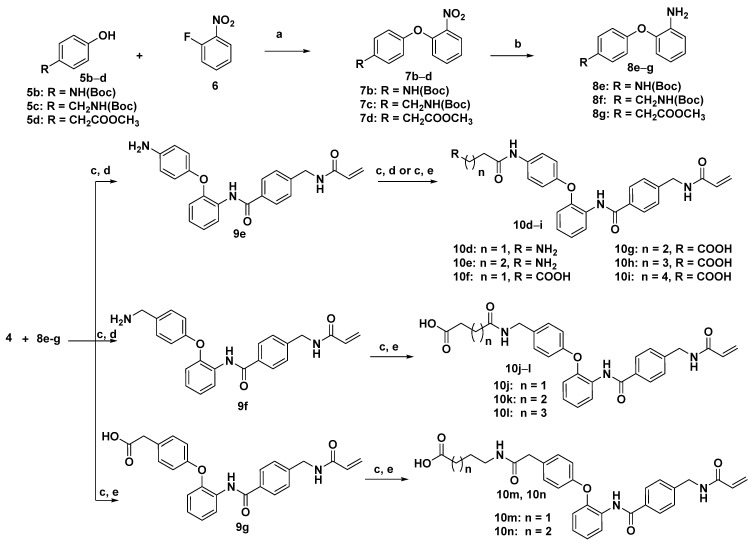
Reagents and conditions: (a) K_2_CO_3_, acetonitrile; (b) iron, NH_4_Cl, 96% EtOH; (c) appropriate linker, HATU, DIPEA, DMF; (d) DCM, TFA; (e) LiOH, H_2_O, THF.

The designed PROTACs were synthesized via amide coupling reactions between linker-functionalized MYC inhibitors (**9a**–**g**, **10a**–**n**, **30**, **31a** and **31b**) and various CRBN- or VHL-based ligands (**14a**–**d**, **15**, **20a**, **20b**, **26** and **33**), as outlined in the following schemes. The synthetic routes leading to the linker-connected MYC inhibitors **9a**–**g** and **10a**–**n** are depicted in Scheme 1 and Scheme 2. Initially, methyl 4-aminomethyl benzoate **1** was reacted with acryloyl chloride **2** to afford the corresponding acrylamide derivative **3**. Subsequently, ester hydrolysis using lithium hydroxide yielded the corresponding carboxylic acid **4** [11]. In parallel, hydroquinone **5a** and the 4-substituted phenol derivatives **5b**–**d** were reacted with 2-fluoronitrobenzene **6** to generate the corresponding diphenyl ether derivatives **7a**–**d** [11]. The 4-(2-nitrophenoxy)phenol **7a** was then alkylated with various alkyl bromides in the presence of cesium carbonate. Subsequent reduction of the nitro group using iron afforded the 2-phenoxyaniline derivatives **8a**–**d** [18].

In addition, the other diphenyl ether derivatives **7b**–**d** containing nitro groups were reduced using the same method to afford the corresponding amine derivatives **8e**–**g** [11]. Subsequently, the 2-phenoxyaniline derivatives **8a**–**g** were subjected to HATU-mediated coupling reaction with the benzoic acid derivative **4.** The resulting intermediates were then treated with either lithium hydroxide for ester hydrolysis or trifluoroacetic acid for Boc deprotection to afford the linker-functionalized MYC inhibitors **9a**–**g**. Finally, the MYC inhibitors **9a, 9b** and **9e**–**g** were further coupled with different linkers followed by either ester hydrolysis or Boc deprotection to generate the corresponding linker-functionalized MYC inhibitors **10a**–**n**.

**Scheme 3:**
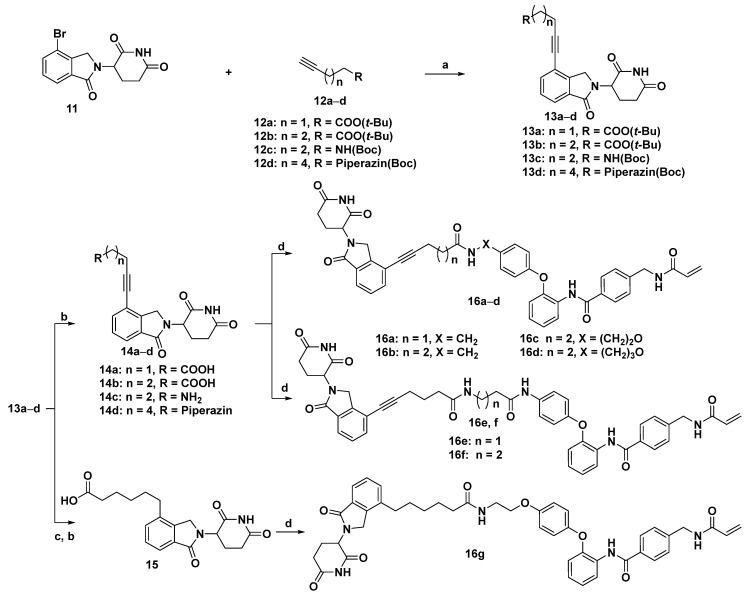
Reagents and conditions: (a) PdCl_2_(PPh_3_)_2_, CuI, TEA, DMF; (b) DCM, TFA; (c) 10% Pd/C, THF; (d) **9a**–**g** or **10a**–**n**, HATU, DIPEA, DMF.

In parallel, several CRBN warheads functionalized bearing different linkers were prepared based upon the scaffolds of known CRBN ligands, including lenalidomide, thalidomide, and other glutarimide derivatives. The lenalidomide-based ligands were synthesized according to the procedure outlined in Scheme 3. Initially, the alkyne-connected lenalidomide derivatives **14a**–**d** were obtained via Sonogashira coupling between the 4-bromolenalidomide derivative **11** and the terminal alkyne linkers **12a**–**d** followed by either Boc deprotection or ester hydrolysis under acidic conditions [19,20]. Similarly, the 4-bromolenalidomide derivative **11** was coupled with the terminal alkyne linker **12b** to afford the alkyne-connected derivative **13b**. Subsequent palladium-catalyzed hydrogenation to saturate the triple bond followed by ester hydrolysis yielded the lenalidomide derivative **15** [21]. Finally, the resulting ligands (**14a**–**d** and **15**) were coupled with the appropriate linker-functionalized inhibitors (**9a**–**g** and **10a**–**n**) to afford the corresponding lenalidomide-based PROTACs **16a**–**g**.

**Scheme 4:**
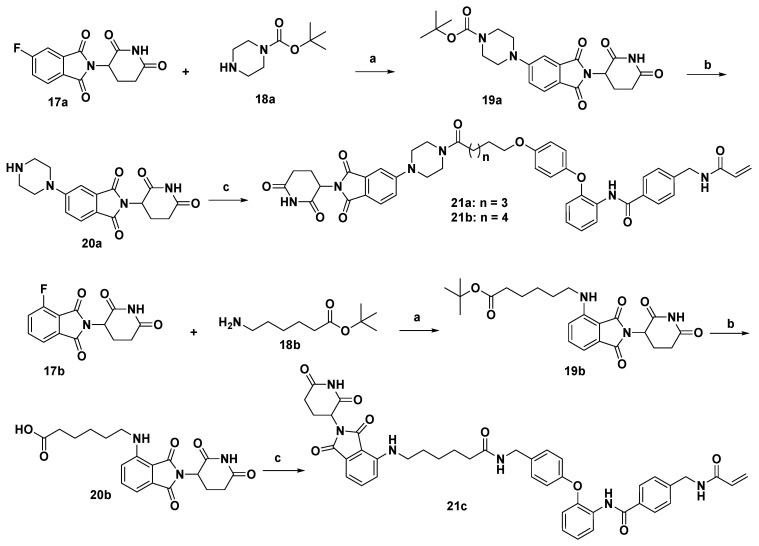
Reagents and conditions: (a) DIPEA, DMSO, 130 °C; (b) DCM, TFA; (c) **9c**–**f**, HATU, DIPEA, DMF.

In addition, the thalidomide-based CRBN ligands **20a** and **20b** were synthesized as outlined in Scheme 4. The 5-fluoro-thalidomide derivative **17a** was reacted with 1-Boc-piperazine **18a** followed by Boc deprotection using trifluoroacetic acid to furnish thalidomide derivative **20a**. Similarly, the 4-fluoro-thalidomide derivative **17b** was reacted with *tert*-butyl 6-aminohexanoate **18b** followed by ester hydrolysis under acidic conditions to afford thalidomide derivative **20b** [22,23]. The resulting ligands **20a** and **20b** were subsequently coupled with the linker-functionalized MYC inhibitors **9c**, **9d**, and **9f** to generate the corresponding thalidomide-based PROTACs **21a**–**c**.

**Scheme 5:**
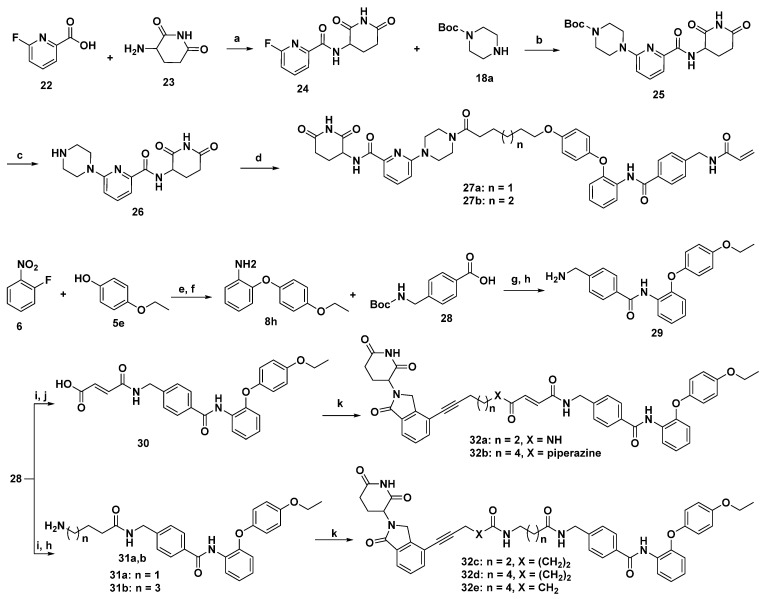
Reagents and conditions: (a) thionyl chloride, acetonitrile, TEA; (b) DIPEA, DMSO, 130 °C; (c) DCM, TFA; (d) **9c** and **9d**, HATU, DIPEA, DMF; (e) K_2_CO_3_, acetonitrile; (f) iron, NH_4_Cl, 96% EtOH; (g) HATU, DIPEA, DMF; (h) 4 M HCl, dioxan; (i) appropriate linker, HATU, DIPEA, DMF; (j) LiOH, H_2_O, THF; (k) **14a**–**d**, HATU, DIPEA, DMF.

In addition, the picolinamide glutarimide derivative **26** was synthesized as depicted in Scheme 5. The 6-fluoropicolinic acid **22** was first coupled with 3-aminopiperidine-2,6-dione **23** to afford the corresponding 6-fluoropicolinamide derivative **24**. This intermediate was subsequently transformed into the piperazine analogue **25** via reaction with 1-Boc-piperazine **18a** followed by Boc deprotection to yield the picolinamide glutarimide derivative **26** [20]. The resulting intermediate was then subjected to amide coupling using the linker-functionalized MYC inhibitors **9c** and **9d**, affording the corresponding PROTACs **27a** and **27b**, respectively.

To further expand this series, additional CRBN-based PROTACs were designed using the acrylamide moiety of EN4 or the propanamide moiety of its saturated analogue EN4-18 as alternative attachment points, as described in Scheme 5. The 2-phenoxy aniline derivative **8h** was prepared via coupling of 4-ethoxyphenol **5e** with 2-fluoronitrobenzene **6** followed by reduction of the resulting nitro intermediate, as previously reported [11]. The resulting aniline derivative **8h** was subsequently coupled with the substituted benzoic acid derivative **28** followed by Boc deprotection to furnish intermediate **29**. Subsequent coupling of several linkers to compound **29** followed by either Boc deprotection or ester hydrolysis, as appropriate, afforded the linker-functionalized inhibitors **30, 31a**, and **31b**. Finally, coupling of these linker-bearing inhibitors with the lenalidomide analogues **14a**–**d** afforded the corresponding PROTACs **32a**–**e**.

**Scheme 6:**
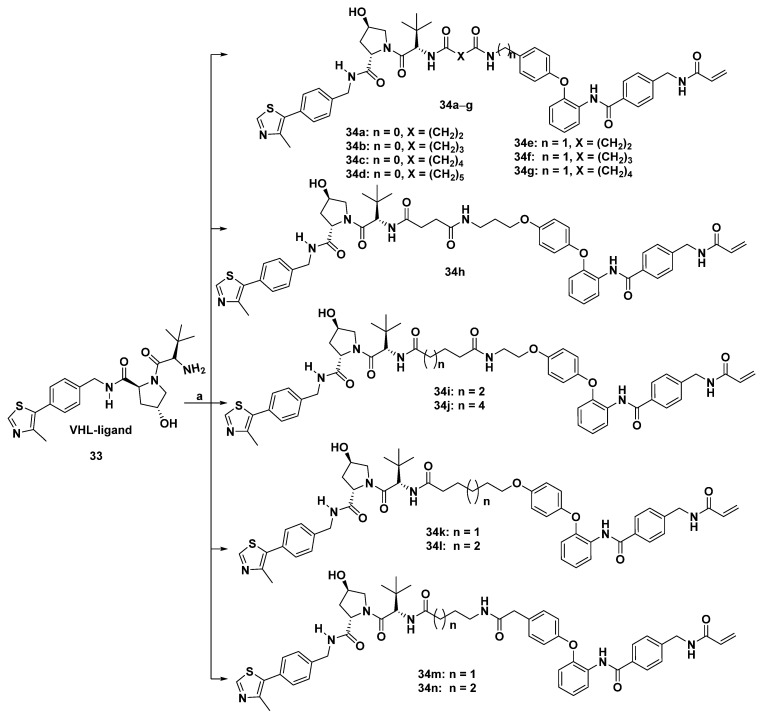
Reagents and conditions: (a) **9c**, **9d** or **10a**–**n**, HATU, DIPEA, DMF.

Finally, the VHL-based PROTACs **34a**–**n** were synthesized by coupling the VHL ligand **33**, prepared according to previously reported procedures, with the linker-functionalized inhibitors **9c**, **9d** and **10a**–**n**, as illustrated in Scheme 6 [20,24].

The structures of all synthesized CRBN- and VHL-based PROTACs are outlined in Table 1 and Table 2, respectively.

### 2.2. Non-Enzymatic Stability Testing

Since some PROTACs have been reported to exhibit chemical instability under cellular assay conditions, we evaluated the stability of selected representative examples of the synthesized PROTACs based on their connected E3 ligase ligand in cellular assay medium (Dulbecco’s modified Eagle medium (DMEM)) using an HPLC-based method [20,25]. The results are summarized in Figure S6, Supplementary Information.

Nearly all tested compounds exhibited relatively high stability under these conditions over 24 h, with the exception of compound **27b**, which was highly unstable and retained only about 50% of its original integrity. In addition, the lenalidomide- and VHL-based PROTACs displayed the highest chemical stability, with no detectable degradation products. In contrast, the thalidomide-based PROTACs showed moderate stability and produced minor degradation products after 24 h.

### 2.3. Microsomal Stability Testing

In order to obtain information on the cellular stability of the compounds and their suitability for further in vivo studies, we tested some of the promising degraders and the references in mouse microsomes. The compounds were incubated with mouse (CD-1) liver microsomes for two hours at 37 degrees Celsius, and the remaining amount of substance was determined by HPLC (Table 3). The compounds underwent rapid degradation, indicating limited use for in vivo studies. Rapid degradation was also observed for the two reference inhibitors KL4-219A and EN4.

### 2.4. Biological Testing

**Figure 3:**
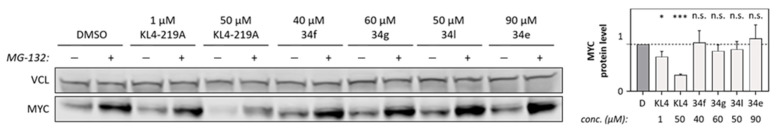
Western blot analysis (*n* = 3) of potential MYC PROTACs. HEK293T cells were treated for 6 h with compounds at indicated concentrations. MG-132 was used as proteasomal inhibitor. Quantification of MYC protein level is depicted on the right side. VCL served as normalization control. D—DMSO, KL4—KL4-219A, * *p* < 0.05, *** *p* < 0.001, n.s.—not significant.

**Figure 4:**
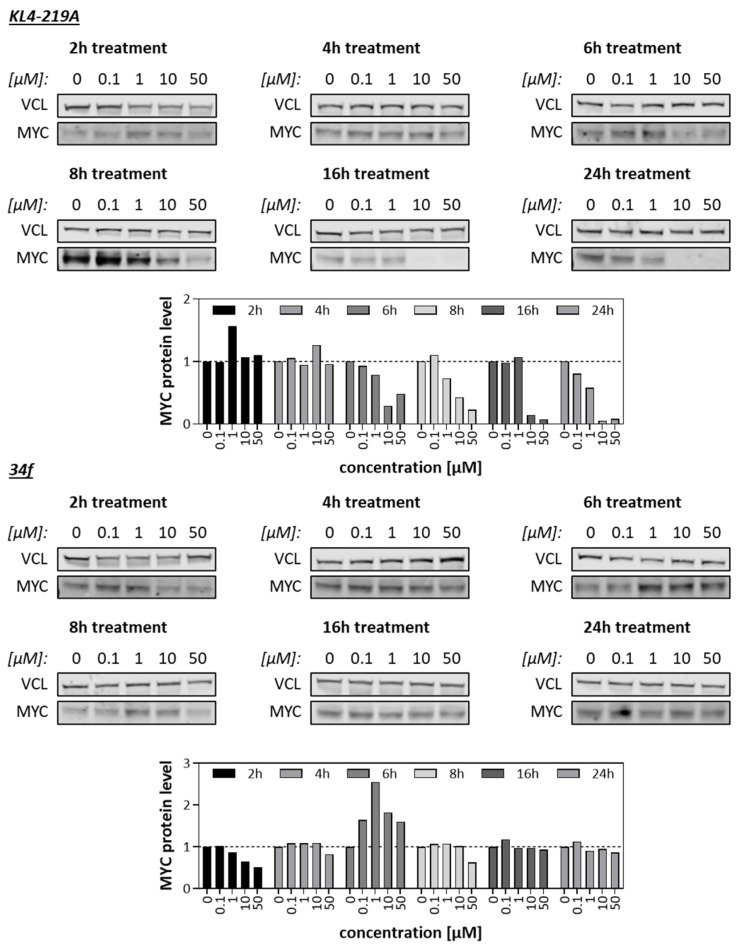
Time and concentration course analysis of KL4-219A and **34f** in HEK293T17 cells analyzed by Western blotting. The bar diagram show quantification of MYC protein levels relative to VCL expression.

Newly synthesized MYC PROTACs were tested on HEK293T, Panc-1 and HCT-116 cells. Firstly, cell viability was assessed after 72 h using a concentration gradient and subsequent EC_50_ determination (Figures S1–S3, Supplementary Information). The degree of inhibition as well as the EC_50_ values, where applicable, are summarized in Table 1 and Table 2. Unfortunately, only a few compounds showed a reduction of cell viability up to 20 µM (Panc-1), 50 µM (HEK293T) or 100 µM (HCT-116). If a reduction was observed, the respective EC_50_ value was in the middle µM range. Complete killing was not achieved with any PROTAC in the analyzed concentration range. Potential reasons for missing inhibition could be low cellular permeability, efflux by transporters, endosomal trapping or rapid metabolic degradation inside cells, among others. Secondly, compounds that showed a reduction in cell viability were tested for protein degradation. Therefore, cells were treated for 6 h at the EC_50_ concentration. None of the tested potential MYC PROTACs resulted in degradation of the protein (Figure 3). In comparison, treatment with the MYC degrader KL4-219A at EC_50_ concentration of 1 μM resulted in approximately 20% reduction of protein. Only by increasing the concentration 50 times to 50 µM was a significant reduction of MYC observed (~67%). A more detailed time and concentration course analysis in HEK293T17 cells revealed overall no degradation at all for compounds **34l** and **34e** (Figure S4, Supplementary Information). KL4-219A showed concentration-dependent MYC degradation over all time points (Figure 4). In contrast, **34f** and **34g** showed slight concentration-dependent MYC degradation only after 2 or 4 h, respectively (Figure 4 and Figure S4, Supplementary Information). We also tested the most active compounds using HEK293T17 cells transiently overexpressing HiBiT-MYC and LgBiT to identify if a luciferase-based approach is more sensitive in detecting MYC degradation. However, all compounds failed to reduce the luciferase activity significantly (Figure S5, Supplementary Information). This suggests rather a crucial failure in cellular entry or ternary complex formation than a simple lack in potency or other mechanistic PROTAC function.

## 3. Materials and Methods

### 3.1. General

All materials and reagents were purchased from chemical suppliers and used without further purification. All solvents were analytically pure and were dried before use. All reactions were monitored by TLC (Kieselgel 60 F254 pre-coated plates, E. Merck, Darmstadt, Germany); the spots were detected by UV lamp at λ 254 nm. For medium-pressure liquid chromatography (MPLC), Biotage SNAP ultra-HP-sphere 20 μm columns containing silica gel were used. Dichloromethane: methanol and n-heptane: ethyl acetate were used as elution systems for MPLC. In the preparative high-pressure liquid chromatography used for purification of several PROTACs, a LiChrosorb^®^ RP-18 (7 μm) 250-25 Merck (Merck, Darmstadt, Germany) column was used. The applied mobile phase was a gradient with increasing polarity composed of acetonitrile/water/formic acid. Purity was determined using HPLC by measuring the UV absorbance at 254 nm. The HPLC consisted of a LiChrosorb^®^ RP-18 (5 μm) 100-4.6 Merck column (Merck, Darmstadt, Germany), two LC-10AD pumps, an SPD-M10A VP PDA detector, and an SIL-HT autosampler, all from the manufacturer Shimadzu (Kyoto, Japan). The absorption spectra were recorded with an SPD-M10A diode array detector Shimadzu spectrophotometer (Kyoto, Japan). Mass spectrometry analyses were performed with a Finnigan MAT710C (Thermo Separation Products, SanJose, CA, USA) for the ESI MS spectra and with an LTQ (linear ion trap) Orbitrap XL hybrid mass spectrometer (Thermo Fisher Scientific, Bremen, Germany) for the HRMS-ESI (high-resolution mass spectrometry) spectra. ^1^HNMR and ^13^CNMR spectra were taken on a Varian Inova 400 using deuterated dimethyl sulfoxide (DMSO-*d*_6_) or deuterated chloroform (CDCl_3_) as solvent. Chemical shifts were referenced to the residual solvent signals. The following abbreviations and formulas for solvents and reagents were used: ethanol (EtOH), *N*,*N*-dimethylformamide (DMF), dimethyl sulfoxide (DMSO), tetrahydrofuran (THF), triethylamine (TEA), water (H_2_O), dichloromethane (DCM), *N*,*N*-diisopropylethylamine (DIPEA), ammonium chloride (NH_4_Cl), potassium carbonate (K_2_CO_3_), O-(7-azabenzotriazol-1-yl)-*N*,*N*,*N′*,*N′*-tetramethyluronium-hexafluorphosphate (HATU), cesium carbonate (Cs_2_CO_3_), bis(triphenylphosphin)palladium(II)-dichloride (PdCl_2_(PPh_3_)_2_), lithium hydroxide (LiOH), copper (I) iodide (CuI), and trifluoroacetic acid (TFA).

### 3.2. General Synthetic Methods

#### 3.2.1. Method I: Amide Coupling

Method I-A: A stirred solution of benzyl amine derivative **1** (1.0 equiv.) and triethylamine (2.5 equiv.) in acetonitrile at 0 °C was treated with acryloyl chloride **2** (1.1 equiv.). After stirring at room temperature for 2 h, the reaction was quenched with 5% acetic acid and extracted with ethyl acetate. The combined organic layers were washed with brine, dried, concentrated, and the crude product purified by MPLC (n-heptane/ethyl acetate).

Method I-B: A mixture of carboxylic acid (1.0 equiv.), DIPEA (4.0 equiv.), and HATU (1.1 equiv.) in DMF was stirred at room temperature for 30 min, followed by addition of the amine (1.0 equiv.). The reaction was stirred at room temperature for 3–12 h, then quenched with 1 M ammonium chloride and extracted with ethyl acetate. The organic layer was washed with 1 M sodium bicarbonate and brine, dried over Na_2_SO_4_, and concentrated. The crude amide was purified by MPLC using n-heptane/ethyl acetate or DCM/MeOH.

#### 3.2.2. Method II: Ester Hydrolysis

Method II-A: A solution of methyl ester-containing intermediate (1.0 equiv.) in THF/H_2_O (3:1) was treated with LiOH·H_2_O (5.0 equiv.) and stirred at room temperature for 4–6 h. After complete hydrolysis, the reaction mixture was acidified with 1 M aqueous HCl to liberate the free acid. The product was extracted with ethyl acetate, and the combined organic layers were washed with brine, dried over Na_2_SO_4_, and concentrated under reduced pressure to afford the corresponding carboxylic acid, which was used directly in the next step without further purification.

Method II-B: The tert-butyl ester-containing intermediate was dissolved or suspended in DCM (5 mL) at 0 °C, followed by drop wise addition of trifluoroacetic acid (5 mL). The mixture was stirred at room temperature for 3–4 h and then concentrated to dryness to give the corresponding carboxylic acid. The crude product was purified by MPLC using a DCM/MeOH solvent system.

#### 3.2.3. Method III: Reaction of Phenols with 2-Fluoronitrobenzene

A mixture of the appropriate phenol derivative (1.0 equiv.; in case of hydroquinone **5a**, 4.0 equiv. was used) and K_2_CO_3_ (4.0 equiv.) in acetonitrile (20 mL) was treated with 2-fluoronitrobenzene **6** (1.0 equiv.) and heated at reflux overnight. After completion, the reaction mixture was quenched with water and extracted with ethyl acetate. The combined organic layers were washed with brine, dried over Na_2_SO_4_, and concentrated. The crude products were purified by MPLC using DCM or n-heptane/ethyl acetate.

#### 3.2.4. Method IV: Reduction of Nitro to Amine Derivatives

To a suspension of nitro intermediate (1.0 equiv.) in 96% ethanol (50 mL) were added iron powder (5.0 equiv.) and ammonium chloride (1.0 equiv.). The mixture was heated at reflux overnight and then filtered through celite, rinsing with methanol. The filtrate was concentrated under reduced pressure, and the residue was partitioned between ethyl acetate and saturated sodium bicarbonate solution. The combined organic layers were dried over anhydrous Na_2_SO_4_, filtered, and concentrated. The crude aniline derivative was used directly in the next step without further purification.

#### 3.2.5. Method V: Boc Deprotection

Method V-A: A solution or suspension of the Boc-protected amine in DCM (5 mL) was cooled to 0 °C, followed by dropwise addition of trifluoroacetic acid (5 mL). The mixture was stirred at room temperature for 1–2 h and then concentrated under reduced pressure to afford the corresponding trifluoroacetate salt, which was used in the next step without further purification.

Method V-B: A solution or suspension of the Boc-protected amine in dioxane (5 mL) was cooled to 0 °C, followed by dropwise addition of 4 M HCl/dioxane (10 mL). The mixture was stirred at room temperature for 5–6 h. The resulting precipitate was filtered, washed with dioxane and then with ether, and dried. The product was obtained as hydrochloride salt and used in the next step without further purification.

#### 3.2.6. Method VI: Sonogashira Coupling

A solution of the 4-bromolenalidomide derivative **11** (1 equiv.), the appropriate alkyne linker (2 equiv.), CuI (0.2 equiv.), and PdCl_2_(PPh_3_)_2_ (0.1 equiv.) in DMF/TEA (1:1, 15 mL) was degassed and purged with argon three times. The mixture was heated at 80 °C for 3–4 h and then concentrated under reduced pressure, and the residue was purified by MPLC using MeOH/DCM.

#### 3.2.7. Method VII: Hydrogenation

A stirred solution of compound **13b** (1 g) in THF (20 mL) was treated with 10% Pd/C (100 mg) and stirred under a hydrogen atmosphere at room temperature overnight. After completion, the reaction was filtered through a short pad of celite, washed with THF, and concentrated to dryness. The crude product was purified by MPLC using MeOH/DCM.

#### 3.2.8. Method VIII: Alkylation Reaction

Compound **7a** (1.0 equiv.) and Cs_2_CO_3_ (1.5 equiv.) in DMF (25 mL) were reacted with the appropriate alkyl bromide (1.1 equiv.) at 80 °C overnight. The mixture was diluted with water and extracted with ethyl acetate, and the combined organic layers were washed with brine, dried over Na_2_SO_4_, and concentrated. The crude product was purified by MPLC using n-heptane/ethyl acetate.

### 3.3. Characterization Data of Key Intermediates and Final Compounds

The preparation and analytical data of intermediates **20a**, **20b** [19,26], **26** [20], and **33** [24] were as reported.

#### 3.3.1. Synthesis and Characterization of Intermediates **3** and **4**

Methyl 4-aminomethyl benzoate **1** was coupled with acryloyl chloride **2** using Method I-A to furnish the acrylamide derivative **3**. Hydrolysis of the resulting ester by Method II-A provided the corresponding carboxylic acid **4**.

*Methyl 4-(acrylamidomethyl)benzoate* (**3**). ^1^H NMR (400 MHz, DMSO-*d*_6_) δ 8.67 (t, *J* = 5.5 Hz, 1H), 7.91 (d, *J* = 8.3 Hz, 2H), 7.38 (d, *J* = 8.4 Hz, 2H), 6.28 (dd, *J* = 17.1, 10.1 Hz, 1H), 6.12 (dd, *J* = 17.1, 2.2 Hz, 1H), 5.62 (dd, *J* = 10.1, 2.2 Hz, 1H), 4.41 (d, *J* = 6.0 Hz, 2H), 3.82 (s, 3H).

*4-(Acrylamidomethyl)benzoic acid* (**4**). ^1^H NMR (400 MHz, DMSO-*d*_6_) δ 12.80 (s, 1H), 8.65 (t, *J* = 5.8 Hz, 1H), 7.88 (d, *J* = 8.3 Hz, 2H), 7.35 (d, *J* = 8.5 Hz, 2H), 6.27 (dd, *J* = 17.1, 10.1 Hz, 1H), 6.12 (dd, *J* = 17.1, 2.2 Hz, 1H), 5.62 (dd, *J* = 10.1, 2.2 Hz, 1H), 4.40 (d, *J* = 6.0 Hz, 2H).

#### 3.3.2. Synthesis and Characterization of Intermediates **7a**–**d**

Using Method III, the diphenyl ether products **7a**–**d** were obtained from the reactions between hydroquinone **5a** or the 4-substituted phenol derivatives **5b**–**d** with 2-fluoronitrobenzene **6**.

*4-(2-Nitrophenoxy)phenol* (**7a**). ^1^H NMR (400 MHz, DMSO-*d*_6_) δ 9.47 (s, 1H), 7.97 (dd, *J* = 8.1, 1.6 Hz, 1H), 7.63–7.55 (m, 1H), 7.27–7.18 (m, 1H), 6.98–6.90 (m, 3H), 6.84–6.75 (m, 2H).

*tert-Butyl (4-(2-nitrophenoxy)phenyl)carbamate* (**7b**). ^1^H NMR (400 MHz, DMSO-*d*_6_) δ 9.38 (s, 1H), 7.99 (d, *J* = 8.1 Hz, 1H), 7.66–7.56 (m, 1H), 7.48 (d, *J* = 8.5 Hz, 2H), 7.30–7.23 (m, 1H), 7.00 (d, *J* = 8.7 Hz, 3H), 1.45 (s, 9H).

*tert-Butyl (4-(2-nitrophenoxy)benzyl)carbamate* (**7c**). ^1^H NMR (400 MHz, DMSO-*d*_6_) δ 8.03 (dd, *J* = 8.2, 1.6 Hz, 1H), 7.69–7.62 (m, 1H), 7.42–7.30 (m, 2H), 7.27 (d, *J* = 8.6 Hz, 2H), 7.08 (dd, *J* = 8.4, 0.9 Hz, 1H), 7.01 (d, *J* = 8.6 Hz, 2H), 4.11 (d, *J* = 6.1 Hz, 2H), 1.38 (s, 9H).

*Methyl 2-(4-(2-nitrophenoxy)phenyl)acetate* (**7d**). ^1^H NMR (400 MHz, DMSO-*d*_6_) δ 8.04 (dd, *J* = 8.1, 1.5 Hz, 1H), 7.72–7.63 (m, 1H), 7.39–7.26 (m, 3H), 7.12 (d, *J* = 7.6 Hz, 1H), 7.01 (d, *J* = 8.5 Hz, 2H), 3.67 (s, 2H), 3.61 (s, 3H).

#### 3.3.3. Synthesis and Characterization of Intermediates **8a**–**d**

The 4-(2-nitrophenoxy)phenol **7a** was alkylated with various alkyl bromides following Method VIII, and the resulting nitro compounds were subsequently reduced according to Method IV to yield the 2-phenoxyaniline derivatives **8a**–**d**.

*tert-Butyl (2-(4-(2-aminophenoxy)phenoxy)ethyl)carbamate* (**8a**). ^1^H NMR (400 MHz, DMSO-*d*_6_) δ 6.95 (t, *J* = 5.2 Hz, 1H), 6.86 (q, *J* = 9.3 Hz, 5H), 6.76 (dd, *J* = 7.9, 1.7 Hz, 1H), 6.65 (dd, *J* = 7.9, 1.3 Hz, 1H), 6.52–6.44 (m, 1H), 4.84 (s, 2H), 3.89 (t, *J* = 5.9 Hz, 2H), 3.25 (dd, *J* = 5.8, 5.8 Hz, 2H), 1.36 (s, 9H).

*tert-Butyl (3-(4-(2-aminophenoxy)phenoxy)propyl)carbamate* (**8b**). ^1^H NMR (400 MHz, DMSO-*d*_6_) δ 6.90–6.80 (m, 6H), 6.76 (dd, *J* = 7.9, 1.6 Hz, 1H), 6.65 (dd, *J* = 7.9, 1.1 Hz, 1H), 6.51–6.45 (m, 1H), 4.84 (s, 2H), 3.90 (t, *J* = 6.3 Hz, 2H), 3.06 (dd, *J* = 6.7, 6.7 Hz, 2H), 1.85–1.73 (m, 2H), 1.36 (s, 9H).

*Methyl 6-(4-(2-aminophenoxy)phenoxy)hexanoate* (**8c**). ^1^H NMR (400 MHz, DMSO-*d*_6_) δ 6.90–6.80 (m, 5H), 6.75 (dd, *J* = 7.9, 1.6 Hz, 1H), 6.65 (dd, *J* = 8.0, 1.3 Hz, 1H), 6.53–6.43 (m, 1H), 4.84 (s, 2H), 3.88 (t, *J* = 6.5 Hz, 2H), 3.56 (s, 3H), 2.31 (t, *J* = 7.4 Hz, 2H), 1.72–1.63 (m, 2H), 1.62–1.50 (m, 2H), 1.45–1.32 (m, 2H).

*Methyl 7-(4-(2-aminophenoxy)phenoxy)heptanoate* (**8d**). ^1^H NMR (400 MHz, DMSO-*d*_6_) δ 6.93–6.79 (m, 5H), 6.75 (dd, *J* = 7.9, 1.6 Hz, 1H), 6.65 (dd, *J* = 7.9, 1.2 Hz, 1H), 6.52–6.44 (m, 1H), 4.84 (s, 2H), 3.88 (t, *J* = 6.4 Hz, 2H), 3.56 (s, 3H), 2.28 (t, *J* = 7.4 Hz, 2H), 1.72–1.60 (m, 2H), 1.58–1.47 (m, 2H), 1.43–1.22 (m, 4H).

#### 3.3.4. Synthesis and Characterization of Intermediates **8e**–**g**

The nitro-containing diphenyl ether derivatives **7b**–**d** were reduced to the corresponding amine derivatives **8e**–**g** using Method IV.

*tert-Butyl (4-(2-aminophenoxy)phenyl)carbamate* (**8e**). ^1^H NMR (400 MHz, DMSO-*d*_6_) δ 9.21 (s, 1H), 7.38 (d, *J* = 8.6 Hz, 2H), 6.89–6.73 (m, 4H), 6.67 (dd, *J* = 8.0, 1.4 Hz, 1H), 6.54–6.45 (m, 1H), 4.83 (s, 2H), 1.45 (s, 9H).

*tert-Butyl (4-(2-aminophenoxy)benzyl)carbamate* (**8f**). ^1^H NMR (400 MHz, DMSO-*d*_6_) δ 7.29 (t, *J* = 5.9 Hz, 1H), 7.16 (d, *J* = 8.5 Hz, 2H), 6.92–6.85 (m, 1H), 6.82 (d, *J* = 8.5 Hz, 2H), 6.78 (dd, *J* = 7.9, 1.6 Hz, 1H), 6.72 (dd, *J* = 7.8, 1.4 Hz, 1H), 6.56–6.47 (m, 1H), 4.82 (s, 2H), 4.05 (d, *J* = 6.6 Hz, 2H), 1.36 (s, 9H).

*Methyl 2-(4-(2-aminophenoxy)phenyl)acetate* (**8g**). ^1^H NMR (400 MHz, DMSO-*d*_6_) δ 7.18 (d, *J* = 8.6 Hz, 2H), 6.93–6.87 (m, 1H), 6.86–6.73 (m, 4H), 6.57–6.48 (m, 1H), 4.86 (s, 2H), 3.60 (s, 2H), 3.59 (s, 3H).

#### 3.3.5. Synthesis and Characterization of Intermediates **8h**

4-Ethoxyphenol **5e** was reacted with 2-fluoronitrobenzene **6** under Method III conditions, and the resulting nitro intermediate was subsequently reduced using Method IV to give the 2-phenoxyaniline derivative **8h.**

*2-(4-Ethoxyphenoxy)aniline* (8**h**). ^1^H NMR (400 MHz, DMSO-*d*_6_) δ 6.91–6.80 (m, 5H), 6.76 (dd, *J* = 7.9, 1.6 Hz, 1H), 6.65 (dd, *J* = 7.9, 1.4 Hz, 1H), 6.53–6.44 (m, 1H), 4.84 (s, 2H), 3.95 (q, *J* = 7.0 Hz, 2H), 1.28 (t, *J* = 7.0 Hz, 3H).

#### 3.3.6. Synthesis and Characterization of Intermediates **9a**–**g**

The 2-phenoxyaniline derivatives **8a**–**g** were coupled with benzoic acid derivative **4** using Method I-B, and the resulting intermediates were subjected to either ester hydrolysis (Method II-A) or Boc deprotection (Method V-A) to afford the linker-connected inhibitors **9a**–**g**.

*4-(Acrylamidomethyl)-N-(2-(4-(2-aminoethoxy)phenoxy)phenyl)benzamide* (**9a**). ^1^H NMR (400 MHz, DMSO-*d*_6_) δ 9.72 (s, 1H), 8.68 (t, *J* = 6.0 Hz, 1H), 8.00 (s, 3H), 7.81 (d, *J* = 8.3 Hz, 2H), 7.73 (dd, *J* = 7.6, 2.1 Hz, 1H), 7.34 (d, *J* = 8.2 Hz, 2H), 7.21–7.08 (m, 2H), 7.02–6.92 (m, 4H), 6.85 (dd, *J* = 7.8, 1.7 Hz, 1H), 6.28 (dd, *J* = 17.1, 10.1 Hz, 1H), 6.11 (dd, *J* = 17.1, 2.2 Hz, 1H), 5.62 (dd, *J* = 10.1, 2.2 Hz, 1H), 4.39 (d, *J* = 6.0 Hz, 2H), 4.09 (t, *J* = 5.1 Hz, 2H), 3.21–3.12 (m, 2H).

*4-(Acrylamidomethyl)-N-(2-(4-(3-aminopropoxy)phenoxy)phenyl)benzamide* (**9b**). ^1^H NMR (400 MHz, DMSO-*d*_6_) δ 9.71 (s, 1H), 8.68 (t, *J* = 6.0 Hz, 1H), 7.82 (d, *J* = 8.2 Hz, 5H), 7.73 (dd, *J* = 7.8, 1.9 Hz, 1H), 7.34 (d, *J* = 8.2 Hz, 2H), 7.20–7.07 (m, 2H), 7.03–6.87 (m, 4H), 6.83 (dd, *J* = 7.7, 1.7 Hz, 1H), 6.28 (dd, *J* = 17.1, 10.1 Hz, 1H), 6.11 (dd, *J* = 17.1, 2.2 Hz, 1H), 5.62 (dd, *J* = 10.1, 2.2 Hz, 1H), 4.39 (d, *J* = 6.0 Hz, 2H), 3.99 (t, *J* = 6.0 Hz, 2H), 2.99–2.90 (m, 2H), 2.02–1.91 (m, 2H).

*6-(4-(2-(4-(Acrylamidomethyl)benzamido)phenoxy)phenoxy)hexanoic acid* (**9c**). ^1^H NMR (400 MHz, DMSO-*d*_6_) δ 11.96 (br, 1H), 9.68 (s, 1H), 8.64 (t, *J* = 5.9 Hz, 1H), 7.85–7.79 (m, 2H), 7.74 (dd, *J* = 7.5, 2.0 Hz, 1H), 7.39–7.29 (m, 2H), 7.20–7.06 (m, 2H), 6.97–6.87 (m, 4H), 6.83 (dd, *J* = 7.8, 1.7 Hz, 1H), 6.27 (dd, *J* = 17.1, 10.1 Hz, 1H), 6.12 (dd, *J* = 17.1, 2.2 Hz, 1H), 5.61 (dd, *J* = 10.1, 2.2 Hz, 1H), 4.39 (d, *J* = 6.0 Hz, 2H), 3.89 (t, *J* = 6.4 Hz, 2H), 2.21 (t, *J* = 7.3 Hz, 2H), 1.75–1.61 (m, 2H), 1.60–1.48 (m, 2H), 1.45–1.32 (m, 2H).

*7-(4-(2-(4-(Acrylamidomethyl)benzamido)phenoxy)phenoxy)heptanoic acid* (**9d**). ^1^H NMR (400 MHz, DMSO-*d*_6_) δ 11.98 (br, 1H), 9.67 (s, 1H), 8.64 (t, *J* = 5.8 Hz, 1H), 7.81 (d, *J* = 8.2 Hz, 2H), 7.74 (dd, *J* = 7.6, 2.0 Hz, 1H), 7.34 (d, *J* = 8.2 Hz, 2H), 7.19–7.06 (m, 2H), 6.98–6.87 (m, 4H), 6.83 (dd, *J* = 7.8, 1.7 Hz, 1H), 6.27 (dd, *J* = 17.1, 10.1 Hz, 1H), 6.11 (dd, *J* = 17.1, 2.2 Hz, 1H), 5.61 (dd, *J* = 10.1, 2.2 Hz, 1H), 4.39 (d, *J* = 6.0 Hz, 2H), 3.89 (t, *J* = 6.4 Hz, 2H), 2.18 (t, *J* = 7.3 Hz, 2H), 1.71–1.60 (m, 2H), 1.55–1.45 (m, 2H), 1.43–1.25 (m, 4H).

*4-(Acrylamidomethyl)-N-(2-(4-aminophenoxy)phenyl)benzamide* (**9e**). ^1^H NMR (400 MHz, DMSO-*d*_6_) δ 9.76 (s, 1H), 8.66 (t, *J* = 5.9 Hz, 1H), 7.78–7.67 (m, 3H), 7.31 (d, *J* = 8.1 Hz, 2H), 7.27–7.09 (m, 4H), 7.04–6.94 (m, 3H), 6.27 (dd, *J* = 17.1, 10.1 Hz, 1H), 6.11 (dd, *J* = 17.1, 2.0 Hz, 1H), 5.62 (dd, *J* = 10.1, 2.1 Hz, 1H), 4.37 (d, *J* = 6.0 Hz, 2H).

*4-(Acrylamidomethyl)-N-(2-(4-(aminomethyl)phenoxy)phenyl)benzamide* (**9f**). ^1^H NMR (400 MHz, DMSO-*d*_6_) δ 9.78 (s, 1H), 8.67 (t, *J* = 6.0 Hz, 1H), 8.11 (s, 3H), 7.80–7.68 (m, 3H), 7.39 (d, *J* = 8.7 Hz, 2H), 7.31 (d, *J* = 8.4 Hz, 2H), 7.26–7.18 (m, 2H), 7.03–6.93 (m, 3H), 6.27 (dd, *J* = 17.1, 10.1 Hz, 1H), 6.11 (dd, *J* = 17.1, 2.2 Hz, 1H), 5.62 (dd, *J* = 10.1, 2.2 Hz, 1H), 4.37 (d, *J* = 6.0 Hz, 2H), 3.96 (q, *J* = 5.5 Hz, 2H).

*2-(4-(2-(4-(Acrylamidomethyl)benzamido)phenoxy)phenyl)acetic acid* (**9g**). ^1^H NMR (400 MHz, DMSO-*d*_6_) δ 12.25 (s, 1H), 9.71 (s, 1H), 8.63 (t, *J* = 5.9 Hz, 1H), 7.79–7.70 (m, 3H), 7.32 (d, *J* = 8.2 Hz, 2H), 7.25–7.12 (m, 4H), 6.99–6.88 (m, 3H), 6.27 (dd, *J* = 17.1, 10.1 Hz, 1H), 6.11 (dd, *J* = 17.1, 2.2 Hz, 1H), 5.61 (dd, *J* = 10.1, 2.2 Hz, 1H), 4.38 (d, *J* = 6.0 Hz, 2H), 3.50 (s, 2H).

#### 3.3.7. Synthesis and Characterization of Intermediates **10a**–**n**

MYC inhibitors **9a**, **9b**, and **9e**–**g** were coupled with different linkers using Method I-B, followed by ester hydrolysis (Method II-A) or Boc deprotection (Method V-A) to afford the linker-connected inhibitors **10a**–**n**.

*6-((2-(4-(2-(4-(Acrylamidomethyl)benzamido)phenoxy)phenoxy)ethyl)amino)-6-oxohexanoic acid* (**10a**). ^1^H NMR (400 MHz, DMSO-*d*_6_) δ 11.94 (br, 1H), 9.68 (s, 1H), 8.64 (t, *J* = 5.9 Hz, 1H), 8.00 (t, *J* = 5.4 Hz, 1H), 7.81 (d, *J* = 8.0 Hz, 2H), 7.77–7.70 (m, 1H), 7.34 (d, *J* = 8.0 Hz, 2H), 7.20–7.07 (m, 2H), 6.98–6.89 (m, 4H), 6.86–6.80 (m, 1H), 6.27 (dd, *J* = 17.1, 10.1 Hz, 1H), 6.11 (dd, *J* = 17.1, 2.1 Hz, 1H), 5.61 (dd, *J* = 10.1, 2.1 Hz, 1H), 4.39 (d, *J* = 6.0 Hz, 2H), 3.91 (t, *J* = 5.6 Hz, 2H), 3.41–3.34 (m, 2H), 2.17 (t, *J* = 6.6 Hz, 2H), 2.07 (t, *J* = 6.7 Hz, 2H), 1.54–1.38 (m, 4H).

*8-((2-(4-(2-(4-(Acrylamidomethyl)benzamido)phenoxy)phenoxy)ethyl)amino)-8-oxooctanoic acid* (**10b**). ^1^H NMR (400 MHz, DMSO-*d*_6_) δ 11.93 (s, 1H), 9.68 (s, 1H), 8.64 (t, *J* = 6.0 Hz, 1H), 7.98 (t, *J* = 5.5 Hz, 1H), 7.81 (d, *J* = 8.3 Hz, 2H), 7.74 (dd, *J* = 7.6, 2.0 Hz, 1H), 7.34 (d, *J* = 8.2 Hz, 2H), 7.19–7.06 (m, 2H), 6.99–6.87 (m, 4H), 6.83 (dd, *J* = 7.7, 1.8 Hz, 1H), 6.27 (dd, *J* = 17.1, 10.1 Hz, 1H), 6.11 (dd, *J* = 17.1, 2.2 Hz, 1H), 5.61 (dd, *J* = 10.1, 2.2 Hz, 1H), 4.39 (d, *J* = 6.0 Hz, 2H), 3.91 (t, *J* = 5.7 Hz, 2H), 3.36 (dd, *J* = 5.7, 5.7 Hz, 2H), 2.15 (t, *J* = 7.3 Hz, 2H), 2.05 (t, *J* = 7.4 Hz, 2H), 1.52–1.38 (m, 4H), 1.29–1.15 (m, 4H).

*4-((3-(4-(2-(4-(Acrylamidomethyl)benzamido)phenoxy)phenoxy)propyl)amino)-4-oxobutanoic acid* (**10c**). ^1^H NMR (400 MHz, DMSO-*d*_6_) δ 12.03 (s, 1H), 9.68 (s, 1H), 8.65 (t, *J* = 5.9 Hz, 1H), 7.89 (t, *J* = 5.4 Hz, 1H), 7.82 (d, *J* = 8.2 Hz, 2H), 7.74 (dd, *J* = 7.6, 1.6 Hz, 1H), 7.34 (d, *J* = 8.2 Hz, 2H), 7.19–7.06 (m, 2H), 6.98–6.87 (m, 4H), 6.83 (dd, *J* = 7.9, 1.4 Hz, 1H), 6.27 (dd, *J* = 17.1, 10.1 Hz, 1H), 6.11 (dd, *J* = 17.1, 2.1 Hz, 1H), 5.61 (dd, *J* = 10.1, 2.1 Hz, 1H), 4.39 (d, *J* = 6.0 Hz, 2H), 3.91 (t, *J* = 6.3 Hz, 2H), 3.16 (dd, *J* = 6.3, 6.3 Hz, 2H), 2.40 (t, *J* = 6.9 Hz, 2H), 2.28 (t, *J* = 6.9 Hz, 2H), 1.85–1.74 (m, 2H).

*4-(Acrylamidomethyl)-N-(2-(4-(3-aminopropanamido)phenoxy)phenyl)benzamide* (**10d**). ^1^H NMR (400 MHz, DMSO-*d*_6_) δ 10.15 (s, 1H), 9.72 (s, 1H), 8.68 (t, *J* = 6.0 Hz, 1H), 7.89–7.70 (m, 5H), 7.54 (d, *J* = 9.0 Hz, 2H), 7.33 (d, *J* = 8.3 Hz, 2H), 7.23–7.10 (m, 2H), 6.99–6.88 (m, 3H), 6.28 (dd, *J* = 17.1, 10.1 Hz, 1H), 6.11 (dd, *J* = 17.1, 2.2 Hz, 1H), 5.61 (dd, *J* = 10.1, 2.2 Hz, 1H), 4.38 (d, *J* = 6.0 Hz, 2H), 3.12–3.02 (m, 2H), 2.67 (t, *J* = 6.8 Hz, 2H).

*4-(Acrylamidomethyl)-N-(2-(4-(4-aminobutanamido)phenoxy)phenyl)benzamide* (**10e**). ^1^H NMR (400 MHz, DMSO-*d*_6_) δ 9.97 (s, 1H), 9.71 (s, 1H), 8.67 (t, *J* = 6.0 Hz, 1H), 7.89–7.70 (m, 6H), 7.54 (d, *J* = 9.0 Hz, 2H), 7.33 (d, *J* = 8.3 Hz, 2H), 7.22–7.10 (m, 2H), 6.99–6.87 (m, 3H), 6.28 (dd, *J* = 17.1, 10.1 Hz, 1H), 6.11 (dd, *J* = 17.1, 2.2 Hz, 1H), 5.61 (dd, *J* = 10.1, 2.2 Hz, 1H), 4.38 (d, *J* = 6.0 Hz, 2H), 2.89–2.77 (m, 2H), 2.38 (t, *J* = 7.2 Hz, 2H), 1.89–1.77 (m, 2H).

*4-((4-(2-(4-(Acrylamidomethyl)benzamido)phenoxy)phenyl)amino)-4-oxobutanoic acid* (**10f**). ^1^H NMR (400 MHz, DMSO-*d*_6_) δ 12.08 (s, 1H), 9.91 (s, 1H), 9.70 (s, 1H), 8.63 (t, *J* = 6.0 Hz, 1H), 7.80 (d, *J* = 8.3 Hz, 2H), 7.74 (dd, *J* = 7.5, 2.1 Hz, 1H), 7.53 (d, *J* = 9.0 Hz, 2H), 7.33 (d, *J* = 8.3 Hz, 2H), 7.21–7.09 (m, 2H), 6.97–6.86 (m, 3H), 6.27 (dd, *J* = 17.1, 10.1 Hz, 1H), 6.11 (dd, *J* = 17.1, 2.2 Hz, 1H), 5.61 (dd, *J* = 10.1, 2.2 Hz, 1H), 4.38 (d, *J* = 6.0 Hz, 2H), 2.53–2.49 (m, 4H).

*5-((4-(2-(4-(Acrylamidomethyl)benzamido)phenoxy)phenyl)amino)-5-oxopentanoic acid* (**10g**). ^1^H NMR (400 MHz, DMSO-*d*_6_) δ 12.04 (s, 1H), 9.85 (s, 1H), 9.70 (s, 1H), 8.63 (t, *J* = 5.9 Hz, 1H), 7.79 (d, *J* = 8.3 Hz, 2H), 7.74 (dd, *J* = 7.5, 2.0 Hz, 1H), 7.54 (d, *J* = 9.0 Hz, 2H), 7.33 (d, *J* = 8.3 Hz, 2H), 7.22–7.09 (m, 2H), 6.97–6.86 (m, 3H), 6.27 (dd, *J* = 17.1, 10.1 Hz, 1H), 6.11 (dd, *J* = 17.1, 2.2 Hz, 1H), 5.61 (dd, *J* = 10.1, 2.2 Hz, 1H), 4.38 (d, *J* = 6.0 Hz, 2H), 2.30 (t, *J* = 7.4 Hz, 2H), 2.25 (t, *J* = 7.3 Hz, 2H), 1.84–1.72 (m, 2H).

*6-((4-(2-(4-(Acrylamidomethyl)benzamido)phenoxy)phenyl)amino)-6-oxohexanoic acid* (**10h**). ^1^H NMR (400 MHz, DMSO-*d*_6_) δ 12.00 (s, 1H), 9.83 (s, 1H), 9.69 (s, 1H), 8.63 (t, *J* = 5.9 Hz, 1H), 7.80 (d, *J* = 8.3 Hz, 2H), 7.74 (dd, *J* = 7.5, 2.0 Hz, 1H), 7.54 (d, *J* = 9.0 Hz, 2H), 7.33 (d, *J* = 8.3 Hz, 2H), 7.21–7.09 (m, 2H), 6.97–6.86 (m, 3H), 6.27 (dd, *J* = 17.1, 10.1 Hz, 1H), 6.11 (dd, *J* = 17.1, 2.2 Hz, 1H), 5.61 (dd, *J* = 10.1, 2.2 Hz, 1H), 4.38 (d, *J* = 6.0 Hz, 2H), 2.30–2.18 (m, 4H), 1.63–1.44 (m, 4H).

*7-((4-(2-(4-(Acrylamidomethyl)benzamido)phenoxy)phenyl)amino)-7-oxoheptanoic acid* (**10i**). ^1^H NMR (400 MHz, DMSO-*d*_6_) δ 11.96 (s, 1H), 9.81 (s, 1H), 9.70 (s, 1H), 8.63 (t, *J* = 5.8 Hz, 1H), 7.83–7.71 (m, 3H), 7.54 (d, *J* = 8.9 Hz, 2H), 7.33 (d, *J* = 8.1 Hz, 2H), 7.22–7.09 (m, 2H), 6.99–6.84 (m, 3H), 6.27 (dd, *J* = 17.1, 10.1 Hz, 1H), 6.11 (dd, *J* = 17.1, 2.1 Hz, 1H), 5.61 (dd, *J* = 10.1, 2.1 Hz, 1H), 4.39 (d, *J* = 5.9 Hz, 2H), 2.25 (t, *J* = 7.4 Hz, 2H), 2.19 (t, *J* = 7.4 Hz, 2H), 1.62–1.44 (m, 4H), 1.32–1.22 (m, 2H).

*4-((4-(2-(4-(Acrylamidomethyl)benzamido)phenoxy)benzyl)amino)-4-oxobutanoic acid* (**10j**). ^1^H NMR (400 MHz, DMSO-*d*_6_) δ 12.06 (s, 1H), 9.71 (s, 1H), 8.64 (t, *J* = 6.0 Hz, 1H), 8.30 (t, *J* = 6.0 Hz, 1H), 7.81–7.69 (m, 3H), 7.32 (d, *J* = 8.3 Hz, 2H), 7.24–7.12 (m, 4H), 6.98–6.87 (m, 3H), 6.27 (dd, *J* = 17.1, 10.1 Hz, 1H), 6.11 (dd, *J* = 17.1, 2.2 Hz, 1H), 5.61 (dd, *J* = 10.1, 2.2 Hz, 1H), 4.38 (d, *J* = 6.0 Hz, 2H), 4.19 (d, *J* = 5.9 Hz, 2H), 2.46–2.31 (m, 4H).

*5-((4-(2-(4-(Acrylamidomethyl)benzamido)phenoxy)benzyl)amino)-5-oxopentanoic acid* (**10k**). ^1^H NMR (400 MHz, DMSO-*d*_6_) δ 12.00 (s, 1H), 9.71 (s, 1H), 8.63 (t, *J* = 5.9 Hz, 1H), 8.26 (t, *J* = 5.9 Hz, 1H), 7.81–7.69 (m, 3H), 7.32 (d, *J* = 8.4 Hz, 2H), 7.23–7.12 (m, 4H), 6.96–6.87 (m, 3H), 6.27 (dd, *J* = 17.1, 10.1 Hz, 1H), 6.11 (dd, *J* = 17.1, 2.2 Hz, 1H), 5.61 (dd, *J* = 10.1, 2.2 Hz, 1H), 4.38 (d, *J* = 6.0 Hz, 2H), 4.18 (d, *J* = 5.9 Hz, 2H), 2.19 (t, *J* = 7.4 Hz, 2H), 2.13 (t, *J* = 7.4 Hz, 2H), 1.77–1.65 (m, 2H).

*6-((4-(2-(4-(Acrylamidomethyl)benzamido)phenoxy)benzyl)amino)-6-oxohexanoic acid* (**10l**). ^1^H NMR (400 MHz, DMSO-*d*_6_) δ 11.97 (s, 1H), 9.71 (s, 1H), 8.63 (t, *J* = 6.0 Hz, 1H), 8.24 (t, *J* = 5.9 Hz, 1H), 7.81–7.70 (m, 3H), 7.32 (d, *J* = 8.2 Hz, 2H), 7.23–7.12 (m, 4H), 6.97–6.87 (m, 3H), 6.27 (dd, *J* = 17.1, 10.1 Hz, 1H), 6.11 (dd, *J* = 17.1, 2.2 Hz, 1H), 5.61 (dd, *J* = 10.1, 2.2 Hz, 1H), 4.38 (d, *J* = 6.0 Hz, 2H), 4.19 (d, *J* = 5.9 Hz, 2H), 2.18 (t, *J* = 7.0 Hz, 2H), 2.10 (t, *J* = 7.0 Hz, 2H), 1.57–1.40 (m, 4H).

*4-(2-(4-(2-(4-(Acrylamidomethyl)benzamido)phenoxy)phenyl)acetamido)butanoic acid* (**10m**). ^1^H NMR (400 MHz, DMSO-*d*_6_) δ 12.06 (s, 1H), 9.70 (s, 1H), 8.63 (t, *J* = 5.9 Hz, 1H), 7.99 (t, *J* = 5.5 Hz, 1H), 7.80–7.70 (m, 3H), 7.32 (d, *J* = 8.2 Hz, 2H), 7.24–7.11 (m, 4H), 6.98–6.86 (m, 3H), 6.27 (dd, *J* = 17.1, 10.1 Hz, 1H), 6.11 (dd, *J* = 17.1, 2.2 Hz, 1H), 5.61 (dd, *J* = 10.1, 2.2 Hz, 1H), 4.38 (d, *J* = 6.0 Hz, 2H), 3.32 (s, 2H), 3.02 (dd, *J* = 6.8, 6.8 Hz, 2H), 2.18 (t, *J* = 7.4 Hz, 2H), 1.65–1.53 (m, 2H).

*5-(2-(4-(2-(4-(Acrylamidomethyl)benzamido)phenoxy)phenyl)acetamido)pentanoic acid* (**10n**). ^1^H NMR (400 MHz, DMSO-*d*_6_) δ 12.02 (br, 1H), 9.70 (s, 1H), 8.63 (t, *J* = 5.9 Hz, 1H), 7.96 (t, *J* = 5.5 Hz, 1H), 7.81–7.70 (m, 3H), 7.32 (d, *J* = 8.2 Hz, 2H), 7.24–7.11 (m, 4H), 6.98–6.86 (m, 3H), 6.27 (dd, *J* = 17.1, 10.1 Hz, 1H), 6.11 (dd, *J* = 17.1, 2.2 Hz, 1H), 5.61 (dd, *J* = 10.1, 2.2 Hz, 1H), 4.38 (d, *J* = 6.0 Hz, 2H), 3.32 (s, 2H), 3.01 (dd, *J* = 6.6, 6.6 Hz, 2H), 2.17 (t, *J* = 7.2 Hz, 2H), 1.52–1.32 (m, 4H).

#### 3.3.8. Synthesis and Characterization of Intermediates **14a**–**d**

The 4-bromolenalidomide derivative **11** was coupled with alkyne linkers **12a**–**d** using Method VI, and the resulting products were subjected to either Boc deprotection (Method V-A) or ester hydrolysis (Method II-B) to afford the alkyne-linked lenalidomide derivatives **14a**–**d**.

*5-(2-(2,6-Dioxopiperidin-3-yl)-1-oxoisoindolin-4-yl)pent-4-ynoic acid* (**14a**). ^1^H NMR (400 MHz, DMSO-*d_6_*) δ 12.36 (s, 1H), 10.98 (s, 1H), 7.73–7.66 (m, 1H), 7.60 (dd, *J* = 7.6, 0.8 Hz, 1H), 7.50 (t, *J* = 7.6 Hz, 1H), 5.13 (dd, *J* = 13.3, 5.1 Hz, 1H), 4.41 (d, *J* = 17.8 Hz, 1H), 4.26 (d, *J* = 17.8 Hz, 1H), 2.98–2.84 (m, 1H), 2.71–2.51 (m, 5H), 2.46–2.37 (m, 1H), 2.06–1.95 (m, 1H).

*6-(2-(2,6-Dioxopiperidin-3-yl)-1-oxoisoindolin-4-yl)hex-5-ynoic acid* (**14b**). ^1^H NMR (400 MHz, DMSO-*d_6_*) δ 12.14 (br, 1H), 10.96 (s, 1H), 7.69 (dd, *J* = 7.6, 0.8 Hz, 1H), 7.63 (dd, *J* = 7.6, 0.9 Hz, 1H), 7.50 (t, *J* = 7.6 Hz, 1H), 5.12 (dd, *J* = 13.3, 5.1 Hz, 1H), 4.44 (d, *J* = 17.7 Hz, 1H), 4.30 (d, *J* = 17.7 Hz, 1H), 2.96–2.83 (m, 1H), 2.66–2.49 (m, 3H), 2.46–2.27 (m, 3H), 2.05–1.94 (m, 1H), 1.85–1.69 (m, 2H).

*3-(4-(5-Aminopent-1-yn-1-yl)-1-oxoisoindolin-2-yl)piperidine-2,6-dione* (**14c**). ^1^H NMR (400 MHz, DMSO-*d_6_*) δ 10.98 (s, 1H), 7.88 (br, 3H), 7.71 (d, *J* = 7.5 Hz, 1H), 7.63 (d, *J* = 7.6 Hz, 1H), 7.51 (t, *J* = 7.6 Hz, 1H), 5.13 (dd, *J* = 13.3, 5.0 Hz, 1H), 4.45 (d, *J* = 17.7 Hz, 1H), 4.29 (d, *J* = 17.7 Hz, 1H), 2.99–2.83 (m, 3H), 2.58 (t, *J* = 7.0 Hz, 3H), 2.45–2.34 (m, 1H), 2.05–1.96 (m, 1H), 1.90–1.76 (m, 2H).

*3-(1-Oxo-4-(7-(piperazin-1-yl)hept-1-yn-1-yl)isoindolin-2-yl)piperidine-2,6-dione* (**14d**). ^1^H NMR (400 MHz, DMSO-*d_6_*) δ 10.96 (s, 1H), 9.29 (s, 2H), 7.69 (dd, *J* = 7.5, 1.1 Hz, 1H), 7.60 (dd, *J* = 7.6, 1.1 Hz, 1H), 7.49 (t, *J* = 7.6 Hz, 1H), 5.12 (dd, *J* = 13.3, 5.1 Hz, 1H), 4.42 (d, *J* = 17.7 Hz, 1H), 4.28 (d, *J* = 17.7 Hz, 1H), 3.52–3.27 (m, 8H), 3.12–3.01 (m, 2H), 2.96–2.78 (m, 1H), 2.62–2.49 (m, 1H), 2.45–2.24 (m, 3H), 2.05–1.94 (m, 1H), 1.70–1.53 (m, 4H), 1.50–1.39 (m, 2H).

#### 3.3.9. Synthesis and Characterization of Intermediate **15**

Using Method VI, 4-bromolenalidomide **11** was coupled with the alkyne linker **12b**. The resulting intermediate underwent palladium-catalyzed hydrogenation (Method IX) and ester hydrolysis (Method II-B) to yield the lenalidomide derivative **15**.

*6-(2-(2,6-Dioxopiperidin-3-yl)-1-oxoisoindolin-4-yl)hexanoic acid* (**15**). ^1^H NMR (400 MHz, DMSO-*d_6_*) δ 11.95 (s, 1H), 10.96 (s, 1H), 7.58–7.50 (m, 1H), 7.48–7.39 (m, 2H), 5.11 (dd, *J* = 13.3, 5.1 Hz, 1H), 4.44 (d, *J* = 17.2 Hz, 1H), 4.29 (d, *J* = 17.1 Hz, 1H), 2.97–2.83 (m, 1H), 2.67–2.54 (m, 3H), 2.45–2.34 (m, 1H), 2.18 (t, *J* = 7.3 Hz, 2H), 2.05–1.94 (m, 1H), 1.65–1.47 (m, 4H), 1.37–1.25 (m, 2H).

#### 3.3.10. Synthesis and Characterization of Intermediates **29**, **30**, **31a** and **31b**

Aniline derivative **8h** was coupled with benzoic acid **28** (Method I-B) and Boc-deprotected (Method V-B) to give intermediate **29**. Subsequent coupling of various linkers to **29** (Method I-B) followed by either ester hydrolysis (Method II-A) or Boc deprotection (Method V-B) furnished the intermediates **30**, **31a**, and **31b**.

*(4-((2-(4-Ethoxyphenoxy)phenyl)carbamoyl)phenyl)methanaminium chloride* (**29**). ^1^H NMR (400 MHz, DMSO-*d*_6_) δ 9.82 (s, 1H), 8.61 (s, 3H), 7.88 (d, *J* = 8.3 Hz, 2H), 7.70 (dd, *J* = 7.7, 1.9 Hz, 1H), 7.58 (d, *J* = 8.3 Hz, 2H), 7.20–7.06 (m, 2H), 6.98–6.79 (m, 5H), 4.09–4.00 (m, 2H), 3.94 (q, *J* = 7.0 Hz, 2H), 1.27 (t, *J* = 7.0 Hz, 3H).

*(E)-4-((4-((2-(4-Ethoxyphenoxy)phenyl)carbamoyl)benzyl)amino)-4-oxobut-2-enoic acid* (**30**). ^1^H NMR (400 MHz, DMSO-*d*_6_) δ 12.86 (s, 1H), 9.68 (s, 1H), 9.02 (t, *J* = 5.9 Hz, 1H), 7.80 (d, *J* = 8.3 Hz, 2H), 7.73 (dd, *J* = 7.7, 1.8 Hz, 1H), 7.34 (d, *J* = 8.3 Hz, 2H), 7.18–7.05 (m, 2H), 7.01–6.78 (m, 6H), 6.54 (d, *J* = 15.5 Hz, 1H), 4.42 (d, *J* = 5.9 Hz, 2H), 3.94 (q, *J* = 7.0 Hz, 2H), 1.27 (t, *J* = 7.0 Hz, 3H).

*4-((4-((2-(4-Ethoxyphenoxy)phenyl)carbamoyl)benzyl)amino)-4-oxobutan-1-aminium chloride* (**31a**). ^1^H NMR (400 MHz, DMSO-*d*_6_) δ 9.70 (s, 1H), 8.58 (t, *J* = 5.9 Hz, 1H), 8.02 (br, 3H), 7.81 (d, *J* = 8.2 Hz, 2H), 7.72 (dd, *J* = 7.7, 1.8 Hz, 1H), 7.32 (d, *J* = 8.2 Hz, 2H), 7.17–7.05 (m, 2H), 6.99–6.84 (m, 4H), 6.81 (dd, *J* = 7.9, 1.5 Hz, 1H), 4.29 (d, *J* = 5.9 Hz, 2H), 3.94 (q, *J* = 6.9 Hz, 2H), 2.69 (d, *J* = 43.1 Hz, 2H), 2.26 (t, *J* = 7.2 Hz, 2H), 1.88–1.70 (m, 2H), 1.27 (t, *J* = 7.0 Hz, 3H).

*6-((4-((2-(4-Ethoxyphenoxy)phenyl)carbamoyl)benzyl)amino)-6-oxohexan-1-aminium chloride* (**31b**). ^1^H NMR (400 MHz, DMSO-*d*_6_) δ 9.69 (s, 1H), 8.43 (t, *J* = 5.9 Hz, 1H), 7.95 (br, 3H), 7.80 (d, *J* = 8.2 Hz, 2H), 7.72 (dd, *J* = 7.7, 1.7 Hz, 1H), 7.30 (d, *J* = 8.2 Hz, 2H), 7.16–7.05 (m, 2H), 6.97–6.84 (m, 4H), 6.81 (dd, *J* = 7.9, 1.5 Hz, 1H), 4.28 (d, *J* = 5.9 Hz, 2H), 3.94 (q, *J* = 6.9 Hz, 2H), 2.78–2.63 (m, 2H), 2.14 (t, *J* = 7.4 Hz, 2H), 1.60–1.43 (m, 4H), 1.36–1.19 (m, 5H).

#### 3.3.11. Synthesis and Characterization of the Final PROTACs

The linker-coupled MYC inhibitors (**9a**–**g**, **10a**–**n**, **30**, **31a** and **31b**) were reacted with the appropriate E3 ligase ligand (**14a**–**d**, **15**, **20a**, **20b**, **26** and **33**) following Method I-B.

*4-(Acrylamidomethyl)-N-(2-(4-((5-(2-(2,6-dioxopiperidin-3-yl)-1-oxoisoindolin-4-yl) pent-4-ynamido)methyl)phenoxy)phenyl)benzamide* (**16a**). ^1^H NMR (400 MHz, DMSO-*d*_6_) δ 10.99 (s, 1H), 9.68 (s, 1H), 8.63 (t, *J* = 6.0 Hz, 1H), 8.44 (t, *J* = 5.9 Hz, 1H), 7.80–7.70 (m, 3H), 7.66 (dd, *J* = 7.5, 0.8 Hz, 1H), 7.54 (dd, *J* = 7.6, 0.9 Hz, 1H), 7.45 (t, *J* = 7.6 Hz, 1H), 7.32 (d, *J* = 8.3 Hz, 2H), 7.23–7.13 (m, 4H), 6.90–6.85 (m, 1H), 6.82 (d, *J* = 8.6 Hz, 2H), 6.27 (dd, *J* = 17.1, 10.1 Hz, 1H), 6.11 (dd, *J* = 17.1, 2.2 Hz, 1H), 5.61 (dd, *J* = 10.1, 2.2 Hz, 1H), 5.13 (dd, *J* = 13.3, 5.1 Hz, 1H), 4.42–4.31 (m, 3H), 4.29–4.19 (m, 3H), 2.95–2.84 (m, 1H), 2.70 (t, *J* = 7.1 Hz, 2H), 2.61–2.52 (m, 1H), 2.47–2.34 (m, 3H), 2.01–1.88 (m, 1H). ^13^C NMR (101 MHz, DMSO-*d*_6_) δ 173.27, 171.39, 170.71, 168.08, 165.56, 165.14, 155.93, 149.93, 144.31, 143.54, 134.78, 134.31, 133.36, 132.34, 131.96, 129.91, 129.03, 128.97, 128.10, 127.58, 126.86, 126.76, 126.02, 124.11, 123.14, 119.56, 119.04, 118.30, 95.98, 76.96, 51.99, 47.29, 42.33, 41.88, 34.86, 31.62, 22.86, 15.99. HRMS (ESI, positive): calcd. for C_42_H_38_N_5_O_7_ [M + H]^+^: *m*/*z* = 724.2766; found: 724.2765. HPLC *t*_R_ = 11.90 min (purity 98.8%).

*4-(Acrylamidomethyl)-N-(2-(4-((6-(2-(2,6-dioxopiperidin-3-yl)-1-oxoisoindolin-4-yl)hex-5-ynamido)methyl)phenoxy)phenyl)benzamide* (**16b**). ^1^H NMR (400 MHz, DMSO-*d*_6_) δ 10.96 (s, 1H), 9.70 (s, 1H), 8.63 (t, *J* = 6.0 Hz, 1H), 8.31 (t, *J* = 5.9 Hz, 1H), 7.78–7.73 (m, 3H), 7.69 (dd, *J* = 7.6, 0.8 Hz, 1H), 7.62 (dd, *J* = 7.6, 0.9 Hz, 1H), 7.49 (t, *J* = 7.6 Hz, 1H), 7.32 (d, *J* = 8.3 Hz, 2H), 7.21–7.16 (m, 4H), 6.91 (d, *J* = 8.5 Hz, 3H), 6.26 (dd, *J* = 17.1, 10.1 Hz, 1H), 6.11 (dd, *J* = 17.1, 2.2 Hz, 1H), 5.61 (dd, *J* = 10.1, 2.2 Hz, 1H), 5.11 (dd, *J* = 13.3, 5.1 Hz, 1H), 4.44 (d, *J* = 17.7 Hz, 1H), 4.38 (d, *J* = 6.0 Hz, 2H), 4.30 (d, *J* = 17.8 Hz, 1H), 4.20 (d, *J* = 5.9 Hz, 2H), 2.93–2.84 (m, 1H), 2.58–2.50 (m, 3H), 2.45–2.37 (m, 1H), 2.29 (t, *J* = 7.3 Hz, 2H), 2.03–1.92 (m, 1H), 1.84–1.77 (m, 2H). ^13^C NMR (101 MHz, DMSO-*d*_6_) δ 173.26, 171.82, 171.37, 168.09, 165.57, 165.13, 155.92, 150.03, 144.23, 143.54, 135.00, 134.55, 133.36, 132.41, 131.96, 129.91, 129.13, 128.98, 128.10, 127.57, 126.87, 126.75, 126.01, 124.08, 123.07, 119.53, 119.21, 118.43, 96.20, 77.14, 52.09, 47.45, 42.32, 41.87, 34.61, 31.64, 24.75, 22.78, 18.92. HRMS (ESI, positive): calcd. for C_43_H_40_N_5_O_7_ [M + H]^+^: *m*/*z* = 738.2922; found: 738.2919. HPLC *t*_R_ = 12.55 min (purity 99.7%).

*4-(Acrylamidomethyl)-N-(2-(4-(2-(6-(2-(2,6-dioxopiperidin-3-yl)-1-oxoisoindolin-4-yl) hex-5-ynamido)ethoxy)phenoxy)phenyl)benzamide* (**16c**). ^1^H NMR (400 MHz, DMSO-*d*_6_) δ 10.96 (s, 1H), 9.68 (s, 1H), 8.64 (t, *J* = 5.6 Hz, 1H), 8.08 (t, *J* = 5.0 Hz, 1H), 7.81 (d, *J* = 7.9 Hz, 2H), 7.74 (d, *J* = 6.8 Hz, 1H), 7.68 (d, *J* = 7.5 Hz, 1H), 7.62 (d, *J* = 7.5 Hz, 1H), 7.49 (t, *J* = 7.6 Hz, 1H), 7.34 (d, *J* = 7.8 Hz, 2H), 7.21–7.08 (m, 2H), 6.93 (q, *J* = 9.1 Hz, 4H), 6.82 (d, *J* = 7.5 Hz, 1H), 6.27 (dd, *J* = 17.0, 10.1 Hz, 1H), 6.11 (dd, *J* = 17.1, 1.2 Hz, 1H), 5.61 (dd, *J* = 10.1, 1.2 Hz, 1H), 5.11 (dd, *J* = 13.2, 4.7 Hz, 1H), 4.48–4.36 (m, 3H), 4.30 (d, *J* = 17.7 Hz, 1H), 3.92 (t, *J* = 5.3 Hz, 2H), 3.44–3.34 (m, 2H), 2.97–2.80 (m, 1H), 2.61–2.51 (m, 1H), 2.46–2.35 (m, 3H), 2.26 (t, *J* = 7.2 Hz, 2H), 2.04–1.94 (m, 1H), 1.84–1.73 (m, 2H). ^13^C NMR (101 MHz, DMSO-*d*_6_) δ 173.26, 172.23, 171.38, 168.09, 165.53, 165.13, 155.03, 150.97, 150.33, 144.22, 143.56, 134.55, 133.41, 132.41, 131.97, 129.26, 128.97, 128.11, 127.59, 126.59, 126.01, 123.37, 123.06, 120.47, 119.21, 118.28, 116.08, 96.24, 77.09, 67.25, 52.10, 47.45, 42.32, 38.66, 34.58, 31.64, 24.74, 22.79, 18.90. HRMS (ESI, positive): calcd. for C_44_H_42_N_5_O_8_ [M + H]^+^: *m*/*z* = 768.3028; found: 768.3032. HPLC *t*_R_ = 12.72 min (purity 99.8%).

*4-(Acrylamidomethyl)-N-(2-(4-(3-(6-(2-(2,6-dioxopiperidin-3-yl)-1-oxoisoindolin-4-yl) hex-5-ynamido)propoxy)phenoxy)phenyl)benzamide* (**16d**). ^1^H NMR (400 MHz, DMSO-*d*_6_) δ 10.97 (s, 1H), 9.68 (s, 1H), 8.64 (t, *J* = 6.0 Hz, 1H), 7.91 (t, *J* = 5.5 Hz, 1H), 7.82 (d, *J* = 8.3 Hz, 2H), 7.74 (dd, *J* = 7.6, 1.9 Hz, 1H), 7.68 (dd, *J* = 7.5, 0.6 Hz, 1H), 7.62 (dd, *J* = 7.6, 0.7 Hz, 1H), 7.49 (t, *J* = 7.6 Hz, 1H), 7.34 (d, *J* = 8.3 Hz, 2H), 7.18–7.06 (m, 2H), 6.92 (dd, *J* = 23.3, 9.2 Hz, 4H), 6.82 (dd, *J* = 7.8, 1.7 Hz, 1H), 6.27 (dd, *J* = 17.1, 10.1 Hz, 1H), 6.11 (dd, *J* = 17.1, 2.2 Hz, 1H), 5.61 (dd, *J* = 10.1, 2.2 Hz, 1H), 5.12 (dd, *J* = 13.3, 5.1 Hz, 1H), 4.45 (d, *J* = 17.7 Hz, 1H), 4.39 (d, *J* = 6.0 Hz, 2H), 4.30 (d, *J* = 17.7 Hz, 1H), 3.91 (t, *J* = 6.2 Hz, 2H), 3.22–3.13 (m, 2H), 2.95–2.83 (m, 1H), 2.61–2.52 (m, 1H), 2.48–2.36 (m, 3H), 2.24 (t, *J* = 7.3 Hz, 2H), 2.06–1.94 (m, 1H), 1.85–1.70 (m, 4H). ^13^C NMR (101 MHz, DMSO-*d*_6_) δ 173.26, 171.85, 171.38, 168.09, 165.52, 165.14, 155.20, 151.01, 150.15, 144.22, 143.56, 134.54, 133.42, 132.41, 131.97, 129.23, 128.98, 128.11, 127.59, 126.57, 126.00, 123.32, 123.07, 120.48, 119.22, 118.21, 115.97, 96.23, 77.10, 66.11, 52.10, 47.44, 42.32, 35.94, 34.67, 31.64, 29.39, 24.76, 22.79, 18.91. HRMS (ESI, positive): calcd. for C_45_H_44_N_5_O_8_ [M + H]^+^: *m*/*z* = 782.3184; found: 782.3184. HPLC *t*_R_ = 12.97 min (purity 97.6%).

*4-(Acrylamidomethyl)-N-(2-(4-(3-(6-(2-(2,6-dioxopiperidin-3-yl)-1-oxoisoindolin-4-yl) hex-5-ynamido)propanamido)phenoxy)phenyl)benzamide* (**16e**). ^1^H NMR (400 MHz, DMSO-*d*_6_) δ 10.96 (s, 1H), 9.88 (s, 1H), 9.69 (s, 1H), 8.63 (t, *J* = 5.9 Hz, 1H), 7.95 (t, *J* = 5.5 Hz, 1H), 7.80 (d, *J* = 8.2 Hz, 2H), 7.76–7.70 (m, 1H), 7.68 (d, *J* = 7.7 Hz, 1H), 7.61 (d, *J* = 7.6 Hz, 1H), 7.58–7.45 (m, 3H), 7.33 (d, *J* = 8.2 Hz, 2H), 7.21–7.09 (m, 2H), 6.97–6.83 (m, 3H), 6.27 (dd, *J* = 17.1, 10.1 Hz, 1H), 6.11 (dd, *J* = 17.1, 2.2 Hz, 1H), 5.61 (dd, *J* = 10.1, 2.2 Hz, 1H), 5.11 (dd, *J* = 13.3, 5.1 Hz, 1H), 4.48–4.36 (m, 3H), 4.30 (d, *J* = 17.7 Hz, 1H), 3.35–3.30 (m, 2H), 2.95–2.83 (m, 1H), 2.64–2.49 (m, 3H), 2.47–2.38 (m, 3H), 2.22 (t, *J* = 7.3 Hz, 2H), 2.04–1.93 (m, 1H), 1.83–1.70 (m, 2H). ^13^C NMR (101 MHz, DMSO-*d*_6_) δ 173.27, 171.98, 171.39, 169.63, 168.09, 165.53, 165.13, 152.23, 150.44, 144.21, 143.55, 135.35, 134.55, 133.39, 132.40, 131.97, 130.03, 129.55, 129.01, 128.96, 128.11, 127.59, 126.75, 126.68, 126.00, 123.72, 123.05, 121.04, 119.21, 119.14, 118.88, 96.25, 77.05, 52.11, 47.45, 42.32, 36.79, 35.56, 34.62, 31.64, 24.84, 22.79, 18.87. HRMS (ESI, positive): calcd. for C_45_H_42_N_6_NaO_8_ [M + Na]^+^: *m*/*z* = 817.2962; found: 817.2969. HPLC *t*_R_ = 12.22 min (purity 97.1%).

*4-(Acrylamidomethyl)-N-(2-(4-(4-(6-(2-(2,6-dioxopiperidin-3-yl)-1-oxoisoindolin-4-yl) hex-5-ynamido)butanamido)phenoxy)phenyl)benzamide* (**16f**). ^1^H NMR (400 MHz, DMSO-*d*_6_) δ 10.96 (s, 1H), 9.84 (s, 1H), 9.69 (s, 1H), 8.63 (s, 1H), 7.91–7.44 (m, 9H), 7.32 (d, *J* = 7.9 Hz, 2H), 7.21–7.08 (m, 2H), 7.01–6.79 (m, 3H), 6.26 (dd, *J* = 16.9, 10.0 Hz, 1H), 6.11 (d, *J* = 16.5 Hz, 1H), 5.61 (d, *J* = 10.2 Hz, 1H), 5.11 (dd, *J* = 12.9, 5.9 Hz, 1H), 4.54–4.17 (m, 4H), 3.06 (dd, *J* = 11.2, 6.5 Hz, 2H), 2.92–2.82 (m, 1H), 2.68–2.52 (m, 3H), 2.45–2.38 (m, 2H), 2.30–2.17 (m, 3H), 2.03–1.93 (m, 1H), 1.84–1.60 (m, 4H). ^13^C NMR (101 MHz, DMSO-*d*_6_) δ 173.26, 171.80, 171.38, 171.02, 168.08, 165.53, 165.12, 152.15, 150.46, 144.23, 143.54, 135.46, 134.55, 133.40, 133.12, 132.41, 131.97, 130.03, 129.53, 129.00, 128.10, 127.58, 126.72, 125.99, 123.69, 123.06, 120.96, 119.19, 118.85, 96.25, 77.09, 52.10, 47.45, 42.32, 38.56, 34.72, 34.20, 31.63, 25.69, 24.80, 22.78, 18.94. HRMS (ESI, positive): calcd. for C_46_H_45_N_6_O_8_ [M + H]^+^: *m*/*z* = 809.3293; found: 809.3300. HPLC *t*_R_ = 9.33 min (purity 95%).

*4-(Acrylamidomethyl)-N-(2-(4-((6-(2-(2,6-dioxopiperidin-3-yl)-1-oxoisoindolin-4-yl)hexanamido)methyl)phenoxy)phenyl)benzamide* (**16g**). ^1^H NMR (400 MHz, DMSO-*d*_6_) δ 10.96 (s, 1H), 9.70 (s, 1H), 8.63 (t, *J* = 5.7 Hz, 1H), 8.21 (t, *J* = 5.6 Hz, 1H), 7.75 (dd, *J* = 12.9, 5.5 Hz, 3H), 7.59–7.50 (m, 1H), 7.42 (d, *J* = 4.2 Hz, 2H), 7.32 (d, *J* = 8.1 Hz, 2H), 7.21–7.13 (m, 4H), 6.91 (d, *J* = 8.6 Hz, 3H), 6.27 (dd, *J* = 17.1, 10.1 Hz, 1H), 6.11 (dd, *J* = 17.1, 2.0 Hz, 1H), 5.61 (dd, *J* = 10.1, 2.0 Hz, 1H), 5.11 (dd, *J* = 13.1, 5.3 Hz, 1H), 4.44 (d, *J* = 17.1 Hz, 1H), 4.38 (d, *J* = 5.8 Hz, 2H), 4.28 (d, *J* = 17.2 Hz, 1H), 4.18 (d, *J* = 5.5 Hz, 2H), 2.97–2.81 (m, 1H), 2.65–2.50 (m, 3H), 2.45–2.34 (m, 1H), 2.23–2.05 (m, 2H), 2.03–1.91 (m, 1H), 1.75–1.51 (m, 4H), 1.33–1.20 (m, 2H). ^13^C NMR (101 MHz, DMSO-*d*_6_) δ 173.30, 172.41, 171.47, 168.80, 165.57, 165.13, 155.89, 150.04, 143.53, 140.93, 137.90, 135.13, 133.37, 131.97, 129.91, 129.11, 128.67, 128.10, 127.57, 126.87, 126.75, 126.00, 124.08, 121.02, 119.51, 118.43, 52.01, 46.68, 42.32, 41.81, 35.69, 31.64, 31.57, 29.43, 28.99, 25.48, 22.94. HRMS (ESI, positive): calcd. for C_43_H_44_N_5_O_7_ [M + H]^+^: *m*/*z* = 742.3235; found: 742.3229. HPLC *t*_R_ = 12.45 min (purity 99.3%).

*4-(Acrylamidomethyl)-N-(2-(4-((6-(4-(2-(2,6-dioxopiperidin-3-yl)-1,3-dioxoisoindolin-5-yl)piperazin-1-yl)-6-oxohexyl)oxy)phenoxy)phenyl)benzamide* (**21a**). ^1^H NMR (400 MHz, DMSO-*d*_6_) δ 11.06 (s, 1H), 9.67 (s, 1H), 8.64 (t, *J* = 5.9 Hz, 1H), 7.81 (d, *J* = 8.2 Hz, 2H), 7.77–7.65 (m, 2H), 7.31 (dd, *J* = 20.5, 5.2 Hz, 3H), 7.24–7.07 (m, 3H), 6.98–6.79 (m, 5H), 6.27 (dd, *J* = 17.1, 10.1 Hz, 1H), 6.11 (dd, *J* = 17.1, 2.2 Hz, 1H), 5.61 (dd, *J* = 10.1, 2.2 Hz, 1H), 5.06 (dd, *J* = 12.9, 5.4 Hz, 1H), 4.39 (d, *J* = 6.0 Hz, 2H), 3.90 (t, *J* = 6.4 Hz, 2H), 3.67–3.38 (m, 8H), 2.93–2.80 (m, 1H), 2.62–2.50 (m, 2H), 2.36 (t, *J* = 7.2 Hz, 2H), 2.05–1.95 (m, 1H), 1.76–1.64 (m, 2H), 1.63–1.49 (m, 2H), 1.48–1.35 (m, 2H). ^13^C NMR (101 MHz, DMSO-*d*_6_) δ 173.23, 171.25, 170.50, 167.95, 167.40, 165.52, 165.13, 155.32, 155.30, 150.97, 150.08, 143.56, 134.29, 133.43, 131.98, 129.24, 128.11, 127.59, 126.56, 126.52, 126.00, 125.35, 123.32, 120.44, 118.91, 118.26, 118.18, 115.94, 108.35, 68.26, 49.25, 47.25, 46.99, 44.52, 42.32, 32.60, 31.44, 29.03, 25.79, 24.88, 22.63. HRMS (ESI, positive): calcd. for C_46_H_47_N_6_O_9_ [M + H]^+^: *m*/*z* = 827.3399; found: 827.3403. HPLC *t*_R_ = 13.44 min (purity 96.1%).

*4-(Acrylamidomethyl)-N-(2-(4-((7-(4-(2-(2,6-dioxopiperidin-3-yl)-1,3-dioxoisoindolin-5-yl)piperazin-1-yl)-7-oxoheptyl)oxy)phenoxy)phenyl)benzamide* (**21b**). ^1^H NMR (400 MHz, DMSO-*d*_6_) δ 11.05 (s, 1H), 9.67 (s, 1H), 8.64 (t, *J* = 5.9 Hz, 1H), 7.81 (d, *J* = 8.2 Hz, 2H), 7.74 (dd, *J* = 7.6, 1.8 Hz, 1H), 7.68 (d, *J* = 8.5 Hz, 1H), 7.33 (d, *J* = 8.5 Hz, 3H), 7.21 (dd, *J* = 8.6, 2.1 Hz, 1H), 7.17–7.07 (m, 2H), 6.92 (dd, *J* = 19.7, 9.2 Hz, 4H), 6.83 (dd, *J* = 7.8, 1.6 Hz, 1H), 6.27 (dd, *J* = 17.1, 10.1 Hz, 1H), 6.11 (dd, *J* = 17.1, 2.2 Hz, 1H), 5.61 (dd, *J* = 10.1, 2.2 Hz, 1H), 5.06 (dd, *J* = 12.9, 5.4 Hz, 1H), 4.39 (d, *J* = 6.0 Hz, 2H), 3.89 (t, *J* = 6.4 Hz, 2H), 3.59 (s, 4H), 3.46 (dd, *J* = 22.1, 0.6 Hz, 4H), 2.93–2.81 (m, 1H), 2.62–2.51 (m, 2H), 2.34 (t, *J* = 7.4 Hz, 2H), 2.05–1.95 (m, 1H), 1.71–1.62 (m, 2H), 1.56–1.47 (m, 2H), 1.44–1.29 (m, 4H). ^13^C NMR (101 MHz, DMSO-*d*_6_) δ 173.20, 171.30, 170.48, 167.95, 167.40, 165.52, 165.13, 155.32, 155.30, 150.97, 150.07, 143.55, 134.29, 133.43, 131.97, 129.24, 128.11, 127.58, 126.57, 126.53, 125.99, 125.35, 123.32, 120.44, 118.91, 118.26, 118.18, 115.94, 108.34, 68.25, 49.24, 47.26, 46.99, 44.53, 42.32, 32.59, 31.43, 29.07, 28.96, 25.82, 25.07, 22.63. HRMS (ESI, positive): calcd. for C_47_H_49_N_6_O_9_ [M + H]^+^: *m/z* = 841.3556; found: 841.3556. HPLC *t*_R_ = 13.85 min (purity 98.3%).

*4-(Acrylamidomethyl)-N-(2-(4-((6-((2-(2,6-dioxopiperidin-3-yl)-1,3-dioxoisoindolin-4- yl)amino)hexanamido)methyl)phenoxy) phenyl)benzamide* (**21c**). ^1^H NMR (400 MHz, DMSO-*d*_6_) δ 11.06 (s, 1H), 9.70 (s, 1H), 8.63 (t, *J* = 5.8 Hz, 1H), 8.23 (t, *J* = 5.8 Hz, 1H), 7.78–7.73 (m, 3H), 7.58–7.49 (m, 1H), 7.32 (d, *J* = 8.1 Hz, 2H), 7.20–7.15 (m, 4H), 7.05 (d, *J* = 8.6 Hz, 1H), 6.99 (d, *J* = 7.0 Hz, 1H), 6.92 (d, *J* = 8.4 Hz, 3H), 6.50 (t, *J* = 5.7 Hz, 1H), 6.27 (dd, *J* = 17.1, 10.1 Hz, 1H), 6.11 (dd, *J* = 17.1, 2.1 Hz, 1H), 5.61 (dd, *J* = 10.1, 2.1 Hz, 1H), 5.03 (dd, *J* = 12.8, 5.2 Hz, 1H), 4.38 (d, *J* = 5.9 Hz, 2H), 4.18 (d, *J* = 5.8 Hz, 2H), 3.28–3.22 (m, 2H), 2.91–2.81 (m, 1H), 2.59–2.52 (m, 2H), 2.11 (t, *J* = 7.3 Hz, 2H), 2.05–1.97 (m, 1H), 1.60–1.48 (m, 4H), 1.37–1.24 (m, 2H). ^13^C NMR (101 MHz, DMSO-*d*_6_) δ 173.22, 172.38, 170.51, 169.38, 167.73, 165.56, 165.12, 155.89, 150.03, 146.90, 143.53, 136.70, 135.13, 133.37, 132.63, 131.97, 129.90, 129.12, 128.10, 127.58, 126.84, 126.73, 126.00, 124.06, 119.49, 118.44, 117.61, 110.82, 109.46, 48.99, 42.32, 42.20, 41.82, 35.70, 31.43, 28.92, 26.43, 25.46, 22.60. HRMS (ESI, positive): calcd. for C_43_H_43_N_6_O_8_ [M + H]^+^: *m*/*z* = 771.3137; found: 771.3136. HPLC *t*_R_ = 12.92 min (purity 99.2%).

*6-(4-(6-(4-(2-(4-(Acrylamidomethyl)benzamido)phenoxy)phenoxy)hexanoyl)piperazin-1-yl)-N-(2,6-dioxopiperidin-3-yl)picolinamide* (**27a**). ^1^H NMR (400 MHz, DMSO-*d*_6_) δ 10.86 (s, 1H), 9.67 (s, 1H), 8.76 (d, *J* = 8.4 Hz, 1H), 8.64 (t, *J* = 5.9 Hz, 1H), 7.81 (d, *J* = 8.3 Hz, 2H), 7.77–7.66 (m, 2H), 7.33 (dd, *J* = 7.6, 5.4 Hz, 3H), 7.18–7.07 (m, 2H), 7.03 (d, *J* = 8.6 Hz, 1H), 6.97–6.86 (m, 4H), 6.83 (dd, *J* = 7.8, 1.7 Hz, 1H), 6.27 (dd, *J* = 17.1, 10.1 Hz, 1H), 6.11 (dd, *J* = 17.1, 2.2 Hz, 1H), 5.61 (dd, *J* = 10.1, 2.2 Hz, 1H), 4.79–4.67 (m, 1H), 4.39 (d, *J* = 6.0 Hz, 2H), 3.90 (t, *J* = 6.4 Hz, 2H), 3.70–3.48 (m, 8H), 2.83–2.72 (m, 1H), 2.52 (dd, *J* = 5.0, 1.4 Hz, 1H), 2.37 (t, *J* = 7.3 Hz, 2H), 2.25–2.15 (m, 1H), 2.02–1.93 (m, 1H), 1.75–1.64 (m, 2H), 1.61–1.50 (m, 2H), 1.47–1.37 (m, 2H). ^13^C NMR (101 MHz, DMSO-*d*_6_) δ 173.44, 172.68, 171.20, 165.52, 165.13, 164.58, 158.04, 155.32, 151.00, 150.08, 147.90, 143.55, 139.30, 133.43, 131.97, 129.23, 128.11, 127.58, 126.58, 126.56, 126.00, 123.31, 120.45, 118.25, 115.94, 111.80, 110.81, 68.26, 49.88, 45.15, 44.97, 44.77, 42.32, 32.65, 31.46, 29.04, 25.81, 24.97, 24.49. HRMS (ESI, positive): calcd. for C_44_H_48_N_7_O_8_ [M + H]^+^: *m*/*z* = 802.3559; found: 802.3561. HPLC *t*_R_ = 13.40 min (purity 97.8%).

*6-(4-(7-(4-(2-(4-(Acrylamidomethyl)benzamido)phenoxy)phenoxy)heptanoyl)piperazin-1-yl)-N-(2,6-dioxopiperidin-3-yl)picolinamide* (**27b**). ^1^H NMR (400 MHz, DMSO-*d*_6_) δ 10.87 (s, 1H), 9.68 (s, 1H), 8.77 (d, *J* = 8.4 Hz, 1H), 8.64 (t, *J* = 5.9 Hz, 1H), 7.81 (d, *J* = 8.2 Hz, 2H), 7.77–7.67 (m, 2H), 7.39–7.28 (m, 3H), 7.18–7.06 (m, 2H), 7.03 (d, *J* = 8.6 Hz, 1H), 6.98–6.77 (m, 5H), 6.27 (dd, *J* = 17.1, 10.1 Hz, 1H), 6.12 (dd, *J* = 17.1, 2.2 Hz, 1H), 5.61 (dd, *J* = 10.1, 2.2 Hz, 1H), 4.78–4.68 (m, 1H), 4.39 (d, *J* = 6.0 Hz, 2H), 3.89 (t, *J* = 6.4 Hz, 2H), 3.71–3.49 (m, 8H), 2.85–2.73 (m, 1H), 2.53 (d, *J* = 3.7 Hz, 1H), 2.35 (t, *J* = 7.4 Hz, 2H), 2.29–2.16 (m, 1H), 2.02–1.93 (m, 1H), 1.72–1.61 (m, 2H), 1.58–1.47 (m, 2H), 1.44–1.30 (m, 4H). ^13^C NMR (101 MHz, DMSO-*d*_6_) δ 173.44, 172.68, 171.26, 165.53, 165.14, 164.59, 158.03, 155.33, 150.99, 150.07, 147.91, 143.56, 139.29, 133.43, 131.97, 129.23, 128.11, 127.59, 126.57, 126.53, 126.00, 123.31, 120.45, 118.24, 115.94, 111.80, 110.80, 68.26, 49.89, 45.15, 44.98, 44.76, 42.33, 41.08, 32.66, 31.46, 29.08, 29.00, 25.84, 25.17, 24.50. HRMS (ESI, positive): calcd. for C_45_H_50_N_7_O_8_ [M + H]^+^: *m*/*z* = 816.3715; found: 816.3722. HPLC *t*_R_ = 13.83 min (purity 98.1%).

*N^1^-(5-(2-(2,6-Dioxopiperidin-3-yl)-1-oxoisoindolin-4-yl)pent-4-yn-1-yl)-N^4^-(4-((2-(4-ethoxyphenoxy)phenyl)carbamoyl)benzyl)fumaramide* (**32a**). ^1^H NMR (400 MHz, DMSO-*d*_6_) δ 10.96 (s, 1H), 9.68 (s, 1H), 8.89 (t, *J* = 5.9 Hz, 1H), 8.47 (t, *J* = 5.6 Hz, 1H), 7.81 (d, *J* = 8.3 Hz, 2H), 7.76–7.66 (m, 2H), 7.63–7.59 (m, 1H), 7.49 (t, *J* = 7.6 Hz, 1H), 7.34 (d, *J* = 8.3 Hz, 2H), 7.18–7.07 (m, 2H), 6.98–6.78 (m, 7H), 5.12 (dd, *J* = 13.3, 5.1 Hz, 1H), 4.46 (d, *J* = 17.9 Hz, 1H), 4.41 (d, *J* = 5.9 Hz, 2H), 4.31 (d, *J* = 17.8 Hz, 1H), 3.94 (q, *J* = 7.0 Hz, 2H), 3.27 (d, *J* = 7.7 Hz, 2H), 2.96–2.83 (m, 1H), 2.62–2.49 (m, 3H), 2.46–2.40 (m, 1H), 2.04–1.93 (m, 1H), 1.78–1.67 (m, 2H), 1.26 (t, *J* = 7.0 Hz, 3H). ^13^C NMR (101 MHz, DMSO-*d*_6_) δ 173.33, 171.40, 168.09, 165.50, 164.31, 164.13, 155.14, 151.00, 150.05, 144.34, 143.19, 134.40, 133.52, 133.46, 132.67, 132.40, 129.20, 129.00, 128.13, 127.51, 126.58, 123.31, 123.09, 120.43, 119.14, 118.24, 115.89, 96.04, 77.12, 63.82, 52.06, 47.44, 42.49, 38.35, 31.63, 28.35, 22.79, 16.89, 15.09. HRMS (ESI, positive): calcd. for C_44_H_42_N_5_O_8_ [M + H]^+^: *m*/*z* = 768.3028; found: 768.3027. HPLC *t*_R_ = 14.55 min (purity 98.7%).

*(E)-4-((4-(4-(7-(2-(2,6-Dioxopiperidin-3-yl)-1-oxoisoindolin-4-yl)hept-6-yn-1-yl)pipera-zin-1-yl)-4-oxobut-2-enamido)methyl)-N-(2-(4-ethoxyphenoxy)phenyl)benzamide* (**32b**). ^1^H NMR (400 MHz, DMSO-*d*_6_) δ 10.96 (s, 1H), 9.67 (s, 1H), 8.94 (t, *J* = 5.8 Hz, 1H), 7.80 (d, *J* = 8.2 Hz, 2H), 7.73 (dd, *J* = 7.6, 1.6 Hz, 1H), 7.67 (d, *J* = 7.4 Hz, 1H), 7.60 (d, *J* = 7.4 Hz, 1H), 7.48 (t, *J* = 7.6 Hz, 1H), 7.34 (d, *J* = 8.2 Hz, 2H), 7.26 (d, *J* = 15.1 Hz, 1H), 7.17–7.06 (m, 2H), 6.99–6.77 (m, 6H), 5.12 (dd, *J* = 13.3, 5.0 Hz, 1H), 4.50–4.35 (m, 3H), 4.28 (d, *J* = 17.6 Hz, 1H), 3.94 (q, *J* = 6.9 Hz, 2H), 3.50 (s, 4H), 2.95–2.81 (m, 1H), 2.61–2.52 (m, 1H), 2.46–2.21 (m, 9H), 2.03–1.94 (m, 1H), 1.62–1.51 (m, 2H), 1.51–1.35 (m, 4H), 1.26 (t, *J* = 6.9 Hz, 3H). ^13^C NMR (101 MHz, DMSO-*d*_6_) δ 173.24, 171.39, 168.08, 165.48, 164.28, 163.57, 155.13, 150.98, 150.05, 144.14, 143.16, 134.67, 134.46, 133.49, 132.39, 129.80, 129.21, 129.01, 128.13, 127.58, 126.57, 126.54, 123.30, 123.01, 120.43, 119.28, 118.23, 115.88, 96.77, 76.86, 63.81, 57.92, 53.59, 52.79, 52.06, 47.37, 45.82, 42.51, 42.04, 31.63, 28.39, 26.62, 26.09, 22.84, 19.17, 15.09. HRMS (ESI, positive): calcd. for C_50_H_53_N_6_O_8_ [M + H]^+^: *m*/*z* = 865.3919; found: 865.3931. HPLC *t*_R_ = 14.13 min (purity 99.6%).

*4-((4-(6-(2-(2,6-Dioxopiperidin-3-yl)-1-oxoisoindolin-4-yl)hex-5-ynamido)butanamido)methyl)-N-(2-(4-ethoxyphenoxy)phenyl)benzamide* (**32c**). ^1^H NMR (400 MHz, DMSO-*d*_6_) δ 10.95 (s, 1H), 9.65 (s, 1H), 8.35 (t, *J* = 5.9 Hz, 1H), 7.85–7.76 (m, 3H), 7.73 (dd, *J* = 7.6, 1.9 Hz, 1H), 7.68 (d, *J* = 7.5 Hz, 1H), 7.65–7.58 (m, 1H), 7.48 (t, *J* = 7.6 Hz, 1H), 7.31 (d, *J* = 8.3 Hz, 2H), 7.17–7.06 (m, 2H), 6.97–6.85 (m, 4H), 6.82 (dd, *J* = 7.9, 1.6 Hz, 1H), 5.10 (dd, *J* = 13.3, 5.1 Hz, 1H), 4.44 (d, *J* = 17.7 Hz, 1H), 4.35–4.22 (m, 3H), 3.94 (q, *J* = 7.0 Hz, 2H), 3.02 (dd, *J* = 12.9, 6.8 Hz, 2H), 2.95–2.81 (m, 1H), 2.61–2.52 (m, 1H), 2.46–2.37 (m, 3H), 2.21 (t, *J* = 7.4 Hz, 2H), 2.13 (t, *J* = 7.5 Hz, 2H), 2.03–1.93 (m, 1H), 1.84–1.71 (m, 2H), 1.69–1.57 (m, 2H), 1.27 (t, *J* = 7.0 Hz, 3H). ^13^C NMR (101 MHz, DMSO-*d*_6_) δ 173.26, 172.33, 171.76, 171.37, 168.09, 165.53, 155.15, 150.98, 150.04, 144.22, 143.95, 134.54, 133.27, 132.40, 129.21, 128.97, 128.04, 127.41, 126.55, 126.50, 123.29, 123.05, 120.46, 119.21, 118.19, 115.89, 96.24, 77.08, 63.82, 52.10, 47.45, 42.19, 38.64, 34.70, 33.28, 31.63, 25.90, 24.79, 22.78, 18.92, 15.09. HRMS (ESI, positive): calcd. for C_45_H_46_N_5_O_8_ [M + H]^+^: *m*/*z* = 784.3341; found: 784.3342. HPLC *t*_R_ = 14.31 min (purity 98.1%).

*4-((6-(6-(2-(2,6-Dioxopiperidin-3-yl)-1-oxoisoindolin-4-yl)hex-5-ynamido)hexanamido)methyl)-N-(2-(4-ethoxyphenoxy)phenyl)benzamide* (**32d**). ^1^H NMR (400 MHz, DMSO-*d*_6_) δ 10.96 (s, 1H), 9.65 (s, 1H), 8.32 (t, *J* = 5.9 Hz, 1H), 7.83–7.72 (m, 4H), 7.70–7.66 (m, 1H), 7.61 (dd, *J* = 7.6, 0.8 Hz, 1H), 7.48 (t, *J* = 7.6 Hz, 1H), 7.30 (d, *J* = 8.3 Hz, 2H), 7.16–7.06 (m, 2H), 6.97–6.85 (m, 4H), 6.82 (dd, *J* = 7.9, 1.7 Hz, 1H), 5.11 (dd, *J* = 13.3, 5.1 Hz, 1H), 4.44 (d, *J* = 17.7 Hz, 1H), 4.35–4.22 (m, 3H), 3.94 (q, *J* = 7.0 Hz, 2H), 3.00 (dd, *J* = 12.8, 6.8 Hz, 2H), 2.94–2.81 (m, 1H), 2.63–2.50 (m, 1H), 2.46–2.35 (m, 3H), 2.24–2.15 (m, 2H), 2.11 (t, *J* = 7.4 Hz, 2H), 2.02–1.93 (m, 1H), 1.82–1.71 (m, 2H), 1.54–1.44 (m, 2H), 1.41–1.31 (m, 2H), 1.27 (t, *J* = 7.0 Hz, 3H), 1.23–1.16 (m, 2H). ^13^C NMR (101 MHz, DMSO-*d*_6_) δ 173.24, 172.59, 171.61, 171.37, 168.09, 165.53, 155.15, 150.96, 150.04, 144.21, 144.04, 134.53, 133.26, 132.40, 129.22, 128.96, 128.03, 127.38, 126.53, 126.49, 123.28, 123.05, 120.46, 119.22, 118.19, 115.88, 96.25, 77.06, 63.81, 52.09, 47.43, 42.15, 38.79, 35.71, 34.71, 31.64, 29.38, 26.58, 25.44, 24.83, 22.79, 18.92, 15.09. HRMS (ESI, positive): calcd. for C_47_H_50_N_5_O_8_ [M + H]^+^: *m*/*z* = 812.3654; found: 812.3653. HPLC *t*_R_ = 14.45 min (purity 98.7%).

*4-((6-(5-(2-(2,6-Dioxopiperidin-3-yl)-1-oxoisoindolin-4-yl)pent-4-ynamido)hexanamido)methyl)-N-(2-(4-ethoxyphenoxy)phenyl)benzamide* (**32e**). ^1^H NMR (400 MHz, DMSO-*d*_6_) δ 10.98 (s, 1H), 9.65 (s, 1H), 8.30 (t, *J* = 5.9 Hz, 1H), 7.89 (t, *J* = 5.5 Hz, 1H), 7.80 (d, *J* = 8.3 Hz, 2H), 7.74 (dd, *J* = 7.7, 1.9 Hz, 1H), 7.68 (dd, *J* = 7.5, 0.8 Hz, 1H), 7.57 (dd, *J* = 7.6, 0.9 Hz, 1H), 7.48 (t, *J* = 7.6 Hz, 1H), 7.30 (d, *J* = 8.3 Hz, 2H), 7.17–7.05 (m, 2H), 6.98–6.85 (m, 4H), 6.82 (dd, *J* = 7.9, 1.6 Hz, 1H), 5.13 (dd, *J* = 13.3, 5.1 Hz, 1H), 4.39 (d, *J* = 17.8 Hz, 1H), 4.34–4.19 (m, 3H), 3.94 (q, *J* = 7.0 Hz, 2H), 3.02 (dd, *J* = 13.0, 6.6 Hz, 2H), 2.96–2.83 (m, 1H), 2.65 (t, *J* = 7.2 Hz, 2H), 2.62–2.53 (m, 1H), 2.44–2.31 (m, 3H), 2.08 (t, *J* = 7.4 Hz, 2H), 2.04–1.95 (m, 1H), 1.52–1.42 (m, 2H), 1.41–1.32 (m, 2H), 1.27 (t, *J* = 7.0 Hz, 3H), 1.23–1.17 (m, 2H). ^13^C NMR (101 MHz, DMSO-*d*_6_) δ 173.24, 172.58, 171.39, 170.42, 168.08, 165.53, 155.15, 150.97, 150.04, 144.27, 144.03, 134.31, 133.26, 132.35, 129.22, 129.00, 128.03, 127.39, 126.54, 126.50, 123.28, 123.11, 120.46, 119.11, 118.19, 115.88, 96.04, 76.82, 63.81, 51.98, 47.27, 42.15, 38.89, 35.68, 34.86, 31.62, 29.40, 26.55, 25.40, 22.89, 15.95, 15.09. HRMS (ESI, positive): calcd. for C_46_H_47_N_5_NaO_8_ [M + Na]^+^: *m/z* = 820.3322; found: 820.3316. HPLC *t*_R_ = 14.23 min (purity 99.2%).

*N^1^-(4-(2-(4-(Acrylamidomethyl)benzamido)phenoxy)phenyl)-N^4^-((S)-1-((2S,4R)-4-hydroxy-2-((4-(4-methylthiazol-5-yl)benzyl)carbamoyl)pyrrolidin-1-yl)-3,3-dimethyl-1-oxobutan-2-yl)succinamide* (**34a**). ^1^H NMR (400 MHz, DMSO-*d*_6_) δ 9.89 (s, 1H), 9.70 (s, 1H), 8.96 (s, 1H), 8.64 (t, *J* = 6.0 Hz, 1H), 8.54 (t, *J* = 6.0 Hz, 1H), 7.92 (d, *J* = 9.3 Hz, 1H), 7.80 (d, *J* = 8.3 Hz, 2H), 7.74 (dd, *J* = 7.5, 2.1 Hz, 1H), 7.53 (d, *J* = 9.0 Hz, 2H), 7.44–7.29 (m, 6H), 7.20–7.10 (m, 2H), 6.93 (d, *J* = 9.1 Hz, 2H), 6.89 (dd, *J* = 7.7, 1.8 Hz, 1H), 6.27 (dd, *J* = 17.1, 10.1 Hz, 1H), 6.11 (dd, *J* = 17.1, 2.2 Hz, 1H), 5.61 (dd, *J* = 10.1, 2.2 Hz, 1H), 5.10 (d, *J* = 3.4 Hz, 1H), 4.53 (d, *J* = 9.4 Hz, 1H), 4.46–4.29 (m, 5H), 4.20 (dd, *J* = 15.9, 5.5 Hz, 1H), 3.69–3.57 (m, 2H), 2.64–2.50 (m, 4H), 2.43 (s, 3H), 2.06–1.97 (m, 1H), 1.93–1.83 (m, 1H), 0.92 (s, 9H). ^13^C NMR (101 MHz, DMSO-*d*_6_) δ 172.36, 171.59, 170.65, 170.01, 165.54, 165.12, 152.05, 151.86, 150.48, 148.16, 143.53, 139.94, 135.56, 133.41, 131.98, 131.60, 130.09, 129.52, 129.08, 128.11, 127.87, 127.60, 126.69, 125.99, 123.66, 120.85, 119.23, 118.79, 69.33, 59.16, 56.91, 56.78, 42.33, 42.11, 38.38, 35.81, 32.25, 30.58, 26.81, 16.39. HRMS (ESI, positive): calcd. for C_49_H_54_N_7_O_8_S [M + H]^+^: *m*/*z* = 900.3749; found: 900.3752. HPLC *t*_R_ = 13.22 min (purity 99.4%).

*N^1^-(4-(2-(4-(Acrylamidomethyl)benzamido)phenoxy)phenyl)-N^5^-((S)-1-((2S,4R)-4-hydroxy-2-((4-(4-methylthiazol-5-yl)benzyl)carbamoyl)pyrrolidin-1-yl)-3,3-dimethyl-1-oxobutan-2-yl)glutarimide* (**34b**). ^1^H NMR (400 MHz, DMSO-*d*_6_) δ 9.83 (s, 1H), 9.70 (s, 1H), 8.96 (s, 1H), 8.64 (t, *J* = 5.9 Hz, 1H), 8.53 (t, *J* = 6.0 Hz, 1H), 7.88 (d, *J* = 9.3 Hz, 1H), 7.80 (d, *J* = 8.2 Hz, 2H), 7.74 (dd, *J* = 7.5, 2.0 Hz, 1H), 7.54 (d, *J* = 9.0 Hz, 2H), 7.43–7.30 (m, 6H), 7.19–7.10 (m, 2H), 6.96–6.85 (m, 3H), 6.27 (dd, *J* = 17.1, 10.1 Hz, 1H), 6.11 (dd, *J* = 17.1, 2.2 Hz, 1H), 5.61 (dd, *J* = 10.1, 2.2 Hz, 1H), 5.12 (d, *J* = 3.3 Hz, 1H), 4.52 (d, *J* = 9.3 Hz, 1H), 4.46–4.29 (m, 5H), 4.20 (dd, *J* = 15.7, 5.4 Hz, 1H), 3.70–3.60 (m, 2H), 2.42 (s, 3H), 2.32–2.15 (m, 4H), 2.06–1.97 (m, 1H), 1.94–1.84 (m, 1H), 1.82–1.71 (m, 2H), 0.92 (s, 9H). ^13^C NMR (101 MHz, DMSO-*d*_6_) δ 172.36, 172.12, 171.06, 170.14, 165.53, 165.12, 152.13, 151.86, 150.48, 148.16, 143.54, 139.94, 135.51, 133.40, 131.98, 129.54, 129.07, 128.11, 127.87, 127.59, 126.72, 126.67, 125.99, 123.68, 121.00, 119.17, 118.84, 69.33, 59.15, 56.88, 56.79, 42.32, 42.10, 38.39, 36.24, 35.63, 34.68, 26.85, 21.93, 16.38. HRMS (ESI, positive): calcd. for C_50_H_55_N_7_NaO_8_S [M + Na]^+^: *m*/*z* = 936.3731; found: 936.3743. HPLC *t*_R_ = 13.32 min (purity 98.7%).

*N^1^-(4-(2-(4-(Acrylamidomethyl)benzamido)phenoxy)phenyl)-N^6^-((S)-1-((2S,4R)-4-hydroxy-2-((4-(4-methylthiazol-5-yl)benzyl)carbamoyl)pyrrolidin-1-yl)-3,3-dimethyl-1-oxobutan-2-yl)adipamide* (**34c**). ^1^H NMR (400 MHz, DMSO-*d*_6_) δ 9.82 (s, 1H), 9.70 (s, 1H), 8.96 (s, 1H), 8.64 (t, *J* = 5.9 Hz, 1H), 8.53 (t, *J* = 6.0 Hz, 1H), 7.82 (dd, *J* = 13.9, 8.8 Hz, 3H), 7.74 (dd, *J* = 7.4, 2.0 Hz, 1H), 7.54 (d, *J* = 9.0 Hz, 2H), 7.43–7.29 (m, 6H), 7.19–7.09 (m, 2H), 6.96–6.86 (m, 3H), 6.27 (dd, *J* = 17.1, 10.1 Hz, 1H), 6.11 (dd, *J* = 17.1, 2.2 Hz, 1H), 5.61 (dd, *J* = 10.1, 2.2 Hz, 1H), 5.10 (d, *J* = 3.6 Hz, 1H), 4.53 (d, *J* = 9.4 Hz, 1H), 4.47–4.30 (m, 5H), 4.20 (dd, *J* = 15.9, 5.3 Hz, 1H), 3.70–3.58 (m, 2H), 2.43 (s, 3H), 2.31–2.10 (m, 4H), 2.07–1.97 (m, 1H), 1.95–1.84 (m, 1H), 1.61–1.44 (m, 4H), 0.92 (s, 9H). ^13^C NMR (101 MHz, DMSO-*d*_6_) δ 172.36, 171.31, 170.15, 165.54, 165.13, 152.12, 151.85, 150.48, 148.15, 143.53, 139.93, 135.51, 133.41, 131.98, 131.60, 130.09, 129.53, 129.07, 128.11, 127.86, 127.60, 126.70, 125.99, 123.67, 120.97, 119.21, 118.83, 69.32, 59.14, 56.79, 42.33, 42.11, 38.39, 36.60, 35.65, 35.19, 26.84, 25.63, 25.39, 16.39. HRMS (ESI, positive): calcd. for C_51_H_57_N_7_NaO_8_S [M + Na]^+^: *m*/*z* = 950.3887; found: 950.3877. HPLC *t*_R_ = 13.38 min (purity 98.1%).

*N^1^-(4-(2-(4-(Acrylamidomethyl)benzamido)phenoxy)phenyl)-N^7^-((S)-1-((2S,4R)-4-hydroxy-2-((4-(4-methylthiazol-5-yl)benzyl)carbamoyl)pyrrolidin-1-yl)-3,3-dimethyl-1-oxobutan-2-yl)heptanediamide* (**34d**). ^1^H NMR (400 MHz, DMSO-*d*_6_) δ 9.81 (s, 1H), 9.69 (s, 1H), 8.96 (s, 1H), 8.63 (t, *J* = 6.0 Hz, 1H), 8.53 (t, *J* = 6.0 Hz, 1H), 7.84–7.77 (m, 3H), 7.74 (dd, *J* = 7.5, 2.1 Hz, 1H), 7.54 (d, *J* = 9.0 Hz, 2H), 7.44–7.29 (m, 6H), 7.19–7.10 (m, 2H), 6.95–6.86 (m, 3H), 6.27 (dd, *J* = 17.1, 10.1 Hz, 1H), 6.11 (dd, *J* = 17.1, 2.2 Hz, 1H), 5.61 (dd, *J* = 10.1, 2.2 Hz, 1H), 5.10 (d, *J* = 3.6 Hz, 1H), 4.52 (d, *J* = 9.4 Hz, 1H), 4.46–4.30 (m, 5H), 4.20 (dd, *J* = 15.8, 5.5 Hz, 1H), 3.69–3.58 (m, 2H), 2.42 (s, 3H), 2.28–2.08 (m, 4H), 2.05–1.97 (m, 1H), 1.93–1.85 (m, 1H), 1.59–1.44 (m, 4H), 1.31– 1.19 (m, 2H), 0.91 (s, 9H). ^13^C NMR (101 MHz, DMSO-*d*_6_) δ 172.46, 172.37, 171.37, 170.15, 165.54, 165.12, 152.09, 151.86, 150.50, 148.15, 143.54, 139.93, 135.57, 133.41, 131.97, 131.60, 130.08, 129.52, 129.07, 128.11, 127.87, 127.59, 126.70, 126.65, 125.99, 123.66, 120.94, 119.21, 118.80, 69.32, 59.14, 56.75, 42.32, 42.11, 38.40, 36.67, 35.64, 35.25, 28.81, 26.83, 25.69, 25.37, 16.39. HRMS (ESI, positive): calcd. for C_52_H_59_N_7_NaO_8_S [M + Na]^+^: *m*/*z* = 964.4044; found: 964.4049. HPLC *t*_R_ = 10.03 min (purity 97.7%).

*N^1^-(4-(2-(4-(Acrylamidomethyl)benzamido)phenoxy)benzyl)-N^4^-((S)-1-((2S,4R)-4-hydroxy-2-((4-(4-methylthiazol-5-yl)benzyl)carbamoyl)pyrrolidin-1-yl)-3,3-dimethyl-1-oxobutan-2-yl)succinamide* (**34e**). ^1^H NMR (400 MHz, DMSO-*d*_6_) δ 9.71 (s, 1H), 8.96 (s, 1H), 8.63 (t, *J* = 5.9 Hz, 1H), 8.53 (t, *J* = 6.0 Hz, 1H), 8.27 (t, *J* = 5.9 Hz, 1H), 7.88 (d, *J* = 9.3 Hz, 1H), 7.80–7.70 (m, 3H), 7.38 (dd, *J* = 8.5, 8.4 Hz, 4H), 7.32 (d, *J* = 8.3 Hz, 2H), 7.23–7.12 (m, 4H), 6.95–6.88 (m, 3H), 6.27 (dd, *J* = 17.1, 10.1 Hz, 1H), 6.11 (dd, *J* = 17.1, 2.2 Hz, 1H), 5.61 (dd, *J* = 10.1, 2.2 Hz, 1H), 5.09 (d, *J* = 3.5 Hz, 1H), 4.51 (d, *J* = 9.3 Hz, 1H), 4.45–4.29 (m, 5H), 4.25–4.15 (m, 3H), 3.68–3.56 (m, 2H), 2.56–2.50 (m, 1H), 2.43 (s, 3H), 2.41–2.28 (m, 3H), 2.06–1.97 (m, 1H), 1.93–1.82 (m, 1H), 0.91 (s, 9H). ^13^C NMR (101 MHz, DMSO-*d*_6_) δ 172.36, 171.81, 171.69, 170.02, 165.57, 165.13, 155.88, 151.86, 150.07, 148.16, 143.54, 139.93, 134.99, 133.37, 131.97, 131.60, 130.09, 129.89, 129.08, 128.11, 127.88, 127.58, 126.84, 126.74, 126.01, 124.05, 119.48, 118.44, 69.33, 59.15, 56.89, 56.74, 42.33, 42.11, 41.88, 38.37, 35.76, 31.39, 30.98, 26.80, 16.38. HRMS (ESI, positive): calcd. for C_50_H_55_N_7_NaO_8_S [M + Na]^+^: *m*/*z* = 936.3731; found: 936.3743. HPLC *t*_R_ = 13.26 min (purity 96.9%).

*N^1^-(4-(2-(4-(Acrylamidomethyl)benzamido)phenoxy)benzyl)-N^5^-((S)-1-((2S,4R)-4-hydroxy-2-((4-(4-methylthiazol-5-yl)benzyl)carbamoyl)pyrrolidin-1-yl)-3,3-dimethyl-1-oxobutan-2-yl)glutarimide* (**34f**). ^1^H NMR (400 MHz, DMSO-*d*_6_) δ 9.70 (s, 1H), 8.96 (s, 1H), 8.63 (t, *J* = 5.9 Hz, 1H), 8.53 (t, *J* = 6.0 Hz, 1H), 8.23 (t, *J* = 5.9 Hz, 1H), 7.85 (d, *J* = 9.3 Hz, 1H), 7.78–7.73 (m, 3H), 7.41–7.31 (m, 6H), 7.20–7.16 (m, 4H), 6.91 (d, *J* = 8.4 Hz, 3H), 6.26 (dd, *J* = 17.1, 10.1 Hz, 1H), 6.11 (dd, *J* = 17.1, 2.1 Hz, 1H), 5.61 (dd, *J* = 10.1, 2.1 Hz, 1H), 5.10 (d, *J* = 3.6 Hz, 1H), 4.51 (d, *J* = 9.3 Hz, 1H), 4.46–4.28 (m, 5H), 4.25–4.14 (m, 3H), 3.68–3.61 (m, 2H), 2.42 (s, 3H), 2.27–2.08 (m, 4H), 2.05–1.98 (m, 1H), 1.92–1.83 (m, 1H), 1.77–1.64 (m, 2H), 0.91 (s, 9H). ^13^C NMR (101 MHz, DMSO-*d*_6_) δ 172.36, 172.16, 172.15, 170.14, 165.57, 165.13, 155.88, 151.86, 150.04, 148.15, 143.53, 139.93, 135.08, 133.37, 131.96, 131.60, 130.08, 129.89, 129.11, 129.08, 128.10, 127.86, 127.57, 126.84, 126.74, 126.01, 124.05, 119.50, 118.45, 69.32, 59.14, 56.84, 42.32, 42.10, 41.84, 38.38, 35.62, 35.32, 34.84, 26.84, 22.20, 16.38. HRMS (ESI, positive): calcd. for C_51_H_58_N_7_O_8_S [M + H]^+^: *m*/*z* = 928.4062; found: 928.4061. HPLC *t*_R_ = 13.38 min (purity 99.7%).

*N^1^-(4-(2-(4-(Acrylamidomethyl)benzamido)phenoxy)benzyl)-N^6^-((S)-1-((2S,4R)-4-hydroxy-2-((4-(4-methylthiazol-5-yl)benzyl)carbamoyl)pyrrolidin-1-yl)-3,3-dimethyl-1-oxobutan-2-yl)adipamide* (**34g**). ^1^H NMR (400 MHz, DMSO-*d*_6_) δ 9.71 (s, 1H), 8.96 (s, 1H), 8.65 (t, *J* = 6.0 Hz, 1H), 8.53 (t, *J* = 6.0 Hz, 1H), 8.22 (t, *J* = 5.9 Hz, 1H), 7.83–7.72 (m, 4H), 7.41–7.31 (m, 6H), 7.22–7.12 (m, 4H), 6.91 (d, *J* = 8.6 Hz, 3H), 6.27 (dd, *J* = 17.1, 10.1 Hz, 1H), 6.11 (dd, *J* = 17.1, 2.2 Hz, 1H), 5.61 (dd, *J* = 10.1, 2.2 Hz, 1H), 5.11 (d, *J* = 3.6 Hz, 1H), 4.52 (d, *J* = 9.4 Hz, 1H), 4.45–4.30 (m, 5H), 4.26–4.14 (m, 3H), 3.68–3.59 (m, 2H), 2.43 (s, 3H), 2.29–2.19 (m, 1H), 2.13–1.96 (m, 4H), 1.94–1.84 (m, 1H), 1.52–1.41 (m, 4H), 0.91 (s, 9H). ^13^C NMR (101 MHz, DMSO-*d*_6_) δ 172.39, 172.37, 170.14, 165.57, 165.13, 155.89, 151.85, 150.02, 148.15, 143.53, 139.94, 135.10, 133.37, 131.97, 131.60, 130.08, 129.91, 129.07, 128.10, 127.86, 127.58, 126.83, 126.73, 125.99, 125.34, 124.06, 119.51, 118.43, 69.32, 59.14, 56.75, 42.33, 42.11, 41.81, 38.39, 35.65, 35.17, 34.82, 30.87, 26.83, 25.66, 25.51, 16.39. HRMS (ESI, positive): calcd. for C_52_H_60_N_7_O_8_S [M + H]^+^: *m*/*z* = 942.4219; found: 942.4220. HPLC *t*_R_ = 13.44 min (purity 97.8%).

*N^1^-(3-(4-(2-(4-(Acrylamidomethyl)benzamido)phenoxy)phenoxy)propyl)-N^4^-((S)-1-((2S,4R)-4-hydroxy-2-((4-(4-methylthiazol-5-yl)benzyl)carbamoyl)pyrrolidin-1-yl)-3,3-dimethyl-1-oxobutan-2-yl)succinamide* (**34h**). ^1^H NMR (400 MHz, DMSO-*d*_6_) δ 9.68 (s, 1H), 8.96 (s, 1H), 8.65 (t, *J* = 5.9 Hz, 1H), 8.53 (t, *J* = 5.9 Hz, 1H), 7.91–7.78 (m, 4H), 7.74 (d, *J* = 7.6 Hz, 1H), 7.46–7.28 (m, 6H), 7.19–7.04 (m, 2H), 6.93 (dd, *J* = 20.9, 9.1 Hz, 4H), 6.82 (d, *J* = 7.7 Hz, 1H), 6.27 (dd, *J* = 17.1, 10.1 Hz, 1H), 6.11 (dd, *J* = 17.1, 2.0 Hz, 1H), 5.61 (dd, *J* = 10.1, 2.0 Hz, 1H), 5.10 (s, 1H), 4.50 (d, *J* = 9.3 Hz, 1H), 4.46–4.27 (m, 5H), 4.21 (dd, *J* = 15.9, 5.3 Hz, 1H), 3.92 (t, *J* = 6.1 Hz, 2H), 3.71–3.55 (m, 2H), 3.24–3.06 (m, 2H), 2.43 (s, 3H), 2.39–2.20 (m, 4H), 2.06–1.96 (m, 1H), 1.94–1.84 (m, 1H), 1.83–1.74 (m, 2H), 0.91 (s, 9H). ^13^C NMR (101 MHz, DMSO-*d*_6_) δ 172.35, 171.84, 171.71, 170.02, 165.53, 165.14, 155.23, 151.85, 151.03, 150.13, 148.16, 143.56, 139.93, 133.42, 131.97, 131.60, 130.09, 129.22, 129.08, 128.11, 127.87, 127.59, 126.57, 126.55, 126.00, 123.30, 120.49, 118.18, 116.00, 69.33, 66.11, 59.15, 56.86, 56.73, 42.32, 42.11, 38.38, 35.98, 35.76, 31.46, 31.03, 29.36, 26.79, 16.38. HRMS (ESI, positive): calcd. for C_52_H_59_N_7_NaO_9_S [M + Na]^+^: *m*/*z* = 980.3993; found: 980.3984. HPLC *t*_R_ = 13.66 min (purity 98.6%).

*N^1^-(2-(4-(2-(4-(Acrylamidomethyl)benzamido)phenoxy)phenoxy)ethyl)-N^6^-((S)-1-((2S,4R)-4-hydroxy-2-((4-(4-methylthiazol-5-yl)benzyl)carbamoyl)pyrrolidin-1-yl)-3,3-dimethyl-1-oxobutan-2-yl)adipamide* (**34i**). ^1^H NMR (400 MHz, DMSO-*d*_6_) δ 9.68 (s, 1H), 8.96 (s, 1H), 8.64 (t, *J* = 6.0 Hz, 1H), 8.53 (t, *J* = 6.0 Hz, 1H), 7.98 (t, *J* = 5.4 Hz, 1H), 7.81 (d, *J* = 8.2 Hz, 3H), 7.74 (dd, *J* = 7.6, 1.8 Hz, 1H), 7.43–7.31 (m, 6H), 7.18–7.07 (m, 2H), 6.99–6.88 (m, 4H), 6.83 (dd, *J* = 7.8, 1.7 Hz, 1H), 6.27 (dd, *J* = 17.1, 10.1 Hz, 1H), 6.11 (dd, *J* = 17.1, 2.2 Hz, 1H), 5.61 (dd, *J* = 10.1, 2.2 Hz, 1H), 5.10 (d, *J* = 3.6 Hz, 1H), 4.52 (d, *J* = 9.4 Hz, 1H), 4.45–4.36 (m, 4H), 4.36–4.30 (m, 1H), 4.20 (dd, *J* = 16.0, 5.4 Hz, 1H), 3.91 (t, *J* = 5.7 Hz, 2H), 3.67–3.57 (m, 2H), 3.37 (dd, *J* = 11.4, 5.8 Hz, 2H), 2.42 (s, 3H), 2.27–2.19 (m, 1H), 2.13–1.98 (m, 4H), 1.93–1.84 (m, 1H), 1.50–1.38 (m, 4H), 0.91 (s, 9H). ^13^C NMR (101 MHz, DMSO-*d*_6_) δ 172.78, 172.36, 170.14, 165.52, 165.13, 155.04, 151.86, 150.98, 150.31, 148.15, 143.55, 139.94, 133.41, 131.97, 131.59, 130.08, 129.25, 129.07, 128.11, 127.86, 127.59, 126.58, 126.00, 123.35, 120.47, 118.25, 116.09, 69.31, 67.26, 59.13, 56.75, 42.32, 42.10, 38.58, 38.39, 35.64, 35.50, 35.13, 26.83, 25.57, 25.40, 16.38. HRMS (ESI, positive): calcd. for C_53_H_62_N_7_O_9_S [M + H]^+^: *m*/*z* = 972.4324; found: 972.4322. HPLC *t*_R_ = 13.57 min (purity 98.8%).

*N^1^-(2-(4-(2-(4-(Acrylamidomethyl)benzamido)phenoxy)phenoxy)ethyl)-N^8^-((S)-1-((2S,4R)-4-hydroxy-2-((4-(4-methylthiazol-5-yl)benzyl)carbamoyl)pyrrolidin-1-yl)-3,3-dimethyl-1-oxobutan-2-yl)octanediamide* (**34j**). ^1^H NMR (400 MHz, DMSO-*d*_6_) δ 9.68 (s, 1H), 8.96 (s, 1H), 8.65 (t, *J* = 6.0 Hz, 1H), 8.53 (t, *J* = 6.0 Hz, 1H), 7.97 (t, *J* = 5.5 Hz, 1H), 7.80 (dd, *J* = 10.3, 5.1 Hz, 3H), 7.74 (dd, *J* = 7.6, 1.9 Hz, 1H), 7.43–7.29 (m, 6H), 7.17–7.07 (m, 2H), 6.97–6.87 (m, 4H), 6.83 (dd, *J* = 7.8, 1.7 Hz, 1H), 6.27 (dd, *J* = 17.1, 10.1 Hz, 1H), 6.11 (dd, *J* = 17.1, 2.2 Hz, 1H), 5.61 (dd, *J* = 10.1, 2.2 Hz, 1H), 5.10 (d, *J* = 2.8 Hz, 1H), 4.52 (d, *J* = 9.4 Hz, 1H), 4.46–4.26 (m, 5H), 4.20 (dd, *J* = 15.8, 5.4 Hz, 1H), 3.91 (t, *J* = 5.7 Hz, 2H), 3.69–3.58 (m, 2H), 3.36 (dd, *J* = 11.2, 5.6 Hz, 2H), 2.42 (s, 3H), 2.27–2.16 (m, 1H), 2.12–1.95 (m, 4H), 1.93–1.83 (m, 1H), 1.50–1.38 (m, 4H), 1.27–1.16 (m, 4H), 0.91 (s, 9H). ^13^C NMR (101 MHz, DMSO-*d*_6_) δ 172.91, 172.51, 172.37, 170.16, 165.52, 165.14, 155.05, 151.85, 150.96, 150.30, 148.15, 143.55, 139.93, 133.42, 131.97, 131.60, 130.08, 129.26, 129.07, 128.11, 127.86, 127.58, 127.36, 126.57, 125.99, 123.35, 120.46, 118.26, 116.07, 69.31, 68.79, 67.25, 59.13, 58.29, 56.78, 56.73, 42.32, 42.11, 38.59, 38.39, 35.76, 35.64, 35.32, 28.91, 28.89, 26.83, 25.78, 25.61, 16.38. HRMS (ESI, positive): calcd. for C_55_H_66_N_7_O_9_S [M + H]^+^: *m*/*z* = 1000.4637; found: 1000.4638. HPLC *t*_R_ = 13.85 min (purity 98.2%).

*(2S,4R)-1-((S)-2-(6-(4-(2-(4-(Acrylamidomethyl)benzamido)phenoxy)phenoxy)hexan-amido)-3,3-dimethylbutanoyl)-4-hydroxy-N-(4-(4-methylthiazol-5-yl)benzyl)pyrrolidine-2-carboxamide* (**34k**). ^1^H NMR (400 MHz, DMSO-*d*_6_) δ 9.68 (s, 1H), 8.96 (s, 1H), 8.64 (t, *J* = 6.0 Hz, 1H), 8.52 (t, *J* = 6.0 Hz, 1H), 7.87–7.78 (m, 3H), 7.74 (dd, *J* = 7.6, 1.9 Hz, 1H), 7.43–7.30 (m, 6H), 7.17–7.07 (m, 2H), 6.96–6.86 (m, 4H), 6.83 (dd, *J* = 7.8, 1.7 Hz, 1H), 6.27 (dd, *J* = 17.1, 10.1 Hz, 1H), 6.12 (dd, *J* = 17.1, 2.2 Hz, 1H), 5.61 (dd, *J* = 10.1, 2.2 Hz, 1H), 5.10 (d, *J* = 3.6 Hz, 1H), 4.53 (d, *J* = 9.4 Hz, 1H), 4.46–4.29 (m, 5H), 4.20 (dd, *J* = 15.9, 5.5 Hz, 1H), 3.88 (t, *J* = 6.5 Hz, 2H), 3.70–3.59 (m, 2H), 2.42 (s, 3H), 2.32–2.22 (m, 1H), 2.18–2.09 (m, 1H), 2.06–1.98 (m, 1H), 1.94–1.85 (m, 1H), 1.71–1.62 (m, 2H), 1.57–1.46 (m, 2H), 1.41–1.31 (m, 2H), 0.92 (s, 9H). ^13^C NMR (101 MHz, DMSO-*d*_6_) δ 172.45, 172.37, 170.15, 165.53, 165.13, 155.34, 151.85, 151.03, 150.03, 148.15, 143.55, 139.94, 133.43, 131.97, 131.60, 130.08, 129.21, 129.07, 128.11, 128.02, 127.87, 127.59, 126.56, 126.52, 126.00, 123.28, 120.49, 118.18, 115.92, 69.31, 68.78, 68.21, 59.14, 56.76, 42.32, 42.10, 38.40, 35.65, 35.25, 28.90, 26.84, 25.64, 16.38. HRMS (ESI, positive): calcd. for C_51_H_59_N_6_O_8_S [M + H]^+^: *m*/*z* = 915.4110; found: 915.4114. HPLC *t*_R_ = 14.46 min (purity 99%).

*(2S,4R)-1-((S)-2-(7-(4-(2-(4-(Acrylamidomethyl)benzamido)phenoxy)phenoxy)-heptanamido)-3,3-dimethylbutanoyl)-4-hydroxy-N-(4-(4-methylthiazol-5-yl)benzyl)pyrrolidine-2-carboxamide* (**34l**). ^1^H NMR (400 MHz, DMSO-*d*_6_) δ 9.68 (s, 1H), 8.96 (s, 1H), 8.64 (t, *J* = 6.0 Hz, 1H), 8.53 (t, *J* = 6.0 Hz, 1H), 7.81 (d, *J* = 8.2 Hz, 3H), 7.74 (dd, *J* = 7.6, 1.9 Hz, 1H), 7.43–7.30 (m, 6H), 7.18–7.05 (m, 2H), 6.91 (dd, *J* = 21.1, 9.2 Hz, 4H), 6.83 (dd, *J* = 7.9, 1.6 Hz, 1H), 6.27 (dd, *J* = 17.1, 10.1 Hz, 1H), 6.11 (dd, *J* = 17.1, 2.2 Hz, 1H), 5.61 (dd, *J* = 10.1, 2.2 Hz, 1H), 5.10 (s, 1H), 4.53 (d, *J* = 9.4 Hz, 1H), 4.46–4.29 (m, 5H), 4.20 (dd, *J* = 15.8, 5.5 Hz, 1H), 3.88 (t, *J* = 6.4 Hz, 2H), 3.68–3.59 (m, 2H), 2.42 (s, 3H), 2.31–2.20 (m, 1H), 2.16–2.06 (m, 1H), 2.06–1.96 (m, 1H), 1.94–1.84 (m, 1H), 1.70–1.60 (m, 2H), 1.55–1.43 (m, 2H), 1.42–1.33 (m, 2H), 1.33–1.23 (m, 2H), 0.92 (s, 9H). ^13^C NMR (101 MHz, DMSO-*d*_6_) δ 172.50, 172.37, 170.15, 165.52, 165.13, 155.34, 151.85, 151.02, 150.04, 148.15, 143.55, 139.94, 133.43, 131.98, 131.60, 130.08, 129.21, 129.07, 128.11, 127.86, 127.59, 126.56, 126.52, 125.99, 123.28, 120.48, 118.19, 115.93, 69.31, 68.25, 59.13, 56.78, 56.73, 42.32, 42.10, 38.40, 35.64, 35.27, 29.06, 28.85, 26.83, 25.82, 25.69, 16.38. HRMS (ESI, positive): calcd. for C_52_H_60_N_6_NaO_8_S [M + Na]^+^: *m*/*z* = 951.4091; found: 951.4091. HPLC *t*_R_ = 14.73 min (purity 97.3%).

*(2S,4R)-1-((S)-2-(4-(2-(4-(2-(4-(Acrylamidomethyl)benzamido)phenoxy)phenyl)-acetamido)butanamido)-3,3-dimethylbutanoyl)-4-hydroxy-N-(4-(4-methylthiazol-5-yl)benzyl)pyrrolidine-2-carboxamide* (**34m**). ^1^H NMR (400 MHz, DMSO-*d*_6_) δ 9.69 (s, 1H), 8.96 (s, 1H), 8.63 (t, *J* = 5.9 Hz, 1H), 8.53 (t, *J* = 6.0 Hz, 1H), 7.97 (t, *J* = 5.4 Hz, 1H), 7.86 (d, *J* = 9.3 Hz, 1H), 7.79–7.71 (m, 3H), 7.38 (dd, *J* = 8.5, 8.5 Hz, 4H), 7.32 (d, *J* = 8.3 Hz, 2H), 7.23–7.12 (m, 4H), 6.96–6.86 (m, 3H), 6.27 (dd, *J* = 17.1, 10.1 Hz, 1H), 6.11 (dd, *J* = 17.1, 2.2 Hz, 1H), 5.61 (dd, *J* = 10.1, 2.2 Hz, 1H), 5.10 (d, *J* = 3.6 Hz, 1H), 4.52 (d, *J* = 9.3 Hz, 1H), 4.46–4.29 (m, 5H), 4.20 (dd, *J* = 16.0, 5.5 Hz, 1H), 3.69–3.59 (m, 2H), 3.33 (s, 2H), 3.00 (dd, *J* = 13.5, 6.8 Hz, 2H), 2.42 (s, 3H), 2.29–2.19 (m, 1H), 2.17–2.07 (m, 1H), 2.05–1.97 (m, 1H), 1.93–1.84 (m, 1H), 1.64–1.54 (m, 2H), 0.92 (s, 9H). ^13^C NMR (101 MHz, DMSO-*d*_6_) δ 172.36, 172.15, 170.43, 170.10, 165.59, 165.13, 155.63, 151.86, 150.01, 148.16, 143.49, 139.93, 133.41, 131.97, 131.88, 131.60, 130.72, 130.09, 129.88, 129.08, 128.10, 127.87, 127.59, 126.75, 126.71, 126.00, 124.00, 119.50, 118.38, 69.32, 59.14, 56.84, 42.33, 42.11, 41.99, 38.85, 38.39, 35.68, 32.95, 26.83, 26.10, 16.38. HRMS (ESI, positive): calcd. for C_51_H_58_N_7_O_8_S [M + H]^+^: *m*/*z* = 928.4062; found: 928.4080. HPLC *t*_R_ = 13.25 min (purity 97.8%).

*(2S,4R)-1-((S)-2-(5-(2-(4-(2-(4-(Acrylamidomethyl)benzamido)phenoxy)phenyl)-acetamido)pentanamido)-3,3-dimethylbutanoyl)-4-hydroxy-N-(4-(4-methylthiazol-5-yl)benzyl)pyrrolidine-2-carboxamide* (**34n**). ^1^H NMR (400 MHz, DMSO-*d*_6_) δ 9.69 (s, 1H), 8.96 (s, 1H), 8.63 (t, *J* = 5.9 Hz, 1H), 8.53 (t, *J* = 6.0 Hz, 1H), 7.95 (t, *J* = 5.5 Hz, 1H), 7.81 (d, *J* = 9.3 Hz, 1H), 7.75 (dd, *J* = 9.6, 5.3 Hz, 3H), 7.38 (dd, *J* = 8.4, 8.4 Hz, 4H), 7.32 (d, *J* = 8.3 Hz, 2H), 7.22–7.10 (m, 4H), 6.97–6.85 (m, 3H), 6.27 (dd, *J* = 17.1, 10.1 Hz, 1H), 6.11 (dd, *J* = 17.1, 2.2 Hz, 1H), 5.61 (dd, *J* = 10.1, 2.2 Hz, 1H), 5.10 (d, *J* = 3.6 Hz, 1H), 4.52 (d, *J* = 9.4 Hz, 1H), 4.46–4.29 (m, 5H), 4.20 (dd, *J* = 15.8, 5.4 Hz, 1H), 3.69–3.58 (m, 2H), 3.32 (s, 2H), 3.00 (dd, *J* = 12.6, 6.6 Hz, 2H), 2.43 (s, 3H), 2.28–2.19 (m, 1H), 2.15–2.06 (m, 1H), 2.05–1.97 (m, 1H), 1.93–1.84 (m, 1H), 1.50–1.41 (m, 2H), 1.39–1.32 (m, 2H), 0.91 (s, 9H). ^13^C NMR (101 MHz, DMSO-*d*_6_) δ 172.37, 170.34, 170.13, 165.59, 165.12, 155.60, 151.86, 150.02, 148.15, 143.49, 139.94, 133.42, 131.97, 131.60, 130.69, 130.09, 129.88, 129.07, 128.10, 127.86, 127.59, 126.74, 126.70, 125.99, 124.00, 119.48, 118.37, 69.32, 59.14, 56.75, 42.33, 42.11, 41.99, 38.88, 38.40, 35.65, 35.01, 29.24, 26.83, 23.43, 16.39. HRMS (ESI, positive): calcd. for C_52_H_60_N_7_O_8_S [M + H]^+^: *m*/*z* = 942.4219; found: 942.4227. HPLC *t*_R_ = 13.32 min (purity 97.9%).

### 3.4. Experimental for Stability Testing

The chemical stability of the synthesized compounds was measured using HPLC assay as in the previously reported procedure [20].

The microsomal stability of selected compounds was evaluated using mouse (CD-1) liver microsomes. The test compounds were incubated at a concentration of 25 μM with microsomal preparations under NADPH-supplemented conditions, and the rate of parent compound depletion was determined over time. Diclofenac sodium (25 μM) was included as a positive control to confirm the validity and performance of the assay.

The test compounds were dissolved in a suitable stock concentration (10 mM in DMSO). Twelve 1.5 mL microcentrifuge tubes were prepared for each test compound (for 0, 10, 20, 30, 60, and 120 min, including duplicate tubes) and the positive and negative controls. The final incubation volume for each tube was 700 µL. Incubations were carried out using 0.1 M potassium phosphate buffer (pH 7.4) (634 μL), mouse (CD-1) microsomes purchased from ThermoFisher 20 mg/mL (17 µL), 10 mM of test compound or positive control (7 μL), 1 M MgCl_2_ (7 µL), and 20 mM NADPH (35 μL). The mixtures, except NADPH, were pre-incubated for 10 min in a thermoshaker maintained at 37 °C, and reactions were initiated by the addition of the NADPH solution. The samples were mixed well by briefly vortexing and then incubated at 37 °C. The samples were collected at six time points (0, 10, 20, 30, 60, and 120 min) to monitor compound degradation. At each time point, 100 μL of the incubation mixture was withdrawn and immediately quenched with an equal volume of ice-cold acetonitrile to terminate enzymatic activity.

The quenched samples were vortexed and centrifuged at 10,000× *g* for 5 min at 4 °C. The supernatants were then put into HPLC vials for analysis. Chromatographic separation was performed using a Shimadzu HPLC system equipped with two LC-10AD pumps and an SIL-HAT autosampler. A LiChrosphere 100 RP-18e column (5 μm, 100 mm; Merck) was used as the stationary phase. The mobile phase consisted of a methanol–water gradient containing 0.05% trifluoroacetic acid (TFA). The injection volume was 20 μL, and analytes were detected using an SPD-M10A VP PDA UV–Vis detector set at 254 or 280 nm.

The remaining concentration of the parent compound at each time point was determined, and the extent of metabolism (as substrate depletion) and % recovery of the test compounds and positive control were calculated.

### 3.5. Experimental Procedure for Cellular Testing

To ascertain the EC_50_ values of compounds, 5 × 10^3^ HEK293T, Panc-1 or HCT-116 cells were seeded in a 96-well plate. Subsequently, a progressive dilution series of the compounds was executed (starting concentration of 20 or 50 µM). These compounds were added to the cells alongside dimethyl sulfoxide (DMSO) as a control condition. Following a 72 h treatment duration, the cell viability was quantified using CellTiter-Glo^®^ (G7572; Promega, Madison, WI, USA) and adjusted relative to the DMSO control. The determination of EC_50_ values was achieved through the utilization of GraphPad Prism software (version 10.1.1) employing non-linear regression analysis.

### 3.6. Western Blot Analysis

For Western blot analysis, 1.5 × 10^5^ Panc-1 or 3 × 10^5^ HEK293T cells were seeded in a 12-well plate. On the next day, compounds were added for 6 h at indicated concentrations. Additional samples were generated by adding MG-132 (Invivogen) for inhibition of proteasomal degradation. Harvested cells were lysed in total lysis buffer (50 mM Tris (pH 7.4), 50 mM NaCl, 1% SDS (*v*/*v*), 2 mM MgCl_2_) supplemented with 0.5 µL Benzonase^®^ (Merck Millipore, Burlington, MA, USA) per 100 µL. Protein concentrations were determined using the DC Protein Assay (Bio-Rad, Hercules, CA, USA), according to manufacturer’s instructions, and the GloMAX Discover (Promega) at 600 nm. A BSA standard curve (0 to 10 mg/mL) served as quantification control. For SDS-PAGE, 4x NuPAGE LDS Sample Buffer (Invitrogen) supplemented with 100 mM DTT was added to the protein lysates and incubated for 3 min at 95 °C. Separation of proteins was accomplished on a NuPAGE Novex 4–12% Bis-Tris protein gel (Invitrogen, Carlsbad, CA, USA) with MOPS SDS-running buffer (50 mM MOPS, 50 mM Tris, 0.1% SDS (*v*/*v*), 1 mM EDTA). The SeeBlue Plus2 Pre-Stained Protein Standard (Thermo Fisher) served as marker for size detection. Subsequently, proteins were transferred using NuPAGE transfer buffer (50 mM Tris, 40 mM glycerol, 0.04% SDS (*v*/*v*), 10% methanol (*v*/*v*)) by wet blotting onto a nitrocellulose membrane (GE Healthcare, Osaka, Japan). Finally, membranes were blocked with 5% (*w*/*v*) milk. Protein expression was analyzed by Western blotting with primary antibodies by using fluorescence-coupled secondary antibodies and an infrared scanner (Li-Cor). The following antibodies were used: MYC (Cell Signaling, Danvers, MA, USA (18583S)), VCL (Sigma (V9131)). Western blotting was carried out in triplicate. Time-course analysis in HEK293T17 cells was done equally at 2, 4, 6, 8, 16 and 24 h of incubation with the most active compounds.

For luciferase-based analysis of potential degradation of the most active compounds, a HiBiT-based assay was performed. Therefore, 8 × 10^5^ HEK293T17 cells were transfected with 2 µg pc-HiBiT-MYC and 2 µg LgBiT Expression Vector (Promega, N2681) using 3.75 µL Lipofectamine 3000. Subsequently, 2 × 10^4^ HEK293T17 cells were seeded into each well of a 96-well plate and treated with these compounds for 2, 4, 6 and 8 h. Afterwards, luciferase activity was measured according to the manufacturer’s protocol (Promega, N3040) without adding the LgBiT protein to the lysate. Luminescence was measured using a Glomax Discover (Promega).

## 4. Conclusions

A series of CRBN- and VHL-based PROTACs derived from the MYC inhibitor EN4 were designed, synthesized, and fully characterized, demonstrating high chemical purity. However, cellular evaluation of the newly synthesized compounds revealed limited biological activity in HEK293T, Panc-1, and HCT-116 cells, with only moderate reductions in cell viability and EC_50_ values in the mid-micromolar range.

Compounds for which EC_50_ values could be determined in HEK293T cells were further investigated for their ability to induce MYC degradation at concentrations corresponding to their EC_50_ values. However, only for the VHL PROTACs **34f** and **34g** was concentration-dependent degradation observed after 2 h or 4 h, whereas at longer incubation (up to 24 h), no MYC degradation was observed. Even for the reference compound KL4-219A, a covalent MYC binder described above, degradation was only detected at high concentrations (50× EC_50_). For the synthesized PROTACs, evaluation at this concentration (50× EC_50_) was not feasible due to limited solubility.

To better understand the reasons for the limited biological effects and the lack of degradation, we also assessed the chemical and metabolic stability in vitro. While most compounds, with one exception, were chemically stable in the assay medium, microsomal stability testing revealed rapid metabolic degradation for nearly all compounds under investigation (Table 3).

Taken together, these results indicate that most of the synthesized PROTACs are ineffective as MYC degraders under the tested conditions. The relatively low activity may be attributed to factors such as insufficient cellular permeation due to the large molecular size of the PROTACs (Figure S7, Supplementary Information) as well as rapid metabolic degradation that we observed in the microsomal testing. In addition, the inactivity of several degraders may result from insufficient binding to MYC or the recruited E3 ligase in cells, or from inefficient formation of the ternary complex. Therefore, further structural optimization and mechanistic studies will be required.

## Figures and Tables

**Table 1:** Chemical structures of developed CRBN-based PROTACs and cellular activity.

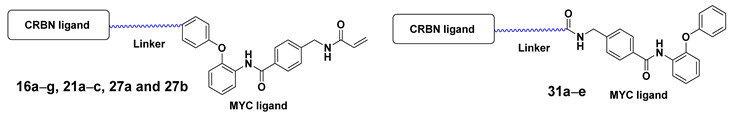					
**Cmpd. ID**	**CRBN Ligand**	**Linker**	**% Inhibition @20 μM** **PANC-1**	**% Inhibition @50 μM** **HEK293T**	**EC_50_ μM** **HCT-116**
**16a** (Abd11)	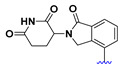	* 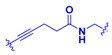 *	n.i.	n.t.	n.i.
**16b** (Abd1)	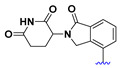	* 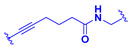 *	n.i.	13 ± 3	n.i.
**16c** (Abd9)	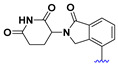	* 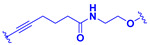 *	n.i.	n.t.	n.i.
**16d** (Abd12)	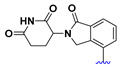	* 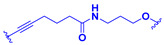 *	n.i.	n.t.	n.i.
**16e** (Abd44)	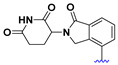	* 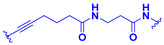 *	n.i.	n.t.	n.i.
**16f** (Abd48)	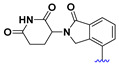	* 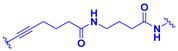 *	20 ± 2	14 ± 1	n.i.
**16g** (Abd8)	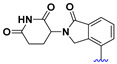	* 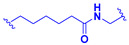 *	12 ± 1	11 ± 1	n.i.
**21a** (Abd42)	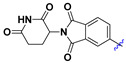	* 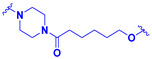 *	6 ± 1	43 ± 1	n.i.
**21b** (Abd25)	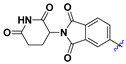	* 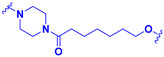 *	n.i.	n.t.	n.i.
**21c** (Abd3)	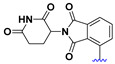	* 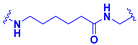 *	n.i.	18 ± 2	n.i.
**27a** (Abd41)	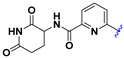	* 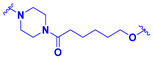 *	n.i.	n.t.	40.2 ± 2.0
**27b** (Abd40)	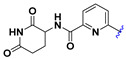	* 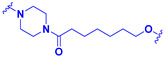 *	n.i.	n.t.	n.i.
**32a** (Abd63)	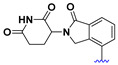	* 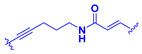 *	n.i.	n.t.	n.i.
**32b** (Abd66)	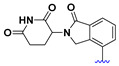	* 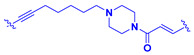 *	6 ± 1	n.t.	n.i.
**32c** (Abd59)	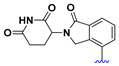	* 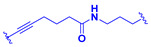 *	9 ± 1	16 ± 1	n.i.
**32d** (Abd62)	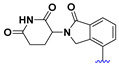	* 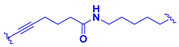 *	14 ± 1	43 ± 2	n.i.
**32e** (Abd61)	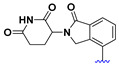	* 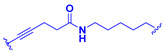 *	8 ± 1	n.t.	n.i.

n.i. = no inhibition, n.t. = not tested.

**Table 2:** Chemical structures of developed VHL-based PROTACs and cellular activity.

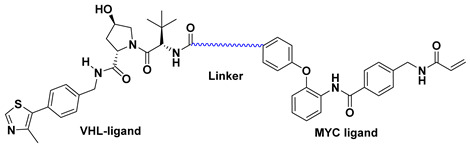					
**Cmpd. ID**	**Linker**	**% Cytotox PANC-1** **@20 μM**	**% Cytotox HEK293T** **@25 μM** **@50 μM**	**EC_50_ μM** **HEK293T**	**EC_50_ μM** **HCT-116**
**34a** (Abd19)	* 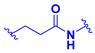 *	n.i.	n.t.		n.i.
**34b** (Abd20)	* 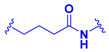 *	n.i.	n.t.		58.0 ± 7.3
**34c** (Abd21)	* 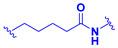 *	n.i.	n.t.		52.7 ± 6.9
**34d** (Abd22)	* 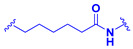 *	n.i.	n.t.		n.i.
**34e** (Abd18)	* 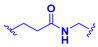 *	n.i.	12 ± 1 38 ± 3	90.0 ± 5.0	>100
**34f** (Abd2)	* 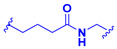 *	17 ± 2	33 ± 3 63 ± 6	37.3 ± 2.5	39.7 ± 1.2
**34g** (Abd5)	* 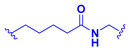 *	13 ± 2	18 ± 2 43 ± 4	61.6 ± 5.1	63.0 ± 8.1
**34h** (Abd17)	* 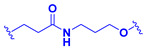 *	8 ± 1	13 ± 1 44 ± 2	>50	>100
**34i** (Abd7)	* 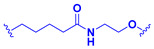 *	n.i.	n.t.		n.i.
**34j** (Abd14)	* 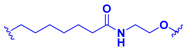 *	n.i.	n.t.		n.i.
**34k** (Abd43)	* 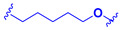 *	n.i.	n.t.		n.i.
**34l** (Abd13)	* 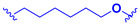 *	6 ± 1	37 ± 3 55 ± 6	>50	>100
**34m** (Abd23)	* 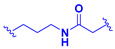 *	n.i.	n.t.		50.3 ± 2.8
**34n** (Abd24)	* 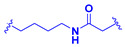 *	n.i.	n.t.		52.6 ± 17.6
EN4		6.0 ± 0.4		11.3 ± 0.6	>25
KL4-219A		1.57 ± 0.11		0.99 ± 0.04	0.82 ± 0.032

n.i. = no inhibition, n.t. = not tested.

**Table 3:** Microsomal stability of PROTACs. Compounds were incubated for 2 h using mouse (CD-1) liver microsomes.

	0 min—cpd. Remaining %	10 min—cpd. Remaining %	20 min—cpd. Remaining %	30 min—cpd. Remaining %	60 min—cpd. Remaining %	120 min—cpd. Remaining %
**34f**	100	46.0 ± 1.8	26.4 ± 0.6	17.1 ± 1.1	3.9 ± 0.2	1.1 ± 0.1
**34g**	100	49.9 ± 1.1	23.6 ± 0.1	15.9 ± 1.1	4.1 ± 0.2	1.4 ± 0.1
**34l**	100	38.3 ± 1.7	33.8 ± 1.1	25.6 ± 0.1	7.5 ± 0.1	2.6 ± 0.1
**34h**	100	21.0 ± 0.6	13.8 ± 0.5	8.7 ± 0.3	3.7 ± 0.2	2.6 ± 0.1
**34e**	100	44.0 ± 3.3	27.9 ± 0.4	17.4 ± 0.2	5.3 ± 0.2	1.8 ± 0.
**16f**	100	21.7 ± 0.2	13.8 ± 0.5	8.1 ± 0.2	2.9 ± 0.1	0.8 ± 0.0
**27a**	100	18.7 ± 0.3	15.2 ± 0.1	14.1 ± 0.4	11.0 ± 0.4	9.1 ± 0.3
**32c**	100	63.9 ± 0.9	36.6 ± 2.7	38.7 ± 0.1	24.0 ± 0.5	12.4 ± 0.1
**32d**	100	56.6 ± 0.1	52.5 ± 0.2	43.9 ± 1.4	19.5 ± 0.3	7.1 ± 0.1
KL4-219A	100	50.0 ± 3.1	24.6 ± 0.4	13.6 ± 0.4	2.6 ± 0.2	0.6 ± 0.1
EN4	100	52.4 ± 2.3	16.1 ± 0.4	14.7 ± 0.3	15.9 ± 0.2	15.1 ± 0.7

## Data Availability

The original contributions presented in this study are included in the Supplementary Material. Further inquiries can be directed to the corresponding author(s).

## Supplementary Materials

The following supporting information can be downloaded at: https://www.mdpi.com/article/10.3390/molecules31061011/s1, Figure S1. EC_50_ determination of tested potential MYC degraders in Panc-1 cells. Cell viability was measured after 72 h with CellTiter-Glo (Promega). n = 4. Figure S2. EC_50_ determination of tested potential MYC degraders in HEK293T17 cells. Cell viability was measured after 72 h with CellTiter-Glo (Promega). n = 4. Figure S3. EC_50_ determination of tested potential MYC degraders in HCT-116 cells. Cell viability was measured after 72 h with CellTiter-Glo (Promega). n = 4. Figure S4. Time and concentration course analysis of most active potential MYC degraders in HEK293T17 cells analyzed by western blotting. The bar diagram show quantification of MYC protein levels relative to VCL expression. n = 1. Figure S5. Time course analysis of most active potential MYC degraders at 50 µM using HiBiT-MYC and LgBiT overexpression in HEK293T17 cells. n = 3. Figure S6. Non-enzymatic stability testing. Stability of PROTACs in assay medium at 37 °C. Figure S7. Predicted ADME properties (in silico) using SwissADME* (https://www.swissadme.ch). Figure S8. ^1^H-NMR, ^13^C-NMR, HPLC chromatogram, and HRMS spectra. Figure S9. HPLC chromatograms, microsomal stability testing.
