# Supplementary material for: Insights into the Design of MYC-Targeting Proteolysis Targeting Chimeras (PROTACs)
Source: Molecules. 2026 Mar 17;31(6):1011. doi: 10.3390/molecules31061011 (PMC13029781; doi:10.3390/molecules31061011)
Supplement: Supplementary file 1 [file molecules-31-01011-s001.zip › molecules-4141664-supplementary.pdf]

Supporting Information

Insights into the Design of MYC-Targeting Proteolysis Targeting Chimeras (PROTACs)

Abdallah M. Alfayomy[1,2], Sven Hagemann[3], Matthias Schmidt[1], Ali Fouad[2], Mohamed Ayman El-Zahabi[4], Stefan Hüttelmaier[3], Wolfgang Sippl[1]

1 Department of Medicinal Chemistry, Institute of Pharmacy, Martin-Luther-University of Halle-Wittenberg, 06120 Halle (Saale), Germany

2 Department of Pharmaceutical Chemistry, Faculty of Pharmacy, Al-Azhar University, Assiut, 71524, Egypt

3 Department of Molecular Medicine, Faculty of Medicine, Martin-Luther University Halle-Wittenberg, 06120 Halle (Saale), Germany

4 Pharmaceutical Medicinal Chemistry & Drug Design Department, Faculty of Pharmacy (Boys), Al-Azhar University, Cairo, Egypt

---

Content page

S1-S3. IC_{50} plots PANC-1, HEK293 and HCT-116 2-6
S4. Time and concentration course analysis, Western blots 7-8
S5. Time course analysis HiBiT-MYC and LgBiT 9
S6. HPLC stability testing 10
S7. Predicted in silico ADME parameters 10
S8. 1H-NMR, 13C-NMR, HPLC chromatogram, and Mass spectra 11-72
S9. HPLC chromatrograms, microsomal stability testing 73-78

Figure S1. EC_{50} determination of tested potential MYC degraders in Panc-1 cells. Cell viability was measured after 72 h with CellTiter-Glo (Promega). n = 4

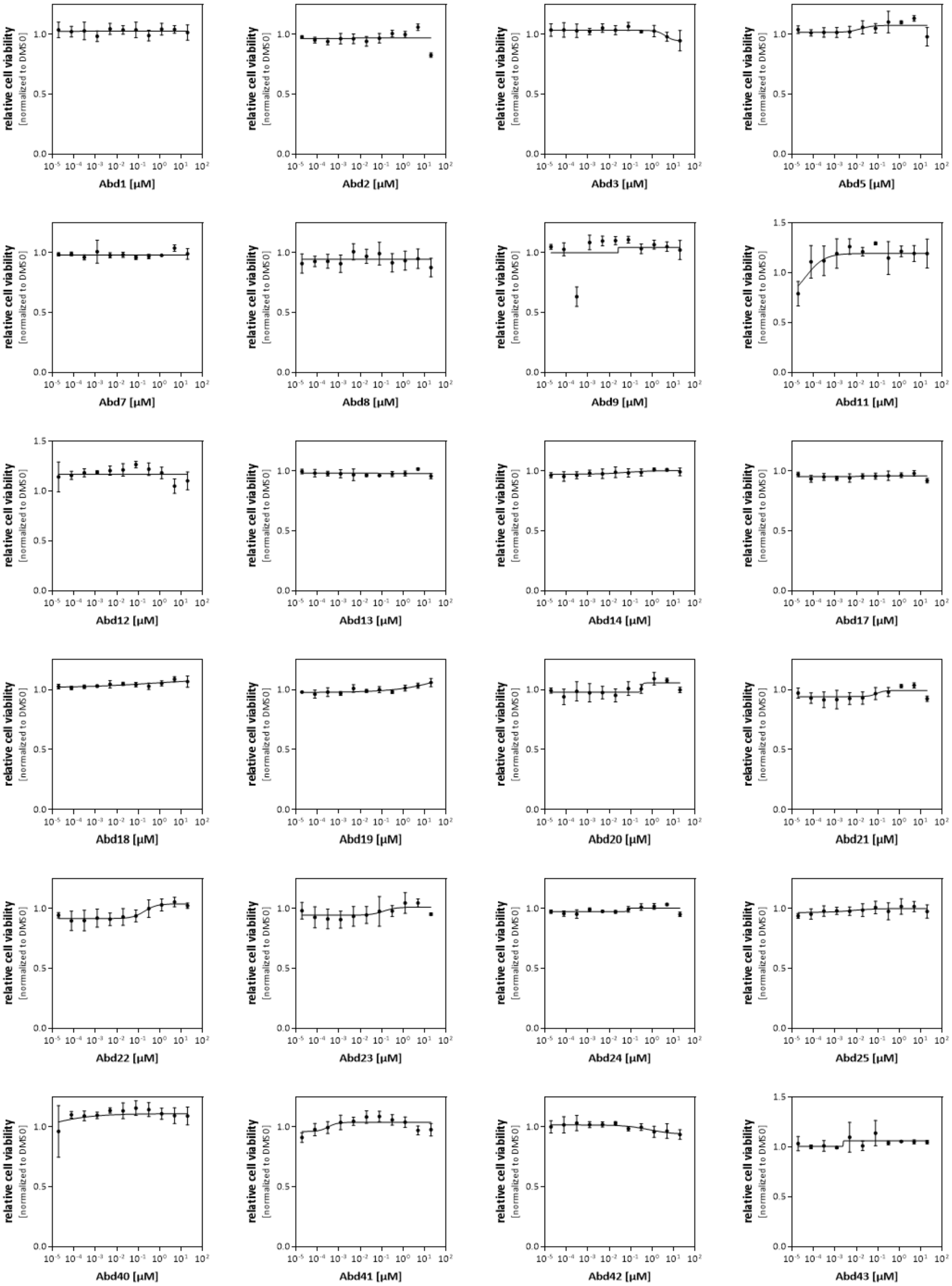

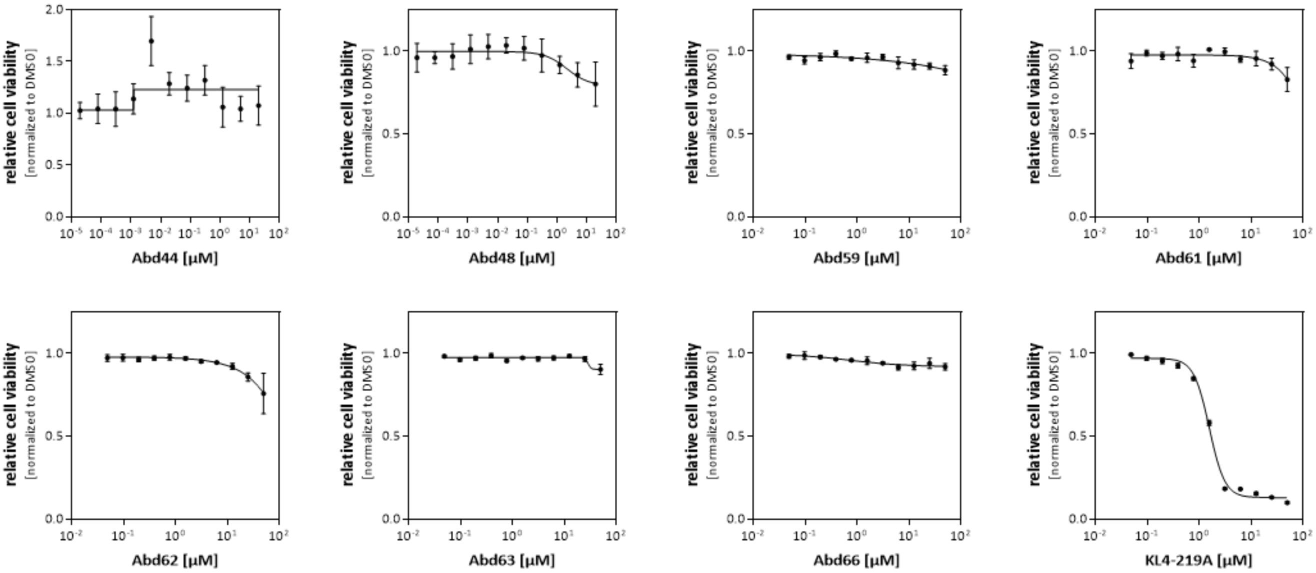
relative cell viability
[normalized to DMSO]
Abd44 [µM]
Abd48 [µM]
Abd59 [µM]
Abd61 [µM]
Abd62 [µM]
Abd63 [µM]
Abd66 [µM]
KL4-219A [µM]

Figure S2. EC_{50} determination of tested potential MYC degraders in HEK293T17 cells. Cell viability was measured after 72 h with CellTiter-Glo (Promega). n = 4.

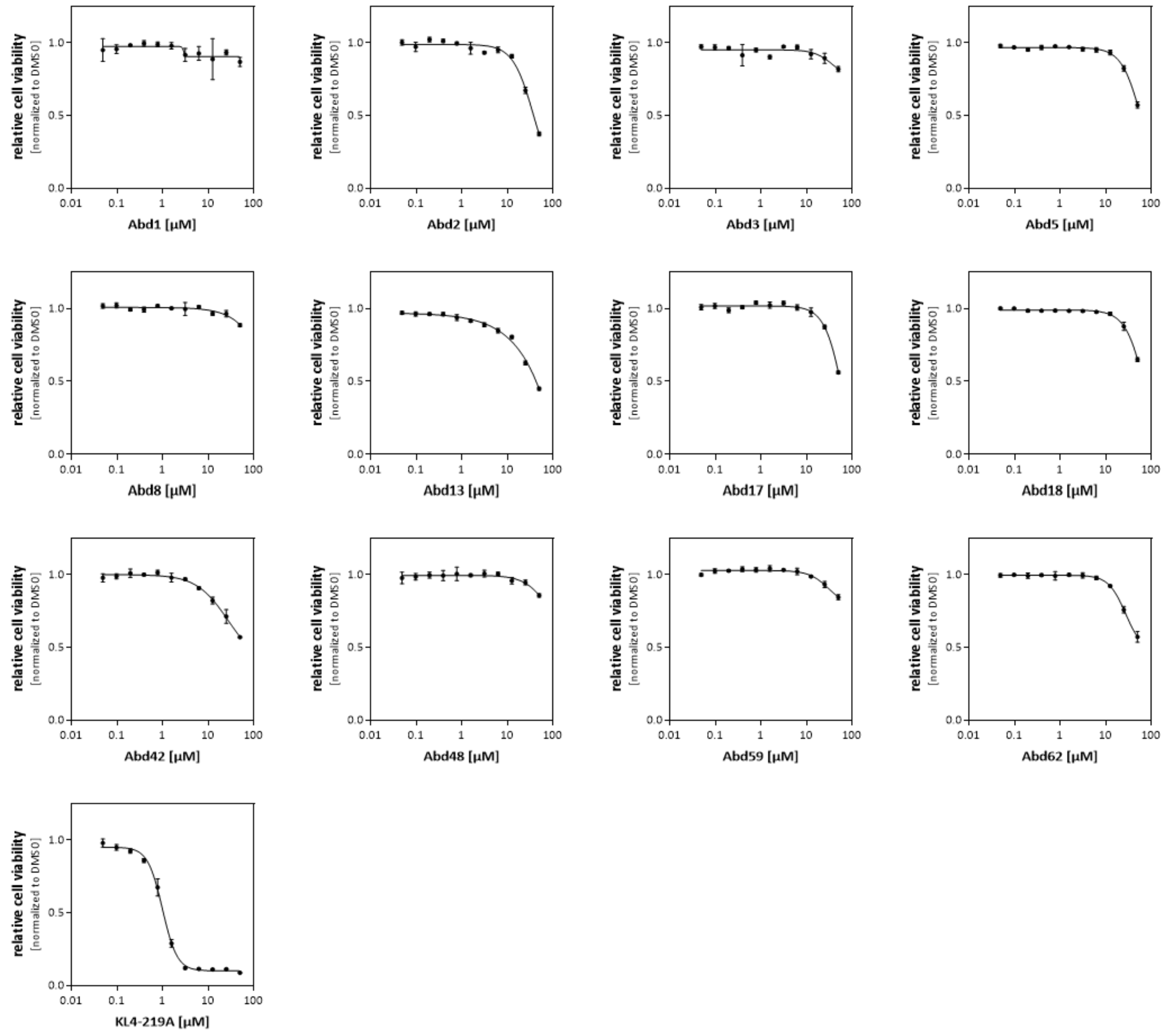

Figure S3. EC_{50} determination of tested potential MYC degraders in HCT-116 cells. Cell viability was measured after 72 h with CellTiter-Glo (Promega). n = 4

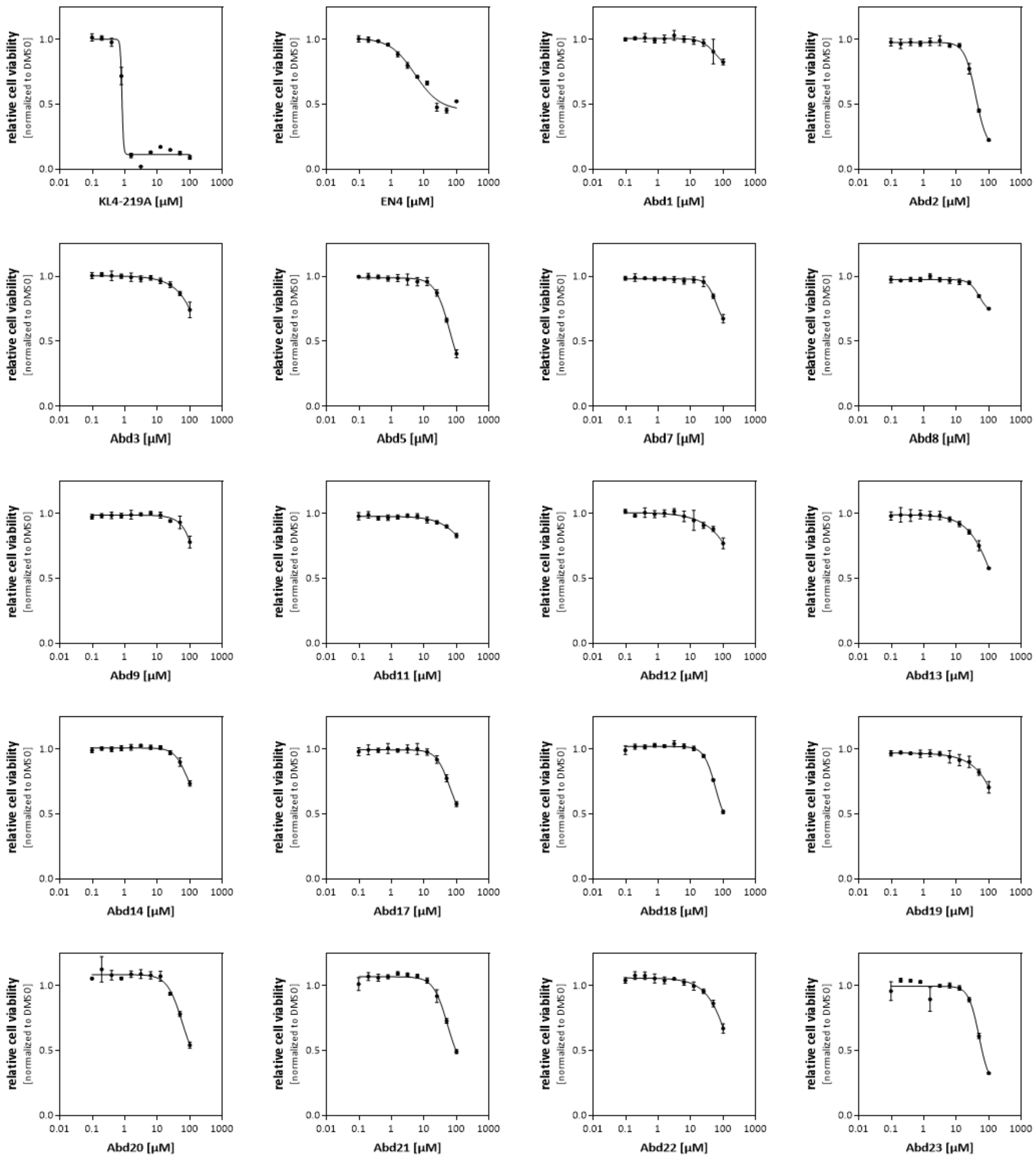

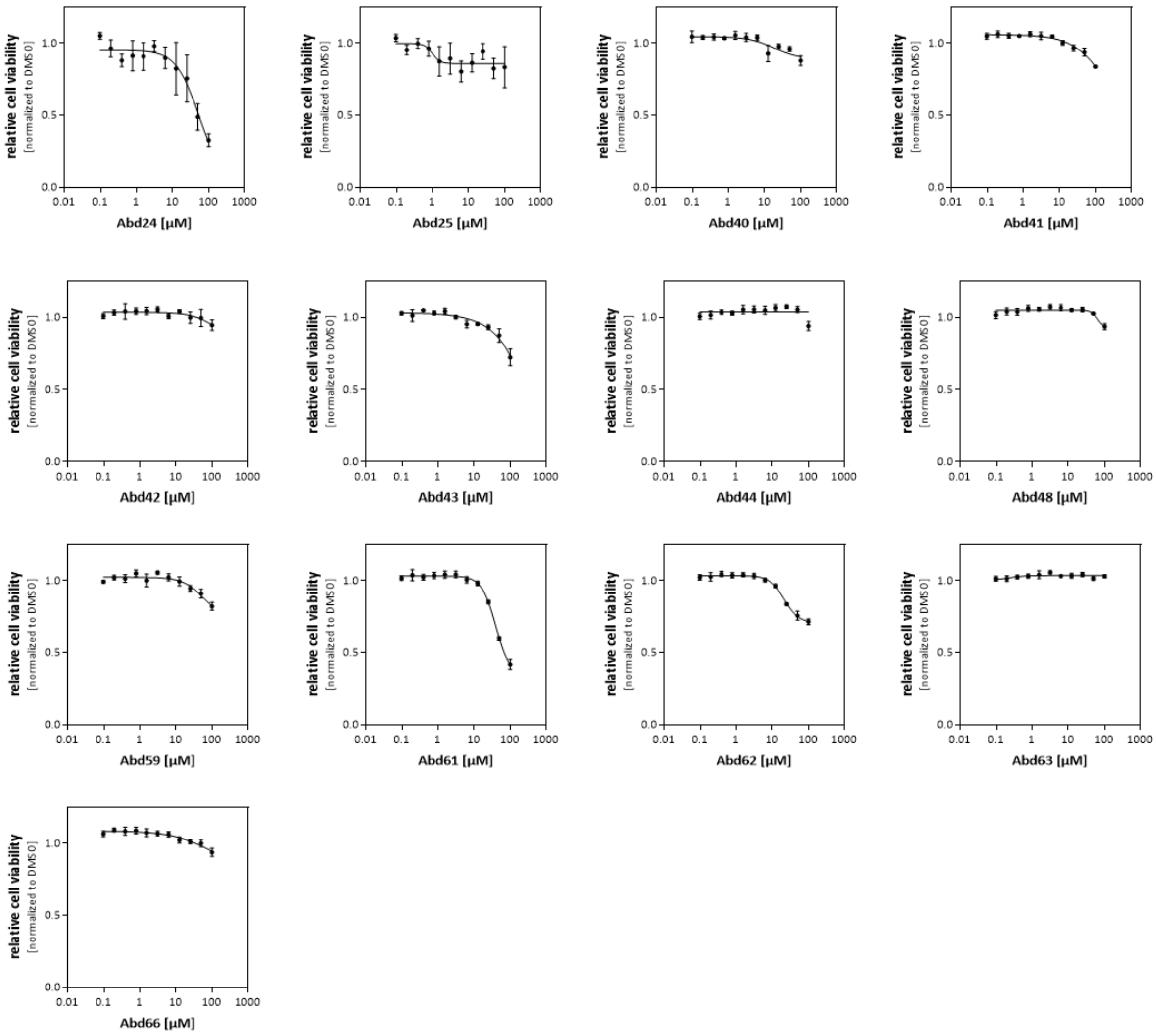
relative cell viability
[normalized to DMSO]
Abd24 [µM]
Abd25 [µM]
Abd40 [µM]
Abd41 [µM]
Abd42 [µM]
Abd43 [µM]
Abd44 [µM]
Abd48 [µM]
Abd59 [µM]
Abd61 [µM]
Abd62 [µM]
Abd63 [µM]
Abd66 [µM]

Figure S4. Time and concentration course analysis of most active potential MYC degraders in HEK293T17 cells analyzed by western blotting. The bar diagram show quantification of MYC protein levels relative to VCL expression. n = 1

34g

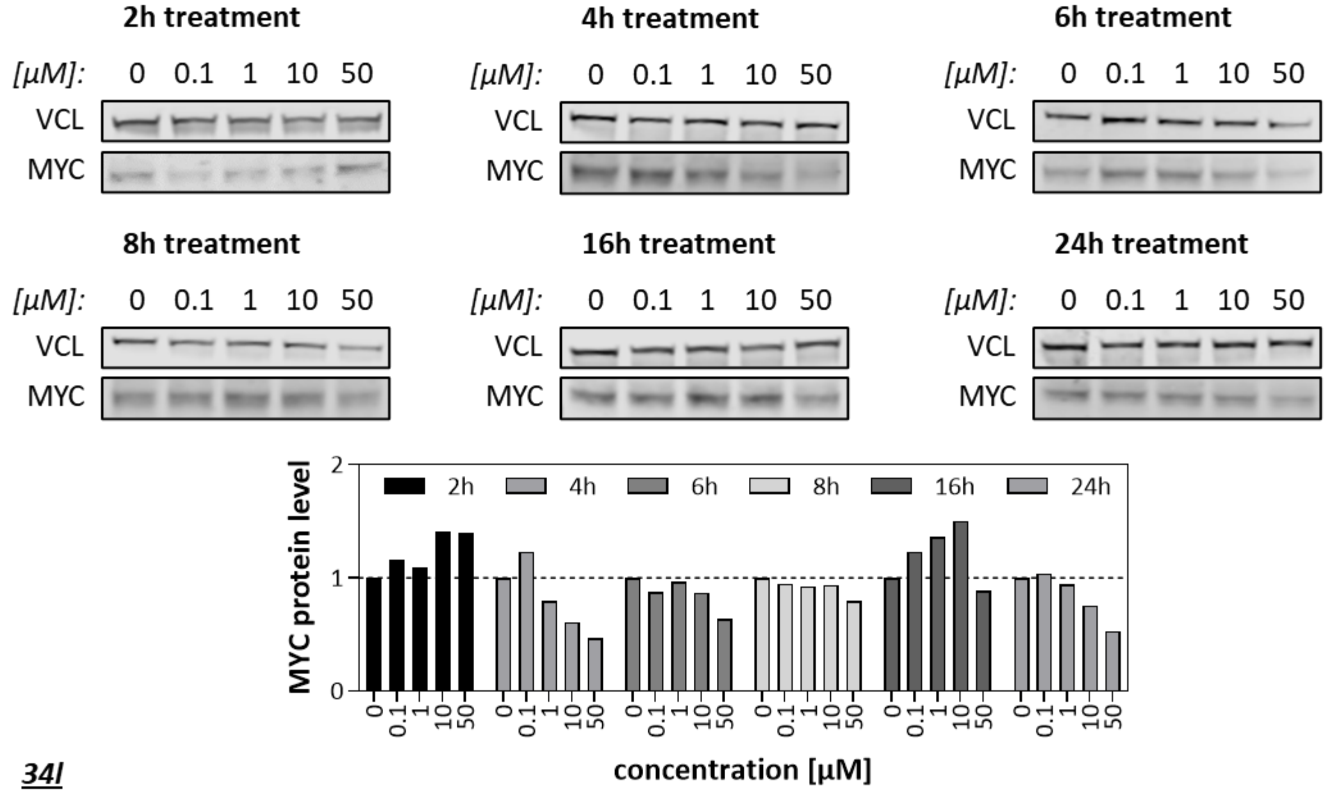

34l

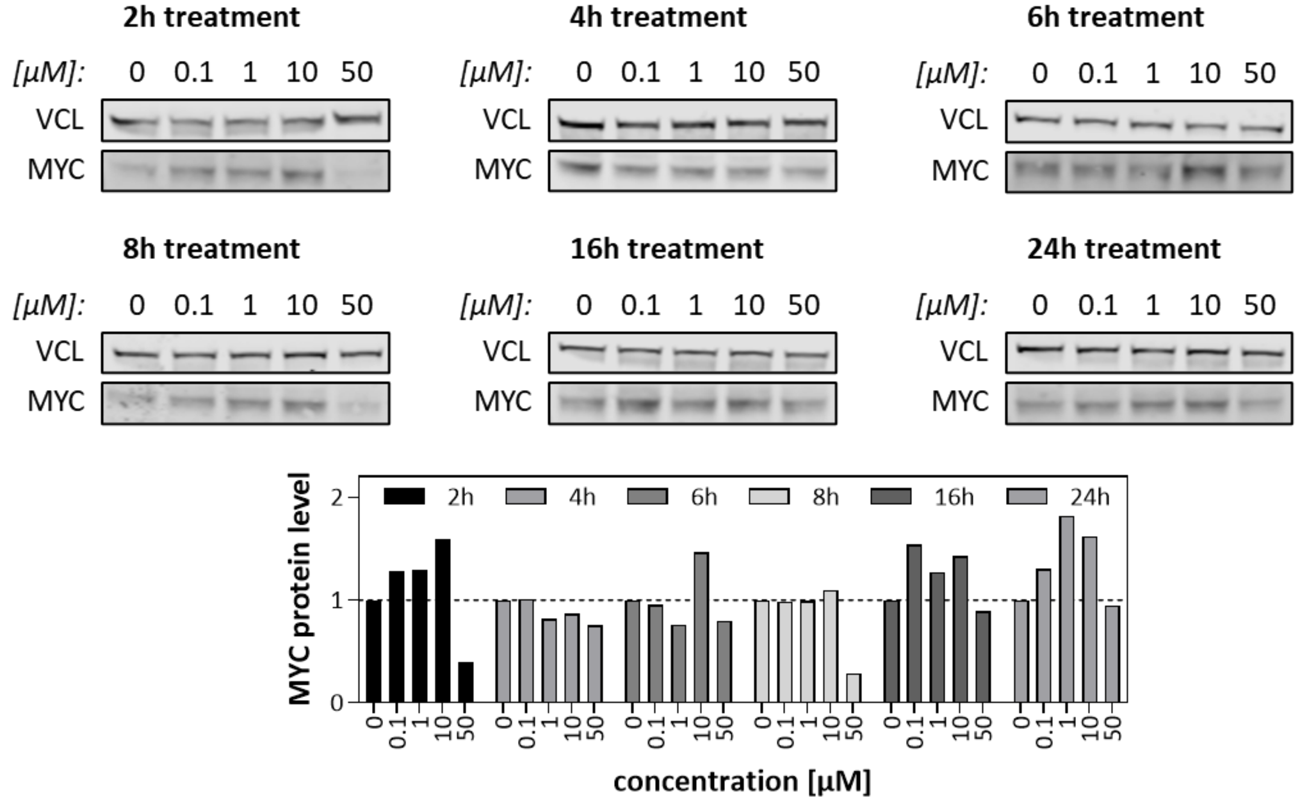

34e

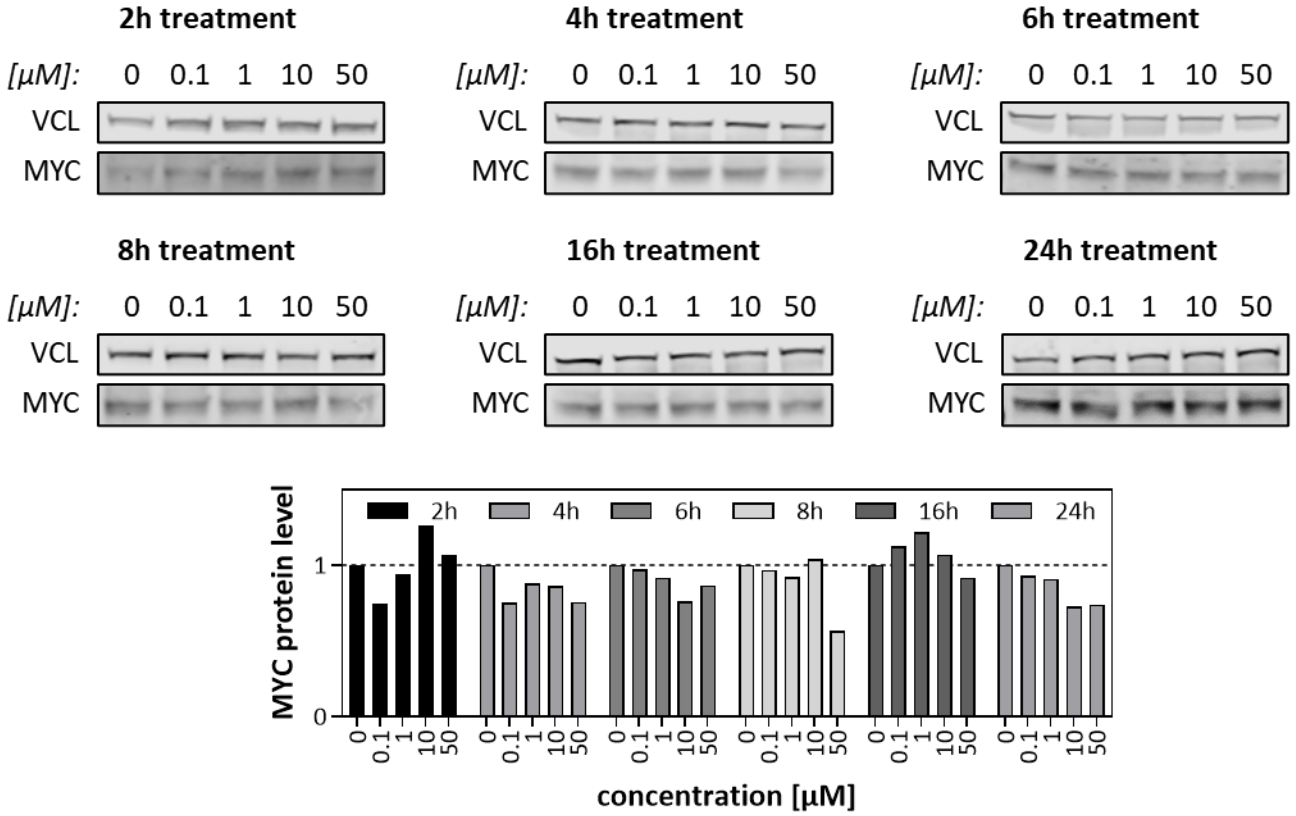

2h treatment
4h treatment
6h treatment
8h treatment
16h treatment
24h treatment
[µM]: 0 0.1 1 10 50
VCL
MYC
2h 4h 6h 8h 16h 24h
MYC protein level
1
0
0 0.1 1 10 50
concentration [µM]

Figure S5. Time course analysis of most active potential MYC degraders at 50 µM using HiBiT-MYC and LgBiT overexpression in HEK293T17 cells. n = 3

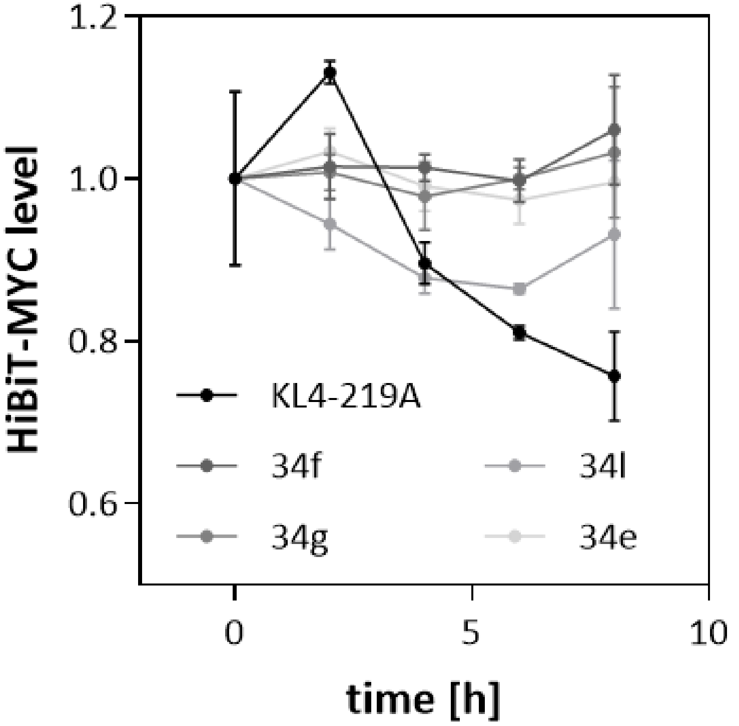

Figure S6. Non-enzymatic stability testing. Stability of PROTACs in assay medium at 37 °C.

Cmpd. ID	Class	% Stability at 37°C HPLC			
		0h	6h	12h	24h
**16a** (Abd11)	CRBN-based PROTAC	100	100	100	100
**16b** (Abd1)	CRBN-based PROTAC	100	100	100	100
**16c** (Abd9)	CRBN-based PROTAC	100	100	100	100
**16d** (Abd12)	CRBN-based PROTAC	100	100	100	100
**16e** (Abd44)	CRBN-based PROTAC	100	100	100	100
**16f** (Abd48)	CRBN-based PROTAC	100	100	98.5	94.3
**16g** (Abd8)	CRBN-based PROTAC	100	100	100	100
**21a** (Abd42)	CRBN-based PROTAC	100	97.5	94.6	87.8
**21b** (Abd25)	CRBN-based PROTAC	100	99.5	97.2	93.7
**21c** (Abd3)	CRBN-based PROTAC	100	100	98.5	90.2
**27a** (Abd41)	CRBN-based PROTAC	100	98.8	95.8	89.6
**27b** (Abd40)	CRBN-based PROTAC	100	97.3	87.3	51.1
**34f** (Abd2)	VHL-based PROTAC	100	100	100	100
**34g** (Abd5)	VHL-based PROTAC	100	100	100	100
**34i** (Abd7)	VHL-based PROTAC	100	100	100	100

Figure S7. Predicted ADME properties (in silico) using SwissADME* (https://www.swissadme.ch)

Cpd.	Mol. weigth	logP consensus	N rot. bonds	H-bonds acc.	H-bonds don.	TPSA Å^2	Lipinski violations	GI absorption
**Abd1**	723.8	3.95	15	7	4	163.01	2	low
**Abd41**	801.9	3.78	21	9	4	188.37	2	low
**Abd20**	914.1	5.19	25	9	6	236.40	3	low
**Abd21**	928.1	5.48	26	9	6	236.40	3	low
**Abd2**	928.1	5.18	26	9	6	236.40	3	low
**Abd5**	942.1	5.62	27	9	6	236.40	3	low
**Abd23**	928.1	5.24	26	9	6	236.40	3	low
**Abd24**	942.1	5.51	27	9	6	236.40	3	low
**EN4**	416.5	4.13	11	4	2	76.66	0	high
**KL4-219A**	370.5	2.85	4	4	0	68.83	0	high

*SwissADME: a free web tool to evaluate pharmacokinetics, drug-likeness and medicinal chemistry friendliness of small molecules. Sci. Rep. (2017) 7:42717.

Figure S8. ^{1}H-NMR, ^{13}C-NMR, HPLC chromatogram, and HRMS spectra

16a (Abd11)

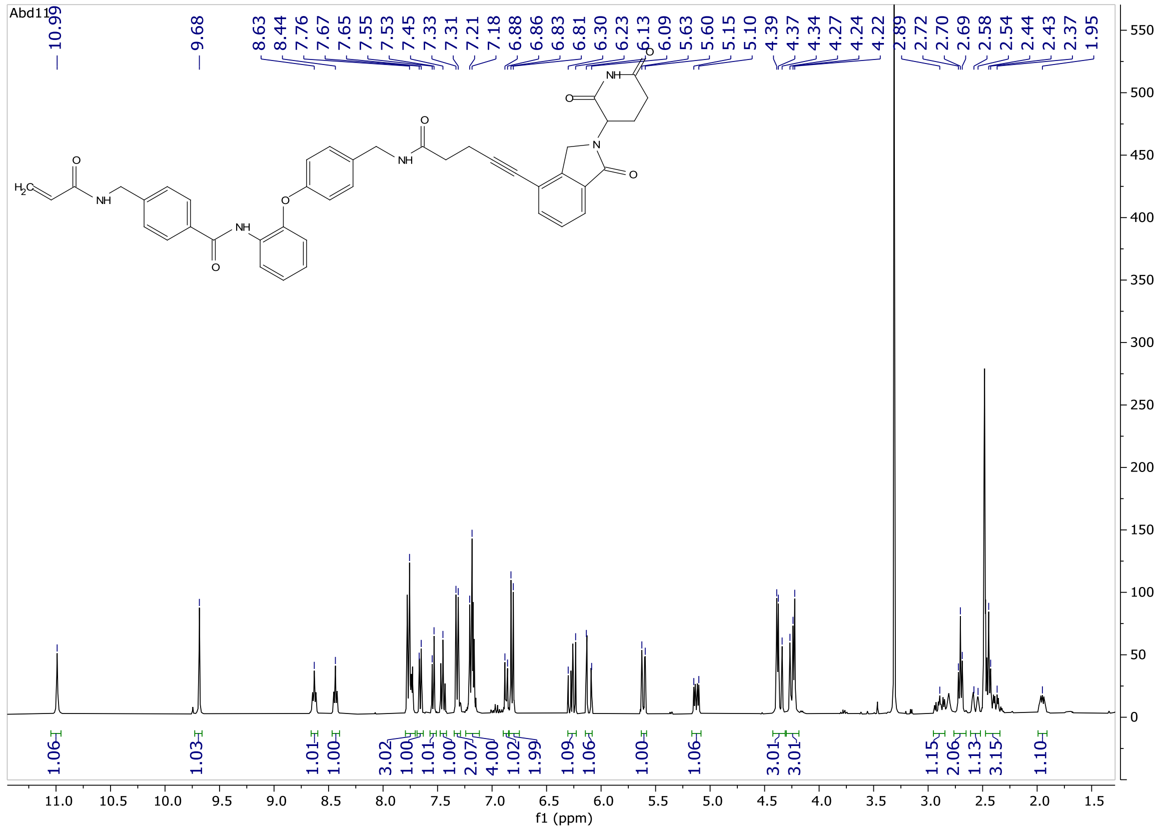

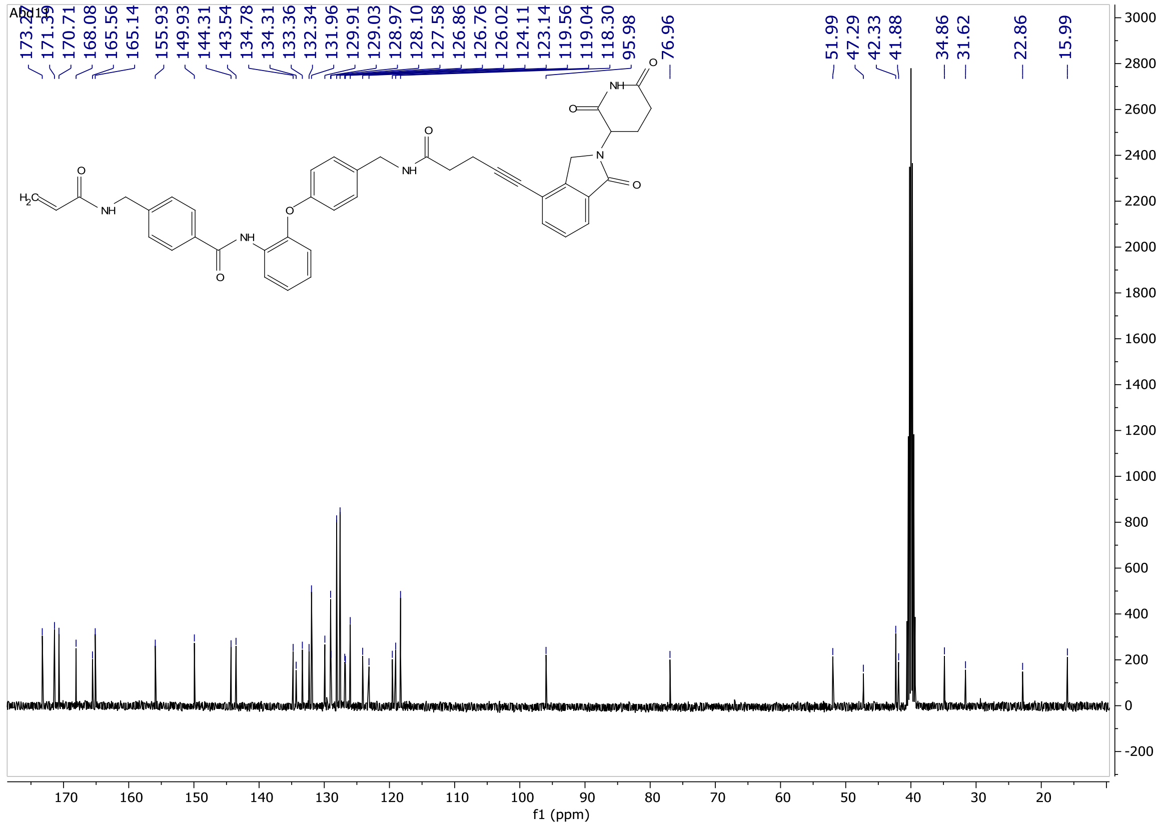

16a (Abd11)

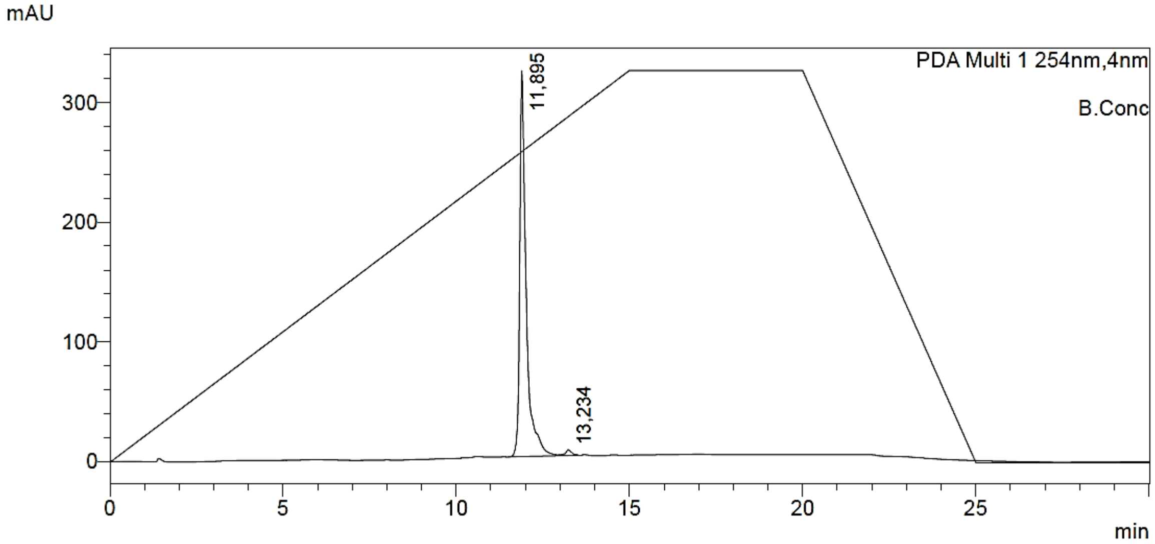

PDA Ch1 254nm

Peak#	Ret. Time	Area	Height	Area%
1	11,895	4190261	322551	98,756
2	13,234	52801	4604	1,244
Total		4243063	327154	100,000

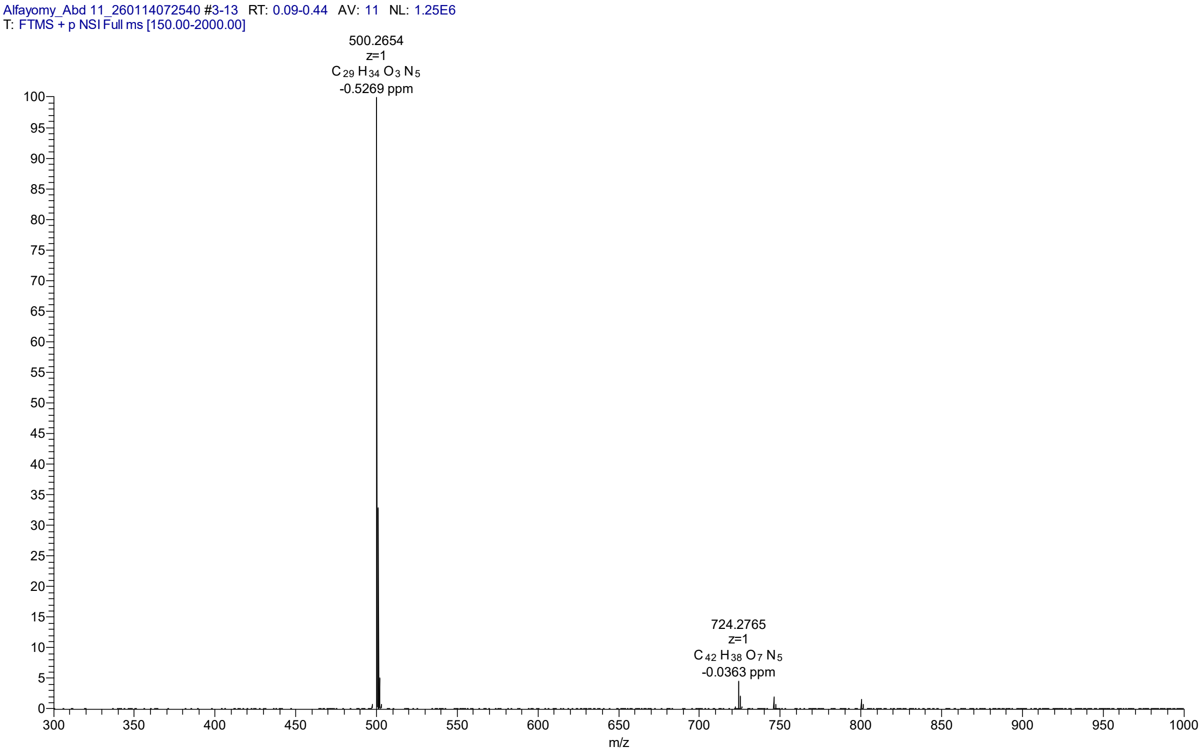

16b (Abd1)

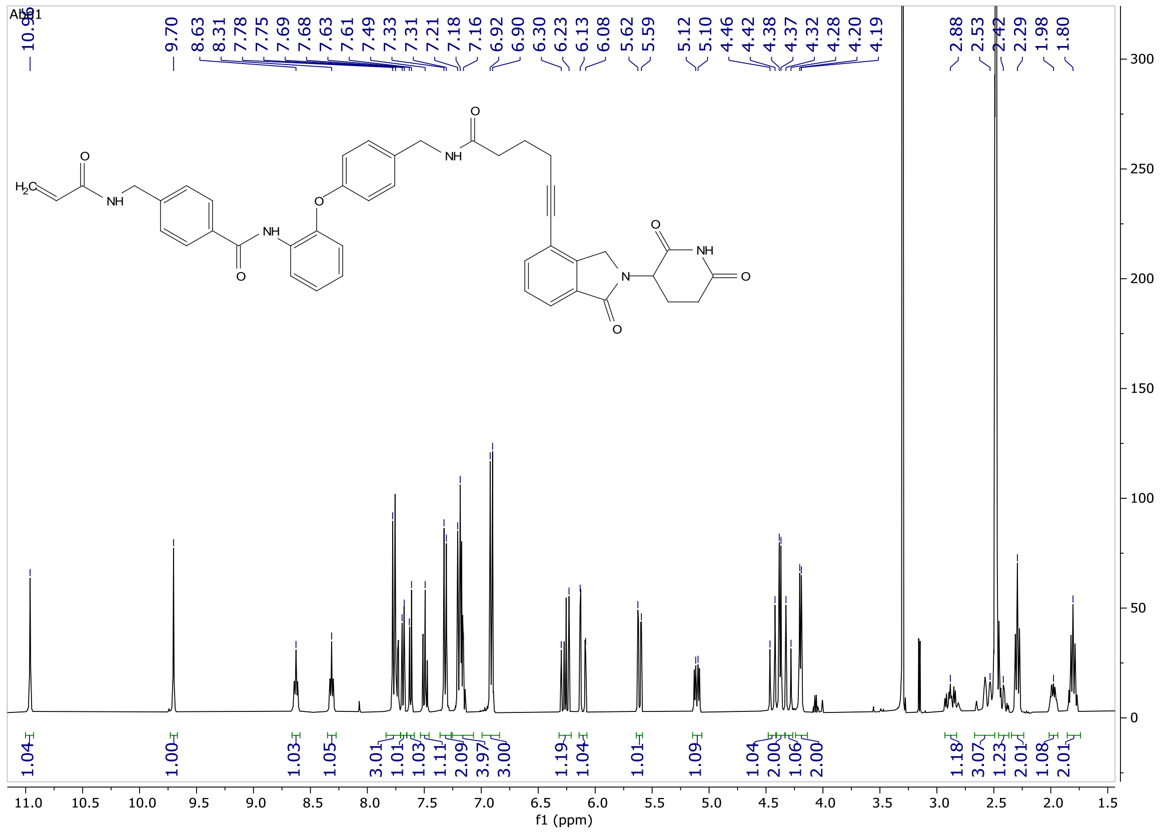

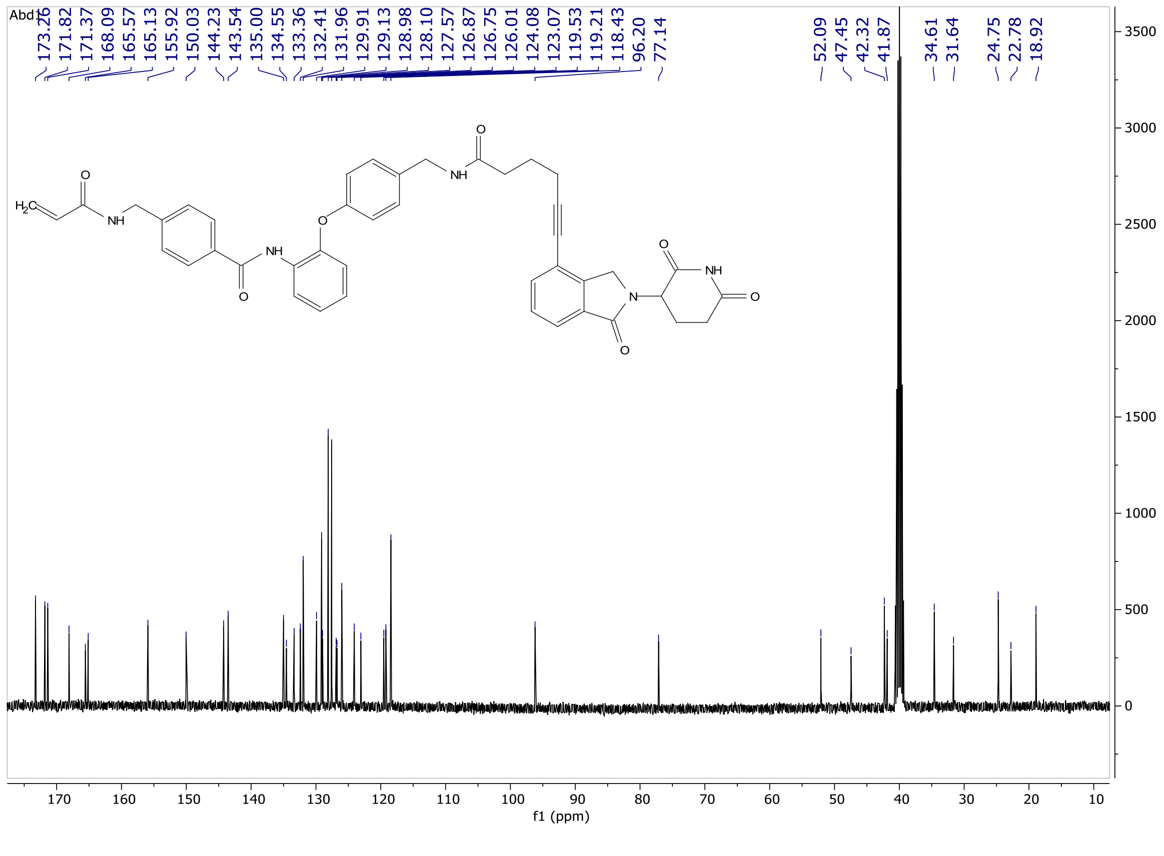

16b (Abd1)

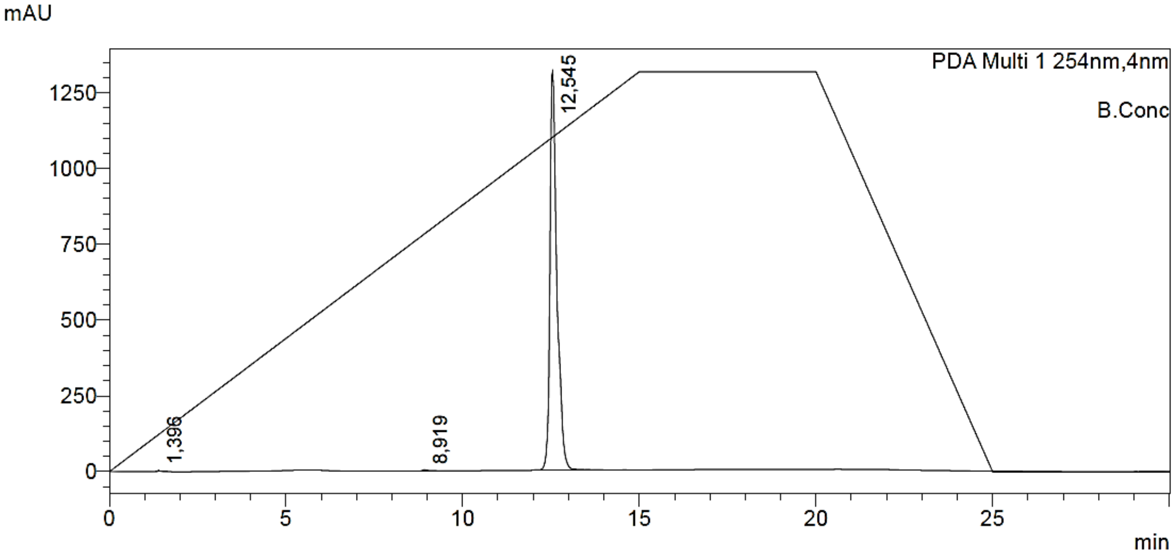

PDA Ch1 254nm

Peak#	Ret. Time	Area	Height	Area%
1	1,396	22843	5167	0,126
2	8,919	38349	3683	0,211
3	12,545	18121388	1313307	99,663
Total		18182580	1322156	100,000

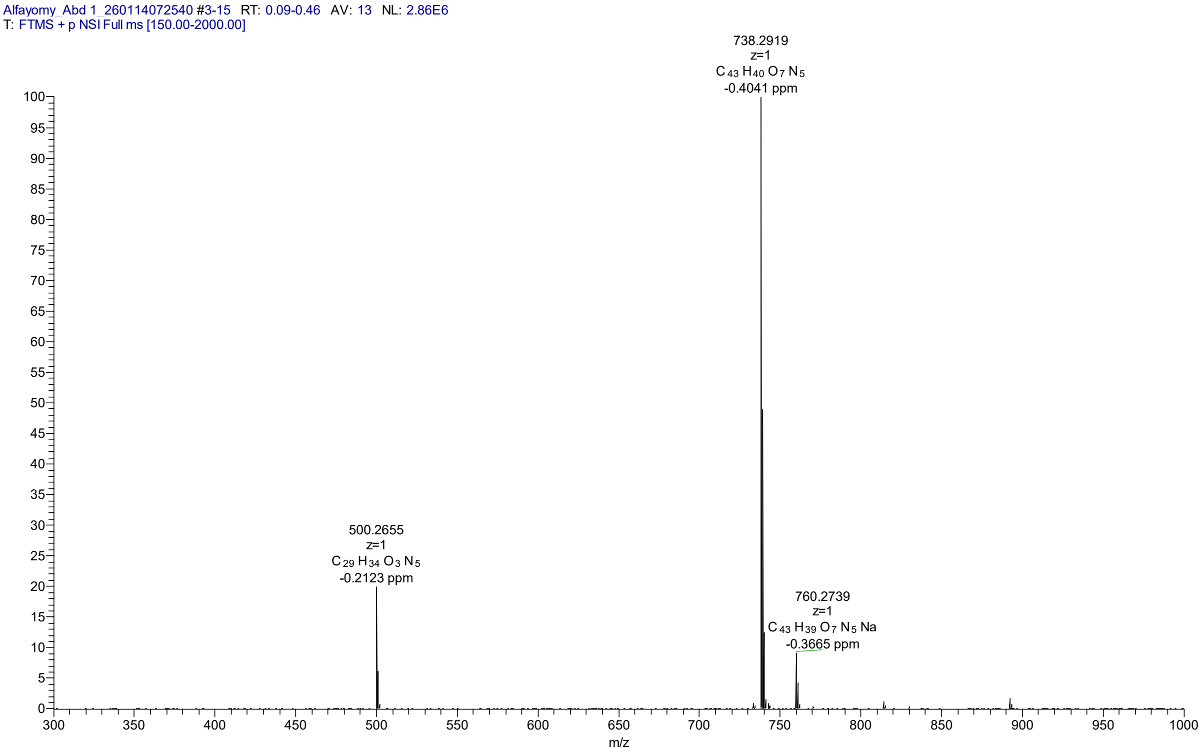

16c (Abd9)

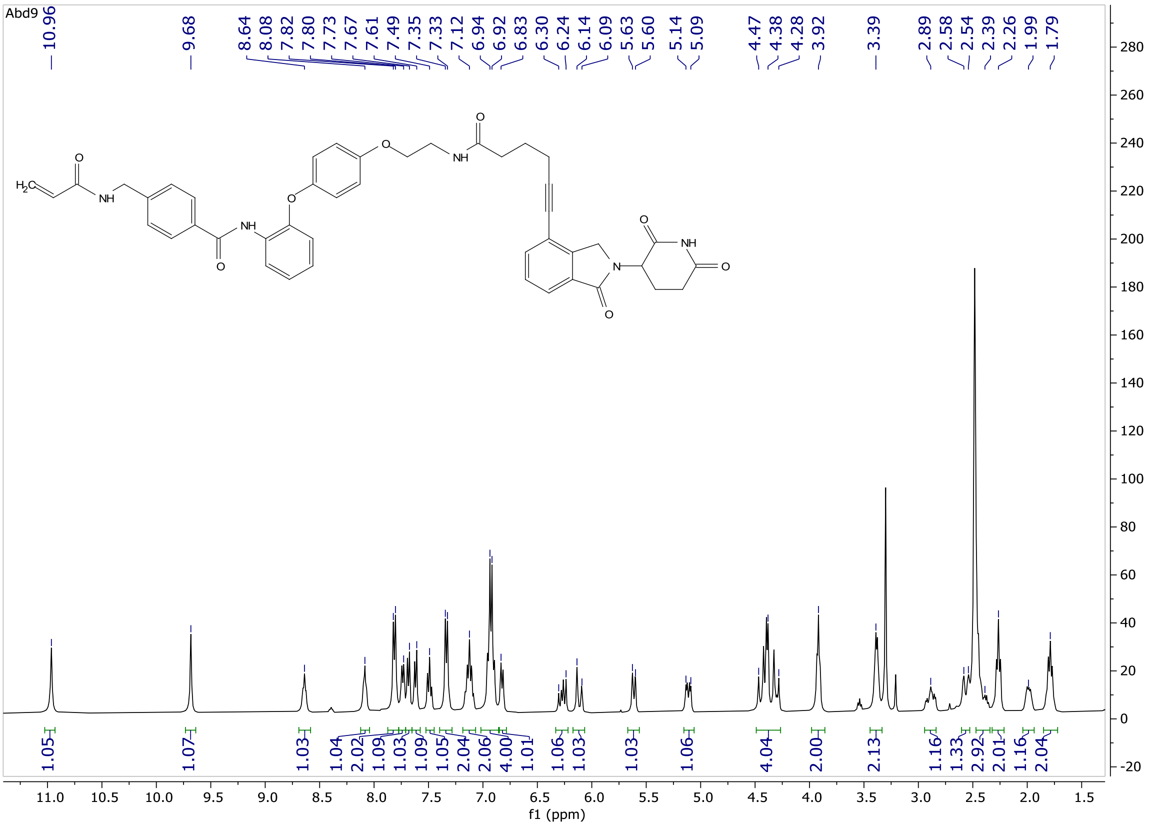

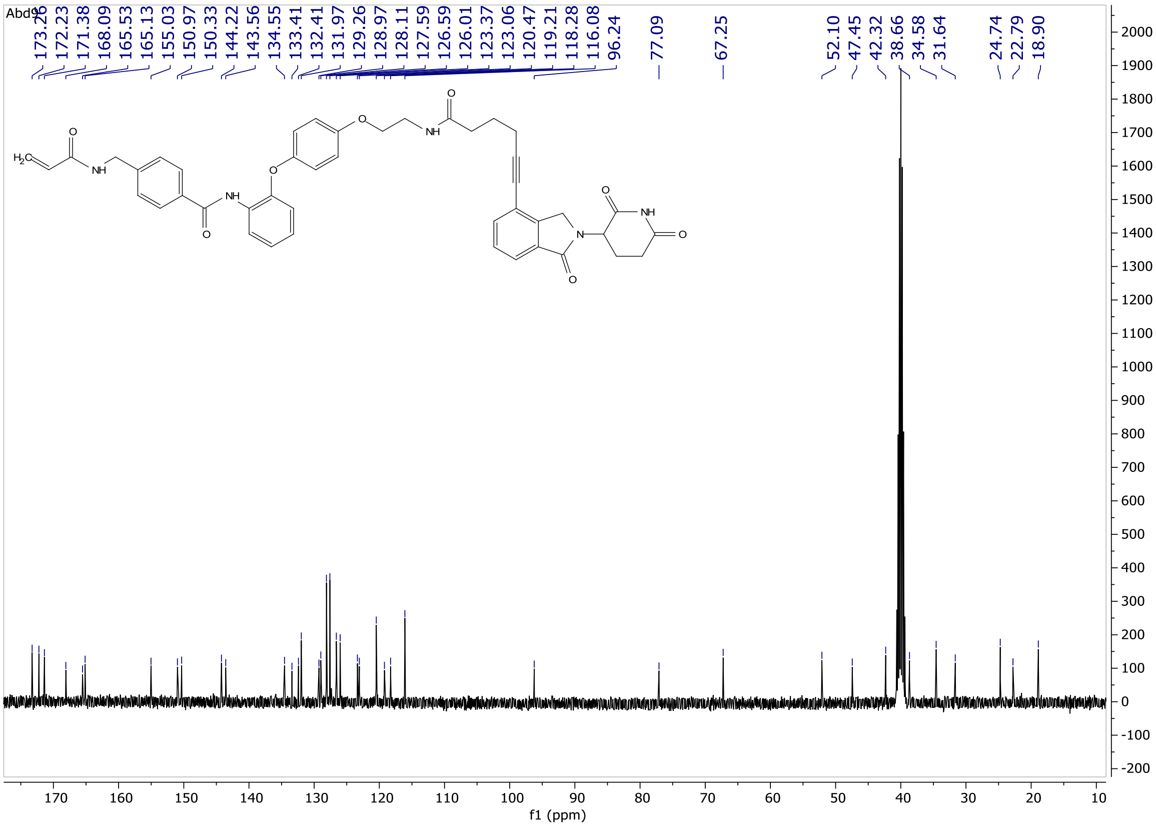

16c (Abd9)

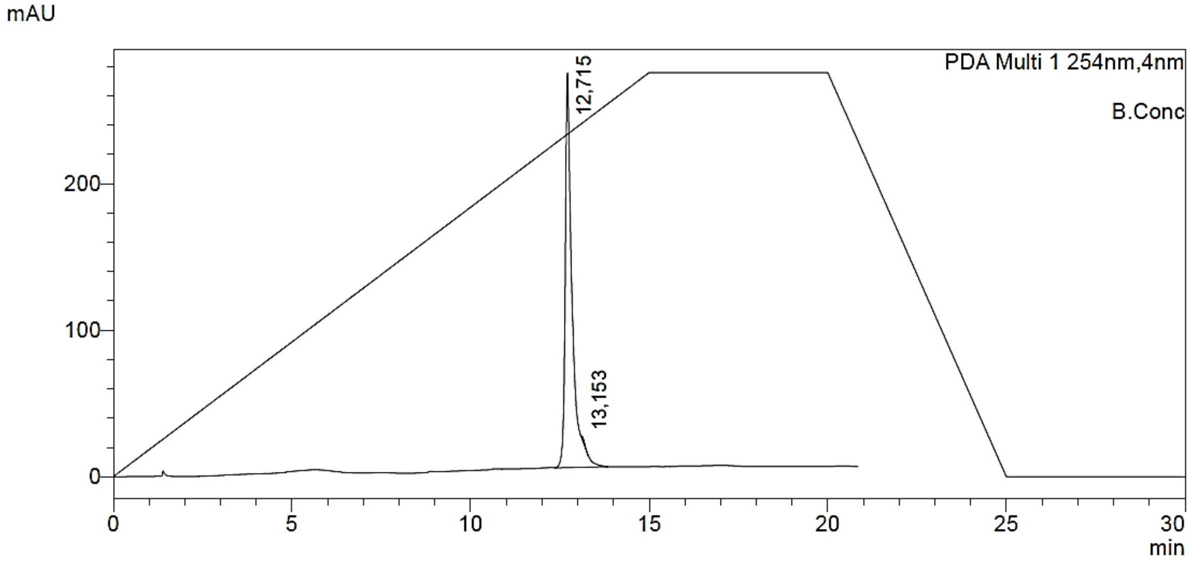

PDA Ch1 254nm

Peak#	Ret. Time	Area	Height	Area%
1	12,715	3616980	269353	99,780
2	13,153	7986	2393	0,220
Total		3624966	271746	100,000

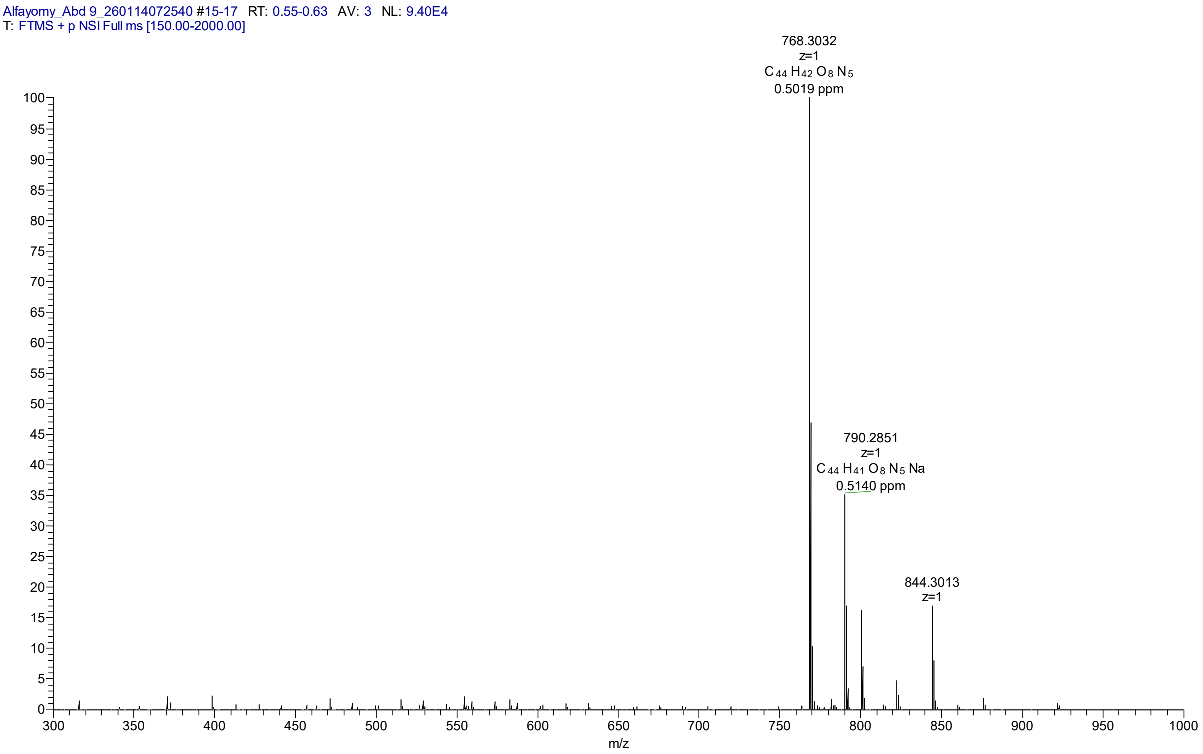

16d (Abd12)

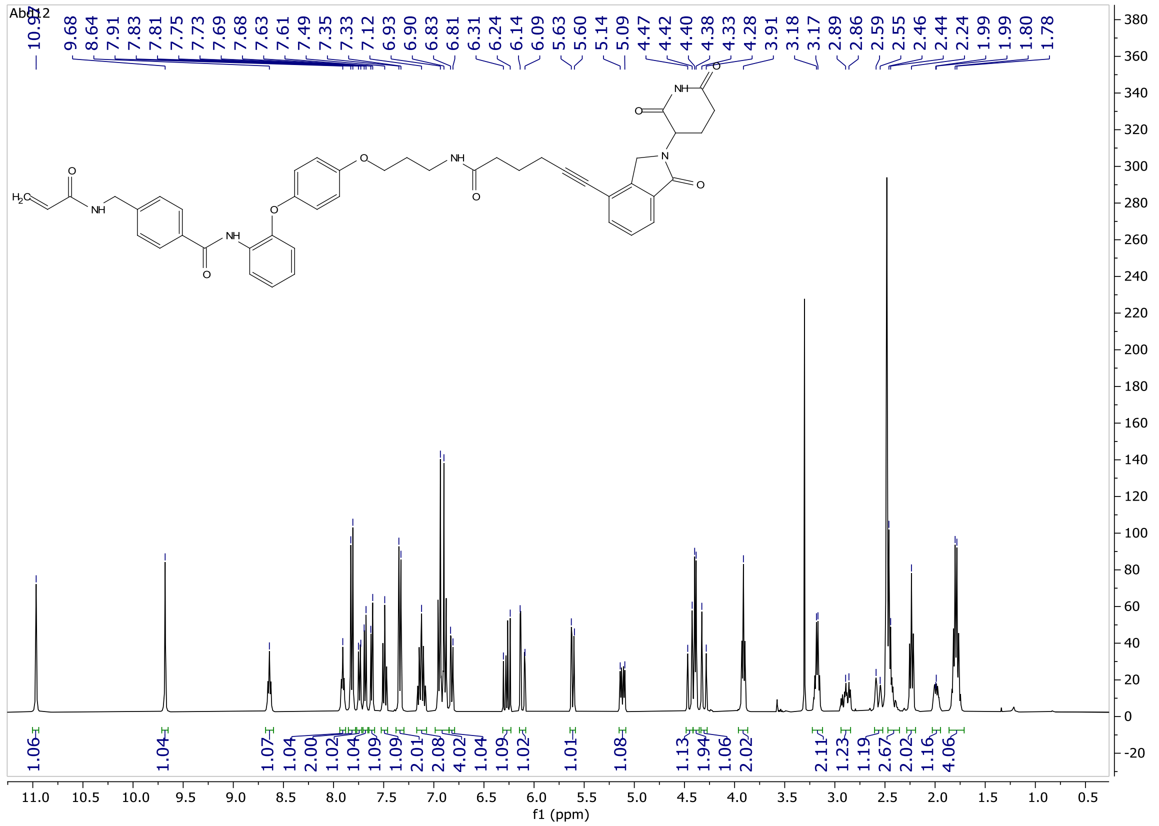

Abd12
10.97
9.68
8.64
7.91
7.83
7.81
7.75
7.73
7.69
7.68
7.63
7.61
7.49
7.35
7.33
7.12
6.93
6.90
6.83
6.81
6.31
6.24
6.14
6.09
5.63
5.60
5.14
5.09
4.47
4.42
4.40
4.38
4.33
4.28
3.91
3.18
3.17
2.89
2.86
2.59
2.55
2.46
2.44
2.24
1.99
1.99
1.80
1.78
1.06
1.04
1.07
1.04
2.00
1.02
1.04
1.09
1.09
2.01
2.08
4.02
1.04
1.09
1.02
1.01
1.08
1.13
1.94
1.06
2.02
2.11
1.23
1.19
2.67
2.02
1.16
4.06
f1 (ppm)

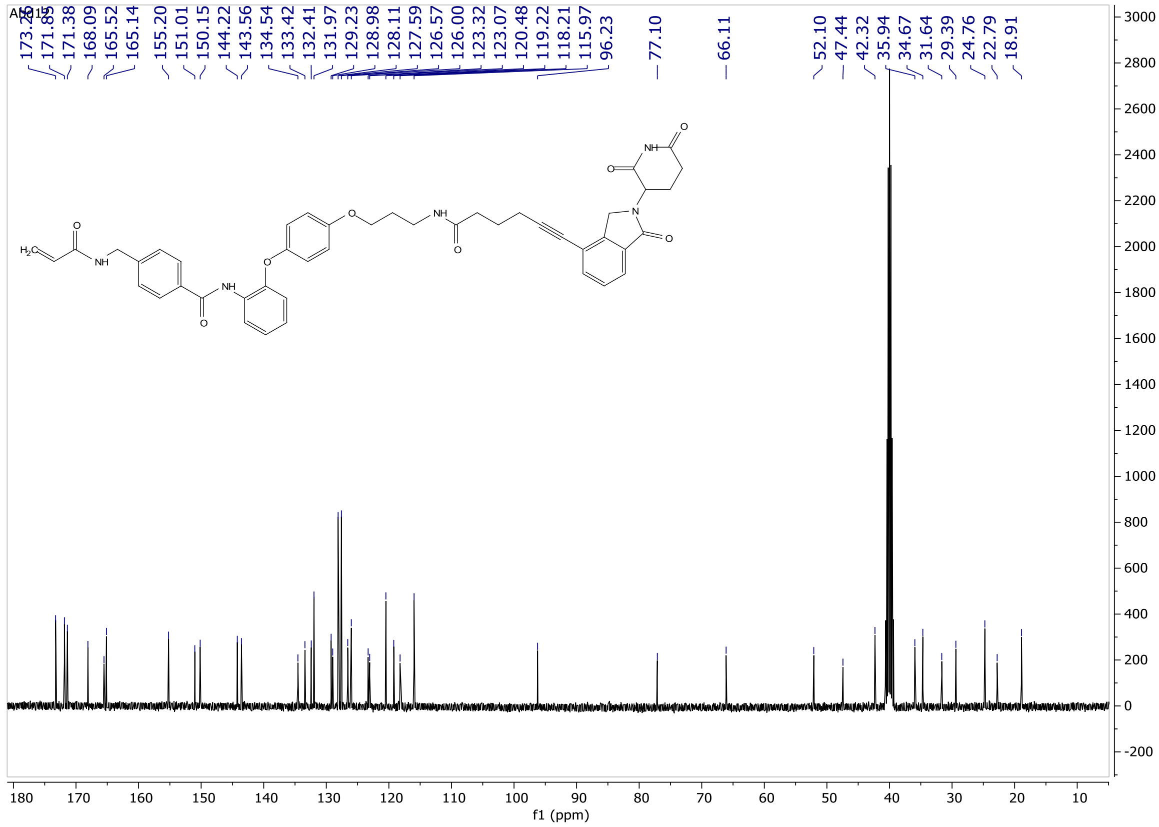

Abd12
173.20
171.85
171.38
168.09
165.52
165.14
155.20
151.01
150.15
144.22
143.56
134.54
133.42
132.41
131.97
129.23
128.98
128.11
127.59
126.57
126.00
123.32
123.07
120.48
119.22
118.21
115.97
96.23
77.10
66.11
52.10
47.44
42.32
35.94
34.67
31.64
29.39
24.76
22.79
18.91
f1 (ppm)

16d (Abd12)

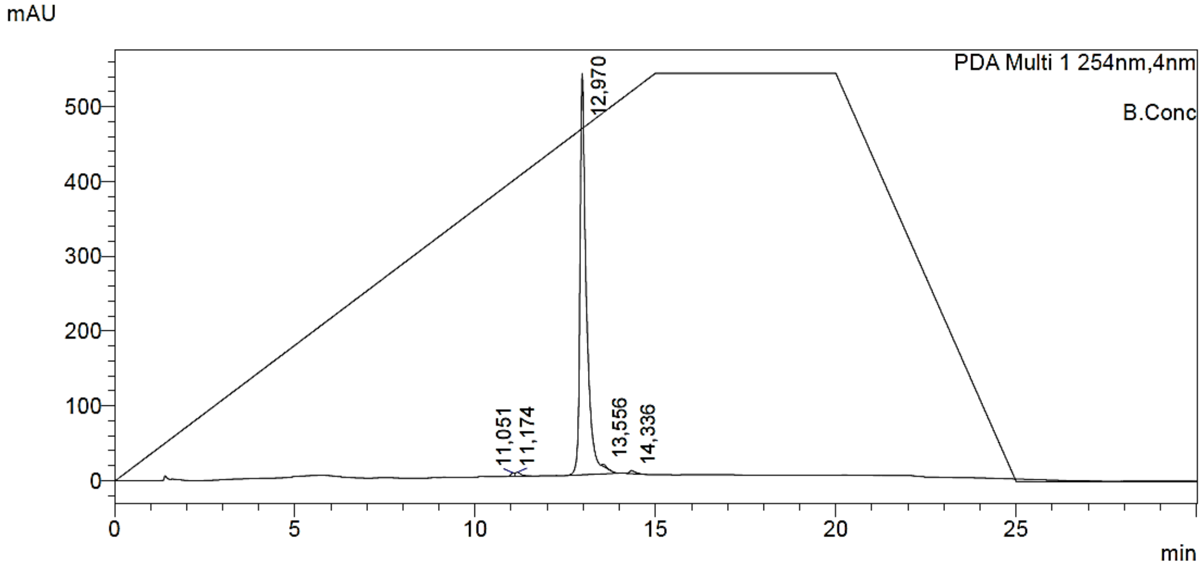

PDA Ch1 254nm

Peak#	Ret. Time	Area	Height	Area%
1	11,051	38525	4954	0,561
2	11,174	57483	5545	0,837
3	12,970	6702716	537319	97,607
4	13,556	20023	2830	0,292
5	14,336	48274	4131	0,703
Total		6867021	554778	100,000

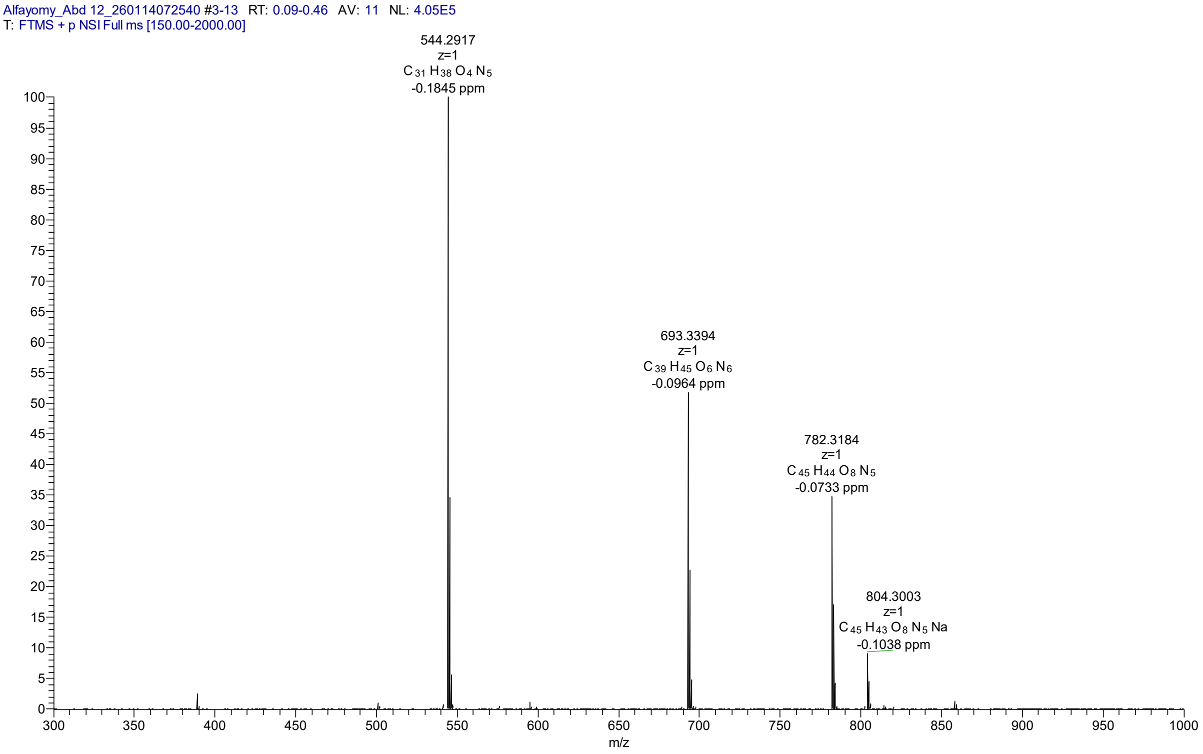

16e (Abd44)

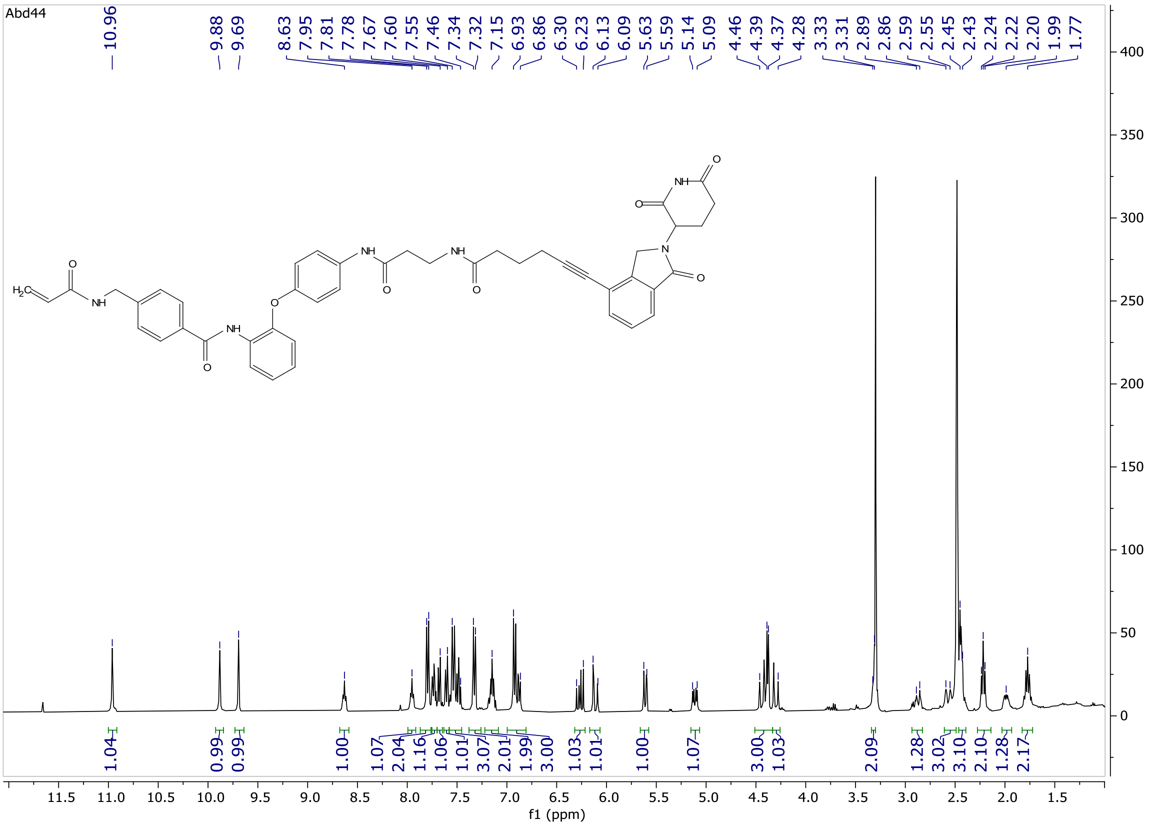

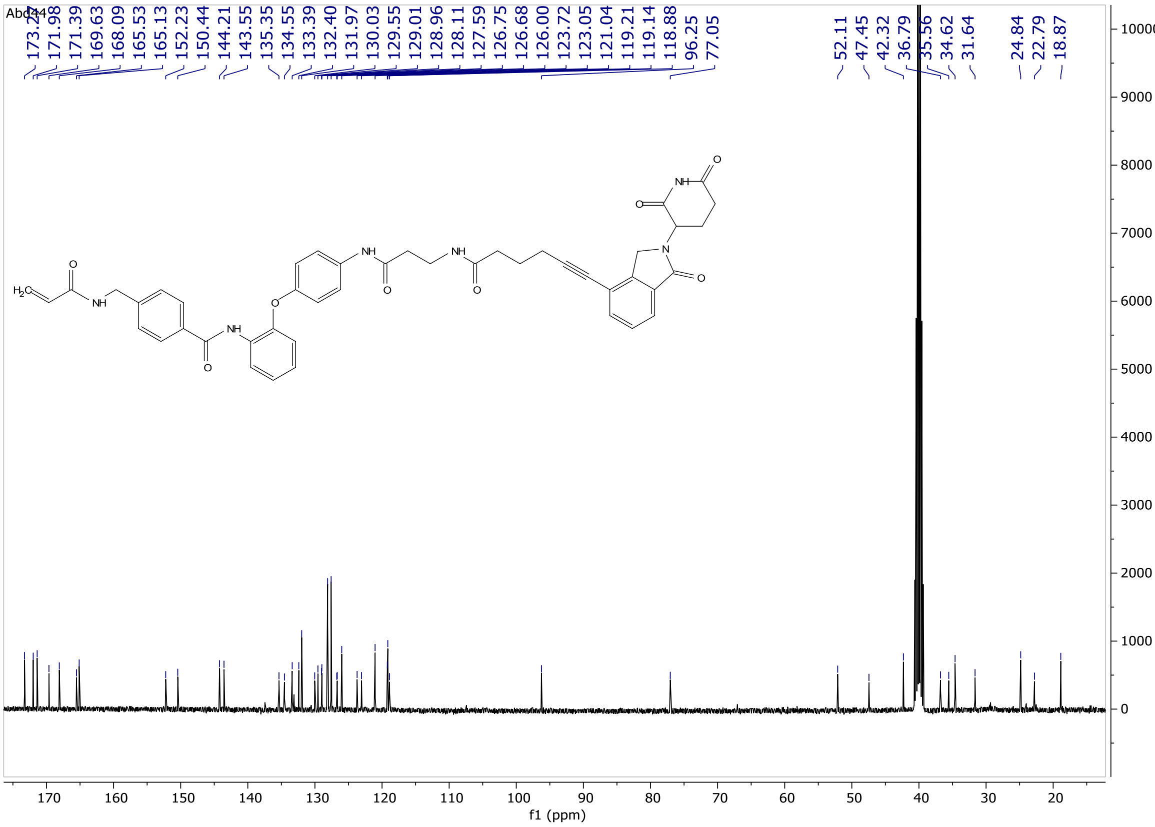

16e (Abd44)

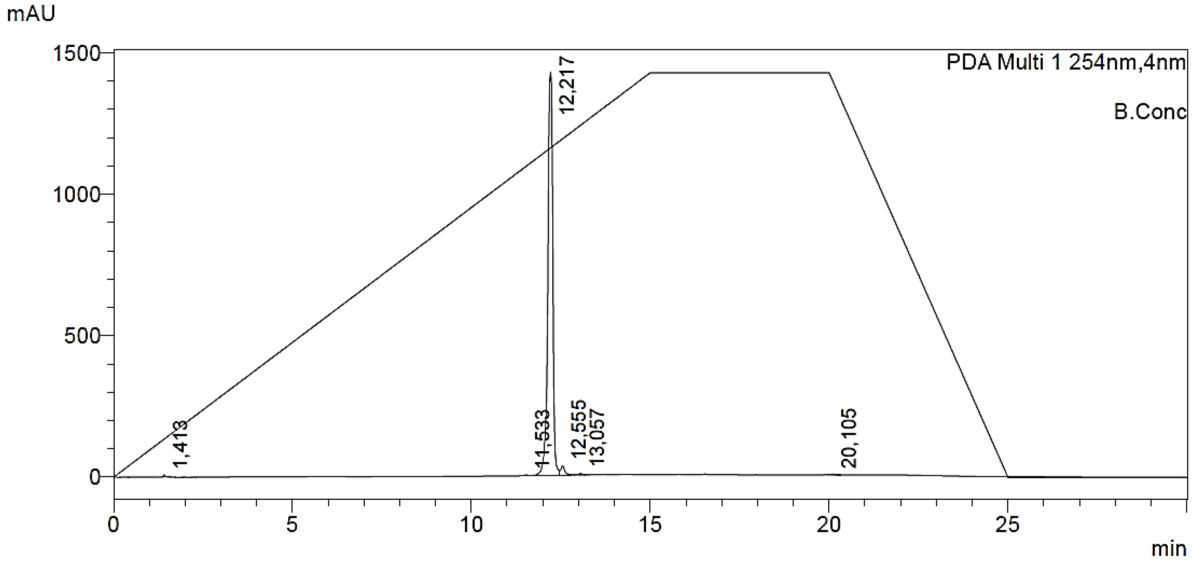

PDA Ch1 254nm

Peak#	Ret. Time	Area	Height	Area%
1	1,413	30560	7327	0,217
2	11,533	16365	2628	0,116
3	12,217	13704051	1422465	97,134
4	12,555	278353	32964	1,973
5	13,057	52532	5081	0,372
6	20,105	26593	2884	0,188
Total		14108453	1473349	100,000

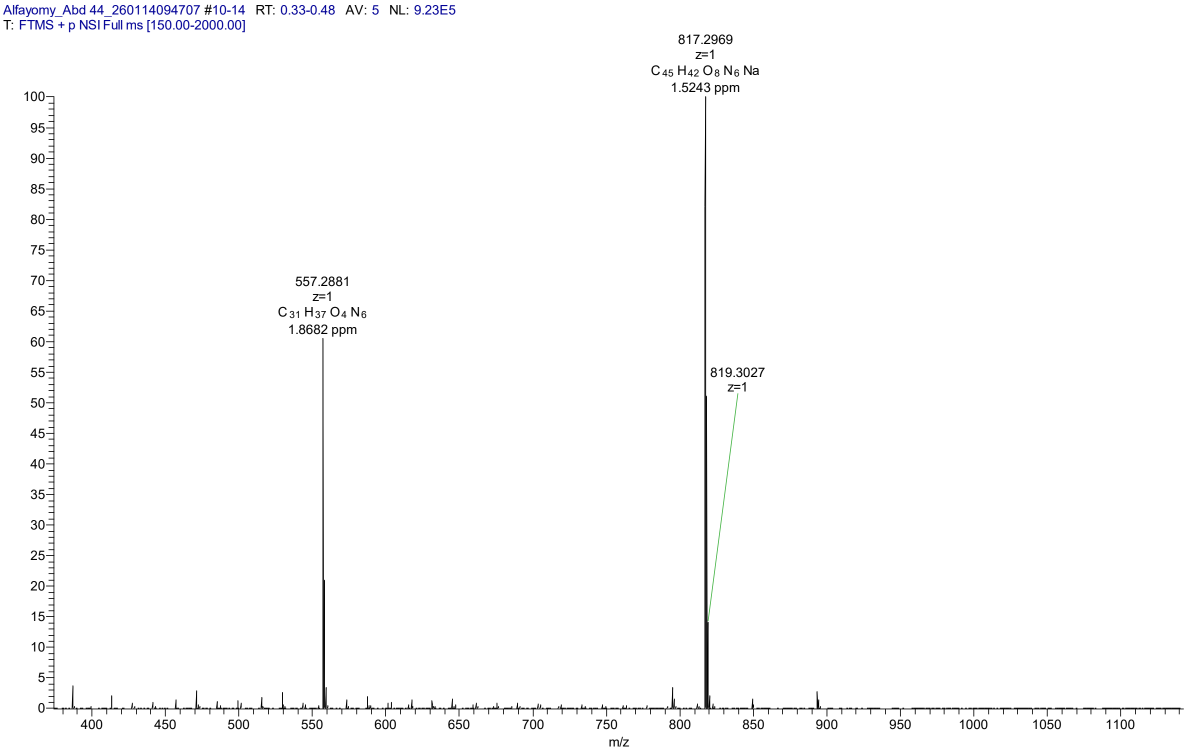

16f (Abd48)

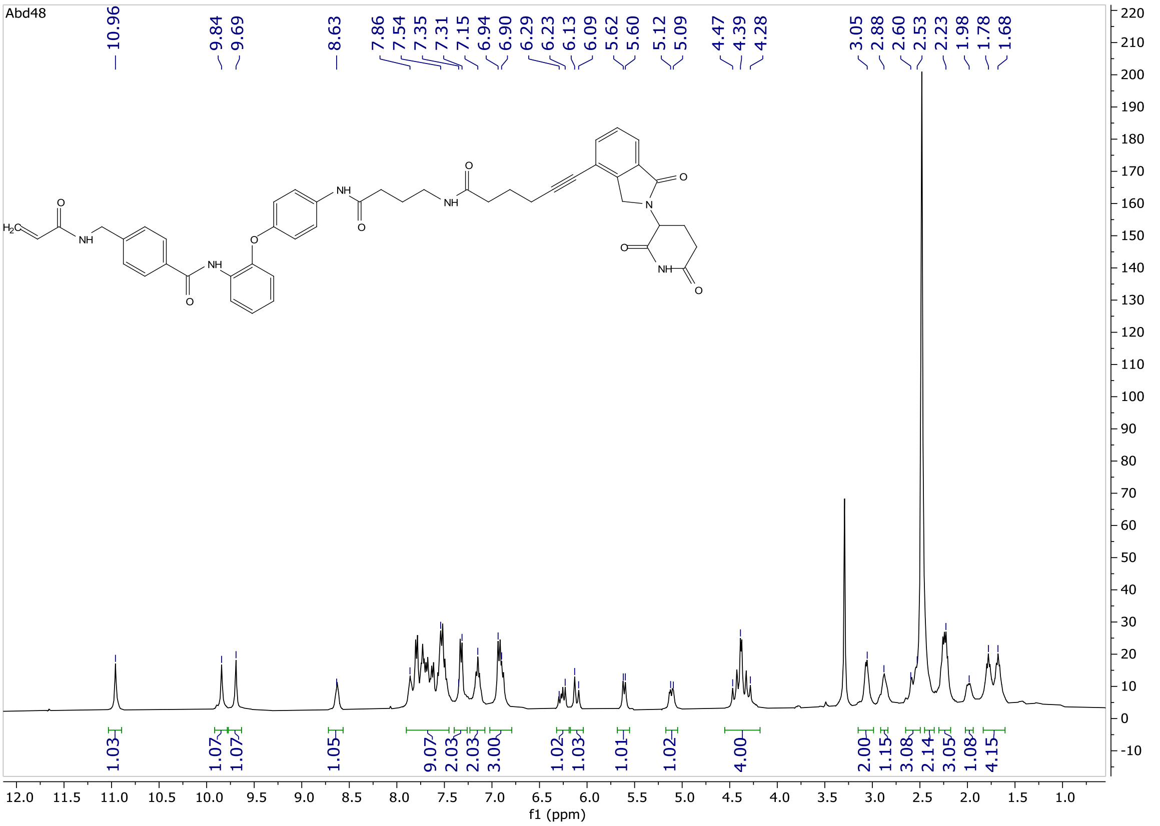
Abd48
10.96
9.84
9.69
8.63
7.86
7.54
7.35
7.31
7.15
6.94
6.90
6.29
6.23
6.13
6.09
5.62
5.60
5.12
5.09
4.47
4.39
4.28
3.05
2.88
2.60
2.53
2.23
1.98
1.78
1.68
1.03
1.07
1.07
1.05
9.07
2.03
2.03
3.00
1.02
1.03
1.01
1.02
4.00
2.00
1.15
3.08
2.14
3.05
1.08
4.15
f1 (ppm)

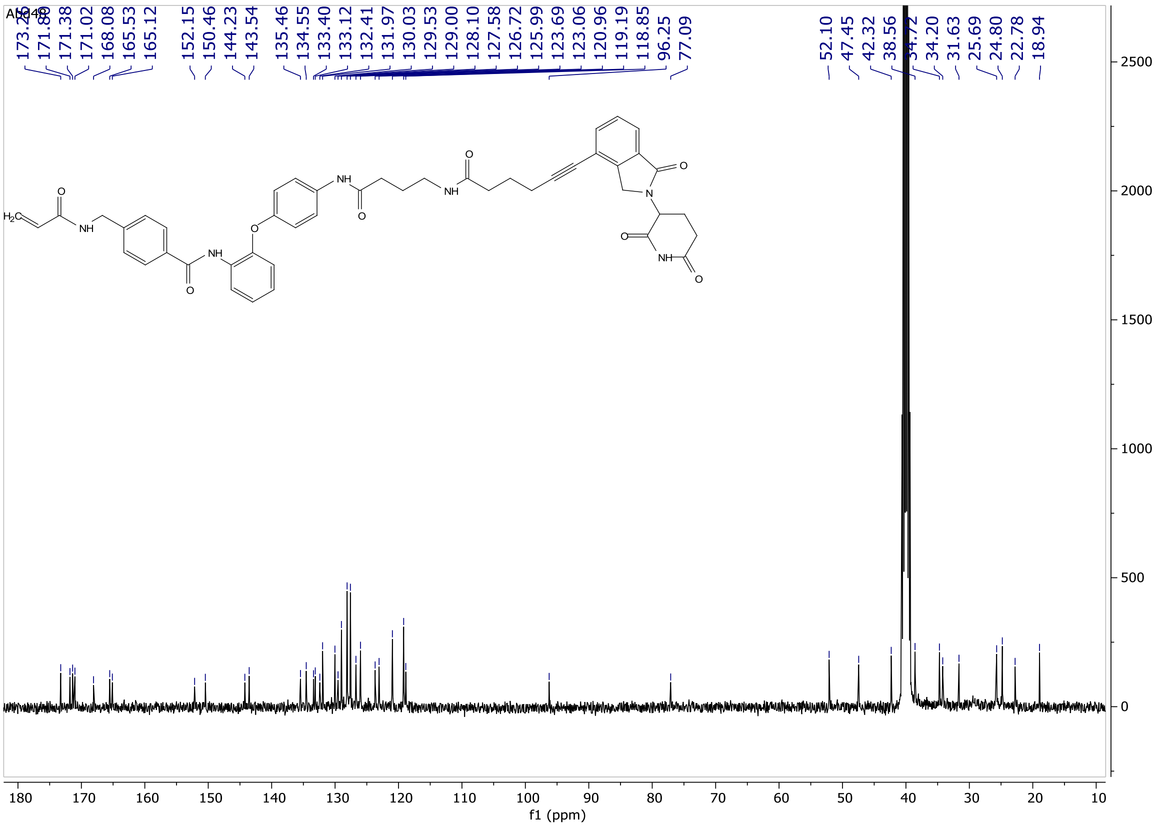
Abd48
173.26
171.86
171.38
171.02
168.08
165.53
165.12
152.15
150.46
144.23
143.54
135.46
134.55
133.40
133.12
132.41
131.97
130.03
129.53
129.00
128.10
127.58
126.72
125.99
123.69
123.06
120.96
119.19
118.85
96.25
77.09
52.10
47.45
42.32
38.56
34.72
34.20
31.63
25.69
24.80
22.78
18.94
f1 (ppm)

16f (Abd48)

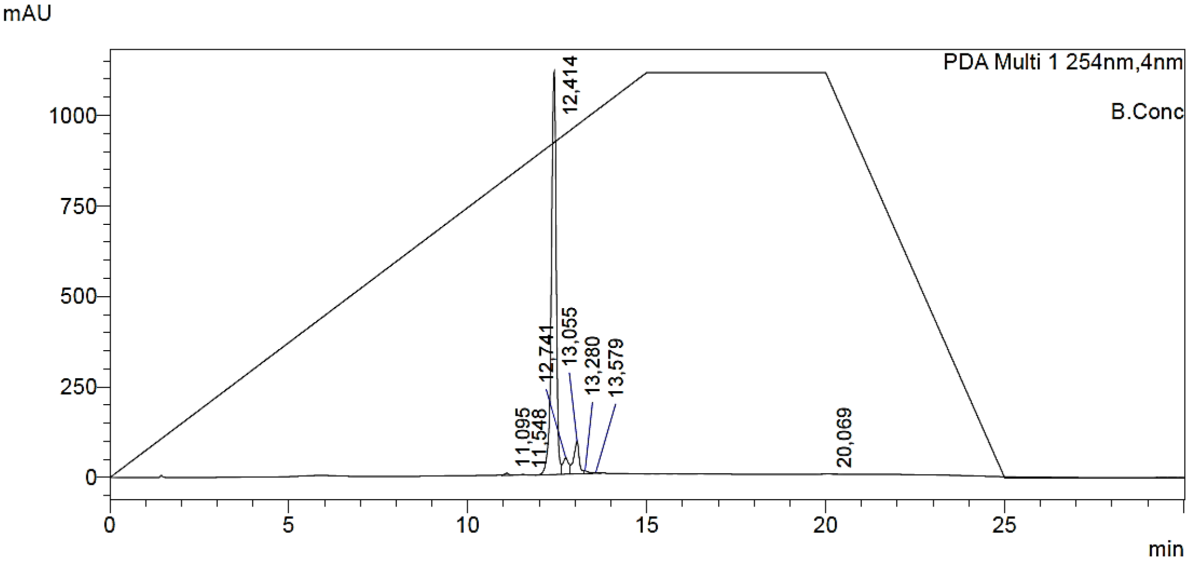

PDA Ch1 254nm

Peak#	Ret. Time	Area	Height	Area%
1	11,095	48151	5895	0,398
2	11,548	21705	2536	0,179
3	12,414	10482865	1108885	86,548
4	12,741	509060	44883	4,203
5	13,055	941516	89456	7,773
6	13,280	71153	7798	0,587
7	13,579	20047	2063	0,166
8	20,069	17701	1465	0,146
Total		12112197	1262981	100,000

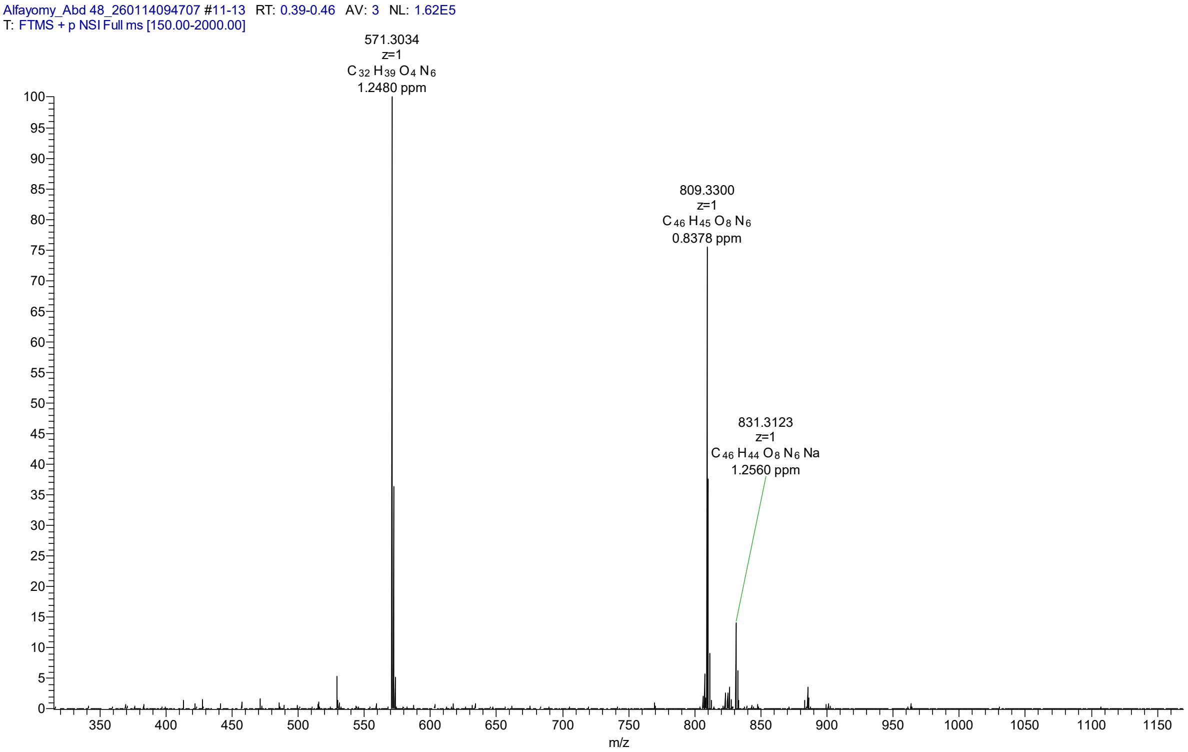

16g (Abd8)

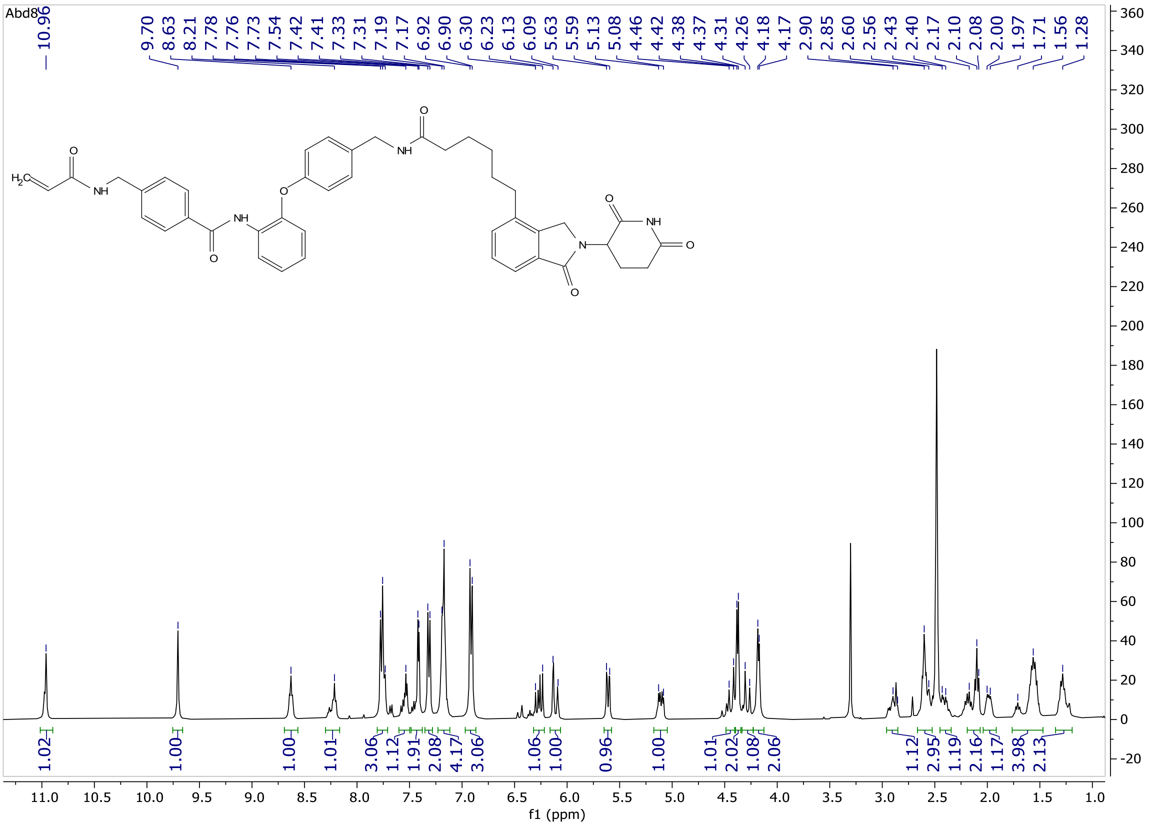
Abd8
10.96
9.70
8.63
8.21
7.78
7.76
7.73
7.54
7.42
7.41
7.33
7.31
7.19
7.17
6.92
6.90
6.30
6.23
6.13
6.09
5.63
5.59
5.13
5.08
4.46
4.42
4.38
4.37
4.31
4.26
4.18
4.17
2.90
2.85
2.60
2.56
2.43
2.40
2.17
2.10
2.08
2.00
1.97
1.71
1.56
1.28
NH
O
H2C
f1 (ppm)

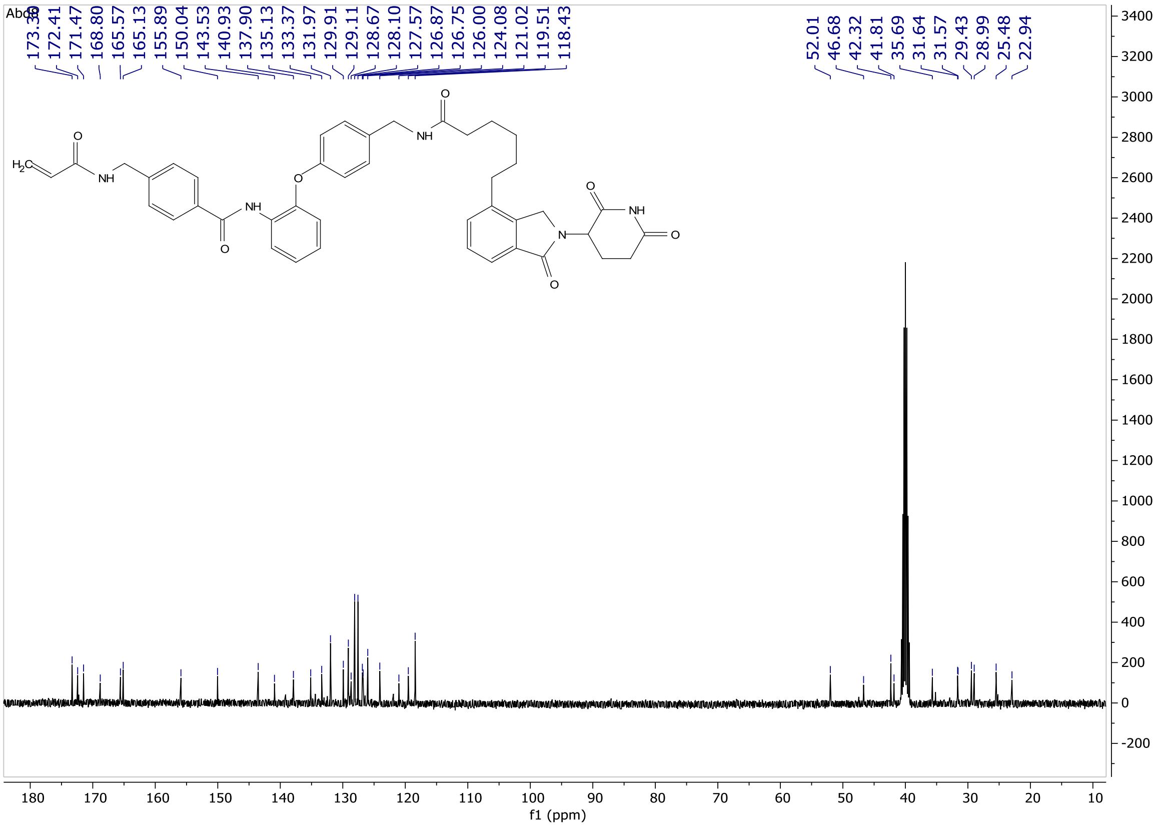
173.30
172.41
171.47
168.80
165.57
165.13
155.89
150.04
143.53
140.93
137.90
135.13
133.37
131.97
129.91
129.11
128.67
128.10
127.57
126.87
126.75
126.00
124.08
121.02
119.51
118.43
52.01
46.68
42.32
41.81
35.69
31.64
31.57
29.43
28.99
25.48
22.94
f1 (ppm)

16g (Abd8)

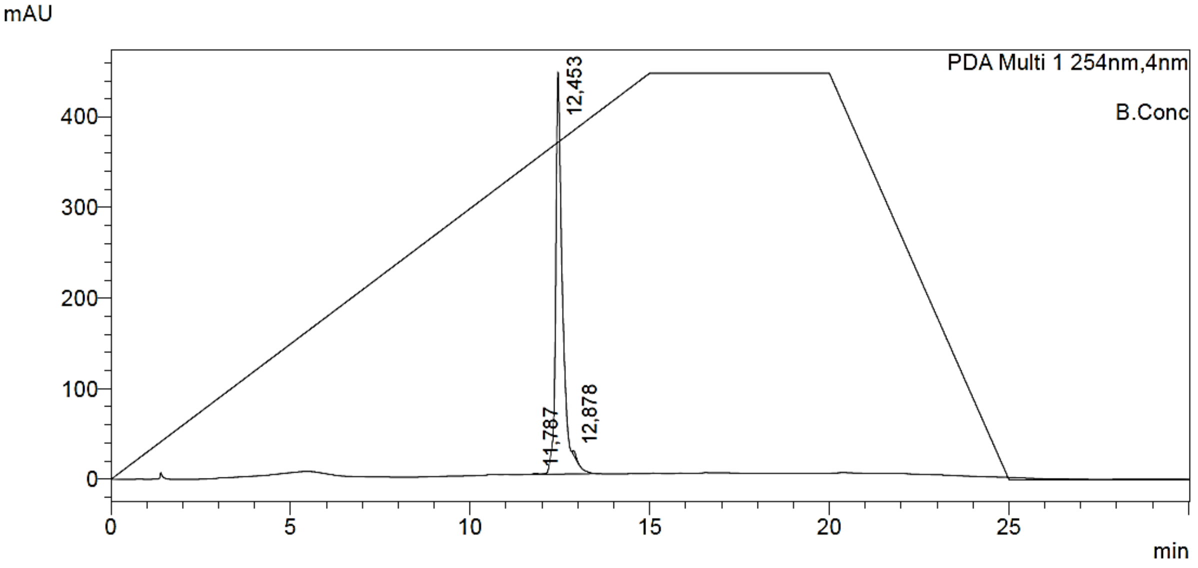

PDA Ch1 254nm

Peak#	Ret. Time	Area	Height	Area%
1	11,787	15456	1064	0,258
2	12,453	5959562	442908	99,301
3	12,878	26504	4169	0,442
Total		6001522	448141	100,000

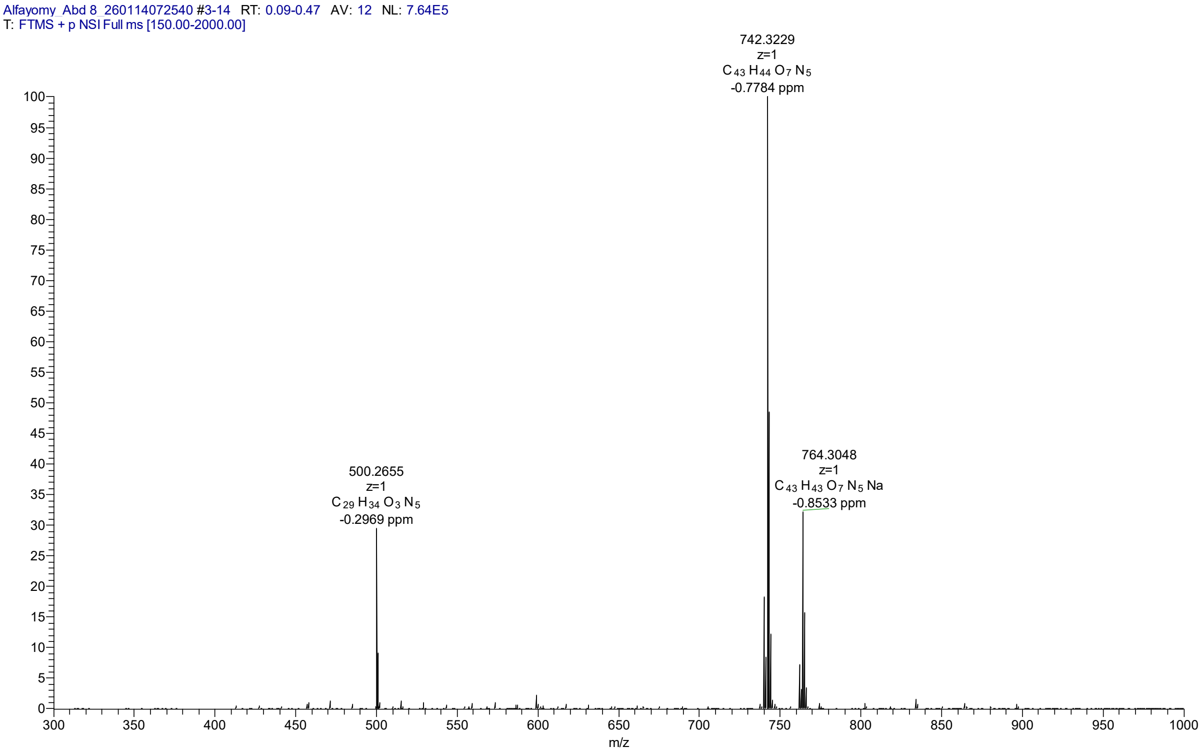

21a (Abd42)

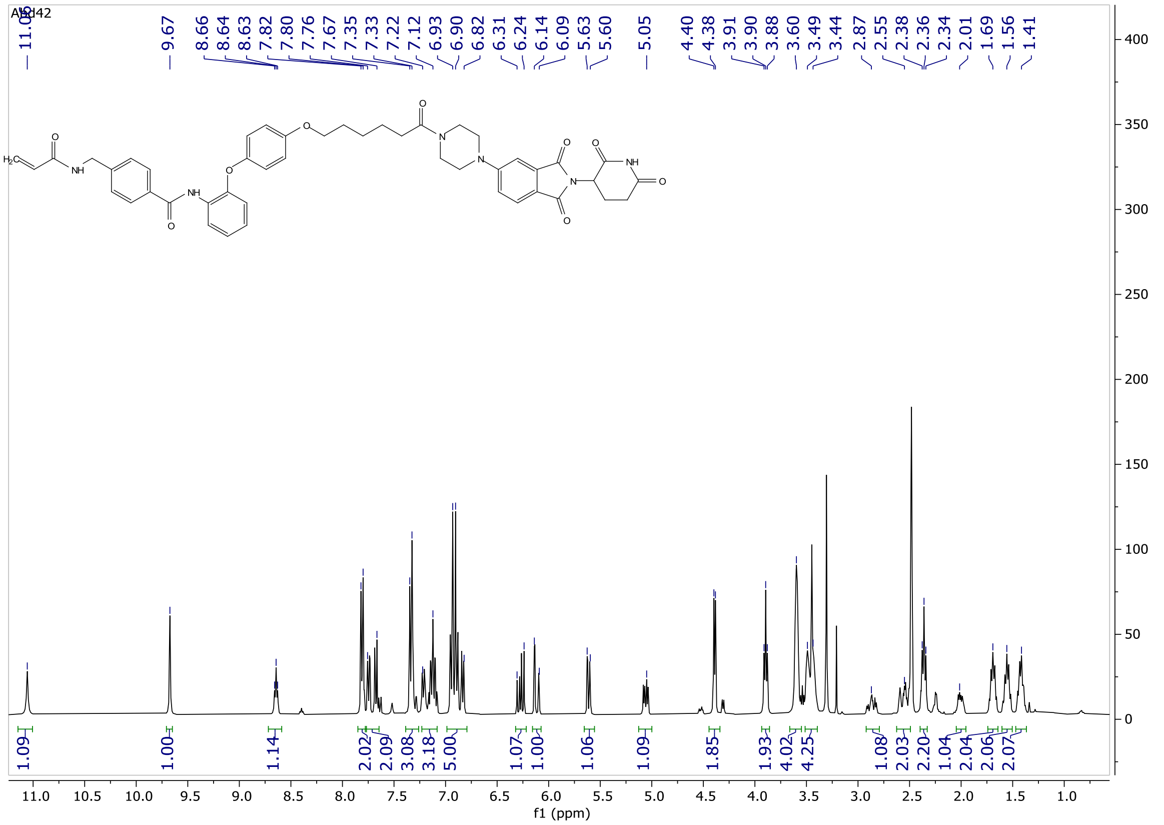

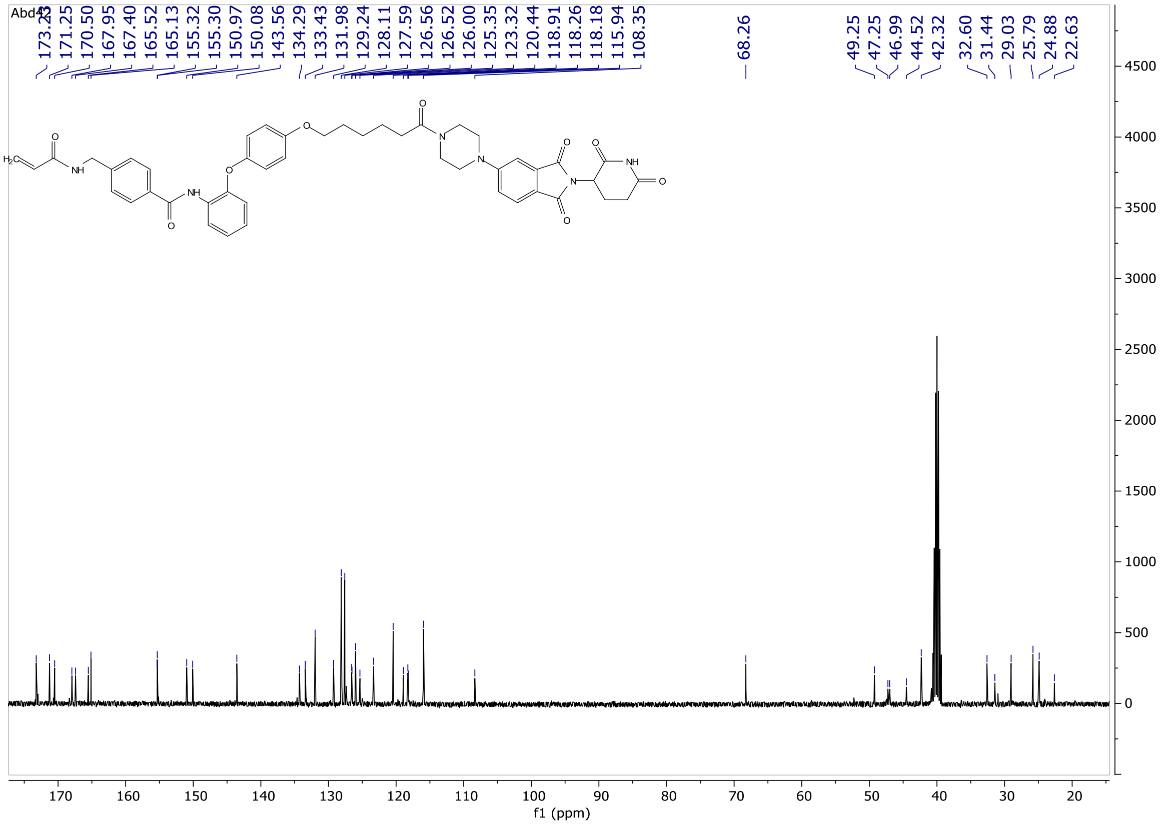

21a (Abd42)

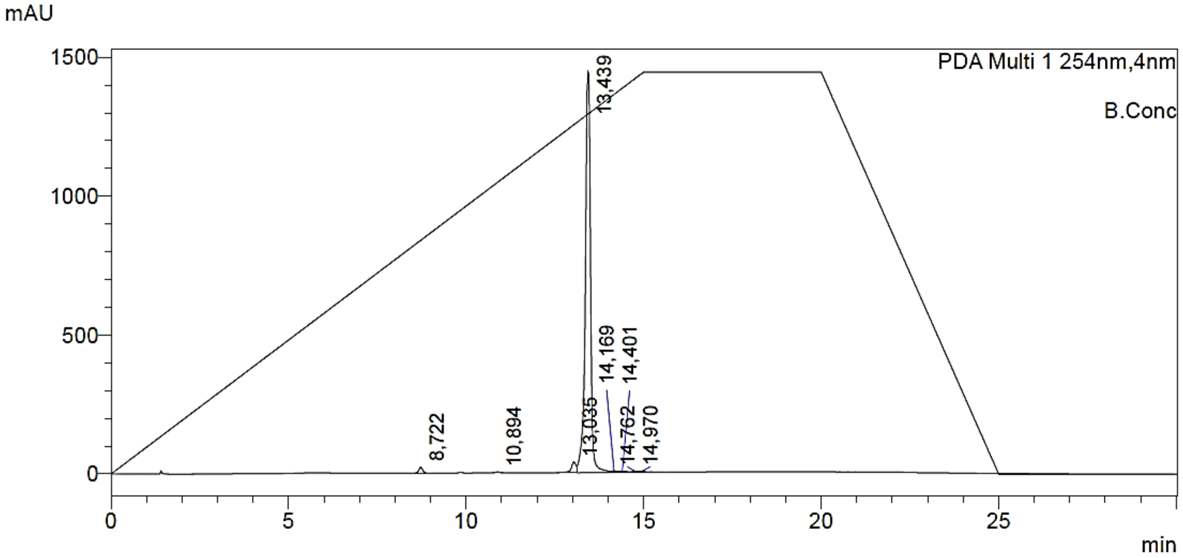

PDA Ch1 254nm

Peak#	Ret. Time	Area	Height	Area%
1	8,722	154020	21124	0,989
2	10,894	16126	2792	0,103
3	13,035	359998	38715	2,311
4	13,439	14979586	1441540	96,141
5	14,169	12387	2087	0,080
6	14,401	7925	1235	0,051
7	14,762	21468	2612	0,138
8	14,970	29361	3702	0,188
Total		15580870	1513806	100,000

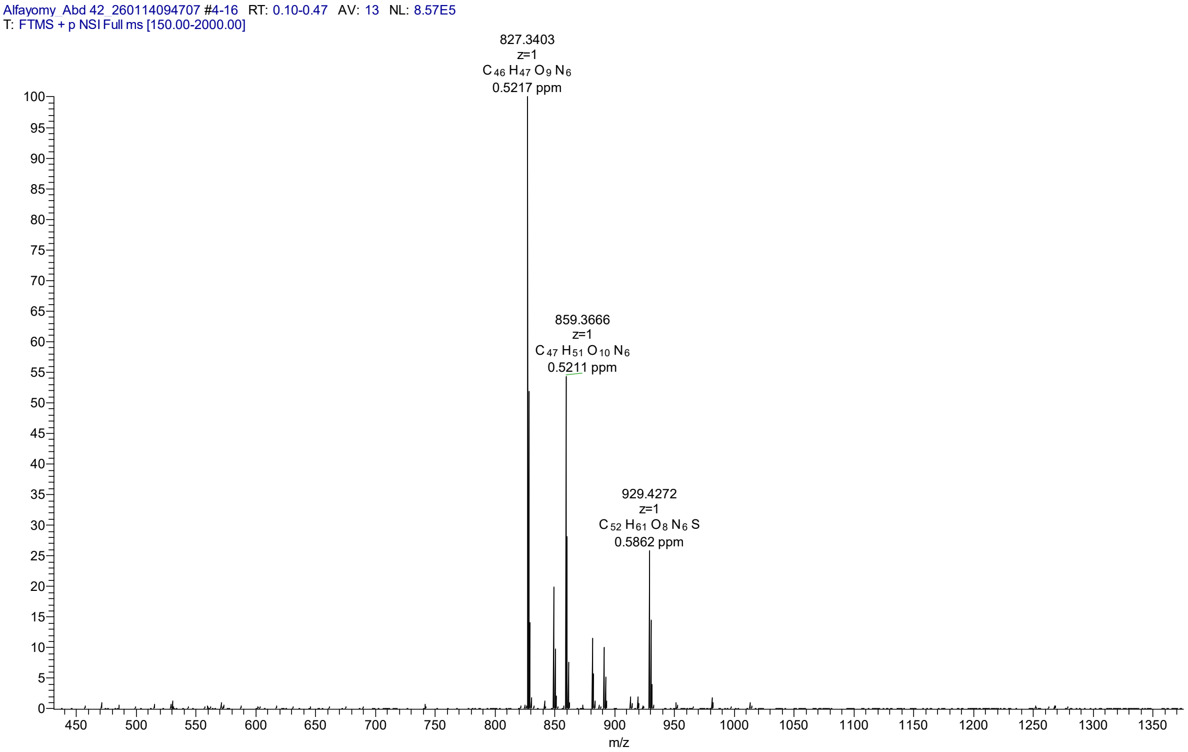

21b (Abd25)

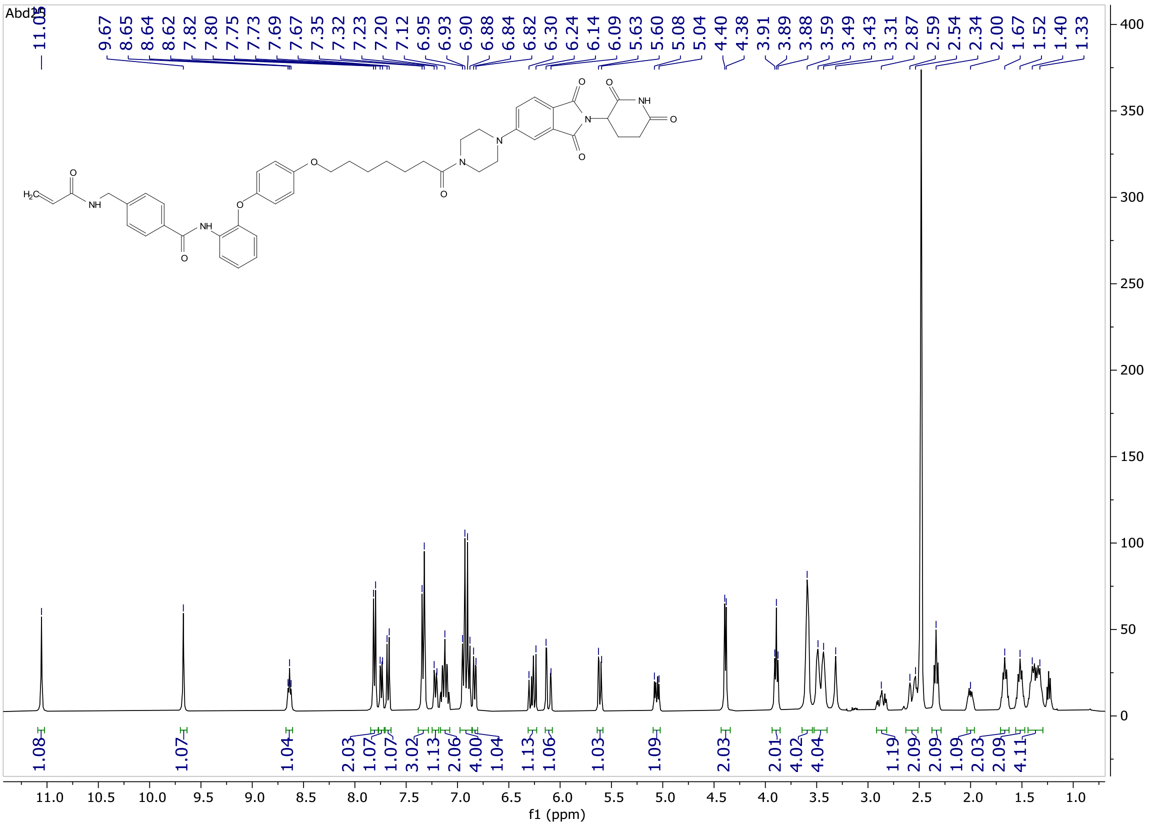

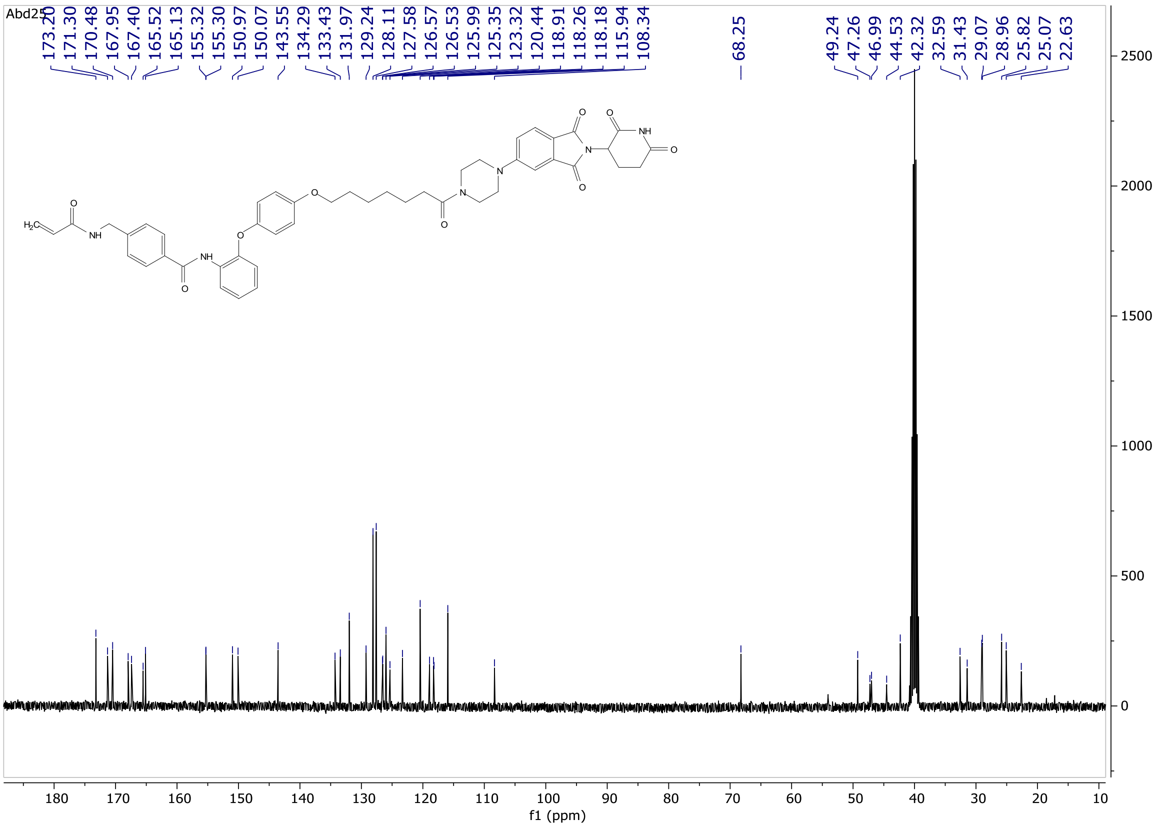

21b (Abd25)

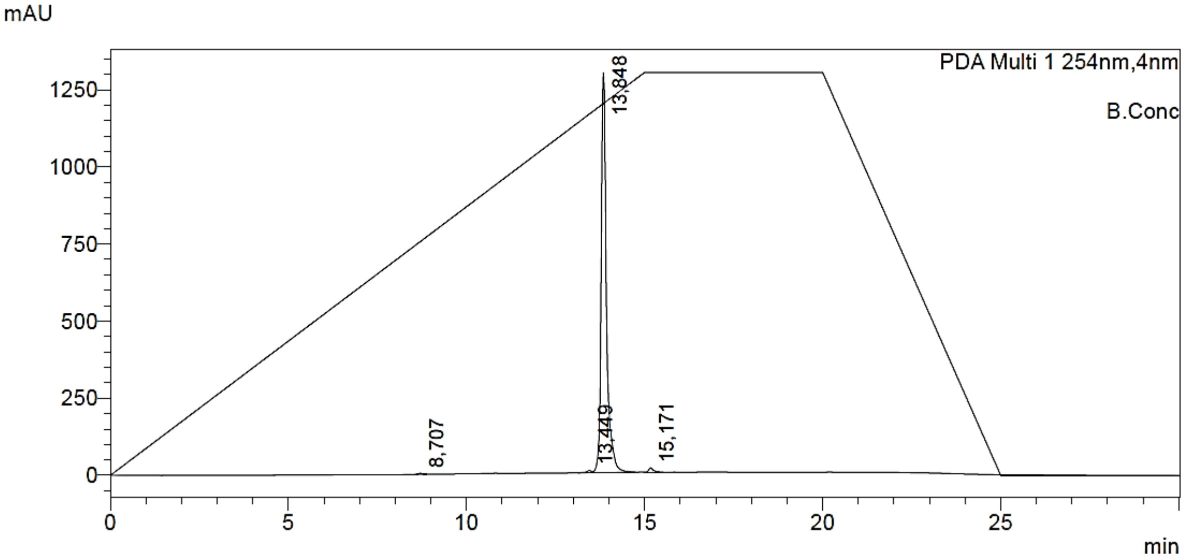

PDA Ch1 254nm

Peak#	Ret. Time	Area	Height	Area%
1	8,707	23171	3334	0,171
2	13,449	74320	7376	0,548
3	13,848	13320453	1297062	98,279
4	15,171	135776	14295	1,002
Total		13553720	1322067	100,000

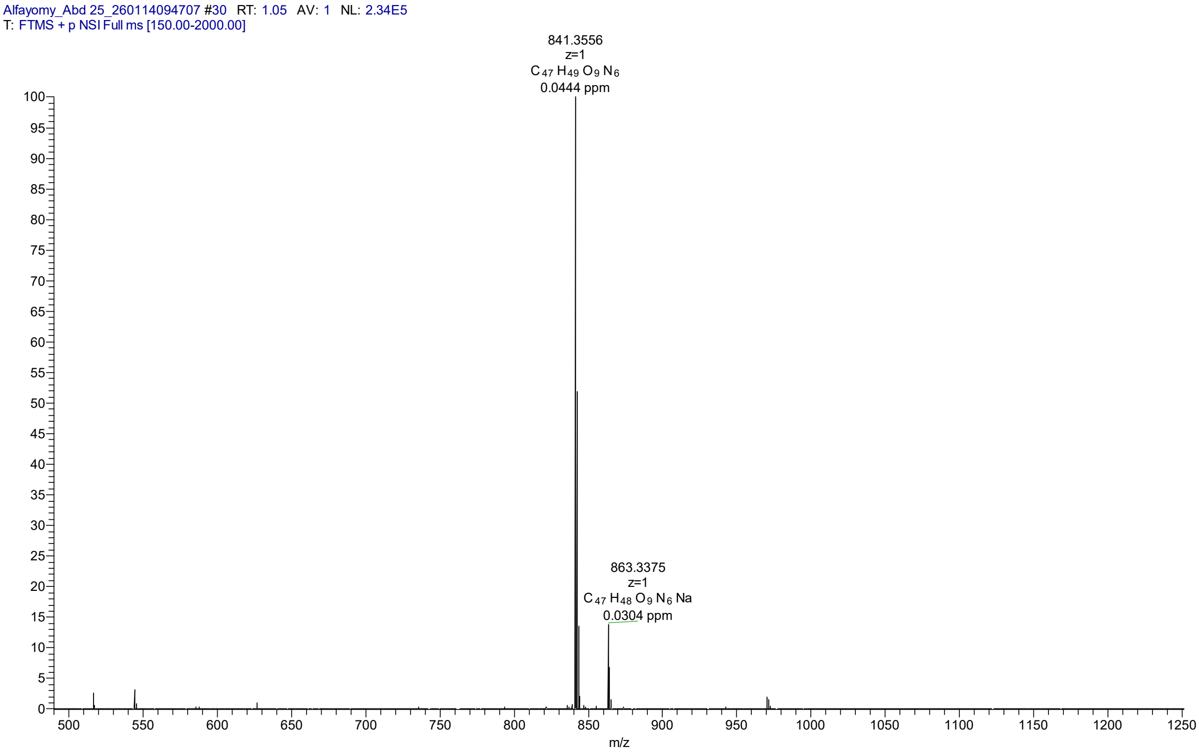

21c (Abd3)

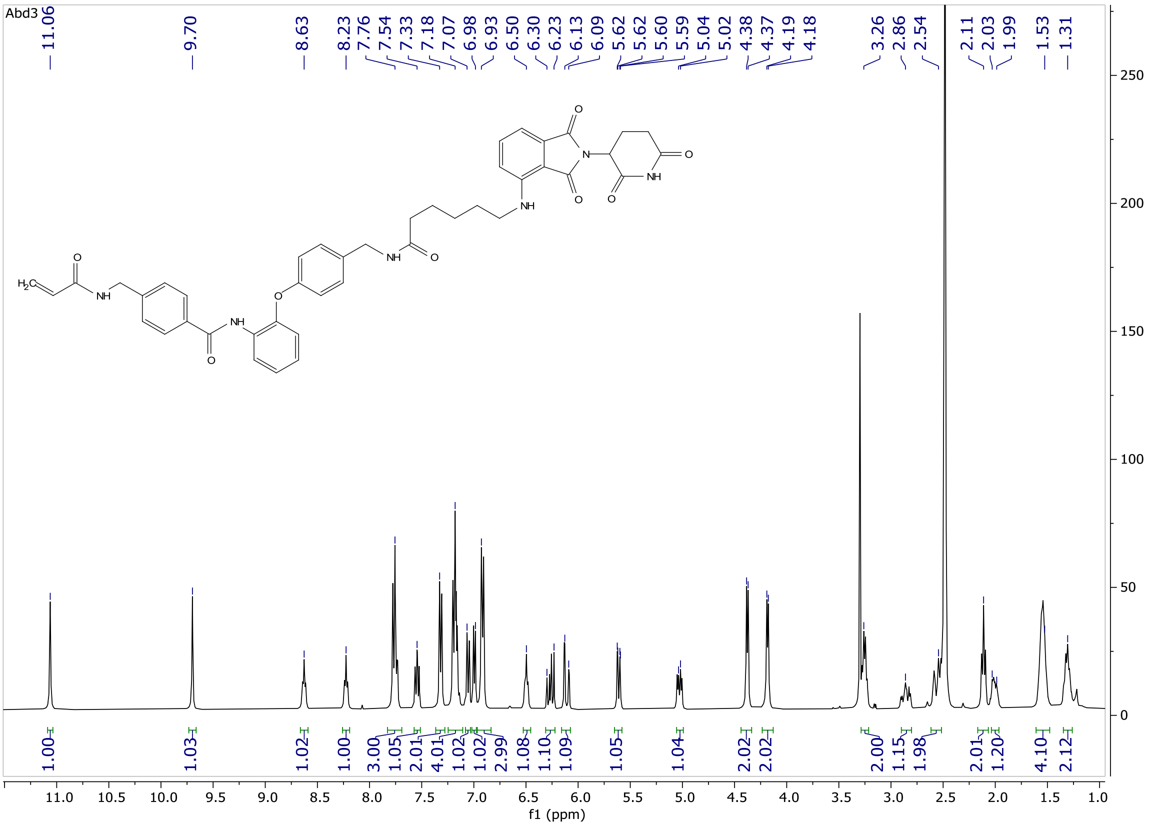

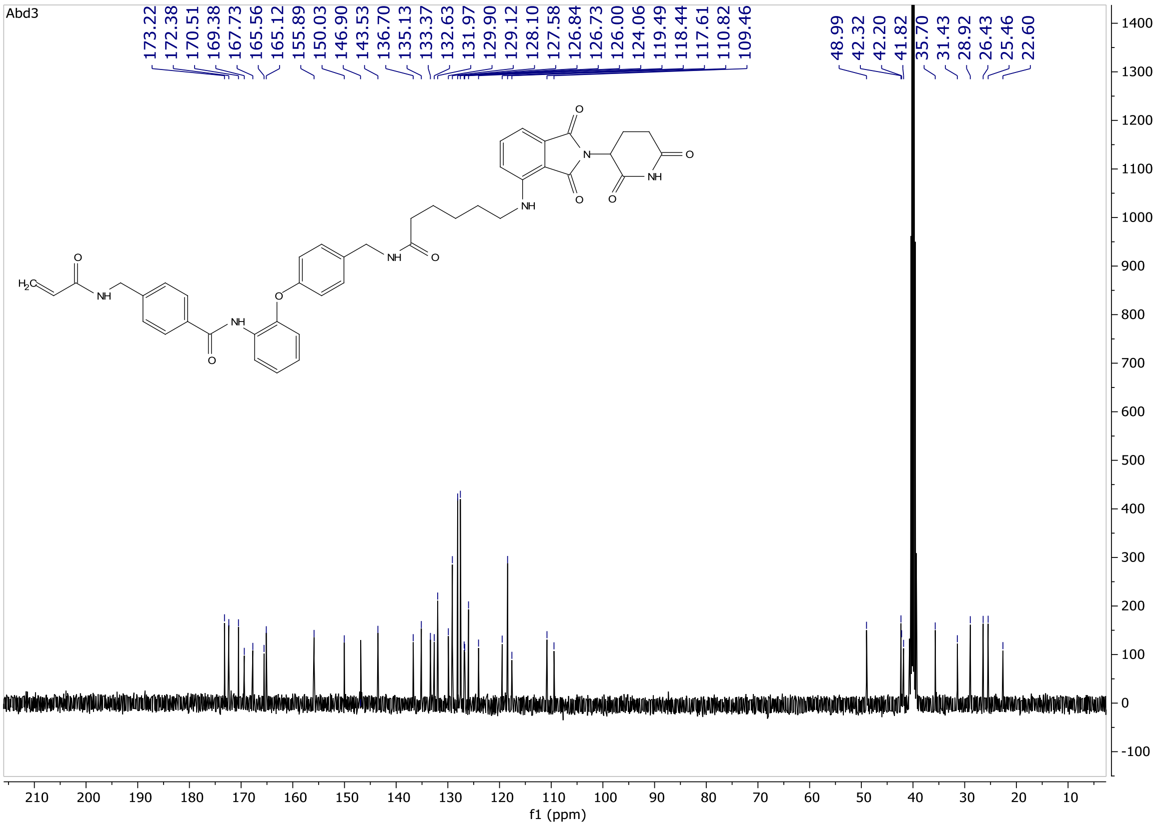

21c (Abd3)

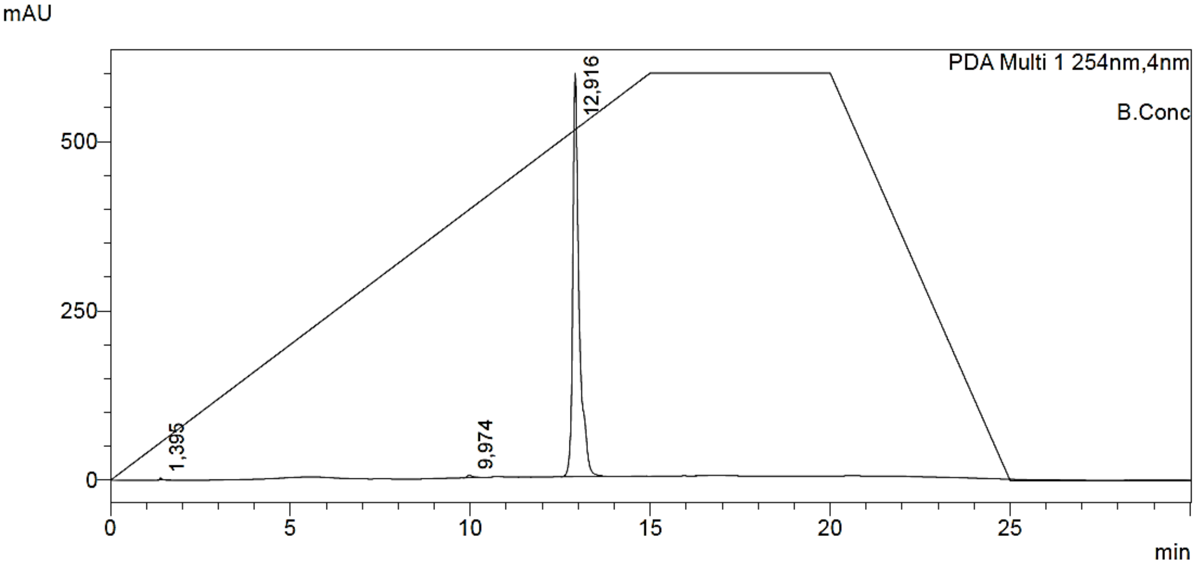

PDA Ch1 254nm

Peak#	Ret. Time	Area	Height	Area%
1	1,395	18331	3727	0,250
2	9,974	36718	3857	0,502
3	12,916	7265945	595690	99,248
Total		7320994	603274	100,000

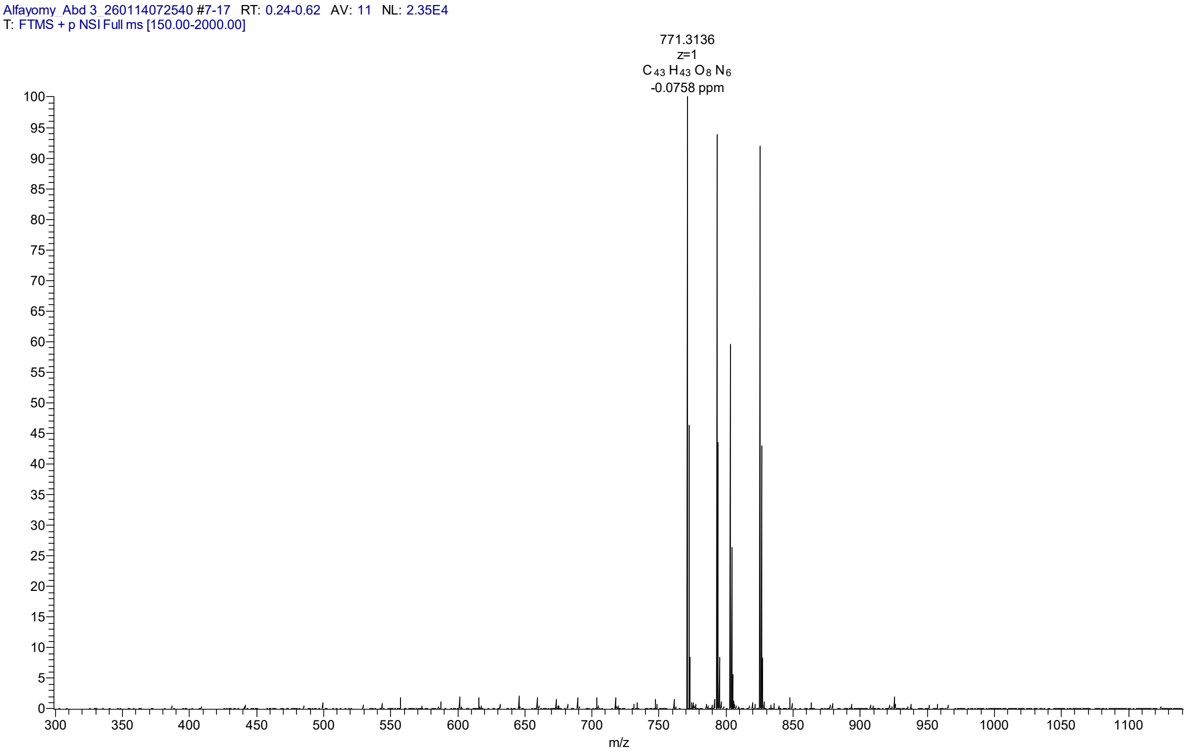

27a (Abd41)

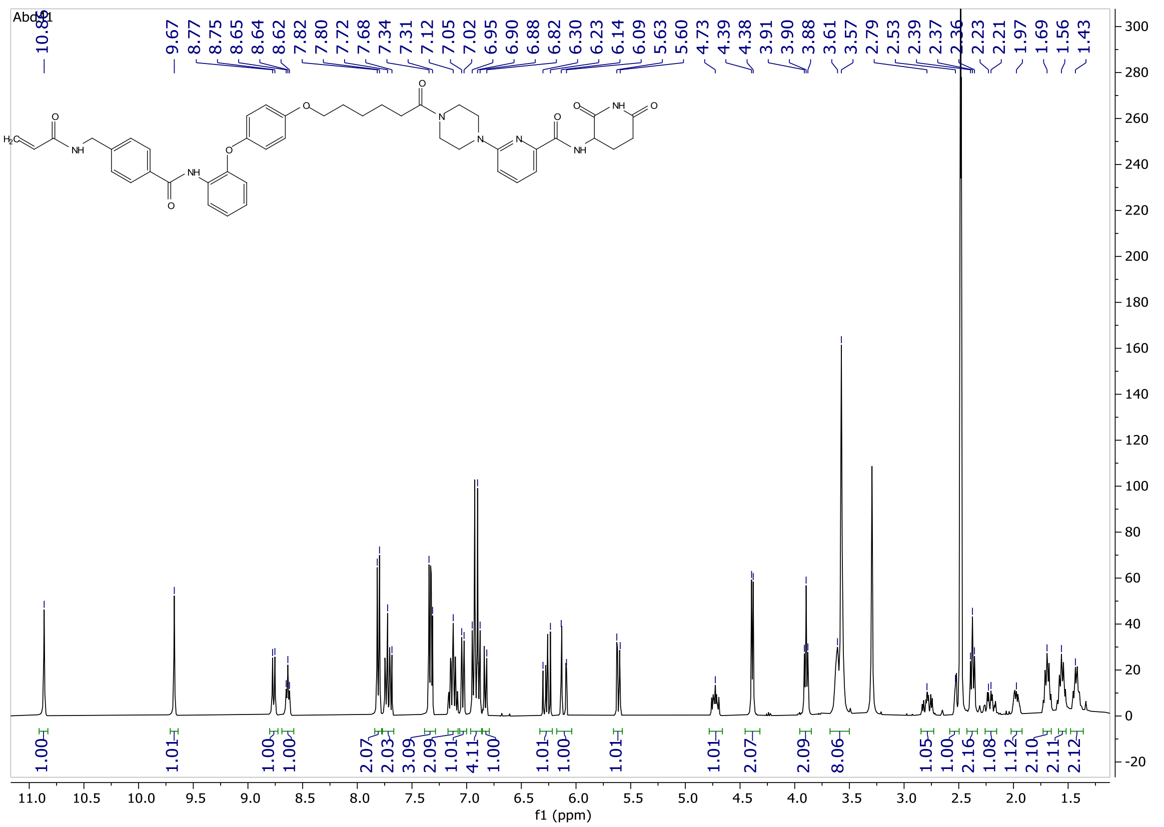

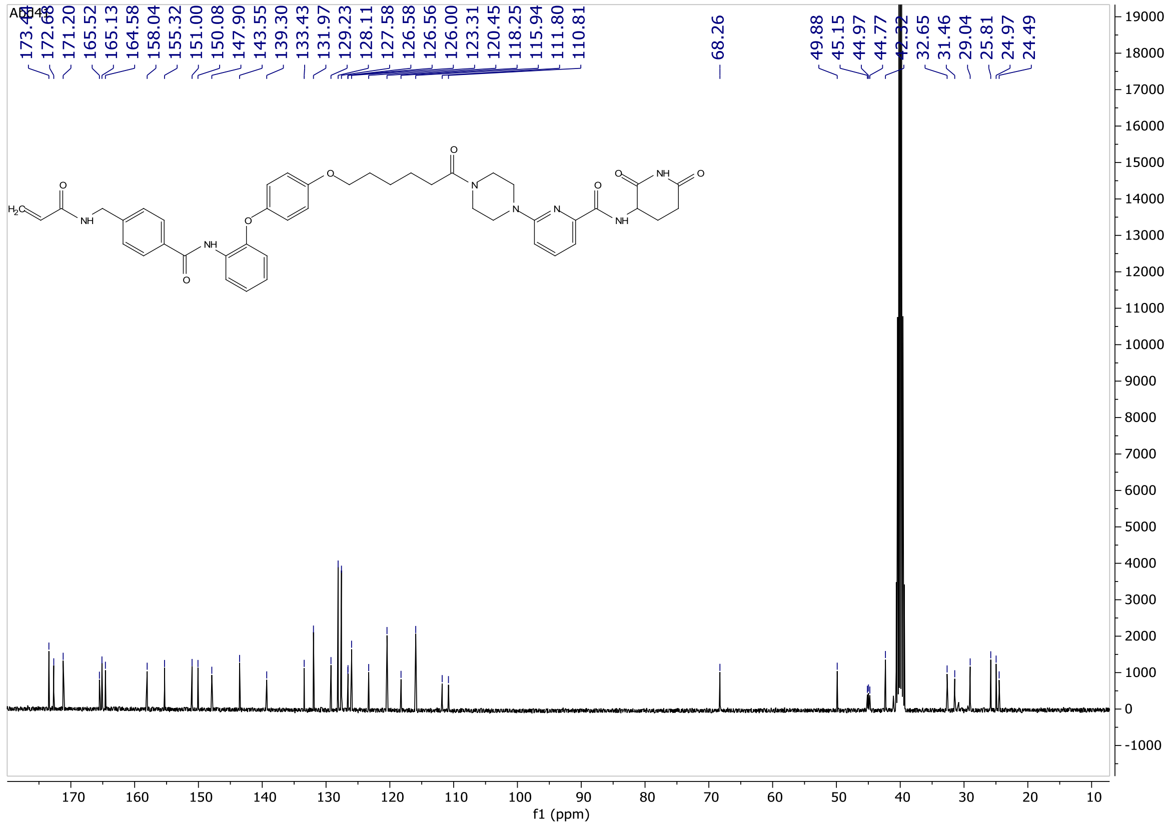

27a (Abd41)

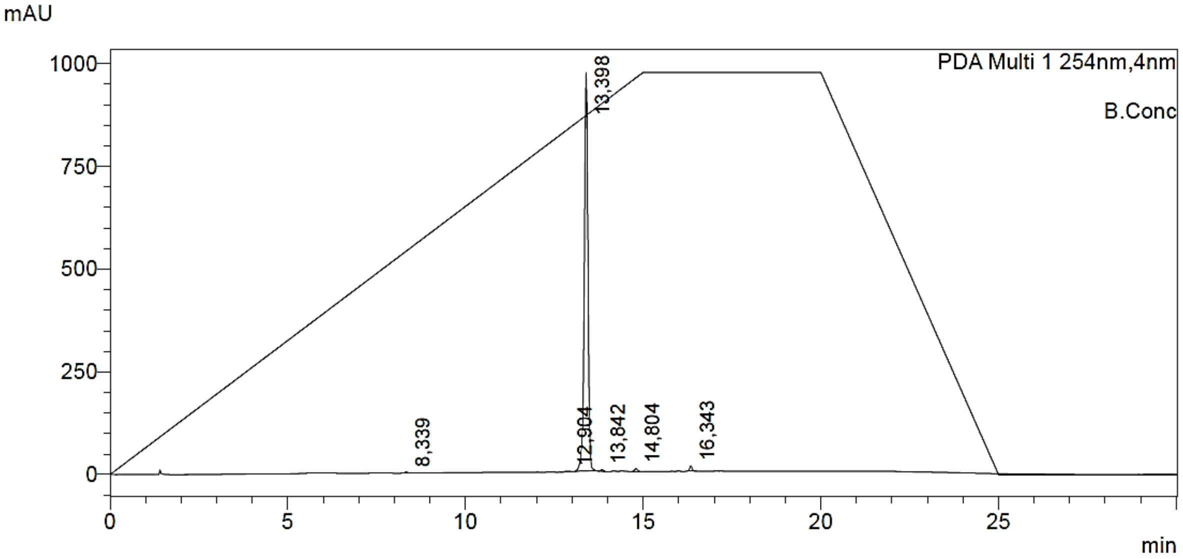

PDA Ch1 254nm

Peak#	Ret. Time	Area	Height	Area%
1	8,339	14726	2372	0,208
2	12,904	17534	1572	0,247
3	13,398	6930506	969853	97,816
4	13,842	17849	3773	0,252
5	14,804	41836	6879	0,590
6	16,343	62795	12911	0,886
Total		7085246	997360	100,000

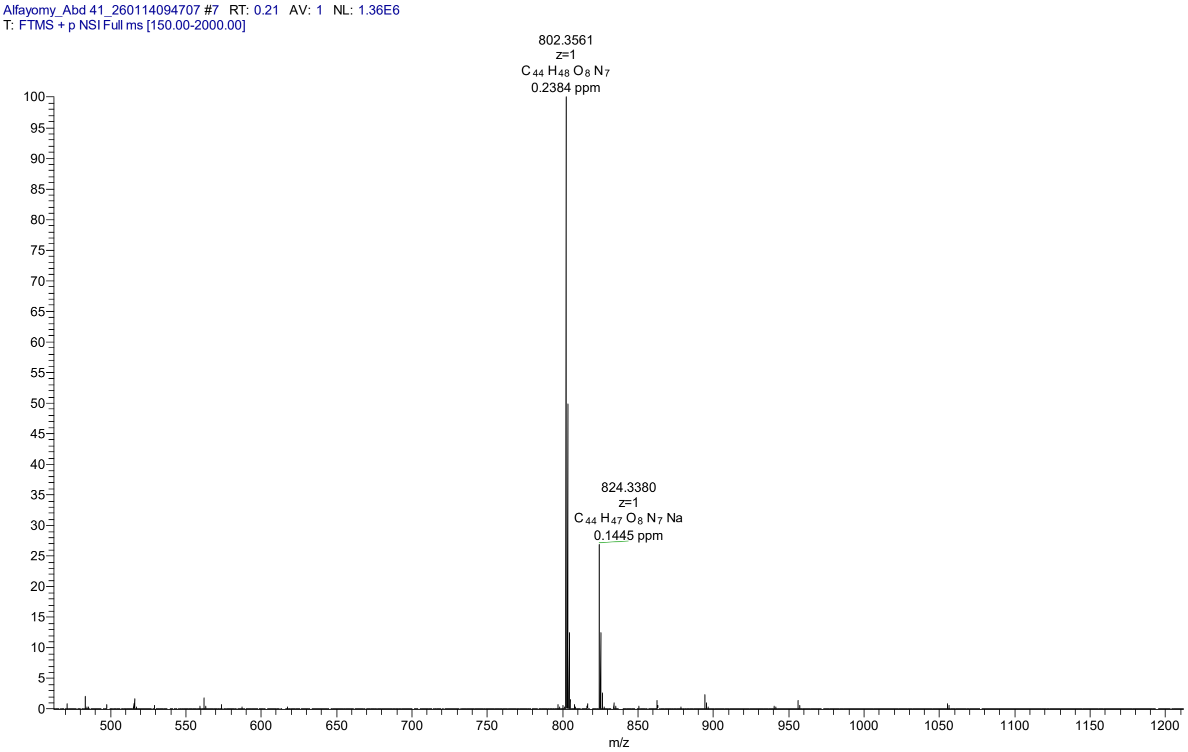

27b (Abd40)

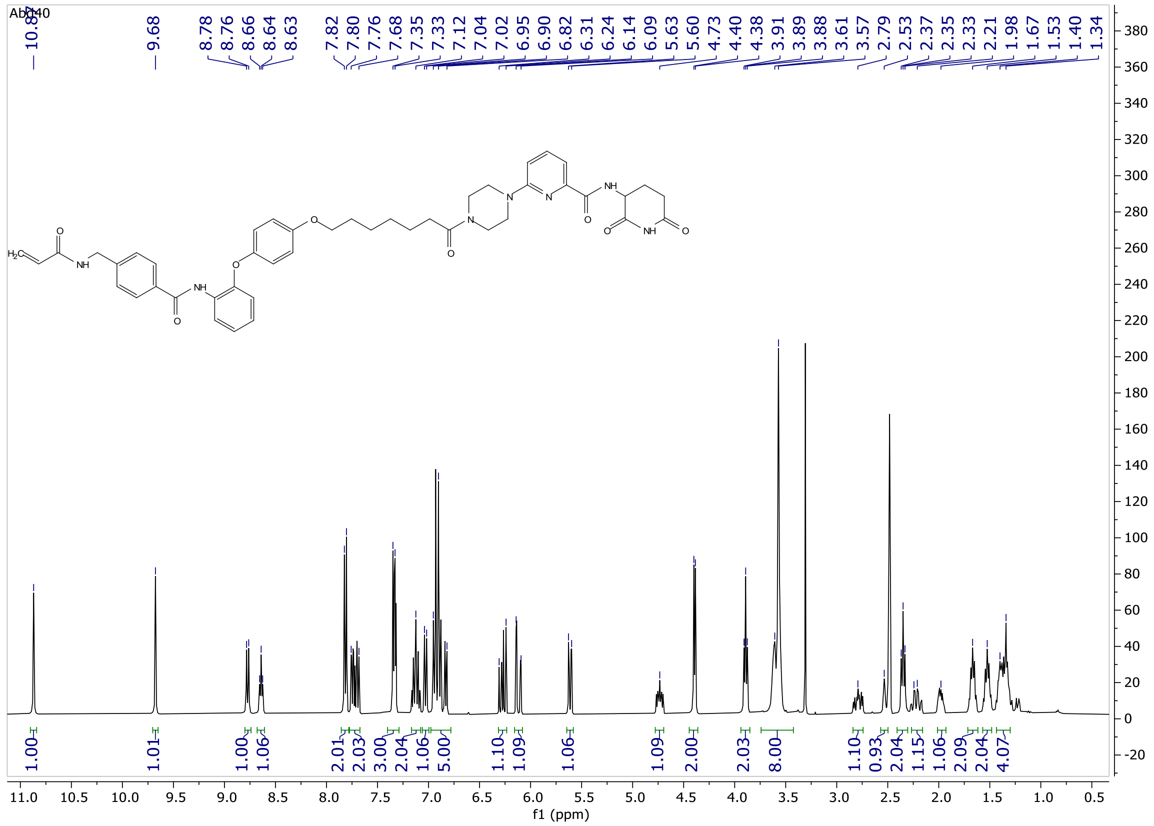

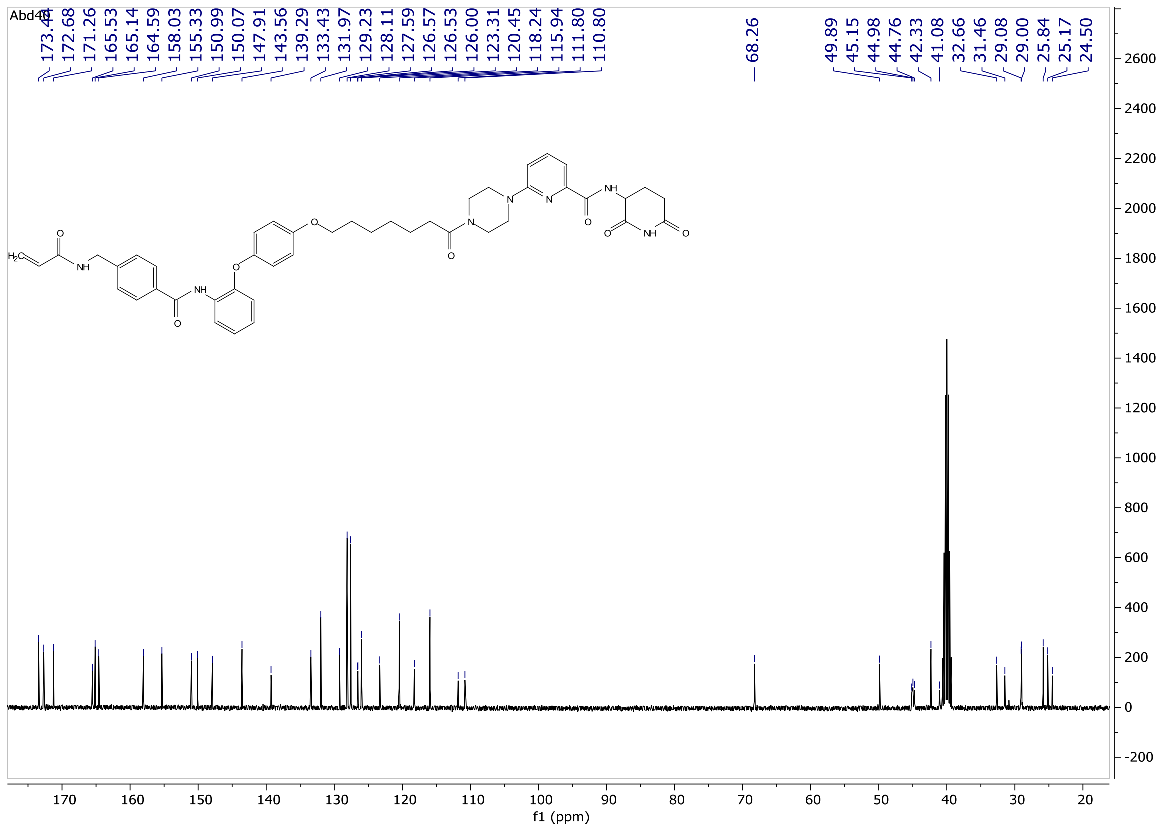

27b (Abd40)

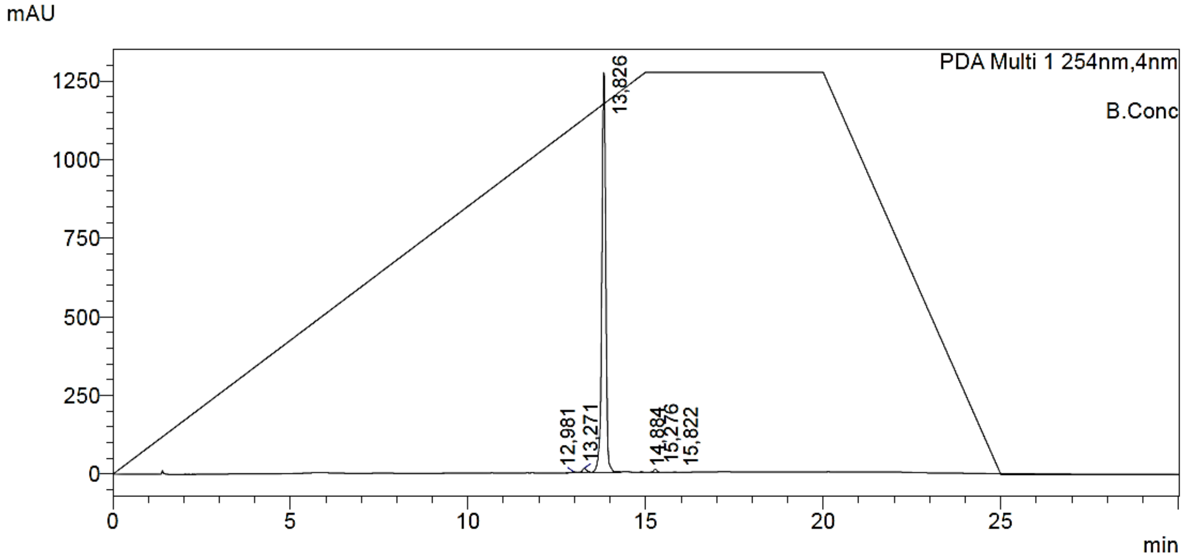

PDA Ch1 254nm

Peak#	Ret. Time	Area	Height	Area%
1	12,981	15380	2623	0,155
2	13,271	96357	12559	0,968
3	13,826	9757381	1270588	98,065
4	14,884	9099	1706	0,091
5	15,276	68995	10616	0,693
6	15,822	2679	574	0,027
Total		9949892	1298666	100,000

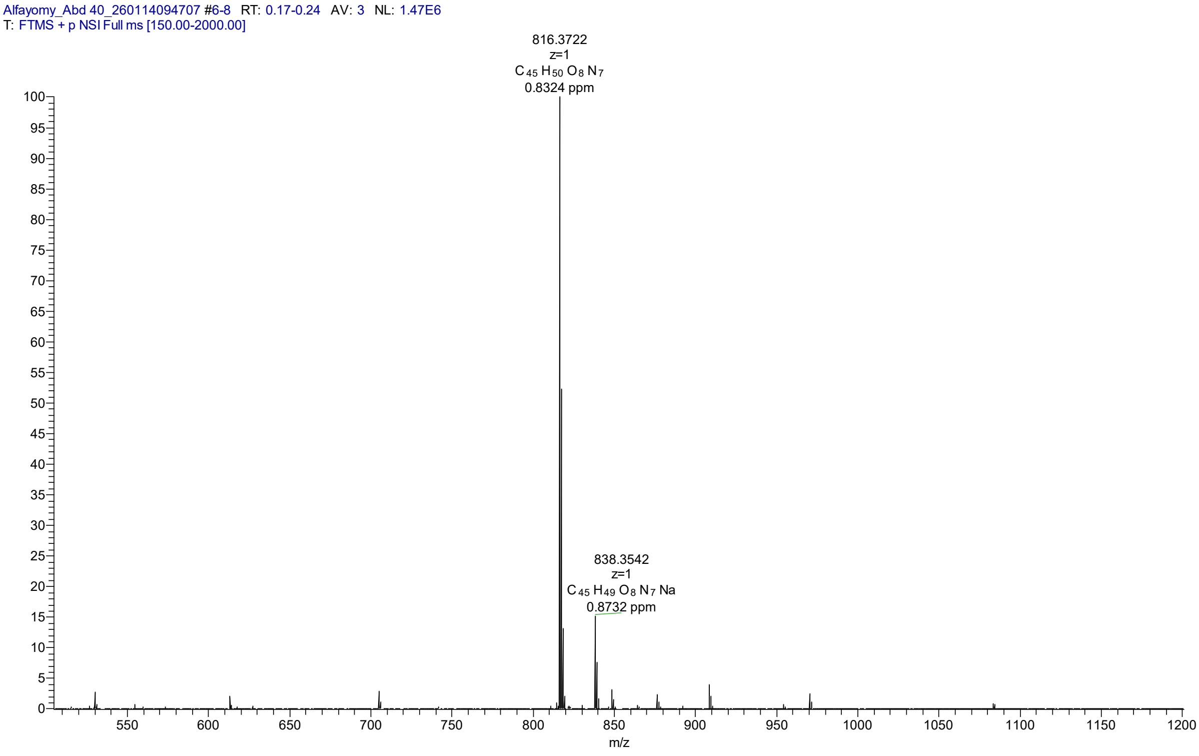

32a (Abd63)

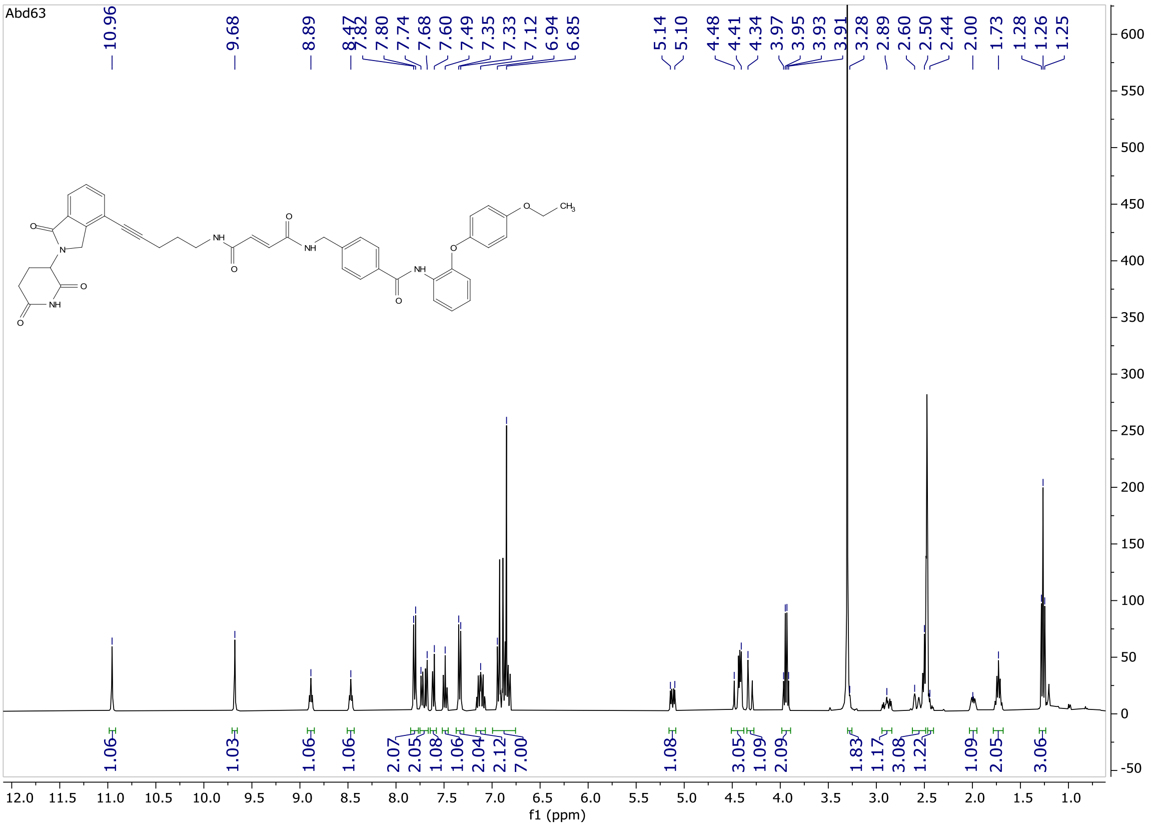

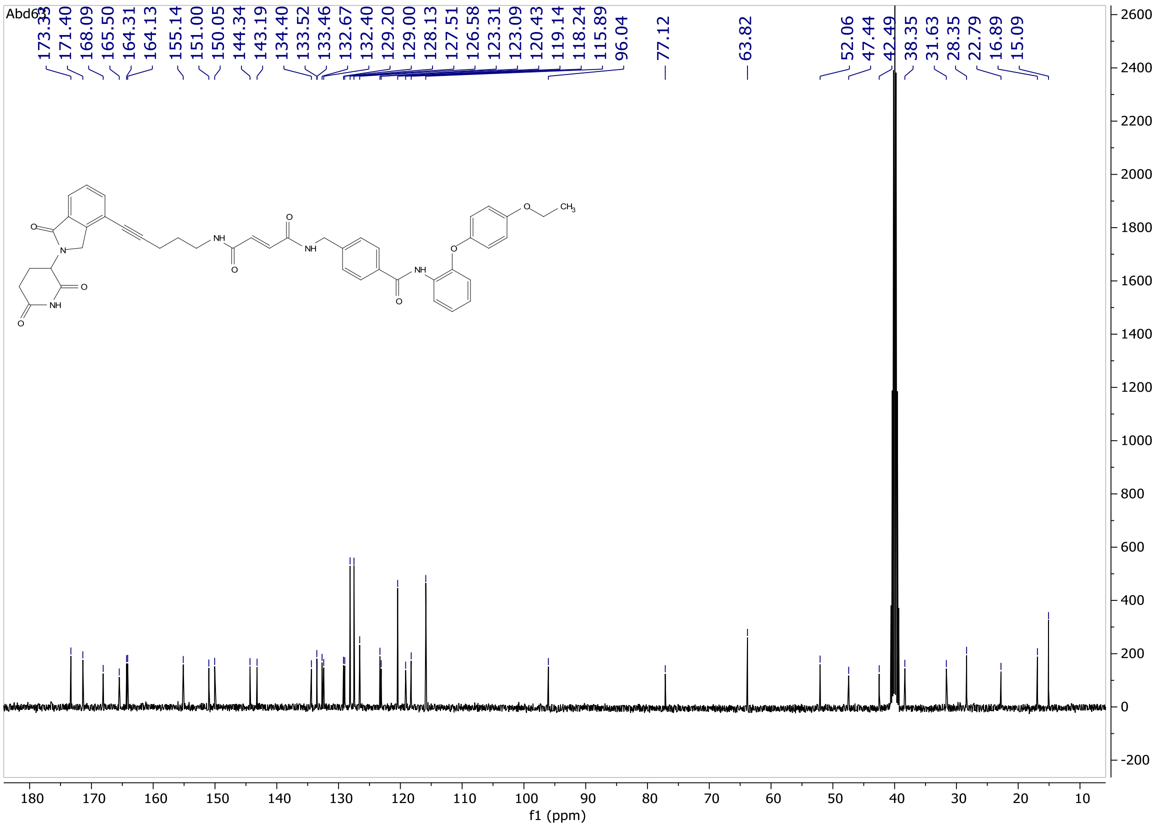

32a (Abd63)

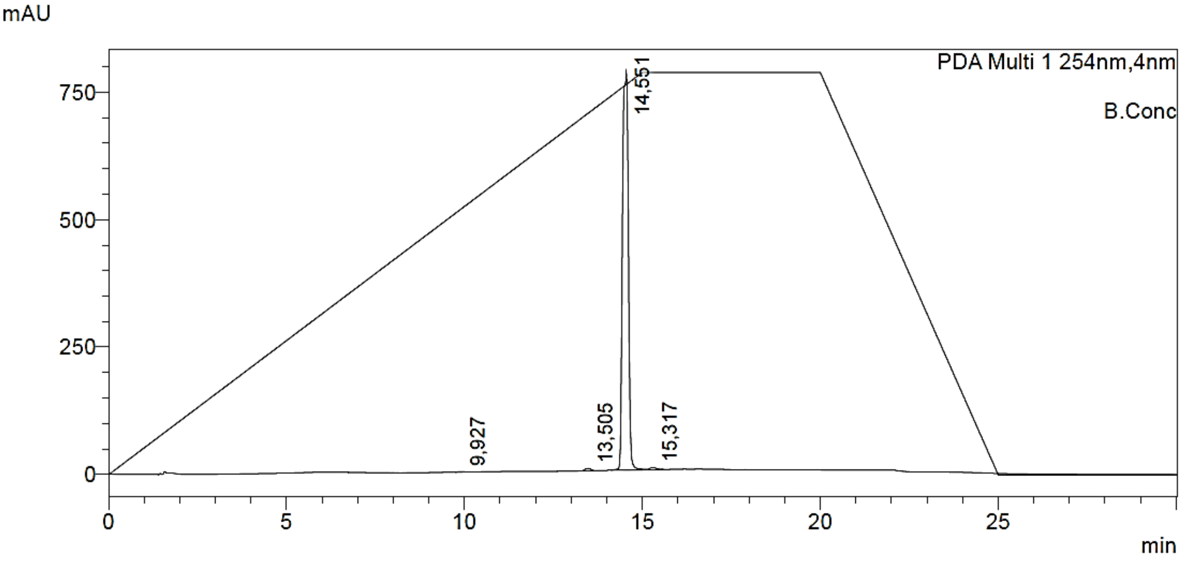

Peak Table

PDA Ch1 254nm

Peak#	Ret. Time	Area	Height	Area%
1	9,927	10614	1006	0,117
2	13,505	48506	4354	0,534
3	14,551	8958966	780544	98,713
4	15,317	57731	4280	0,636
Total		9075816	790184	100,000

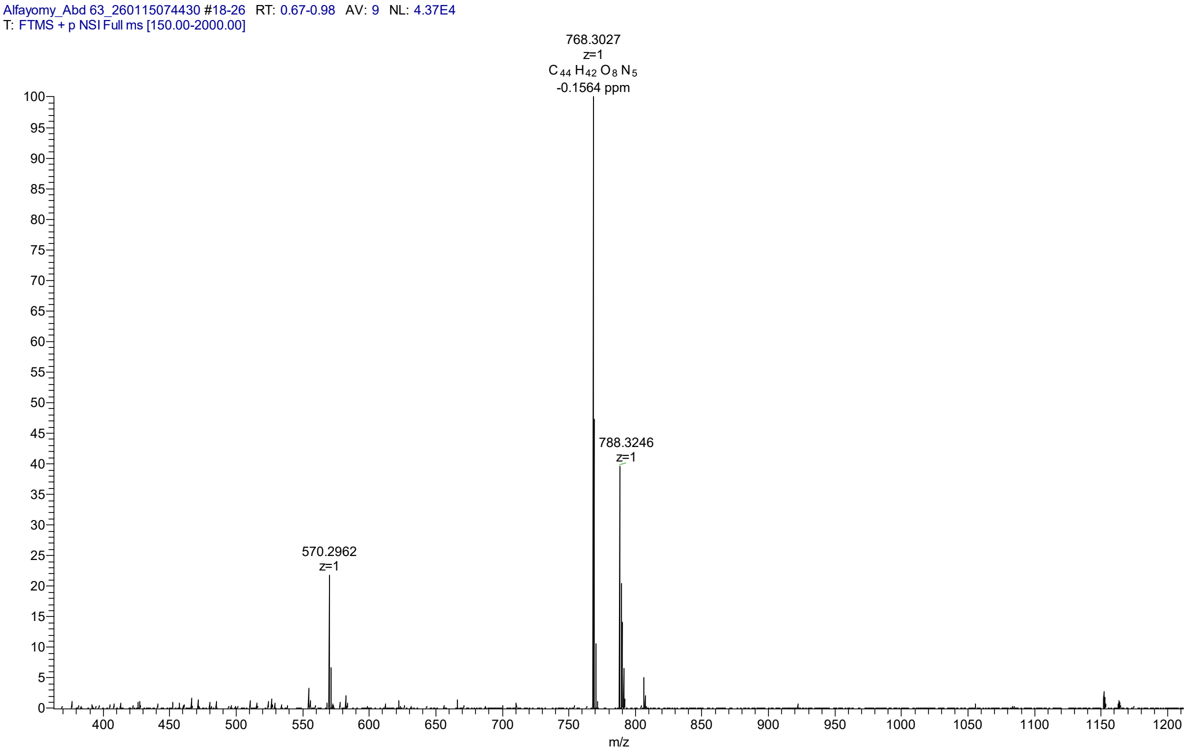

32b (Abd66)

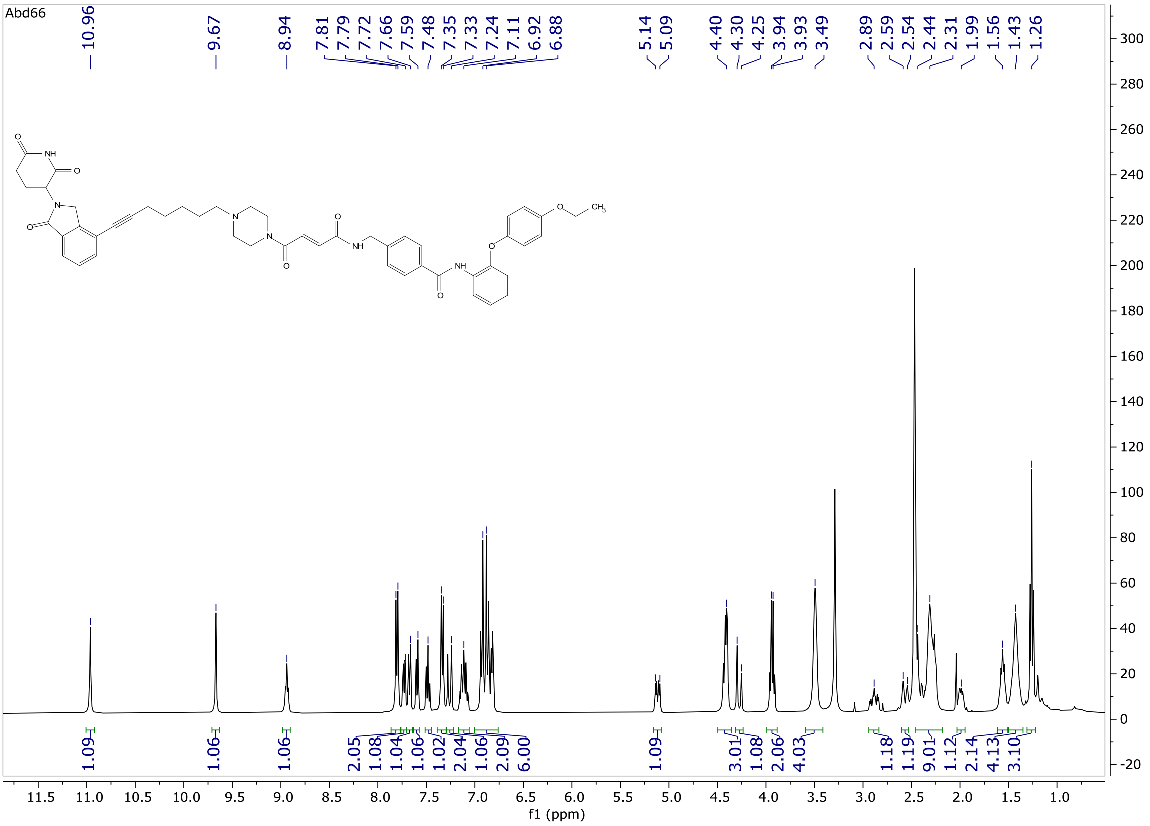

Abd66
10.96
9.67
8.94
7.81 7.79 7.72 7.66 7.59 7.48 7.35 7.33 7.24 7.11 6.92 6.88
5.14 5.09
4.40 4.30 4.25 3.94 3.93 3.49
2.89 2.59 2.54 2.44 2.31 1.99 1.56 1.43 1.26
1.09 1.06 1.06 2.05 1.08 1.04 1.06 1.02 2.04 1.06 2.09 6.00 1.09 3.01 1.08 2.06 4.03 1.18 1.19 9.01 1.12 2.14 4.13 3.10
f1 (ppm)

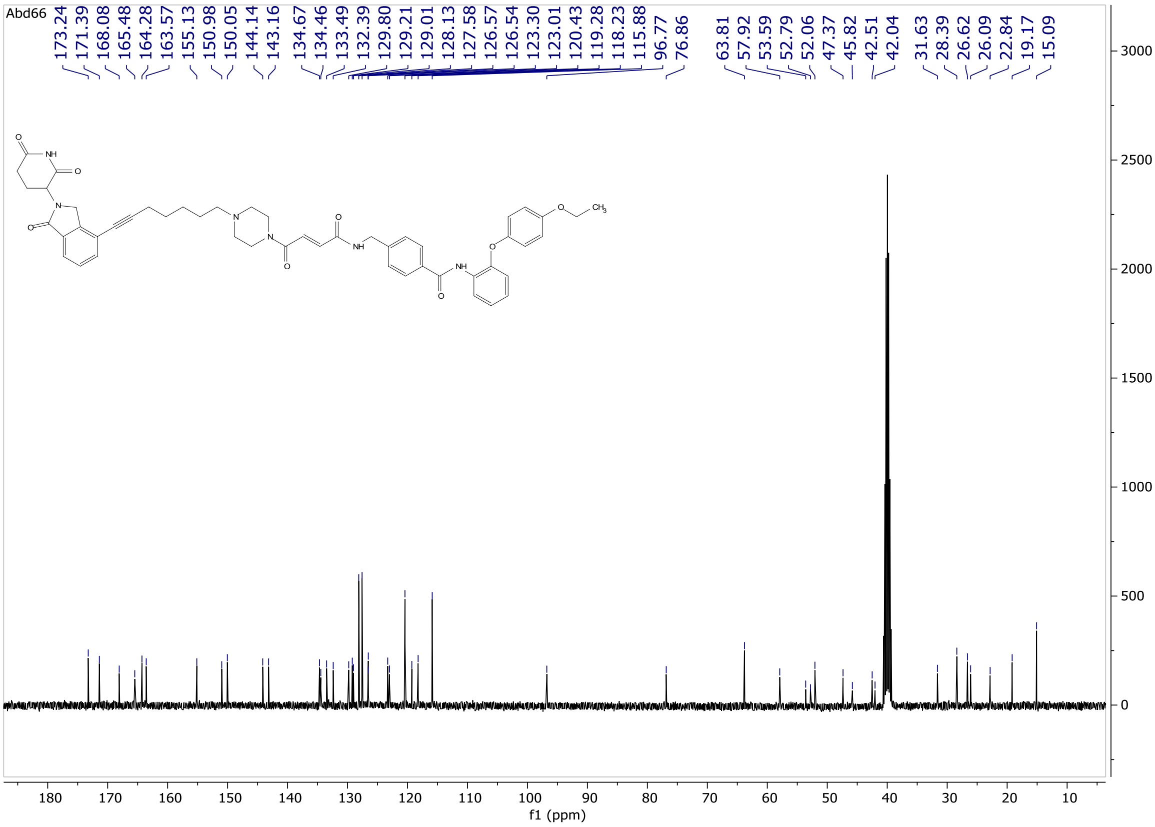

Abd66
173.24 171.39 168.08 165.48 164.28 163.57 155.13 150.98 150.05 144.14 143.16 134.67 134.46 133.49 132.39 129.80 129.21 129.01 128.13 127.58 126.57 126.54 123.30 123.01 120.43 119.28 118.23 115.88 96.77 76.86
63.81 57.92 53.59 52.79 52.06 47.37 45.82 42.51 42.04 31.63 28.39 26.62 26.09 22.84 19.17 15.09
f1 (ppm)

32b (Abd66)

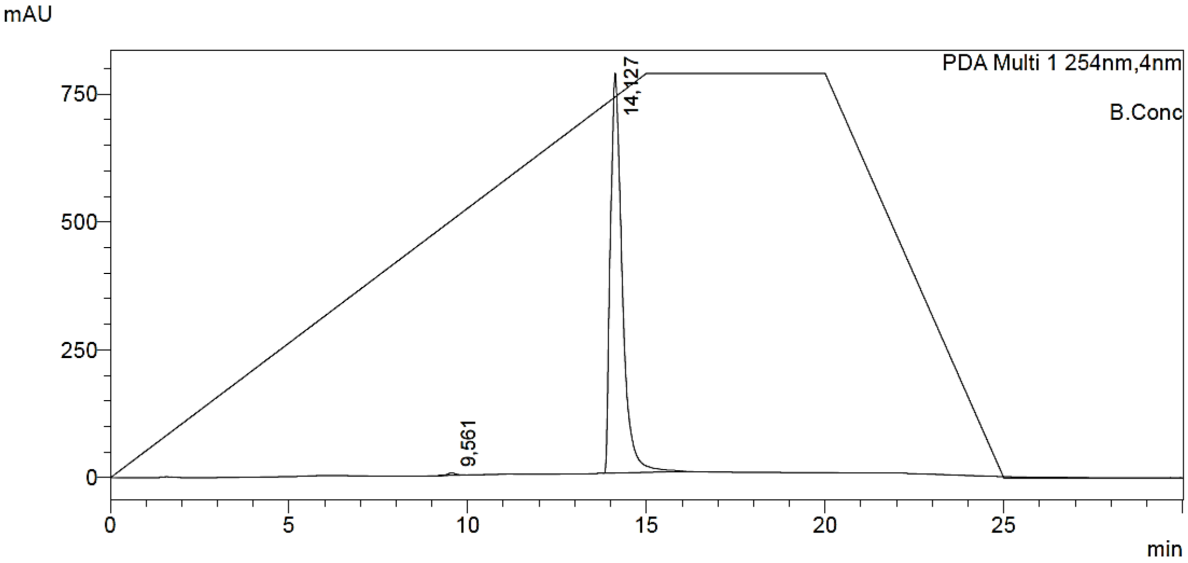

PDA Ch1 254nm

Peak#	Ret. Time	Area	Height	Area%
1	9,561	81314	4788	0,448
2	14,127	18052578	781058	99,552
Total		18133892	785846	100,000

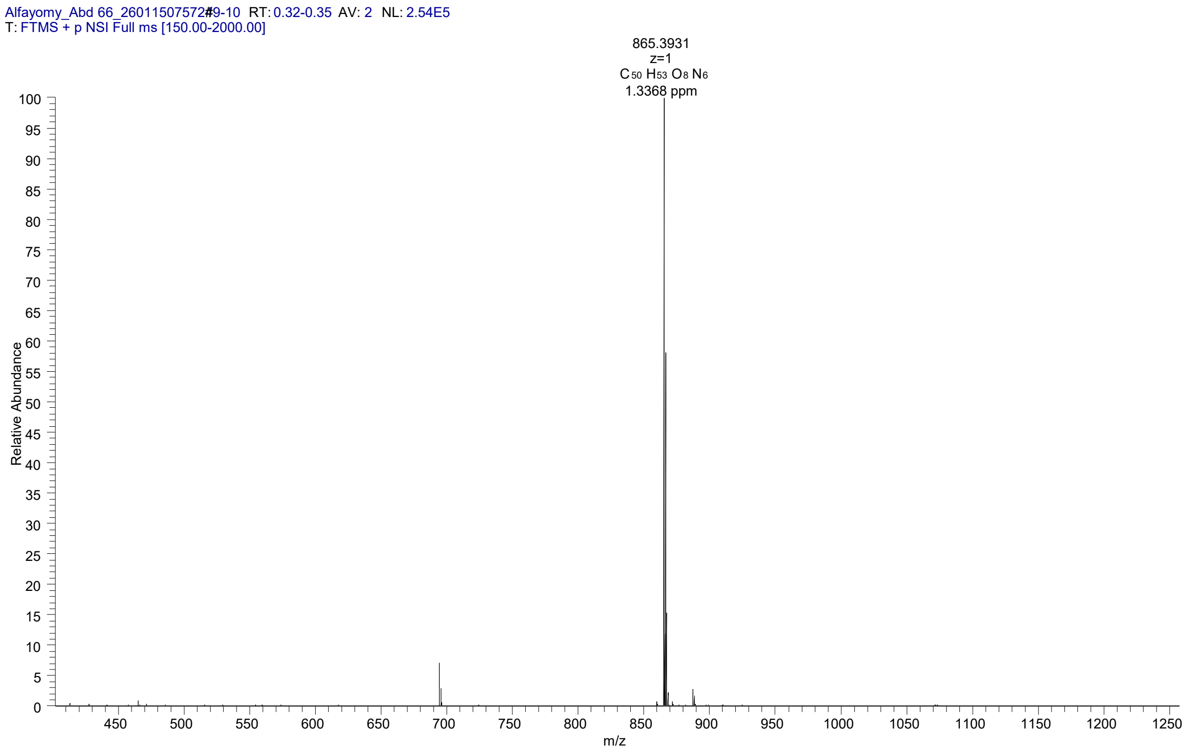

32c (Abd59)

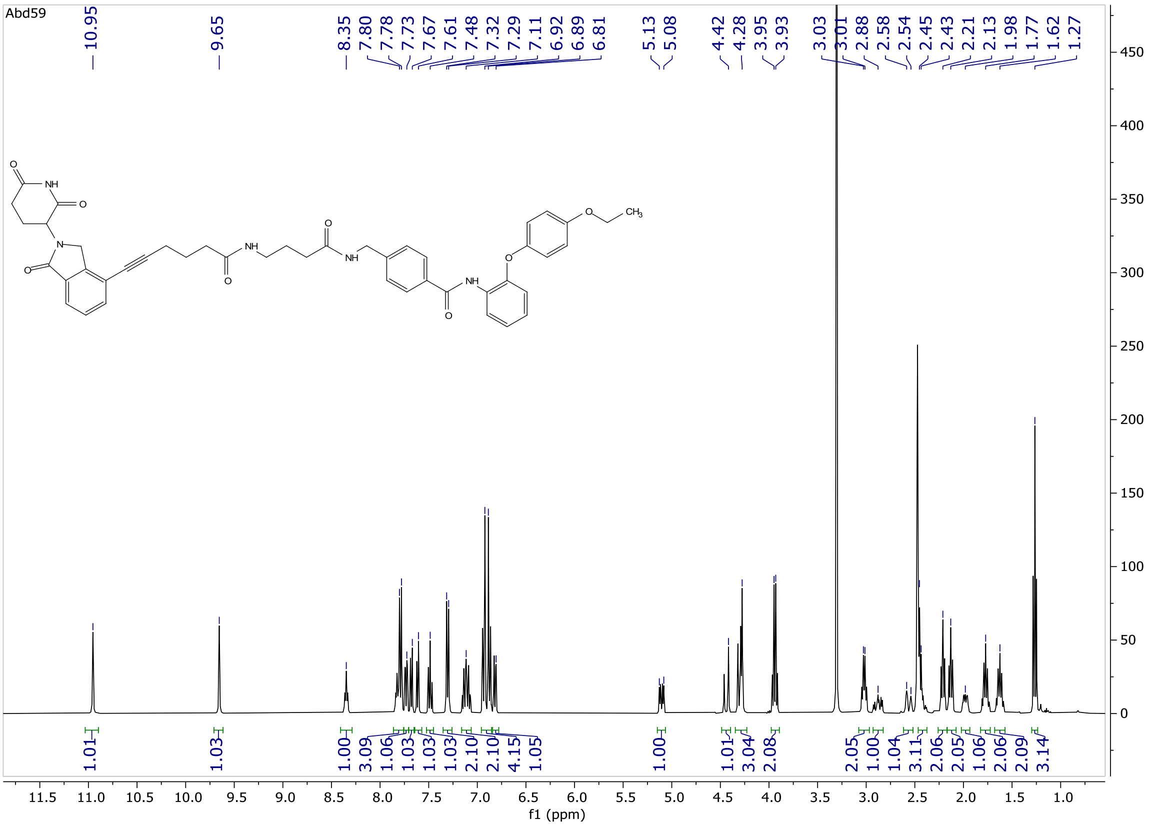
Abd59
10.95
9.65
8.35
7.80
7.78
7.73
7.67
7.61
7.48
7.32
7.29
7.11
6.92
6.89
6.81
5.13
5.08
4.42
4.28
3.95
3.93
3.03
3.01
2.88
2.58
2.54
2.45
2.43
2.21
2.13
1.98
1.77
1.62
1.27
1.01
1.03
1.00
3.09
1.06
1.03
1.03
2.10
2.10
4.15
1.05
1.00
1.01
3.04
2.08
2.05
1.00
1.04
3.11
2.06
2.05
1.06
2.06
2.09
3.14
f1 (ppm)

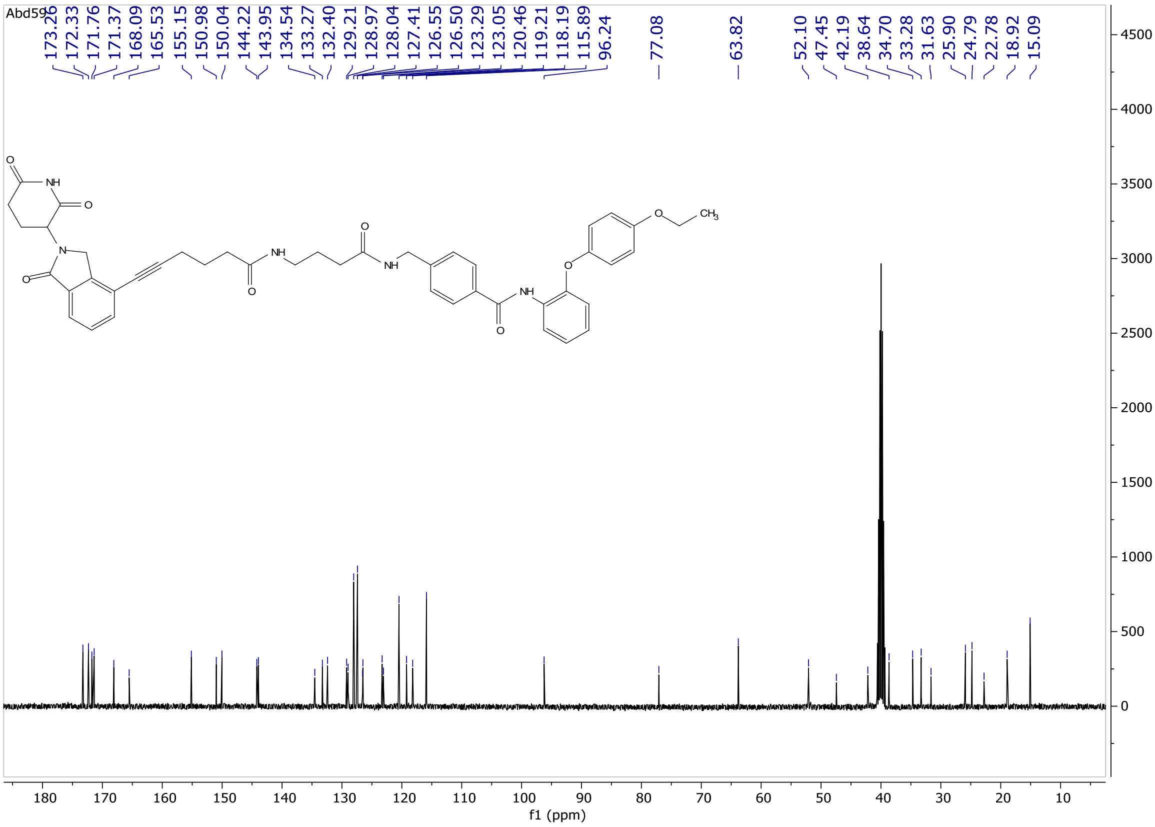
Abd59
173.26
172.33
171.76
171.37
168.09
165.53
155.15
150.98
150.04
144.22
143.95
134.54
133.27
132.40
129.21
128.97
128.04
127.41
126.55
126.50
123.29
123.05
120.46
119.21
118.19
115.89
96.24
77.08
63.82
52.10
47.45
42.19
38.64
34.70
33.28
31.63
25.90
24.79
22.78
18.92
15.09
f1 (ppm)

32c (Abd59)

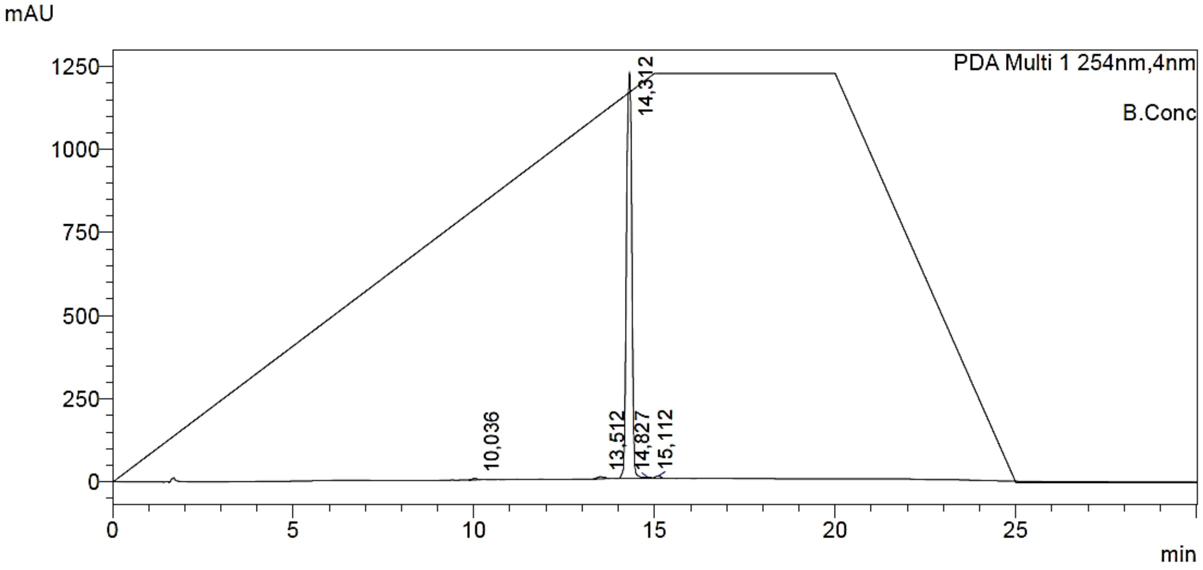

PDA Ch1 254nm

Peak#	Ret. Time	Area	Height	Area%
1	10,036	40193	4790	0,316
2	13,512	81771	6369	0,642
3	14,312	12490242	1218778	98,084
4	14,827	39829	2801	0,313
5	15,112	82183	7507	0,645
Total		12734219	1240244	100,000

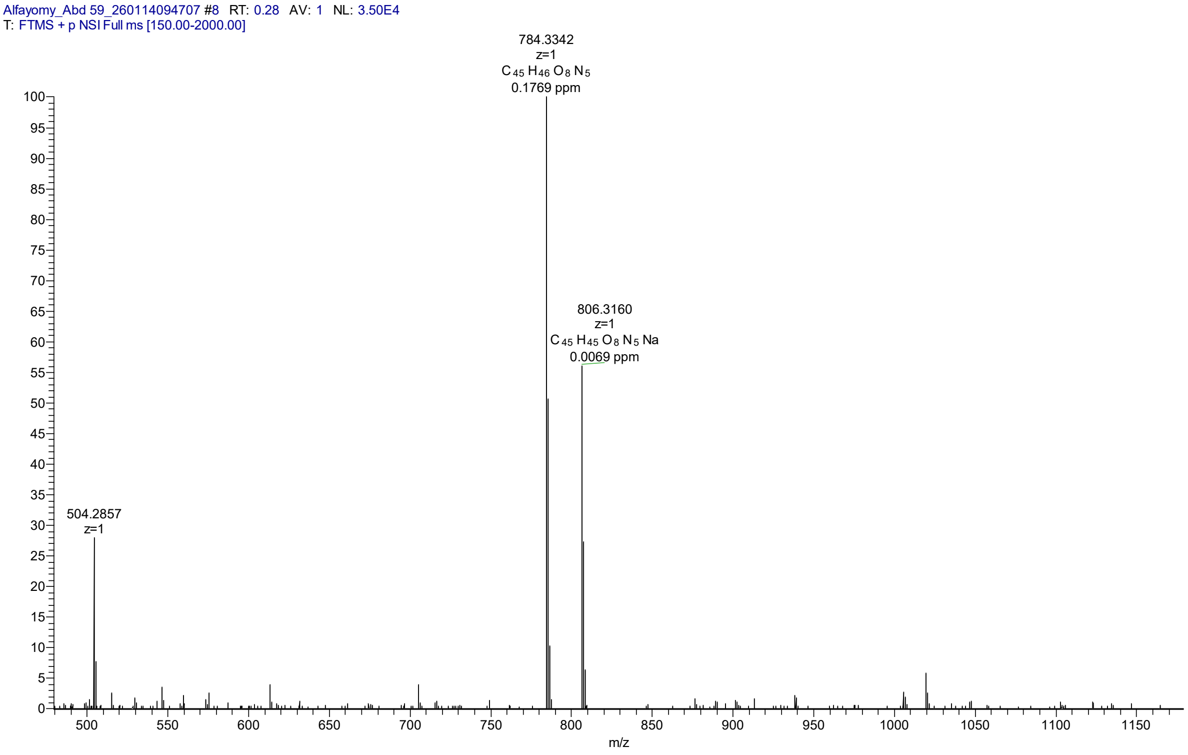

32d (Abd62)

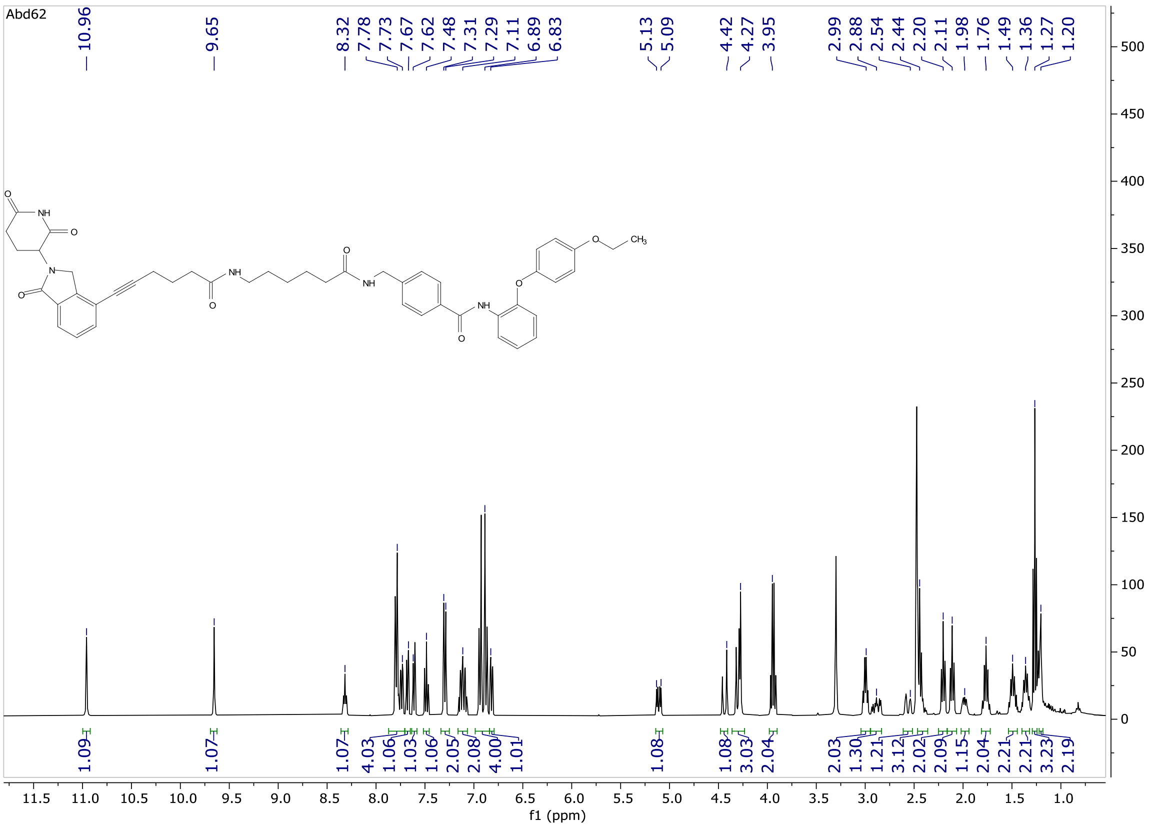

Abd62
10.96
9.65
8.32
7.78
7.73
7.67
7.62
7.48
7.31
7.29
7.11
6.89
6.83
5.13
5.09
4.42
4.27
3.95
2.99
2.88
2.54
2.44
2.20
2.11
1.98
1.76
1.49
1.36
1.27
1.20
f1 (ppm)

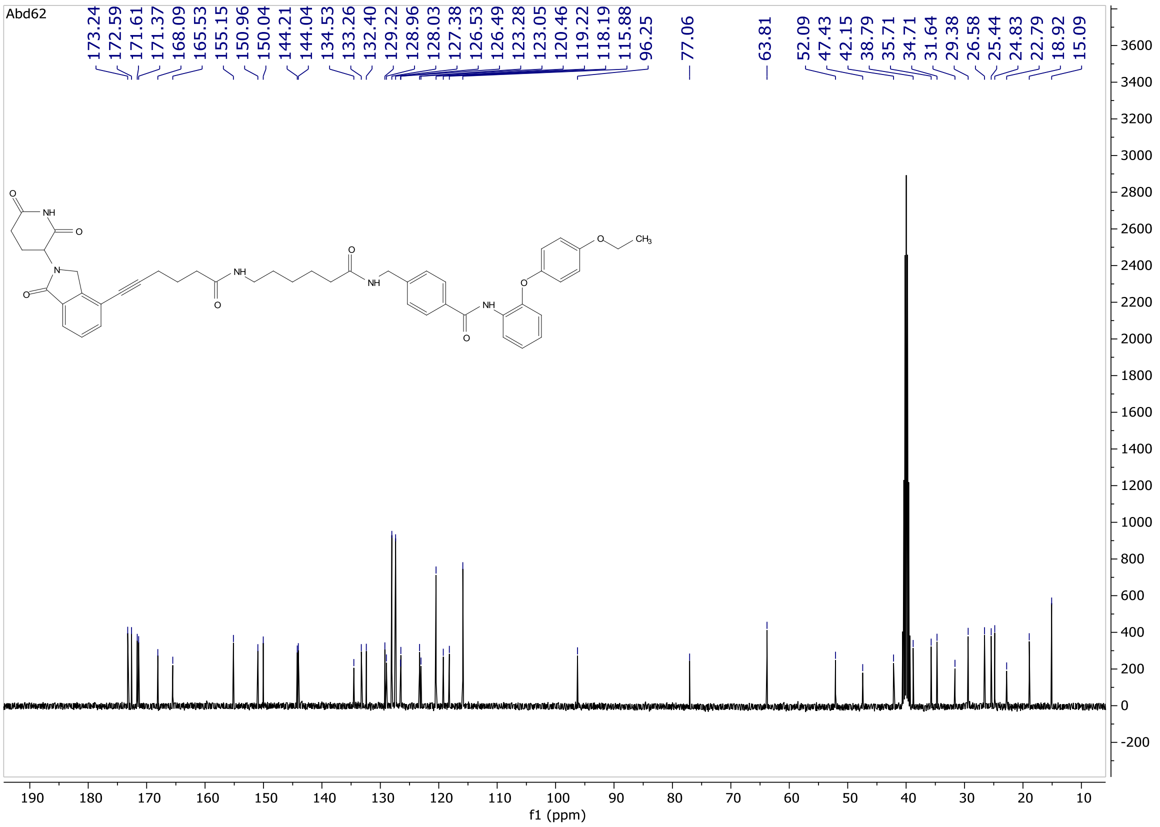

Abd62
173.24
172.59
171.61
171.37
168.09
165.53
155.15
150.96
150.04
144.21
144.04
134.53
133.26
132.40
129.22
128.96
128.03
127.38
126.53
126.49
123.28
123.05
120.46
119.22
118.19
115.88
96.25
77.06
63.81
52.09
47.43
42.15
38.79
35.71
34.71
31.64
29.38
26.58
25.44
24.83
22.79
18.92
15.09
f1 (ppm)

32d (Abd62)

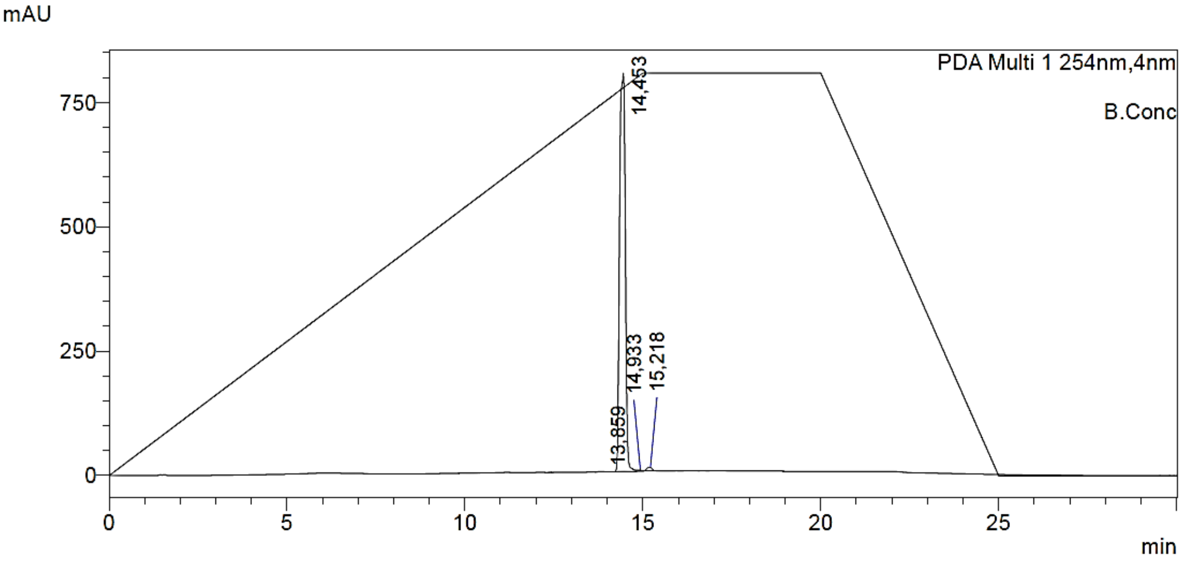

PDA Ch1 254nm

Peak#	Ret. Time	Area	Height	Area%
1	13,859	13562	1145	0,150
2	14,453	8928432	800665	98,739
3	14,933	22395	1925	0,248
4	15,218	78045	6860	0,863
Total		9042434	810595	100,000

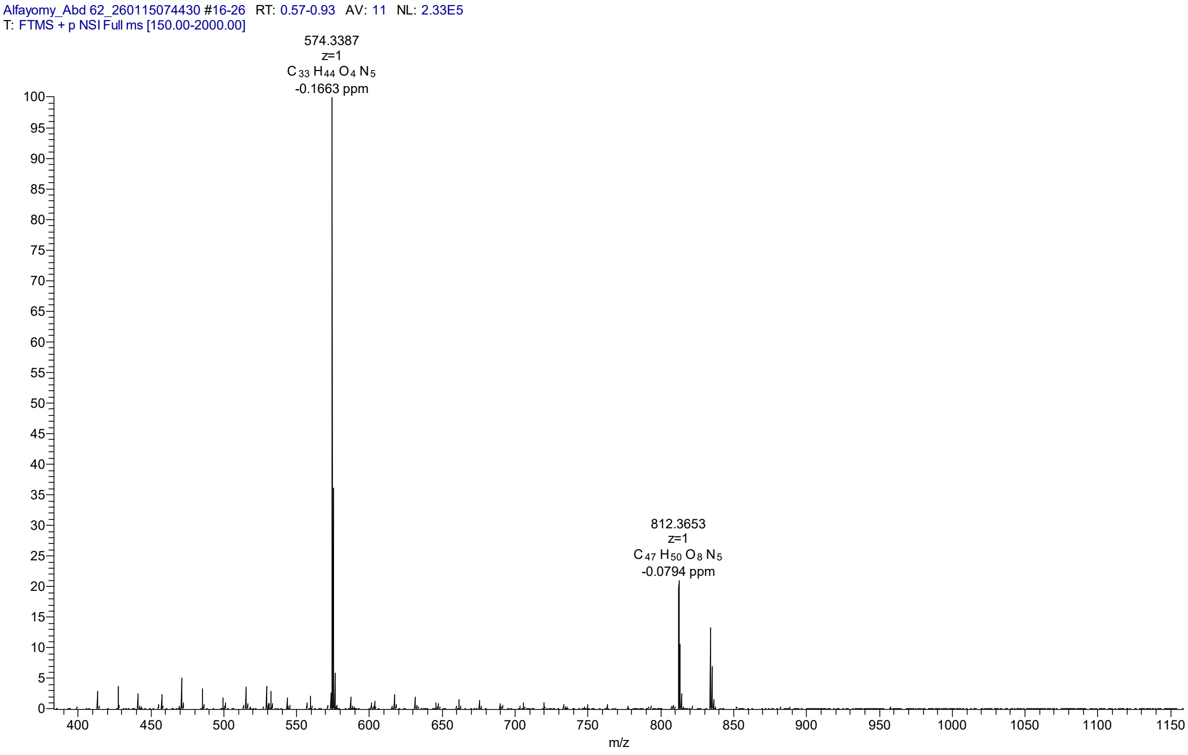

32e (Abd61)

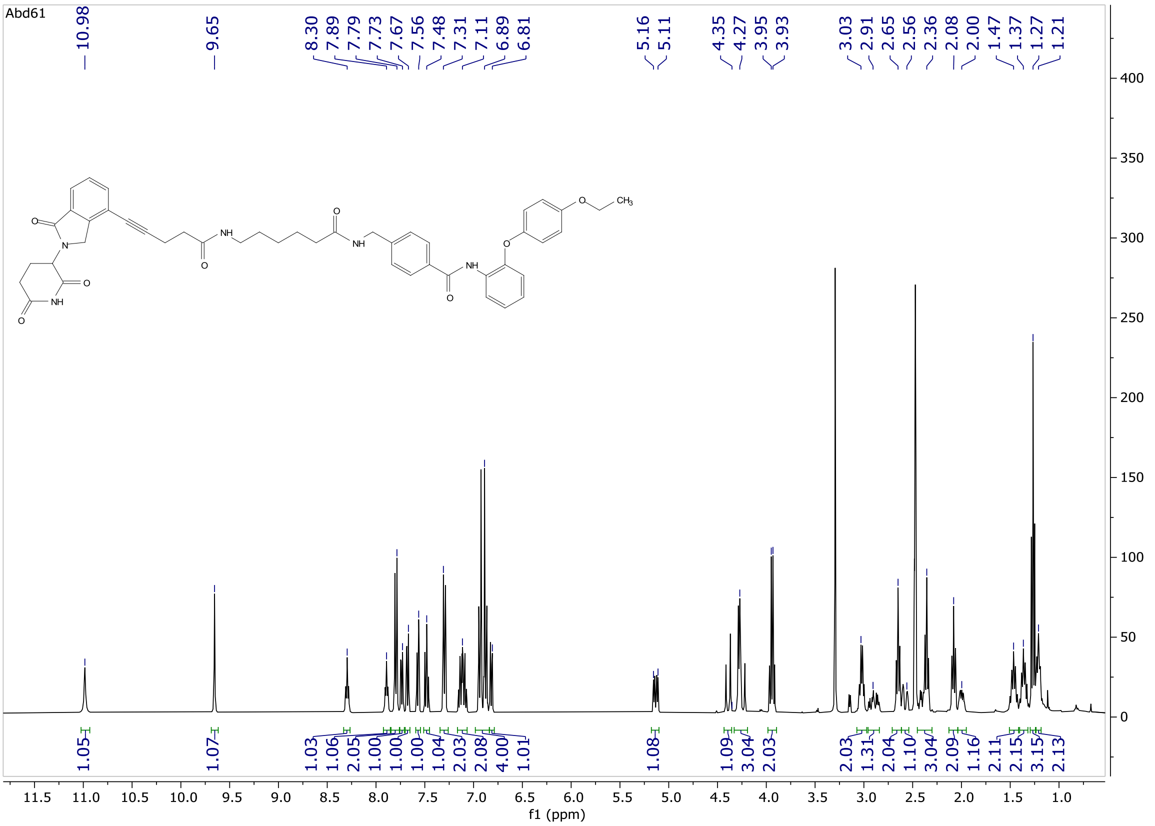
Abd61
10.98
9.65
8.30 7.89 7.79 7.73 7.67 7.56 7.48 7.31 7.11 6.89 6.81
5.16 5.11
4.35 4.27 3.95 3.93
3.03 2.91 2.65 2.56 2.36 2.08 2.00 1.47 1.37 1.27 1.21
1.05 1.07 1.03 1.06 2.05 1.00 1.00 1.00 1.04 2.03 2.08 4.00 1.01 1.08 1.09 3.04 2.03 2.03 1.31 2.04 1.10 3.04 2.09 1.16 2.11 2.15 3.15 2.13
O
N
NH
O
O
O
NH
O
NH
NH
O
O
O
CH3
f1 (ppm)

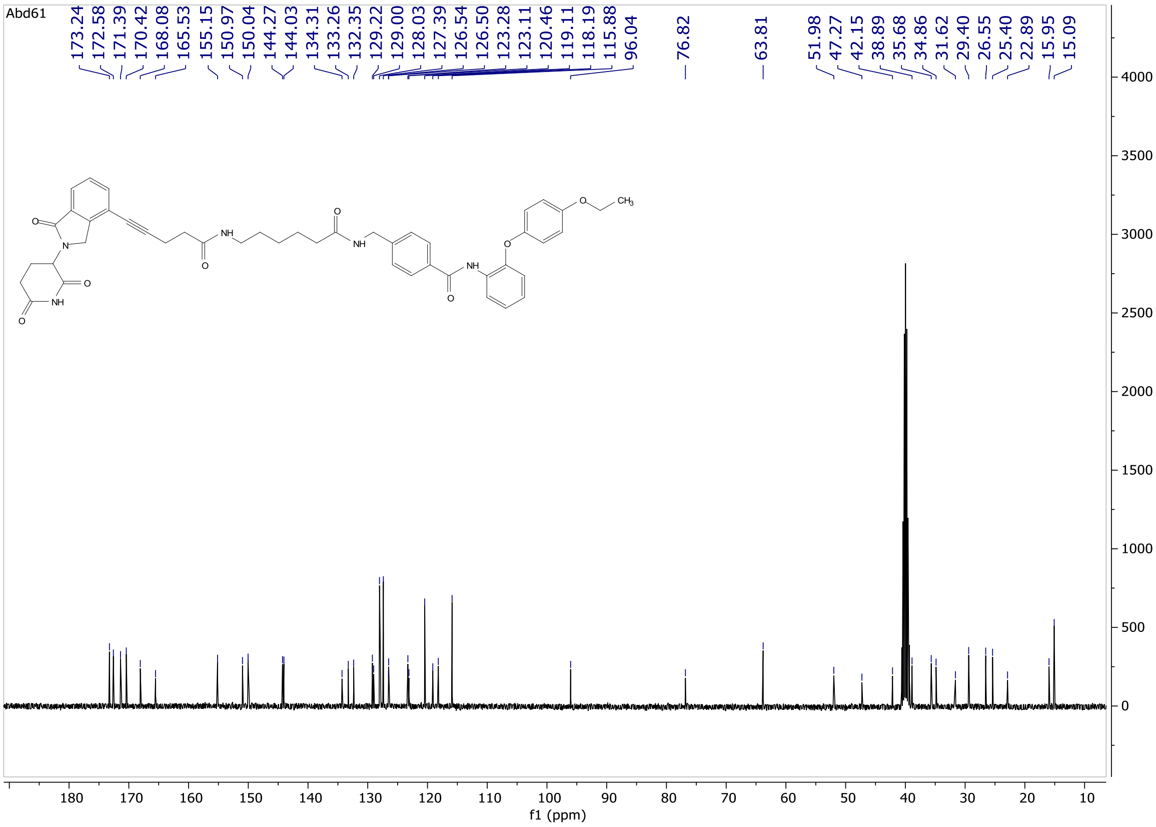
Abd61
173.24 172.58 171.39 170.42 168.08 165.53 155.15 150.97 150.04 144.27 144.03 134.31 133.26 132.35 129.22 129.00 128.03 127.39 126.54 126.50 123.28 123.11 120.46 119.11 118.19 115.88 96.04
76.82
63.81
51.98 47.27 42.15 38.89 35.68 34.86 31.62 29.40 26.55 25.40 22.89 15.95 15.09
O
N
NH
O
O
O
NH
O
NH
NH
O
O
O
CH3
f1 (ppm)

32e (Abd61)

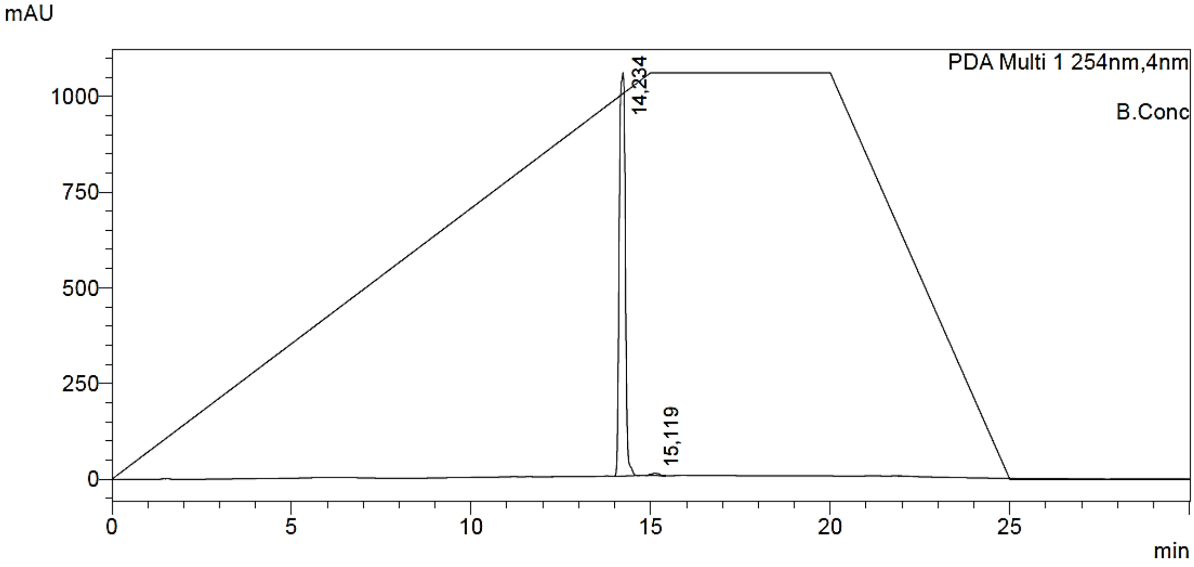

PDA Ch1 254nm

Peak#	Ret. Time	Area	Height	Area%
1	14,234	12261999	1052902	99,197
2	15,119	99253	6380	0,803
Total		12361252	1059282	100,000

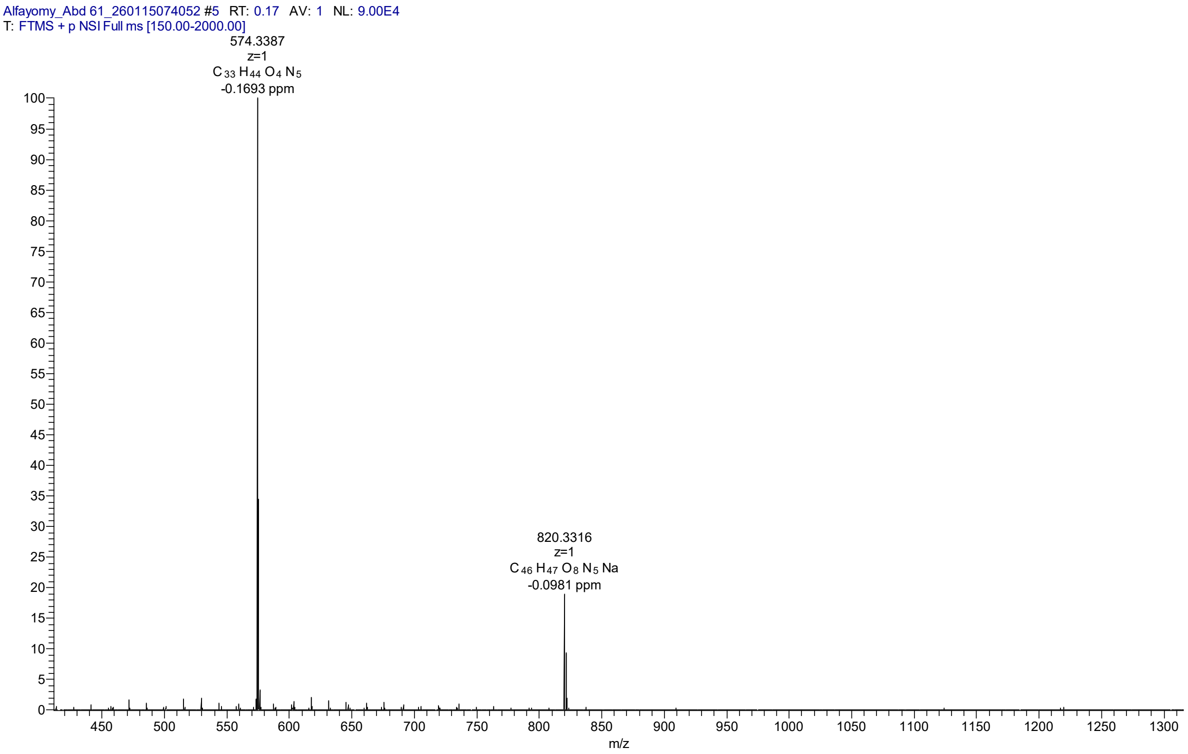

34a (Abd19)

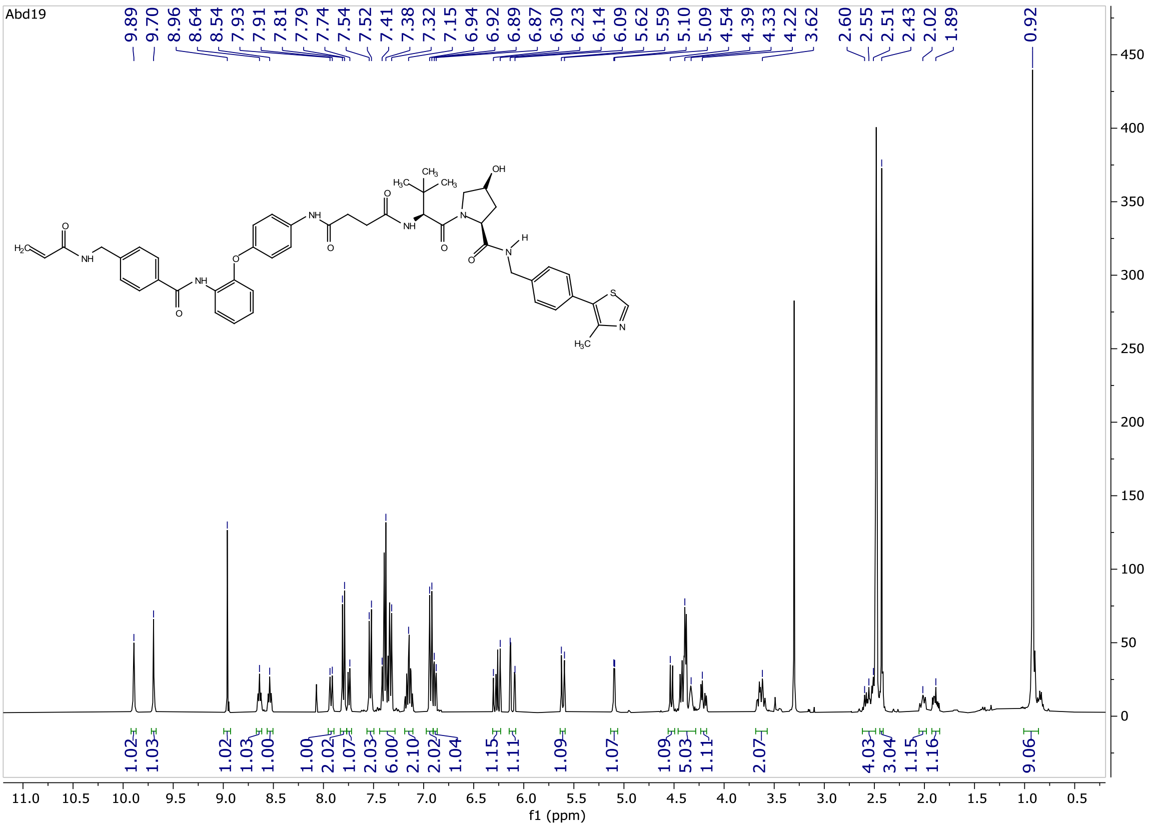
Abd19
9.89 9.70 8.96 8.64 8.54 7.93 7.91 7.81 7.79 7.74 7.54 7.52 7.41 7.38 7.32 7.15 6.94 6.92 6.89 6.87 6.30 6.23 6.14 6.09 5.62 5.59 5.10 5.09 4.54 4.39 4.33 4.22 3.62 2.60 2.55 2.51 2.43 2.02 1.89 0.92
1.02 1.03 1.02 1.03 1.00 1.00 2.02 1.07 2.03 6.00 2.10 2.02 1.04 1.15 1.11 1.09 1.07 1.09 5.03 1.11 2.07 4.03 3.04 1.15 1.16 9.06
f1 (ppm)

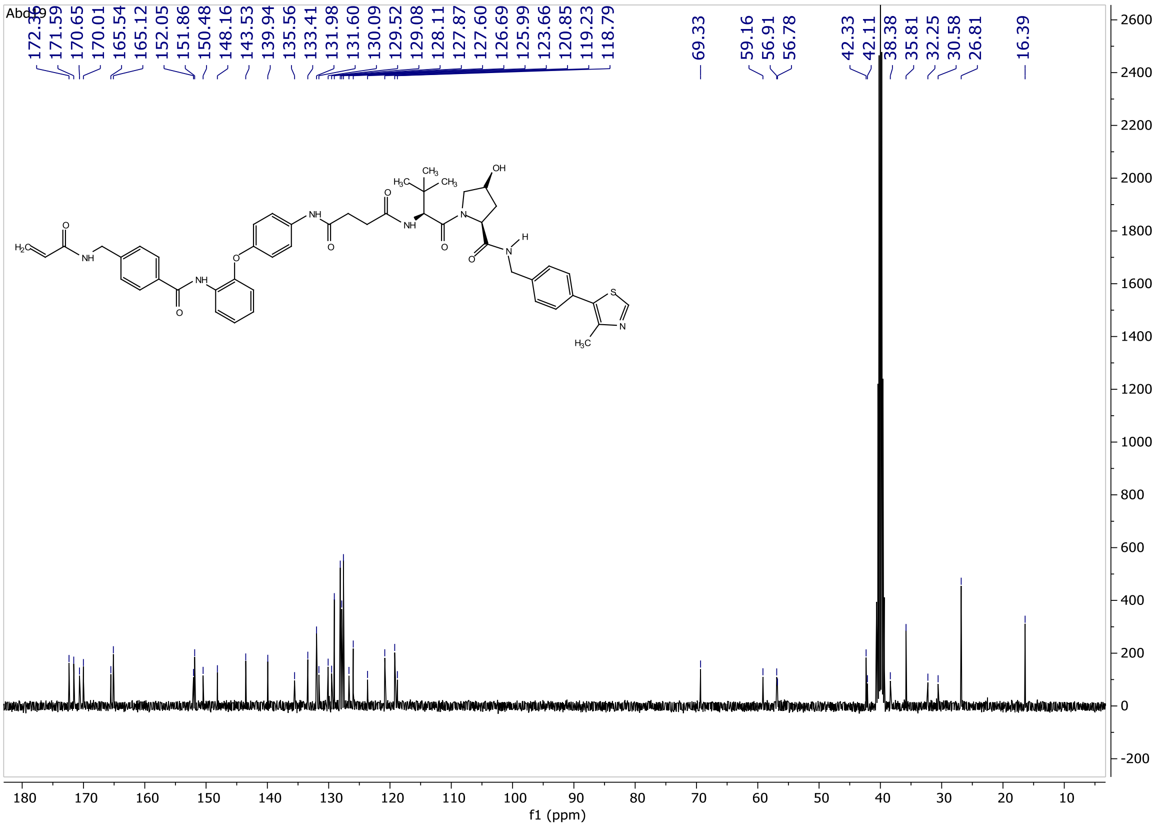
Abd19
172.3 171.59 170.65 170.01 165.54 165.12 152.05 151.86 150.48 148.16 143.53 139.94 135.56 133.41 131.98 131.60 130.09 129.52 129.08 128.11 127.87 127.60 126.69 125.99 123.66 120.85 119.23 118.79 69.33 59.16 56.91 56.78 42.33 42.11 38.38 35.81 32.25 30.58 26.81 16.39
f1 (ppm)

34a (Abd19)

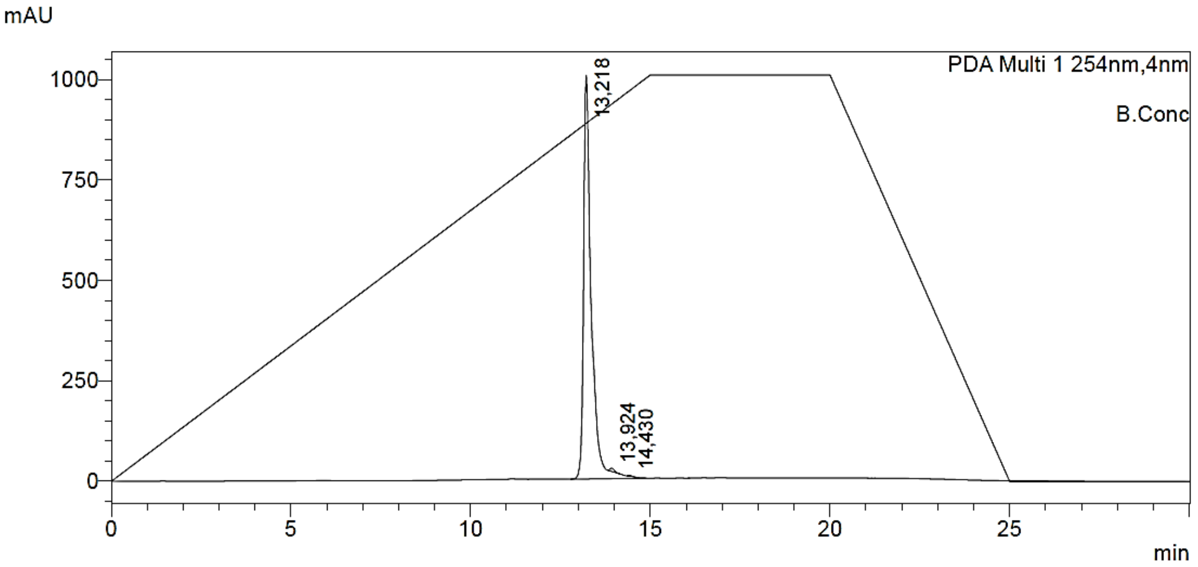

PDA Ch1 254nm

Peak#	Ret. Time	Area	Height	Area%
1	13,218	14967292	1004978	99,391
2	13,924	73965	8573	0,491
3	14,430	17693	2520	0,117
Total		15058949	1016071	100,000

Alfayomy_Abd 19_260114090833 #3-13 RT: 0.09-0.46 AV: 11 NL: 7.04E4
T: FTMS + p NSI Full ms [150.00-2000.00]

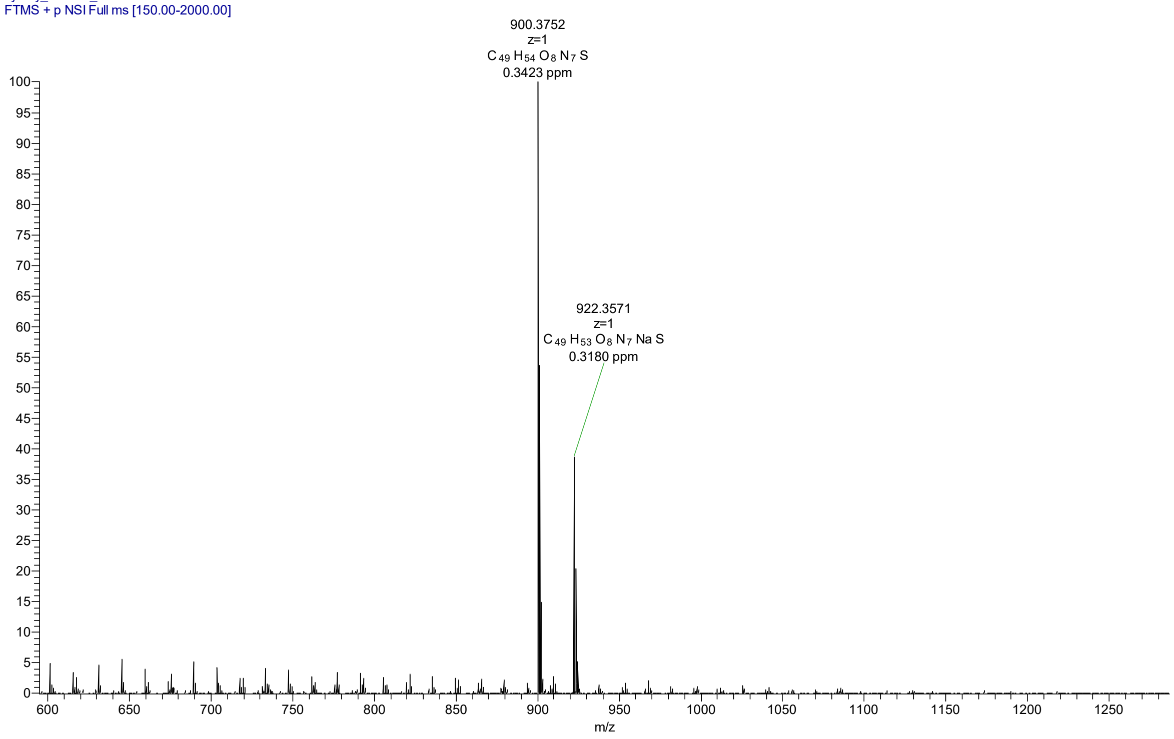

34b (Abd20)

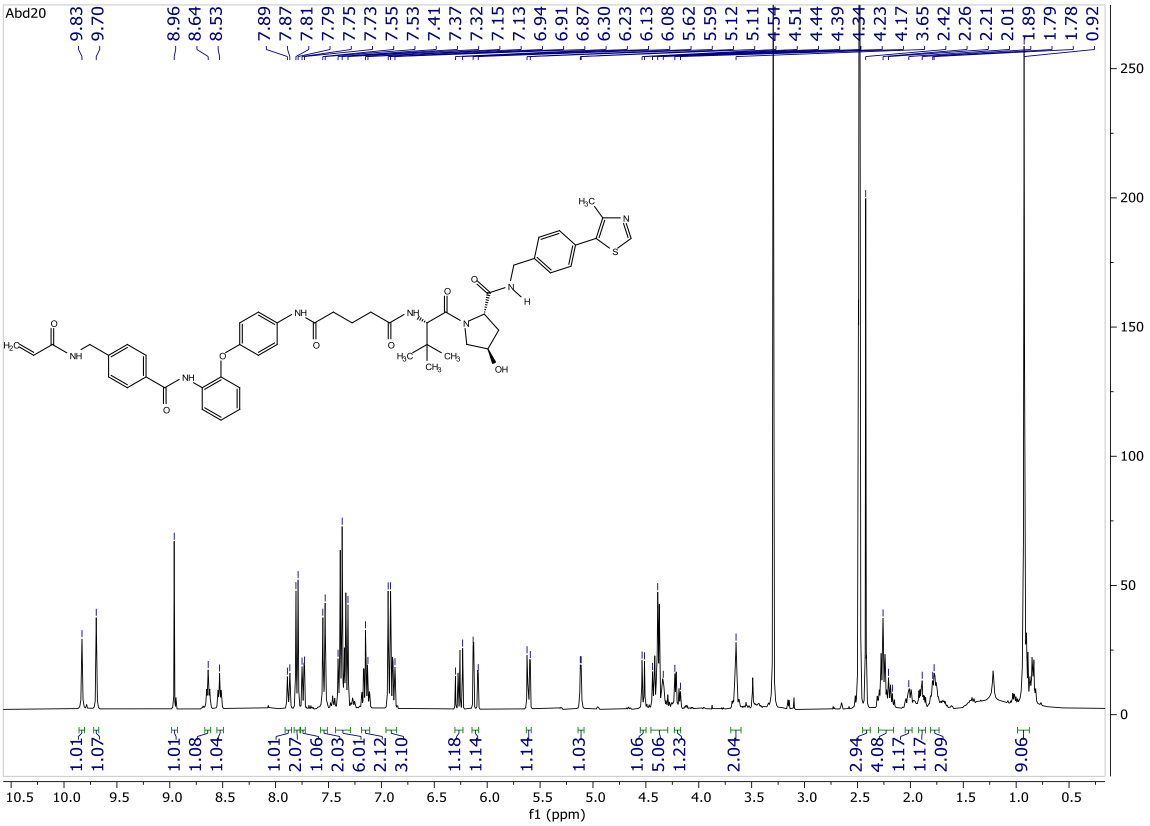

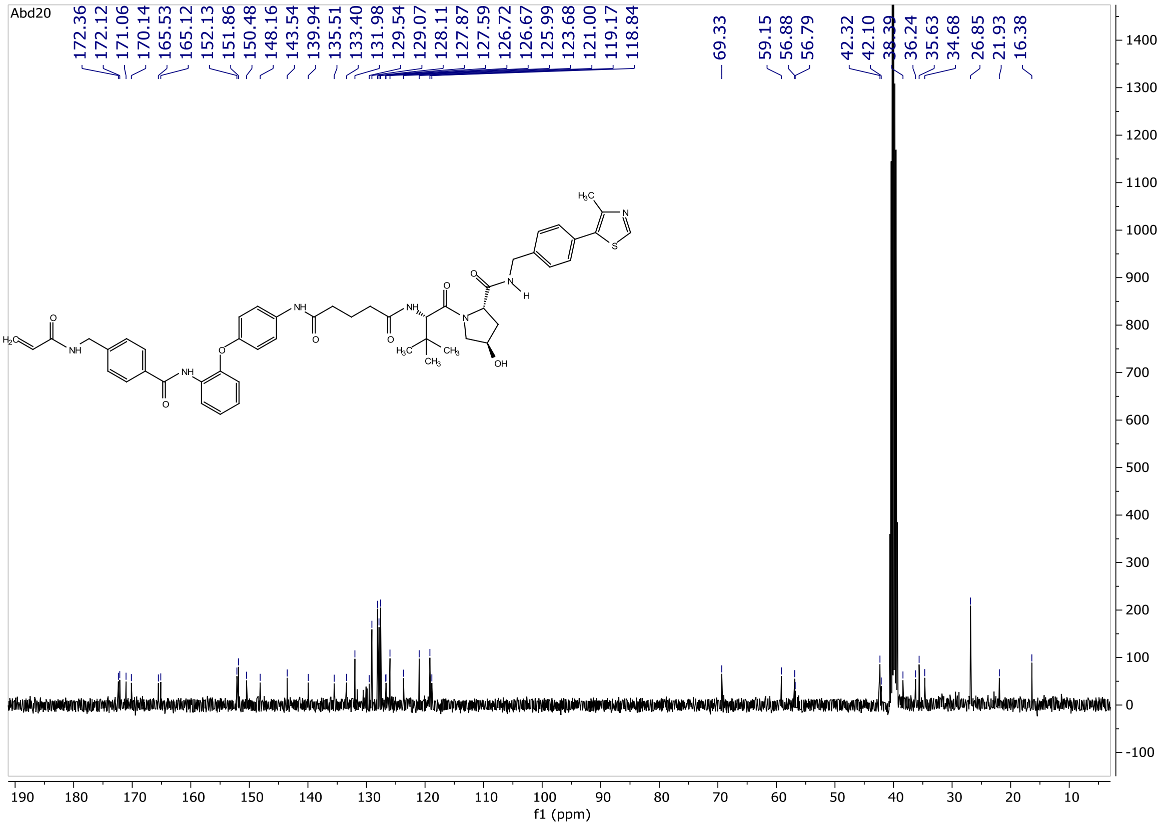

34b (Abd20)

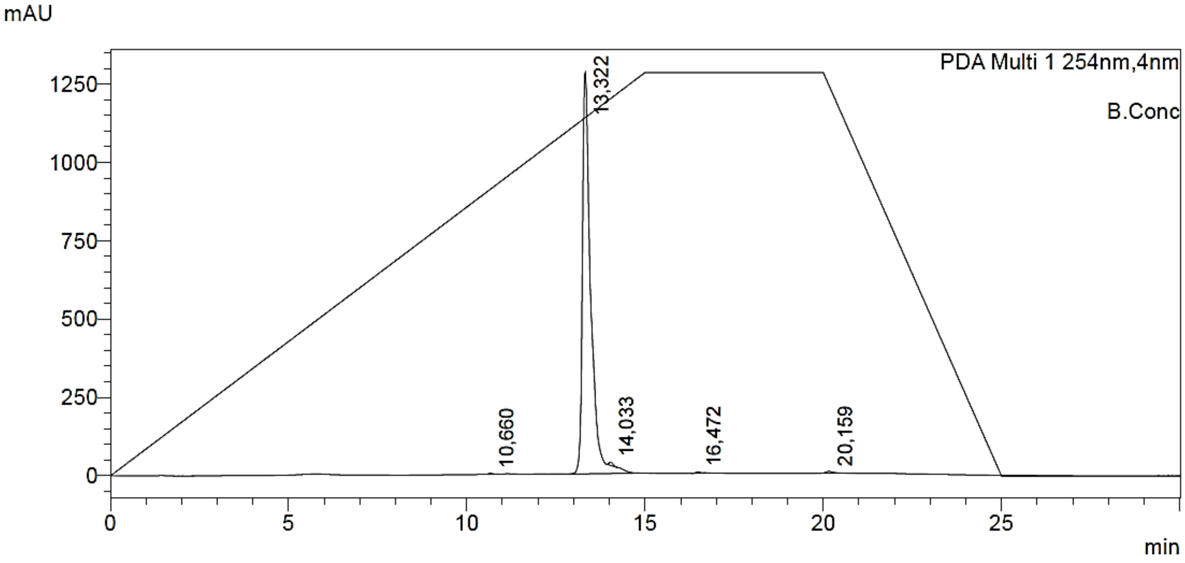

PDA Ch1 254nm

Peak#	Ret. Time	Area	Height	Area%
1	10,660	27488	2601	0,130
2	13,322	20881149	1279728	98,730
3	14,033	78961	9897	0,373
4	16,472	40248	4651	0,190
5	20,159	121868	6245	0,576
Total		21149715	1303121	100,000

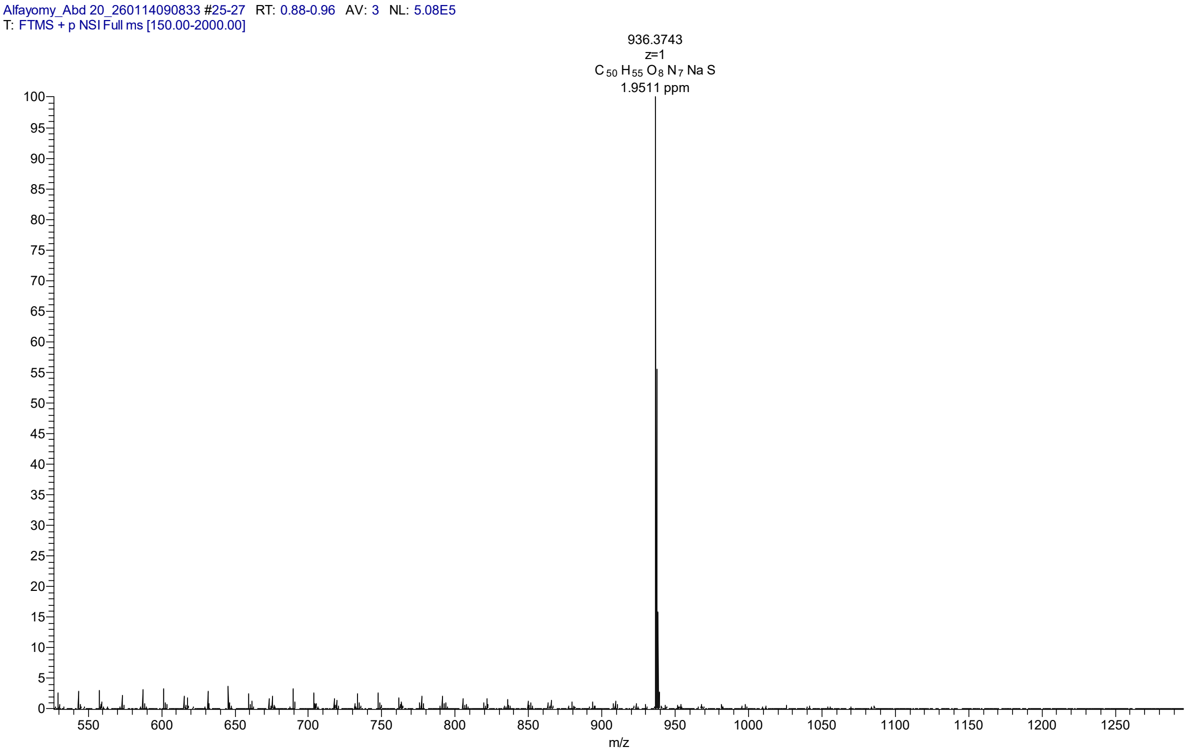

34c (Abd21)

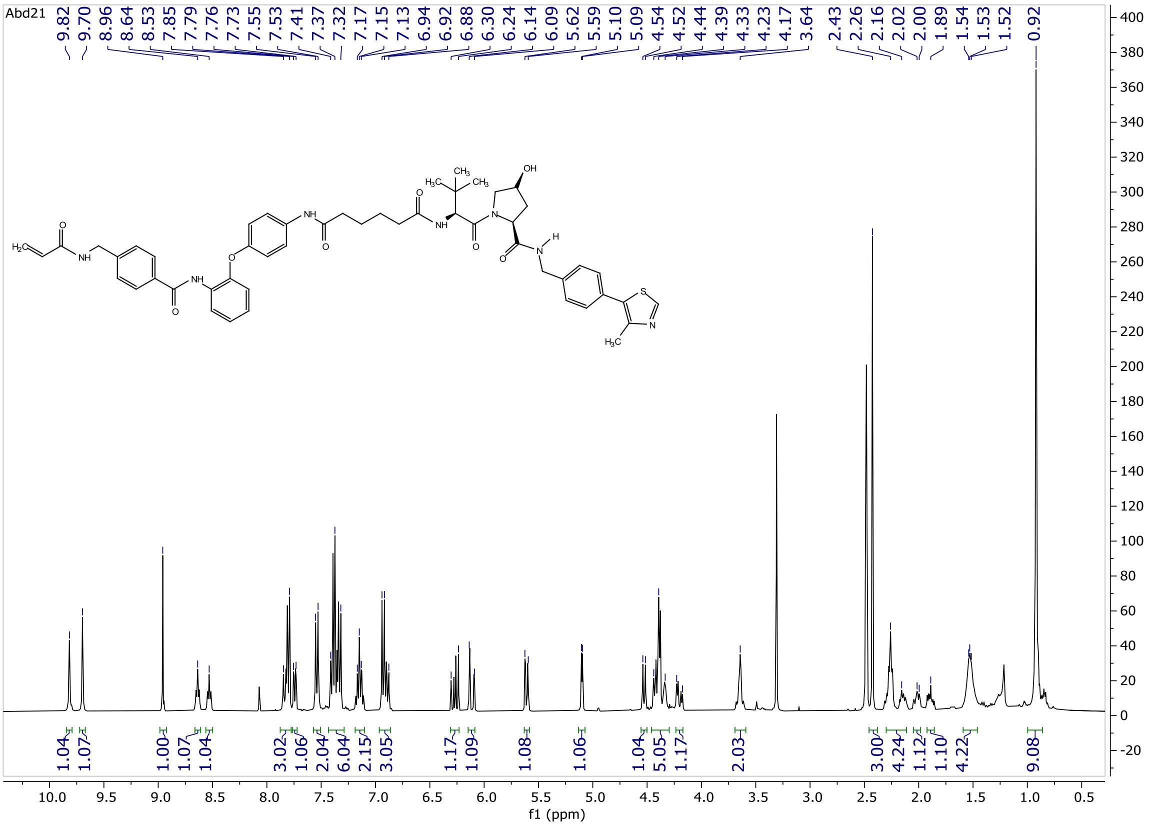

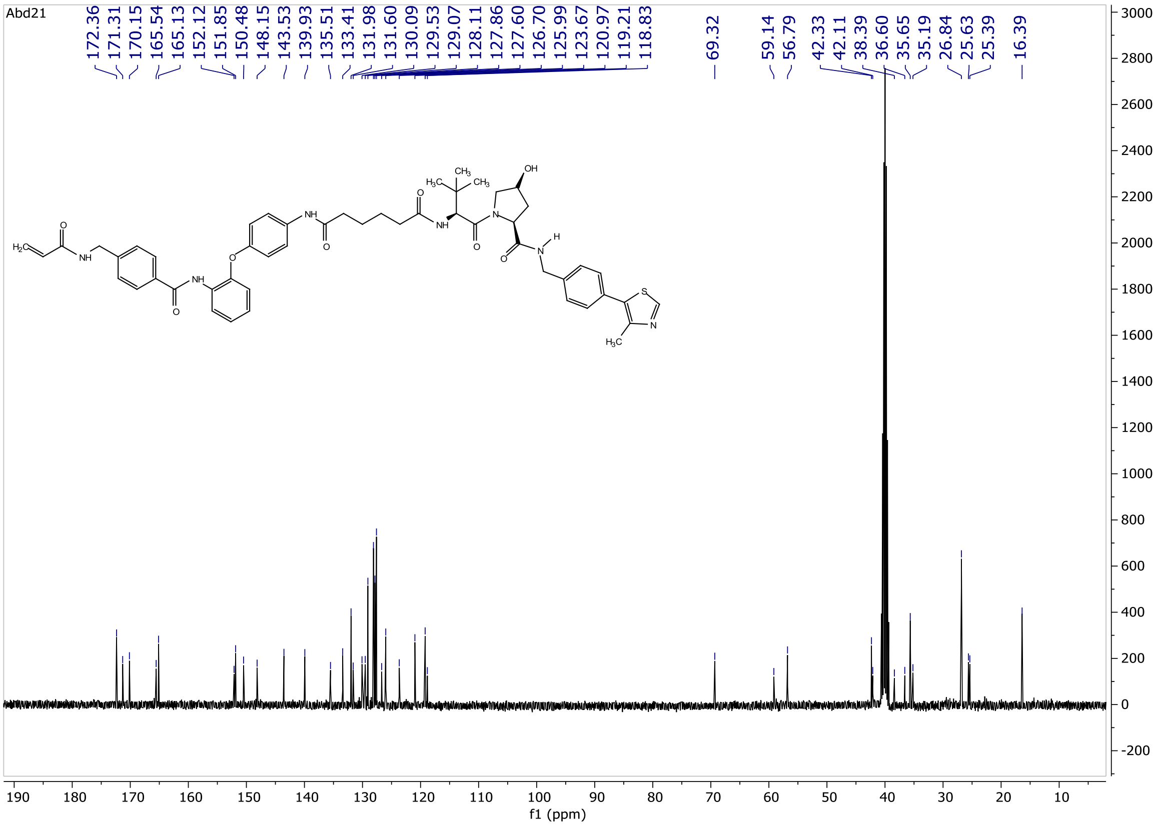

34c (Abd21)

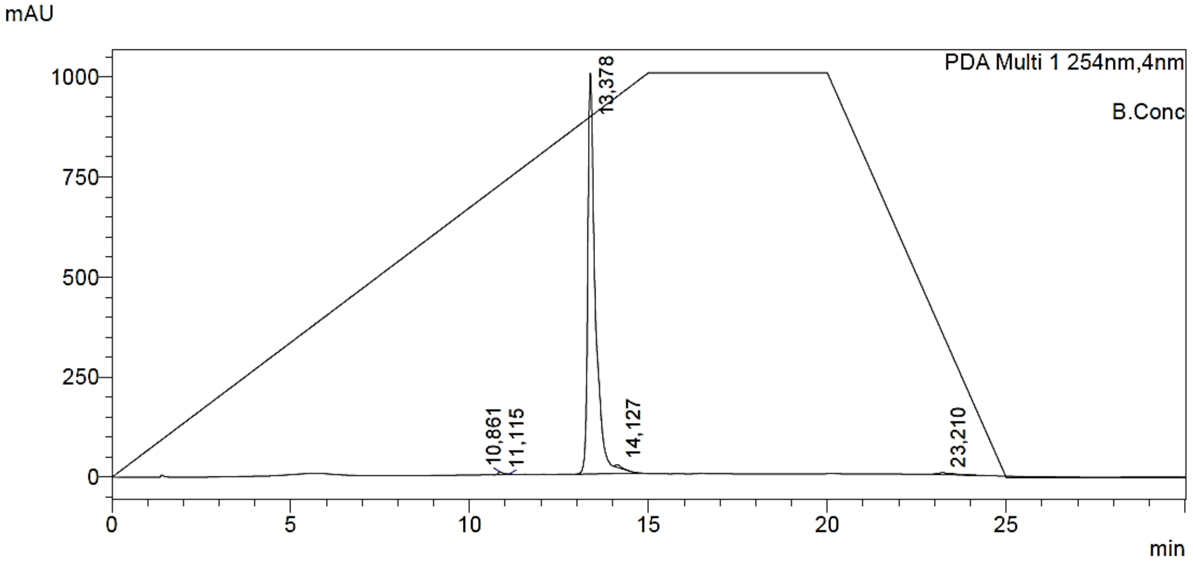

PDA Ch1 254nm

Peak#	Ret. Time	Area	Height	Area%
1	10,861	66907	6248	0,439
2	11,115	18545	1895	0,122
3	13,378	14953461	1001965	98,088
4	14,127	69135	6702	0,453
5	23,210	136833	4361	0,898
Total		15244881	1021170	100,000

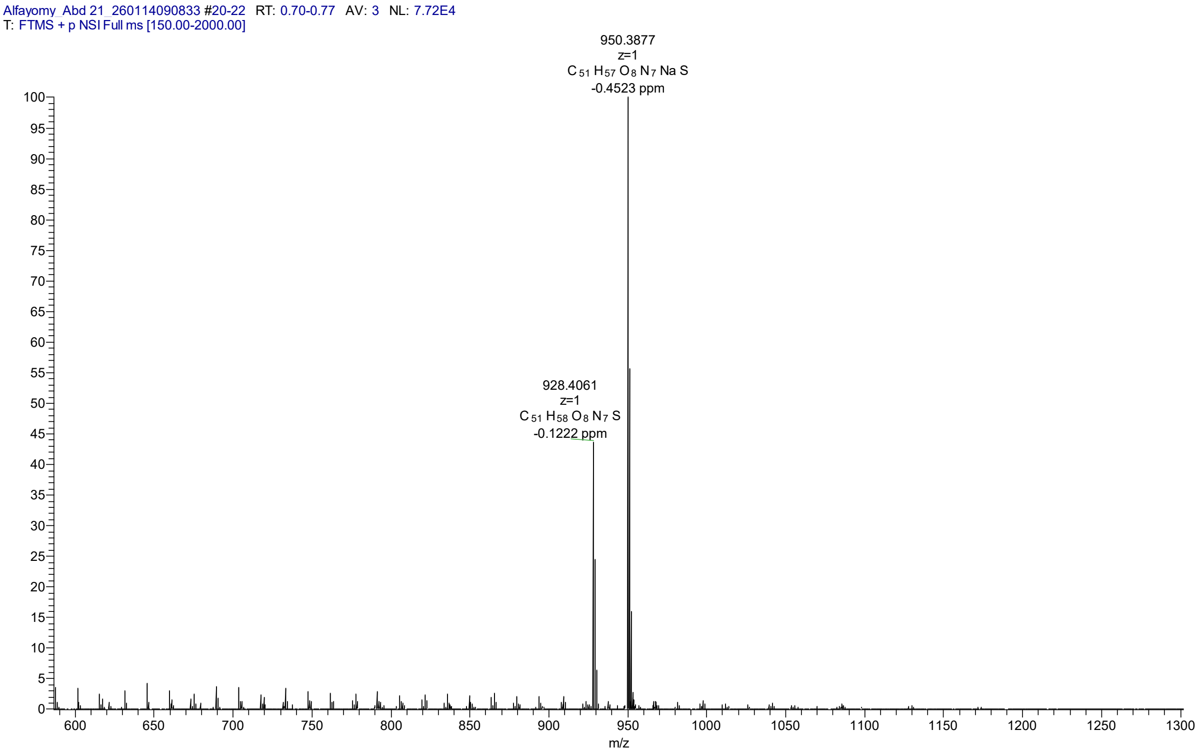

34d (Abd22)

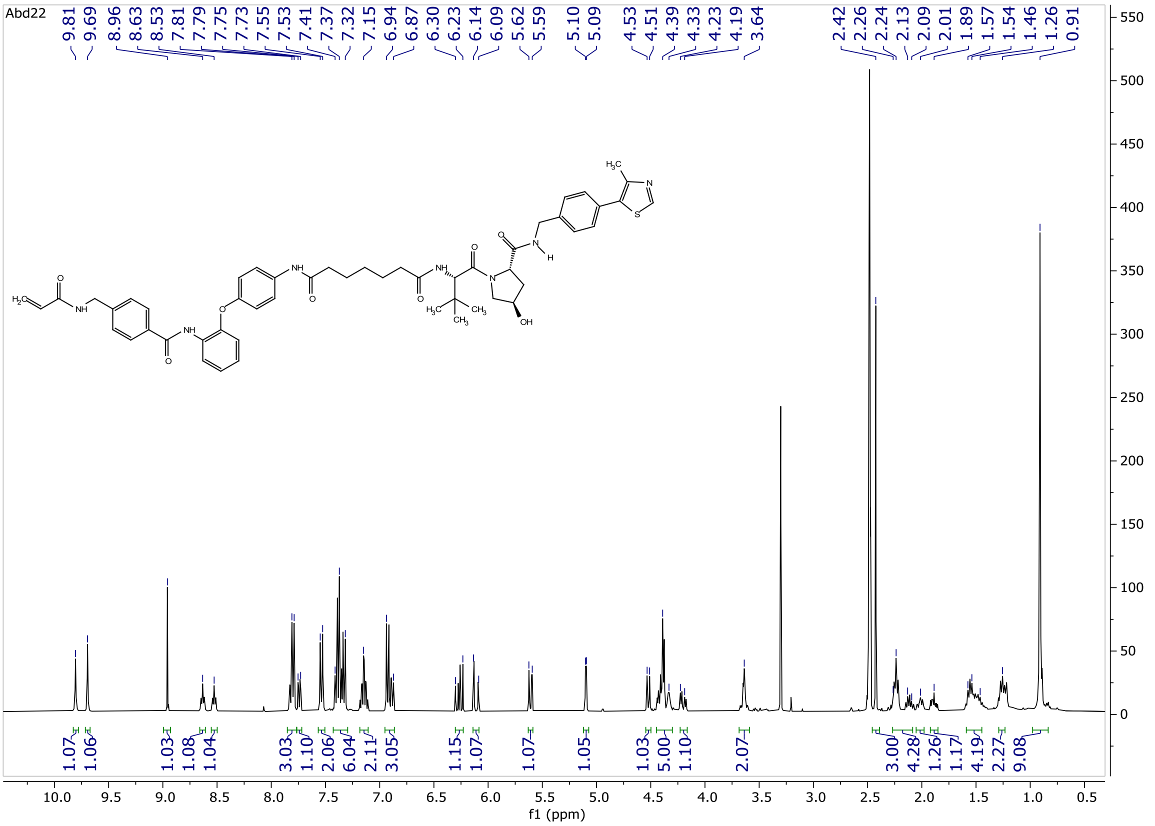
Abd22
9.81 9.69 8.96 8.63 8.53 7.81 7.79 7.75 7.73 7.55 7.53 7.41 7.37 7.32 7.15 6.94 6.87 6.30 6.23 6.14 6.09 5.62 5.59 5.10 5.09 4.53 4.51 4.39 4.33 4.23 4.19 3.64 2.42 2.26 2.24 2.13 2.09 2.01 1.89 1.57 1.54 1.46 1.26 0.91
1.07 1.06 1.03 1.08 1.04 3.03 1.10 2.06 6.04 2.11 3.05 1.15 1.07 1.07 1.05 1.03 5.00 1.10 2.07 3.00 4.28 1.26 1.17 4.19 2.27 9.08
f1 (ppm)

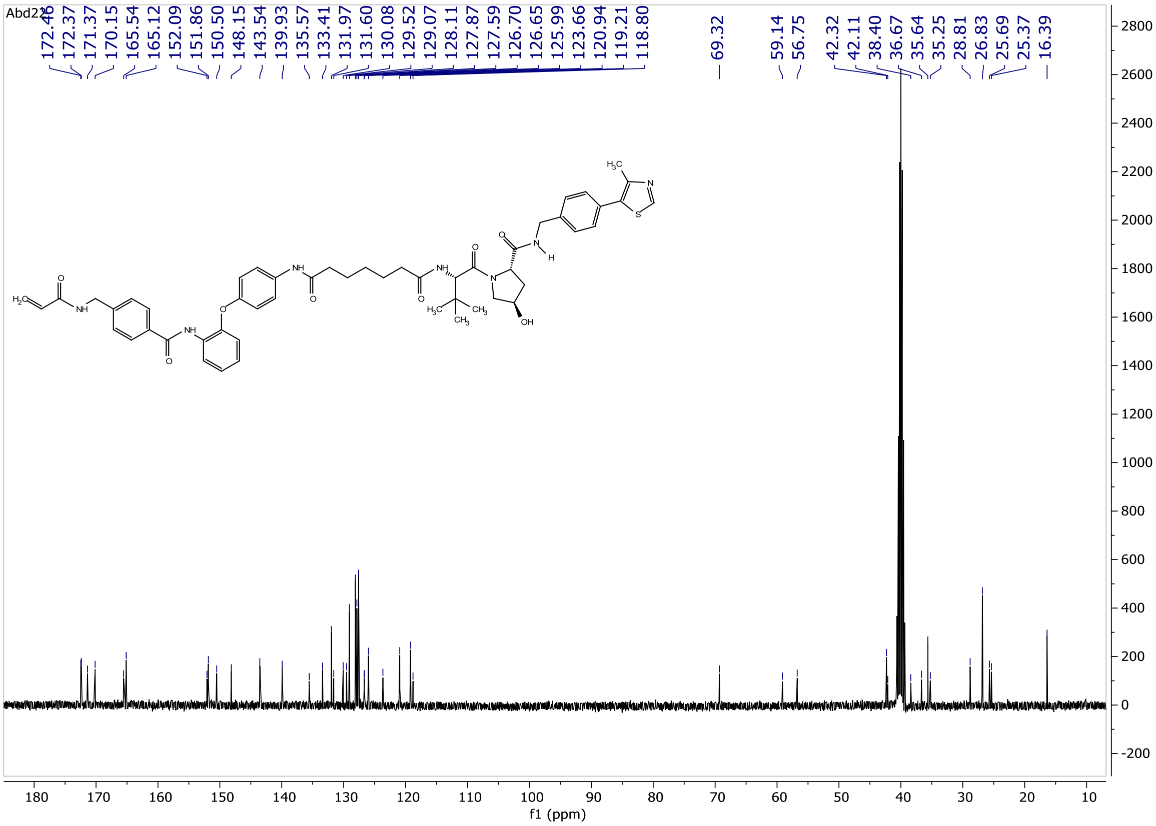
Abd22
172.46 172.37 171.37 170.15 165.54 165.12 152.09 151.86 150.50 148.15 143.54 139.93 135.57 133.41 131.97 131.60 130.08 129.52 129.07 128.11 127.87 127.59 126.70 126.65 125.99 123.66 120.94 119.21 118.80 69.32 59.14 56.75 42.32 42.11 38.40 36.67 35.64 35.25 28.81 26.83 25.69 25.37 16.39
f1 (ppm)

34d (Abd22)

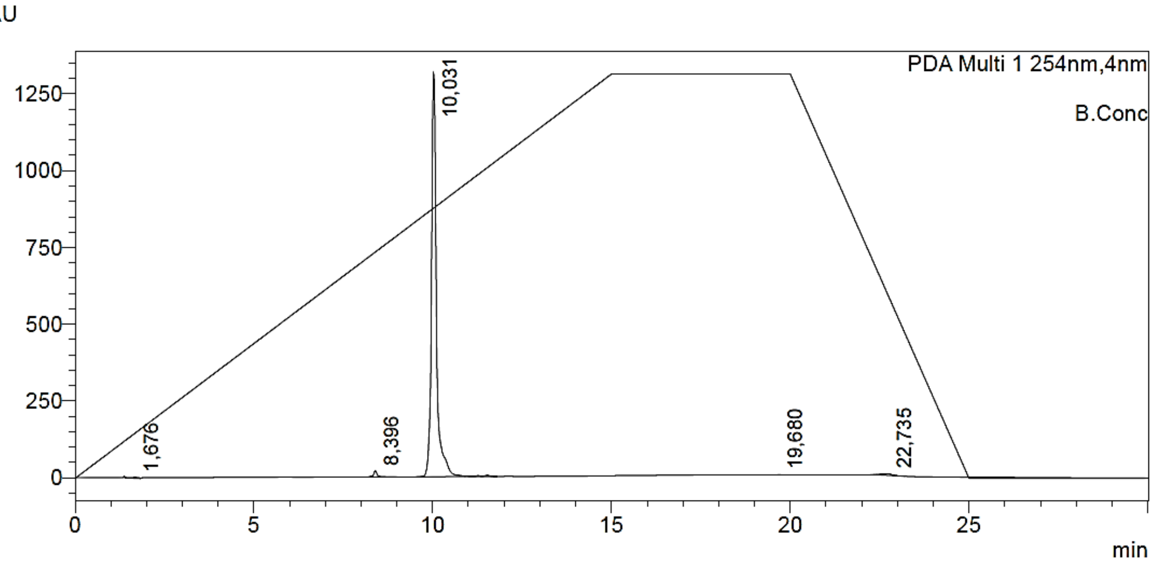

PDA Ch1 254nm

Peak#	Ret. Time	Area	Height	Area%
1	1,676	48945	2597	0,374
2	8,396	136553	20342	1,043
3	10,031	12797558	1311531	97,714
4	19,680	8186	899	0,063
5	22,735	105710	5079	0,807
Total		13096952	1340448	100,000

Alfayomy_Abd 22_260114092442 #3-5 RT: 0.09-0.17 AV: 3 NL: 8.44E4
T: FTMS + p NSI Full ms [150.00-2000.00]

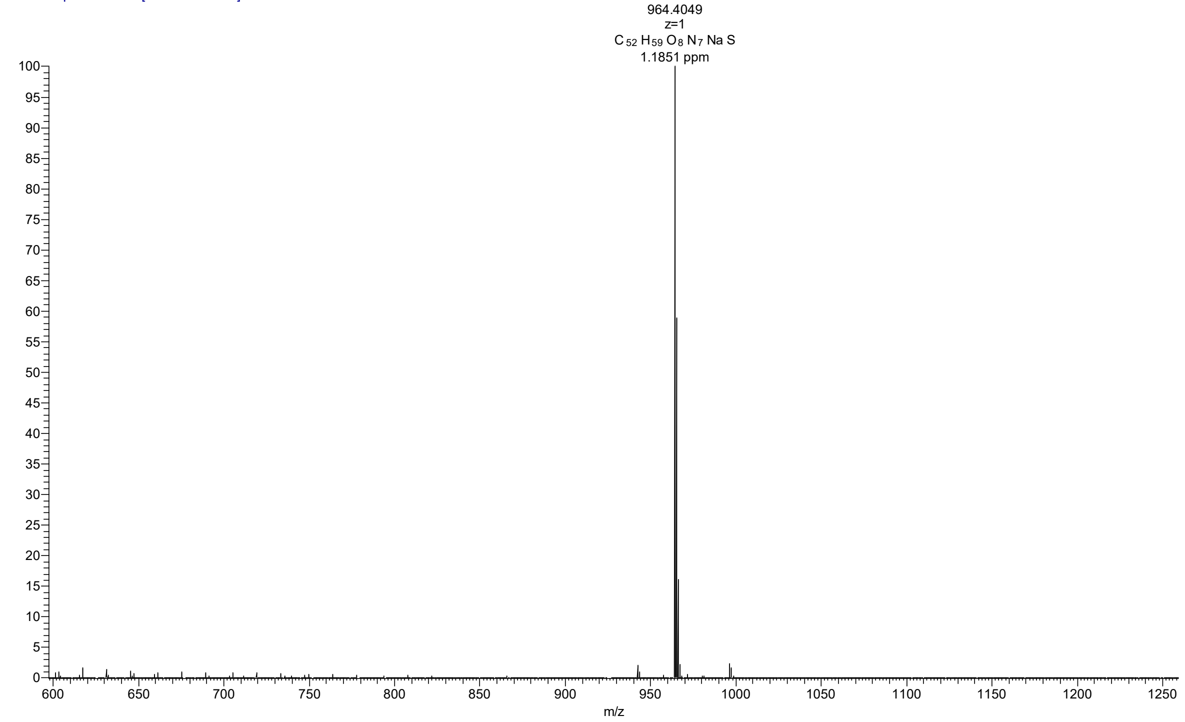

34e (Abd18)

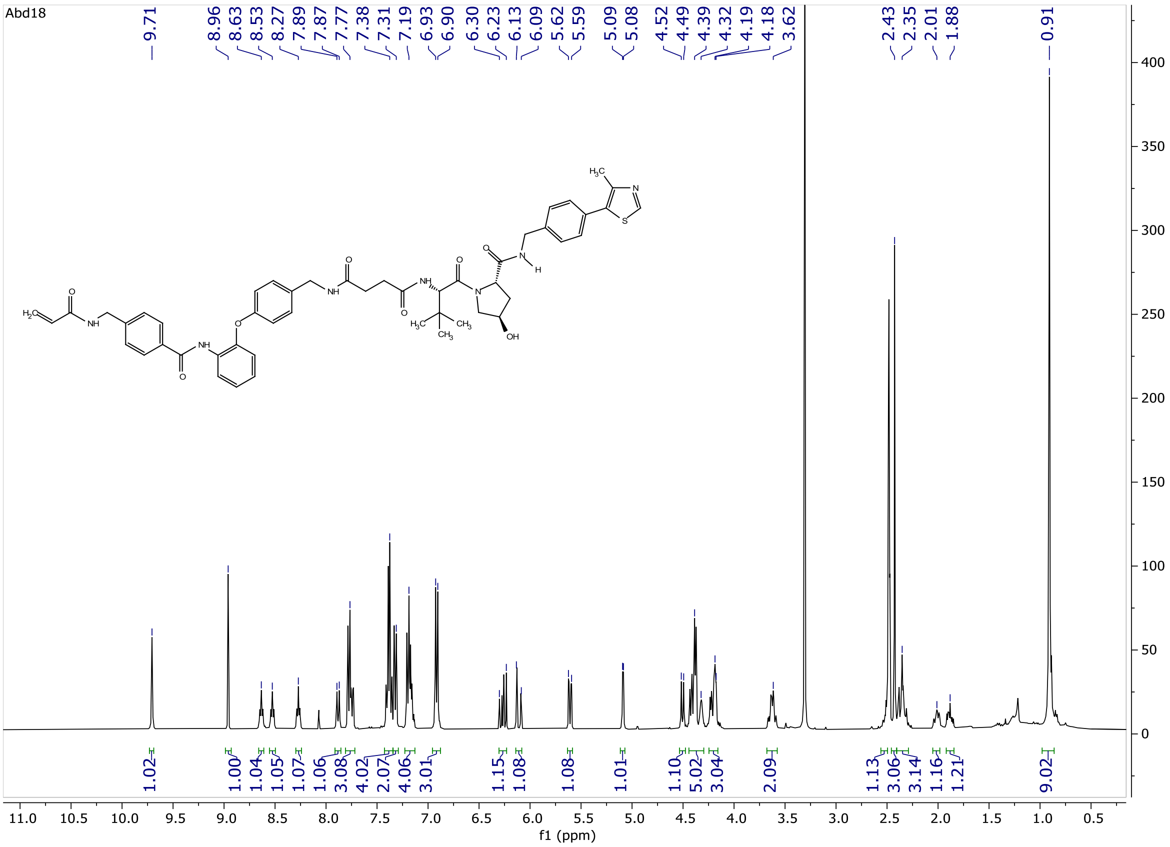
Abd18
9.71 8.96 8.63 8.53 8.27 7.89 7.87 7.77 7.38 7.31 7.19 6.93 6.90 6.30 6.23 6.13 6.09 5.62 5.59 5.09 5.08 4.52 4.49 4.39 4.32 4.19 4.18 3.62 2.43 2.35 2.01 1.88 0.91
1.02 1.00 1.04 1.05 1.07 1.06 3.08 4.02 2.07 4.06 3.01 1.15 1.08 1.08 1.01 1.10 5.02 3.04 2.09 1.13 3.06 3.14 1.16 1.21 9.02
f1 (ppm)

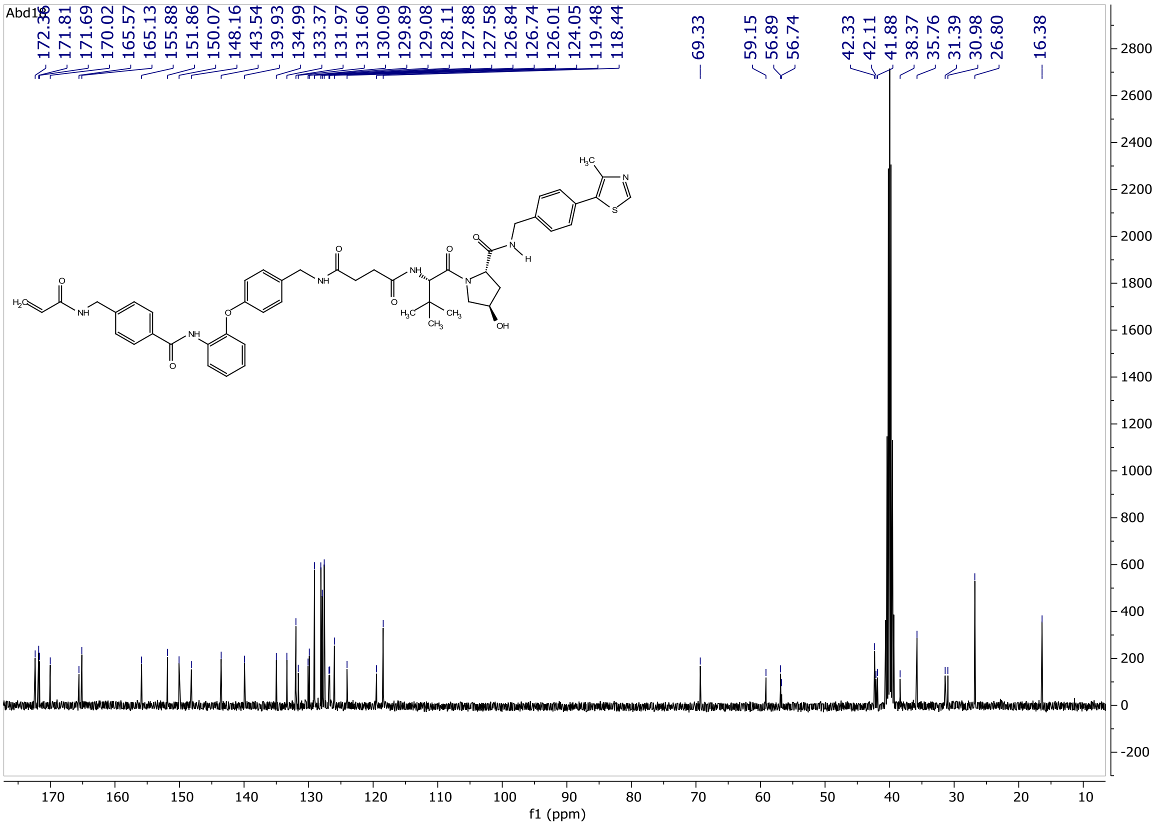
Abd18
172.36 171.81 171.69 170.02 165.57 165.13 155.88 151.86 150.07 148.16 143.54 139.93 134.99 133.37 131.97 131.60 130.09 129.89 129.08 128.11 127.88 127.58 126.84 126.74 126.01 124.05 119.48 118.44 69.33 59.15 56.89 56.74 42.33 42.11 41.88 38.37 35.76 31.39 30.98 26.80 16.38
f1 (ppm)

34e (Abd18)

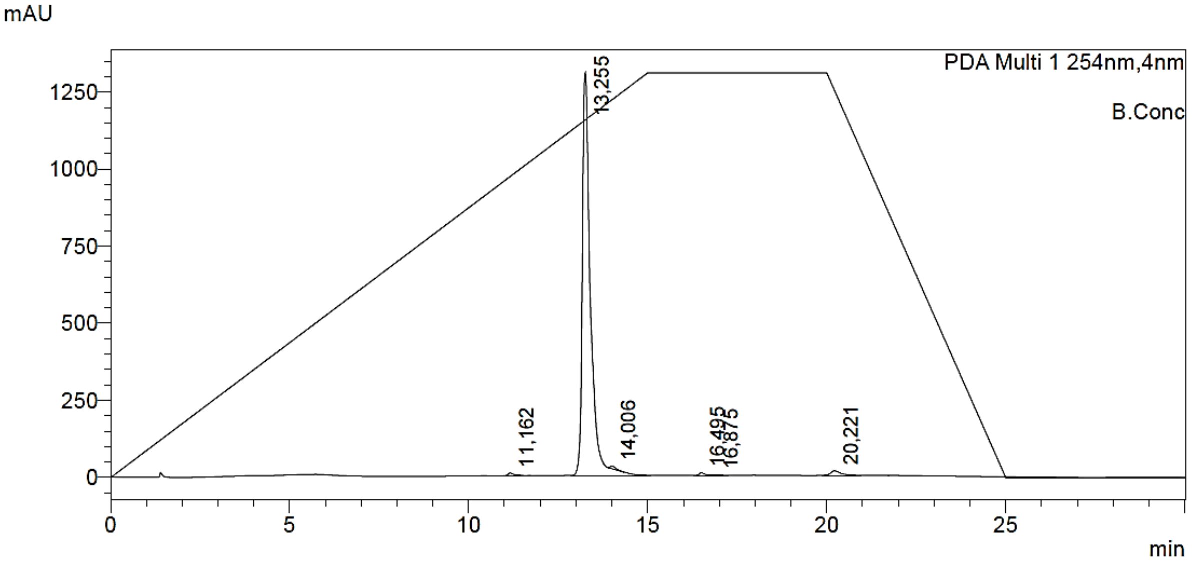

PDA Ch1 254nm

Peak#	Ret. Time	Area	Height	Area%
1	11,162	130375	8970	0,611
2	13,255	20680550	1306101	96,943
3	14,006	85965	8344	0,403
4	16,495	105364	9299	0,494
5	16,875	23164	1836	0,109
6	20,221	307170	15015	1,440
Total		21332588	1349564	100,000

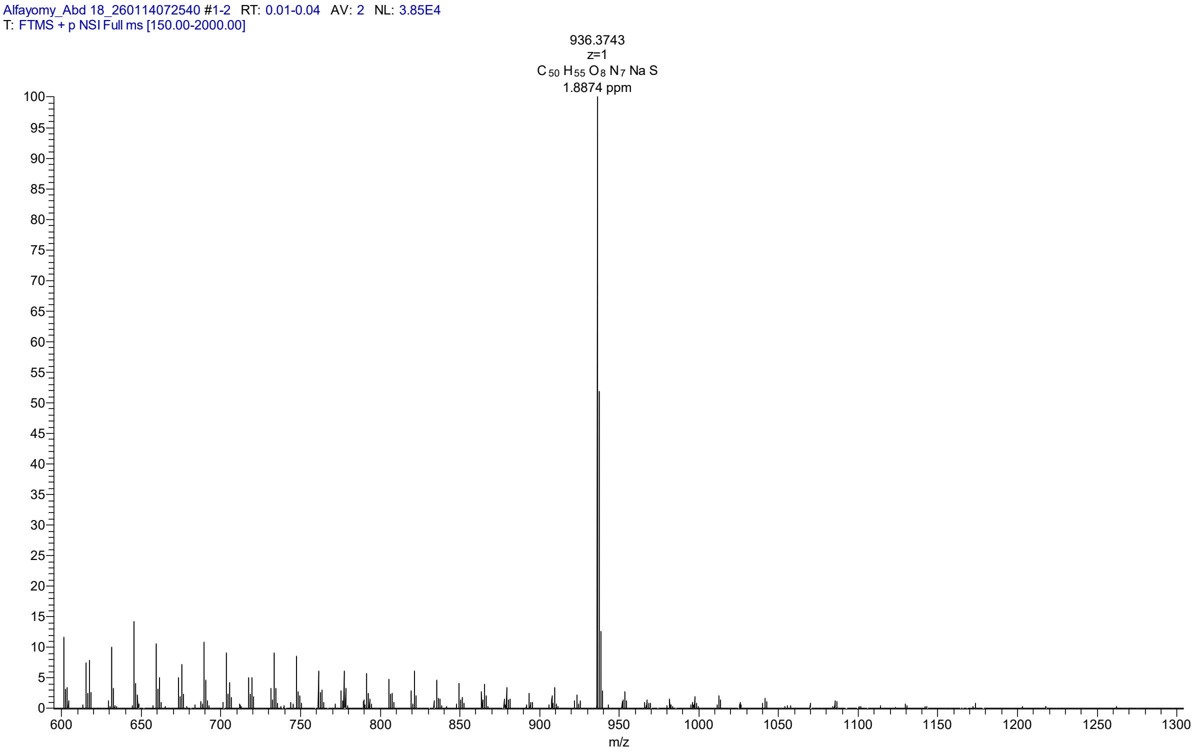

34f (Abd2)

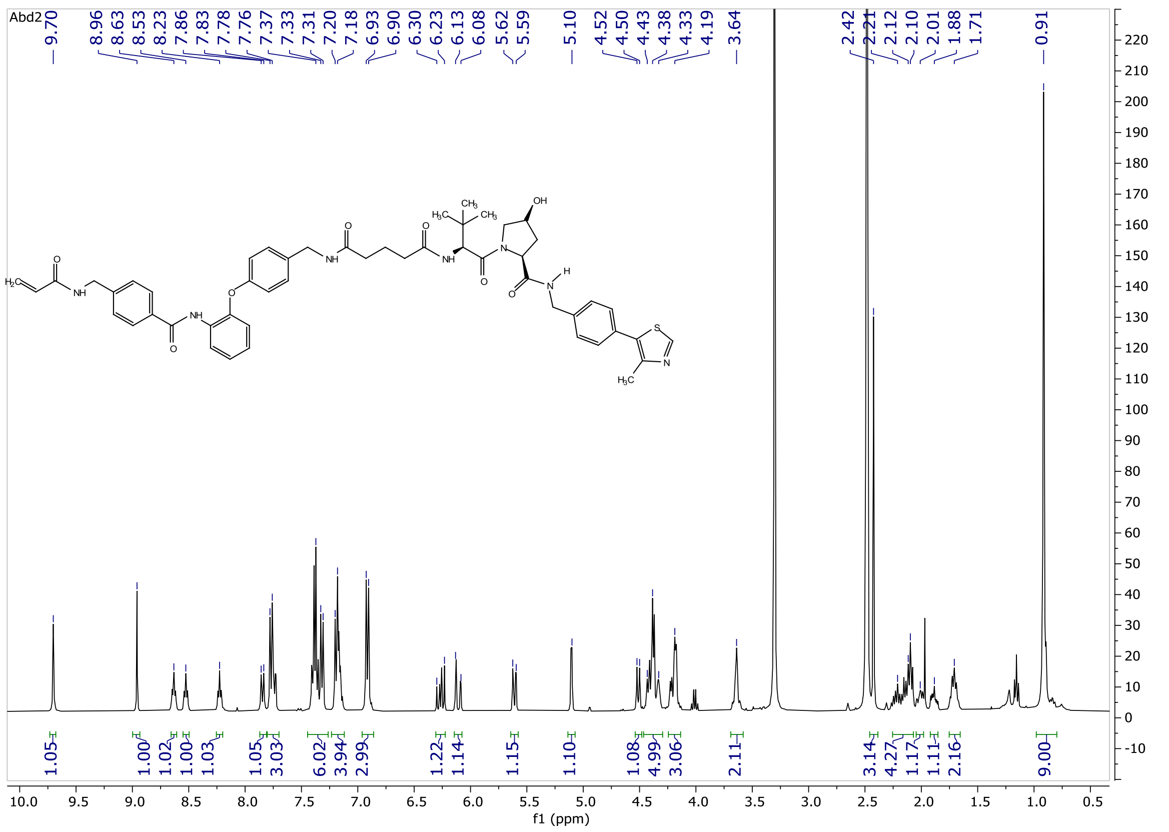

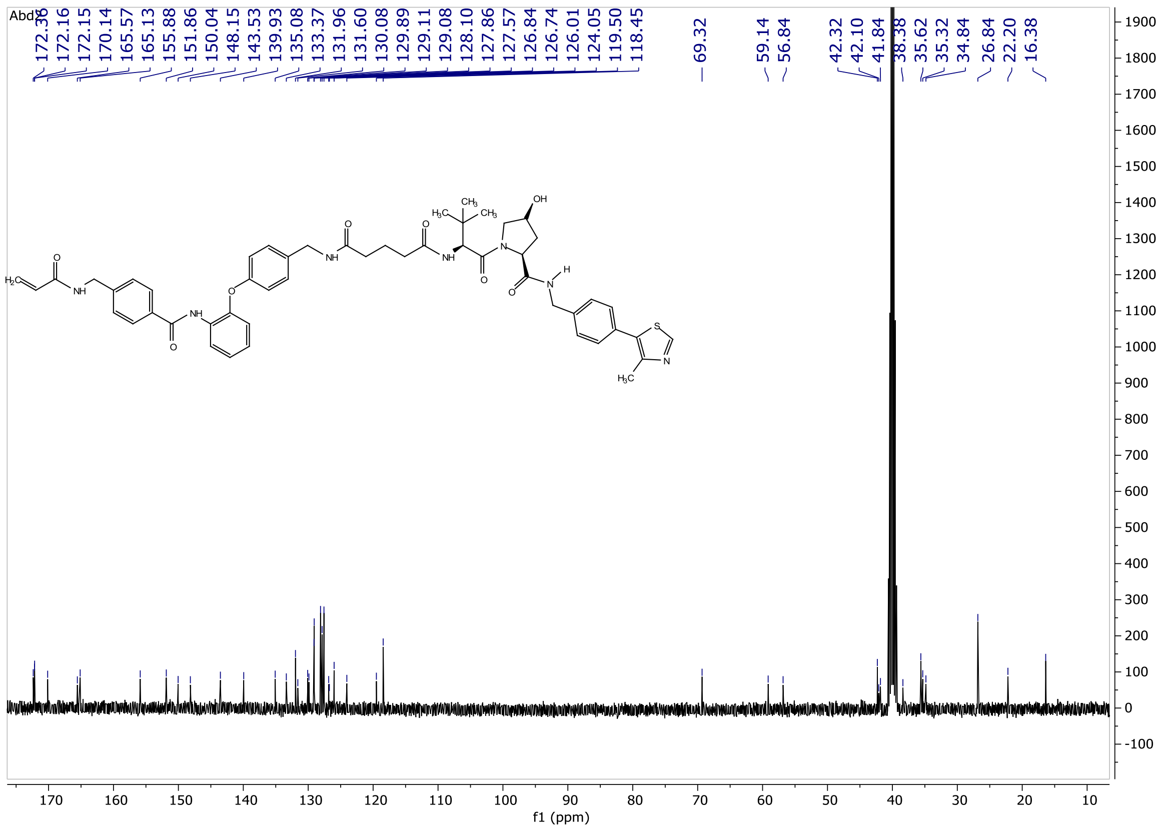

34f (Abd2)

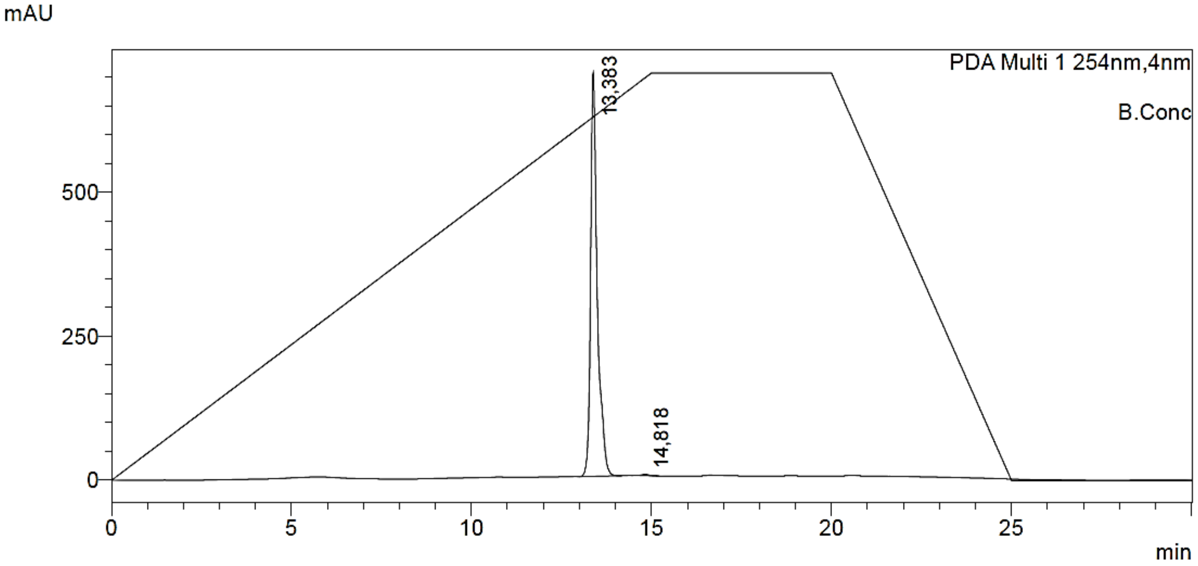

PDA Ch1 254nm

Peak#	Ret. Time	Area	Height	Area%
1	13,383	9099602	700363	99,700
2	14,818	27370	2367	0,300
Total		9126972	702730	100,000

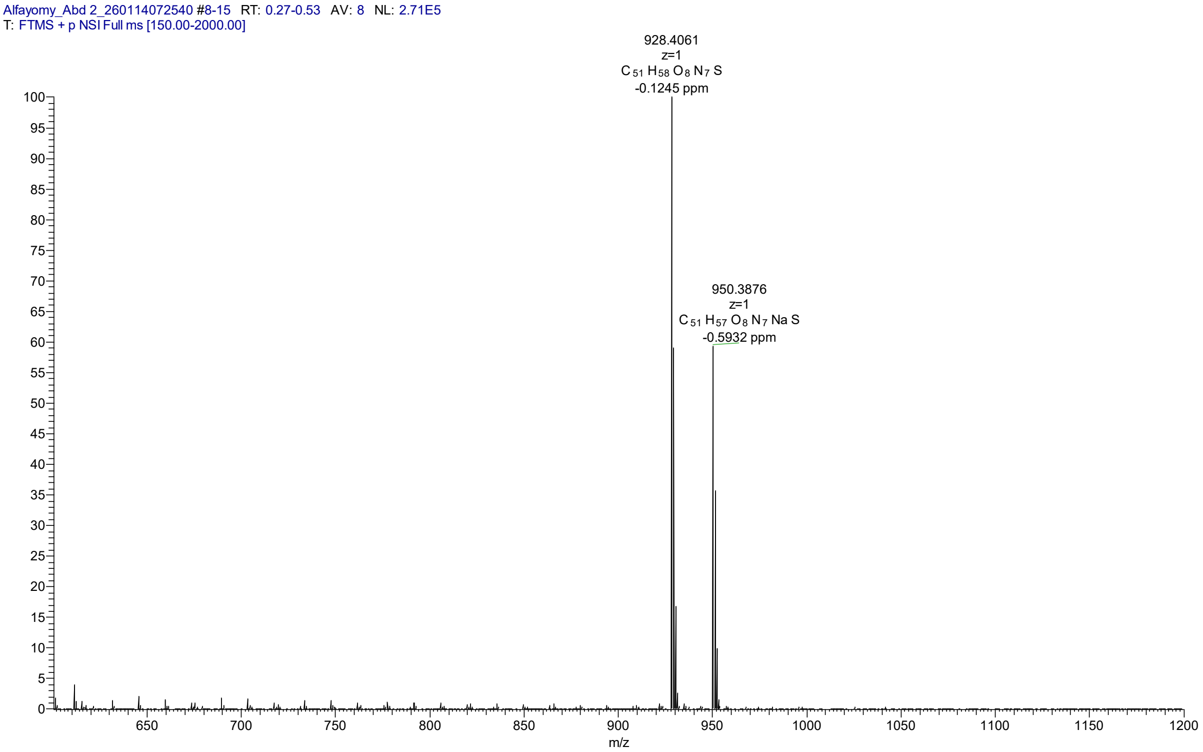

34g (Abd5)

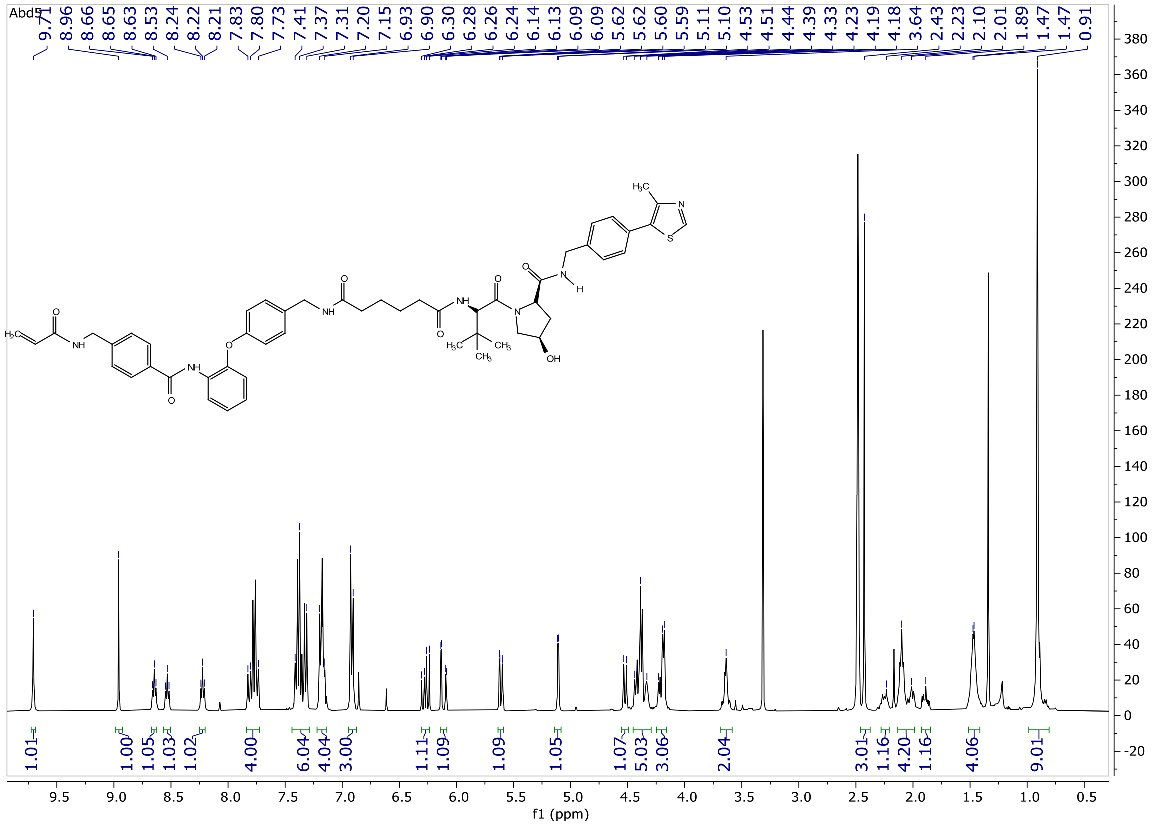
Abd5
9.71 8.96 8.66 8.65 8.63 8.53 8.24 8.22 8.21 7.83 7.80 7.73 7.41 7.37 7.31 7.20 7.15 6.93 6.90 6.30 6.28 6.26 6.24 6.14 6.13 6.09 6.09 5.62 5.62 5.60 5.59 5.11 5.10 4.53 4.51 4.44 4.39 4.33 4.23 4.19 4.18 3.64 2.43 2.23 2.10 2.01 1.89 1.47 1.47 0.91
1.01 1.00 1.05 1.03 1.02 4.00 6.04 4.04 3.00 1.11 1.09 1.09 1.05 1.07 5.03 3.06 2.04 3.01 1.16 4.20 1.16 4.06 9.01
f1 (ppm)

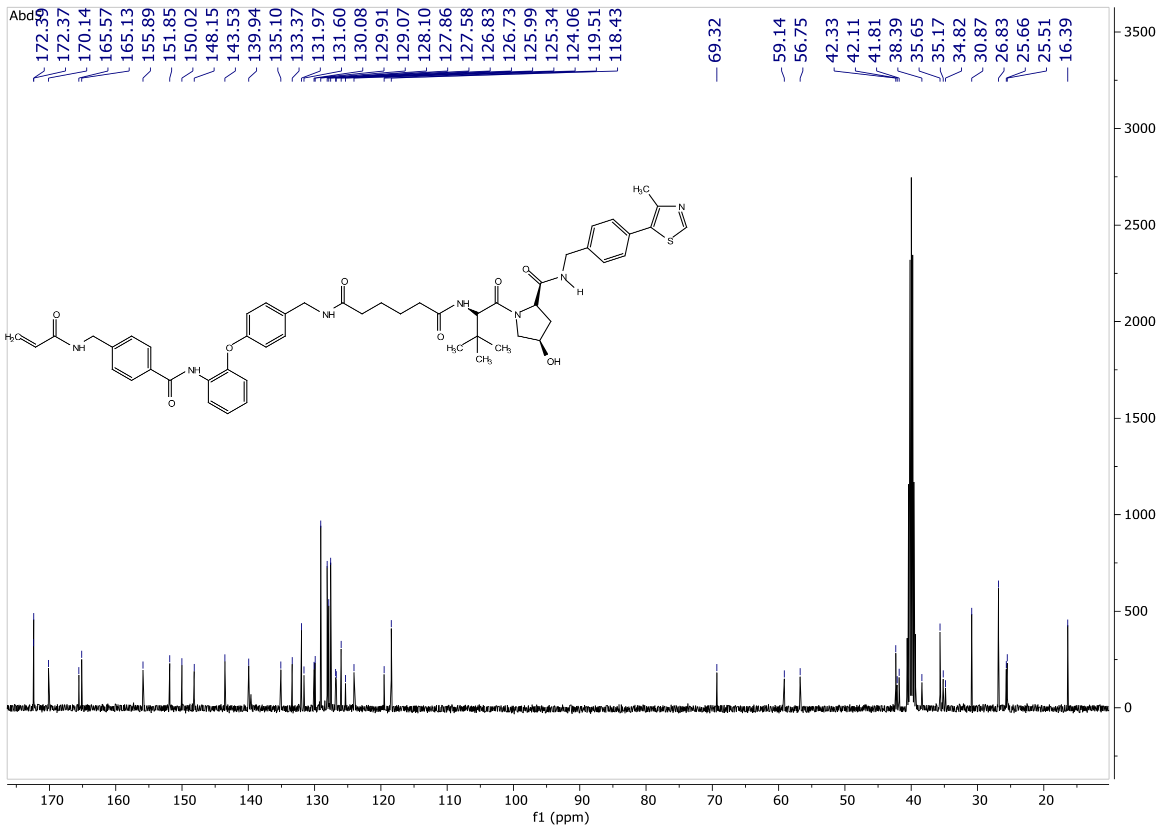
Abd5
172.39 172.37 170.14 165.57 165.13 155.89 151.85 150.02 148.15 143.53 139.94 135.10 133.37 131.97 131.60 130.08 129.91 129.07 128.10 127.86 127.58 126.83 126.73 125.99 125.34 124.06 119.51 118.43 69.32 59.14 56.75 42.33 42.11 41.81 38.39 35.65 35.17 34.82 30.87 26.83 25.66 25.51 16.39
f1 (ppm)

34g (Abd5)

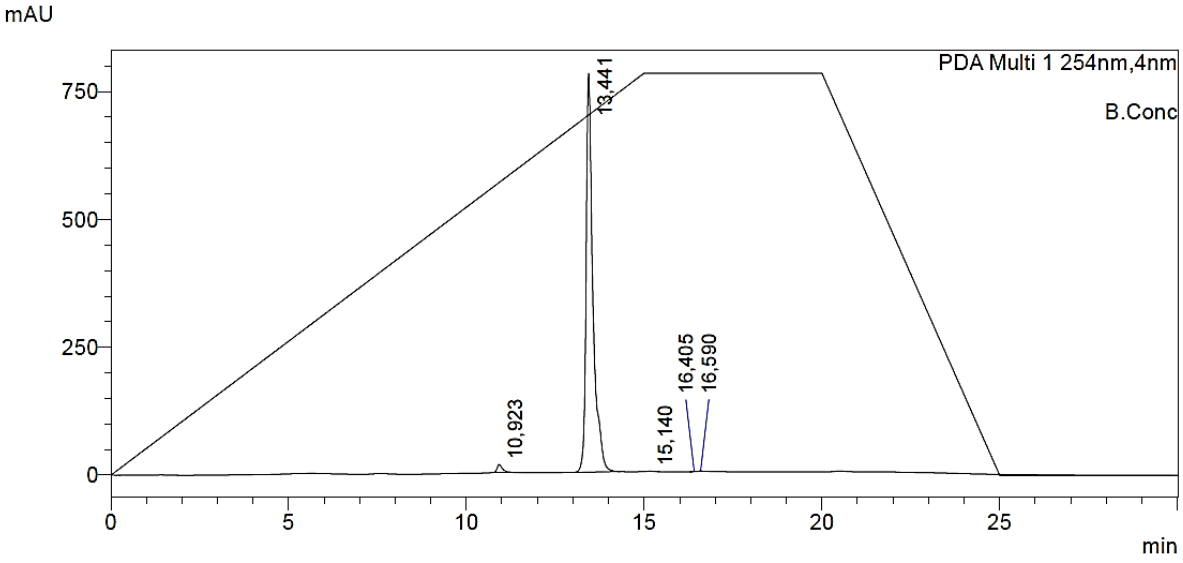

PDA Ch1 254nm

Peak#	Ret. Time	Area	Height	Area%
1	10,923	190582	16201	1,796
2	13,441	10371136	779829	97,757
3	15,140	20672	1872	0,195
4	16,405	11254	1289	0,106
5	16,590	15454	1648	0,146
Total		10609099	800839	100,000

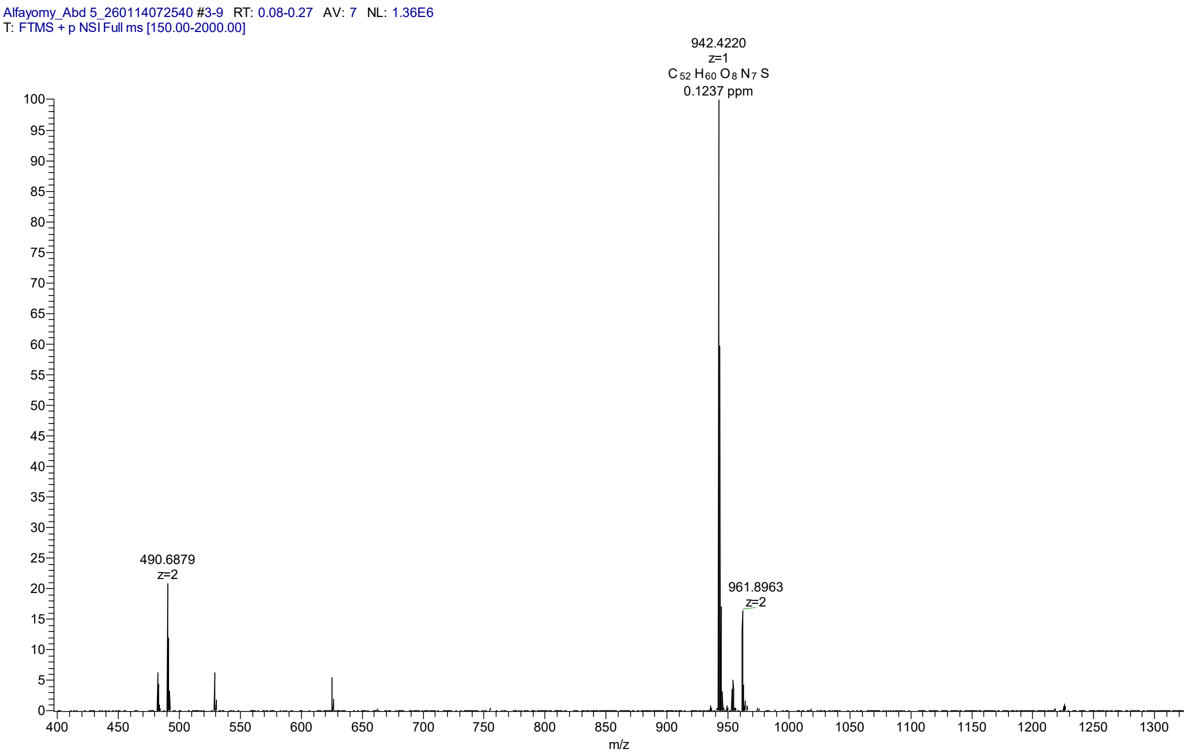

34h (Abd17)

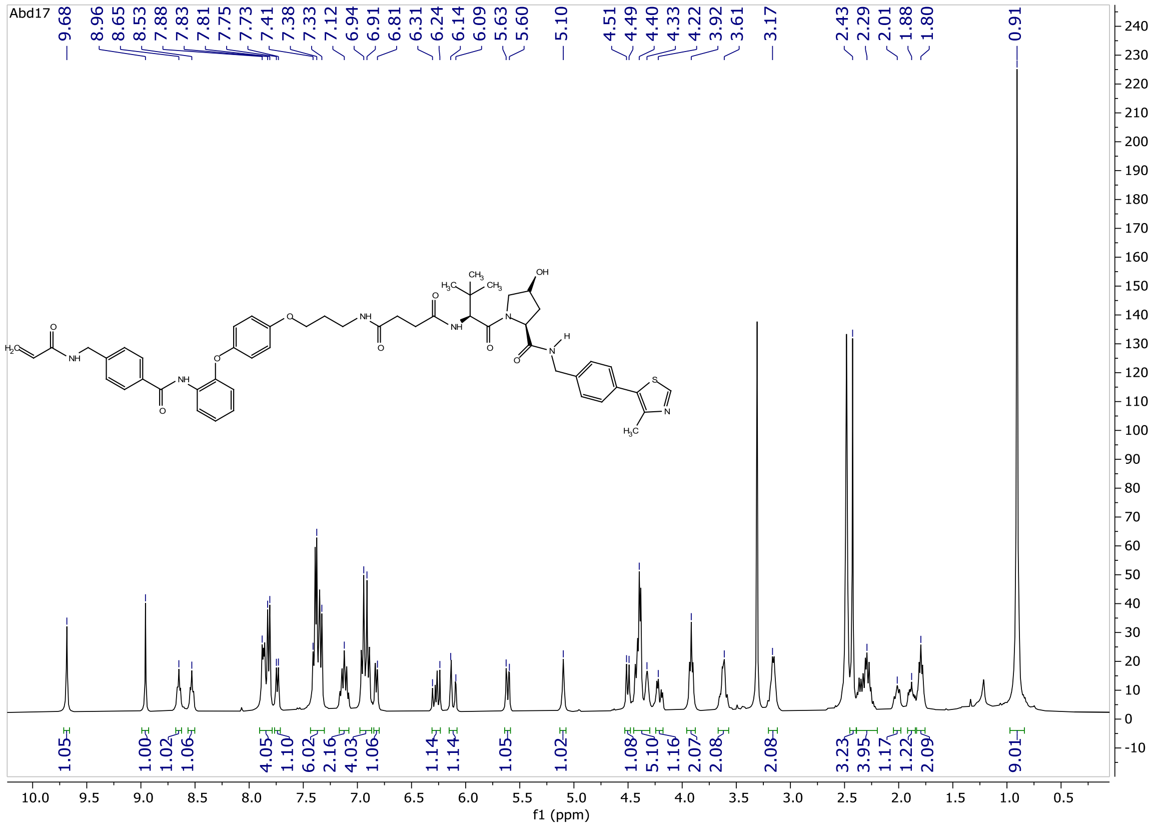

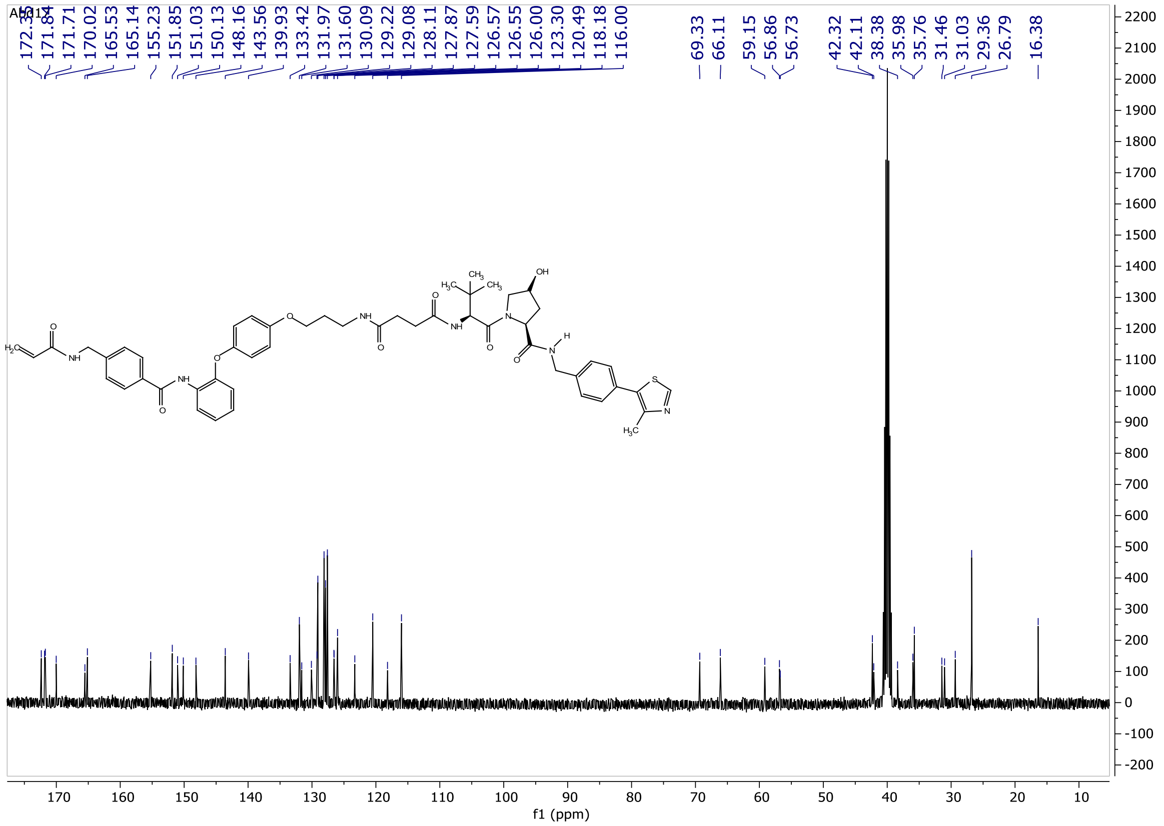

34h (Abd17)

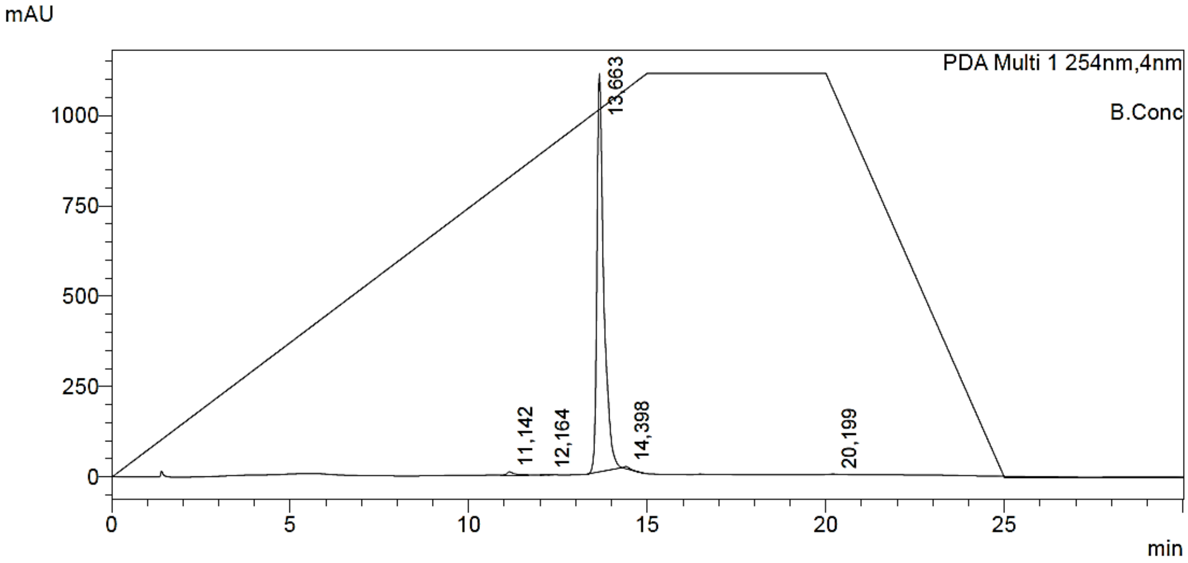

PDA Ch1 254nm

Peak#	Ret. Time	Area	Height	Area%
1	11,142	125501	8990	0,835
2	12,164	11230	1184	0,075
3	13,663	14811098	1102067	98,585
4	14,398	49736	5944	0,331
5	20,199	26142	1750	0,174
Total		15023707	1119935	100,000

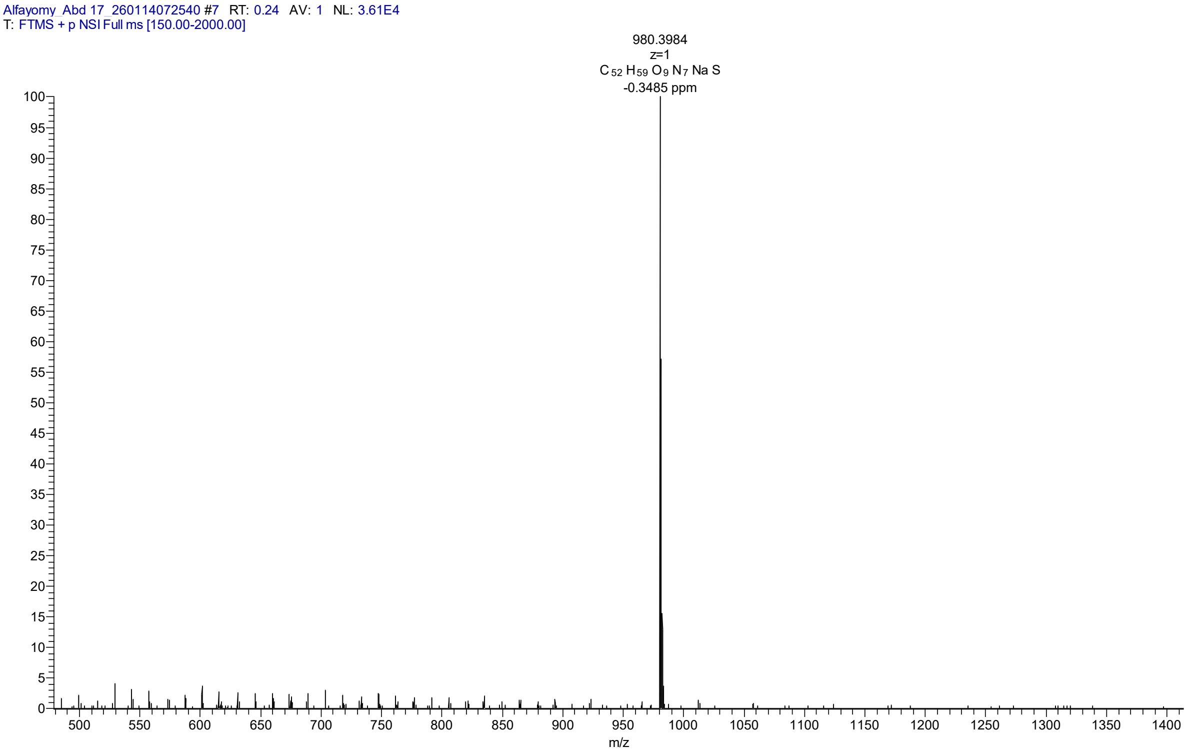

34i (Abd7)

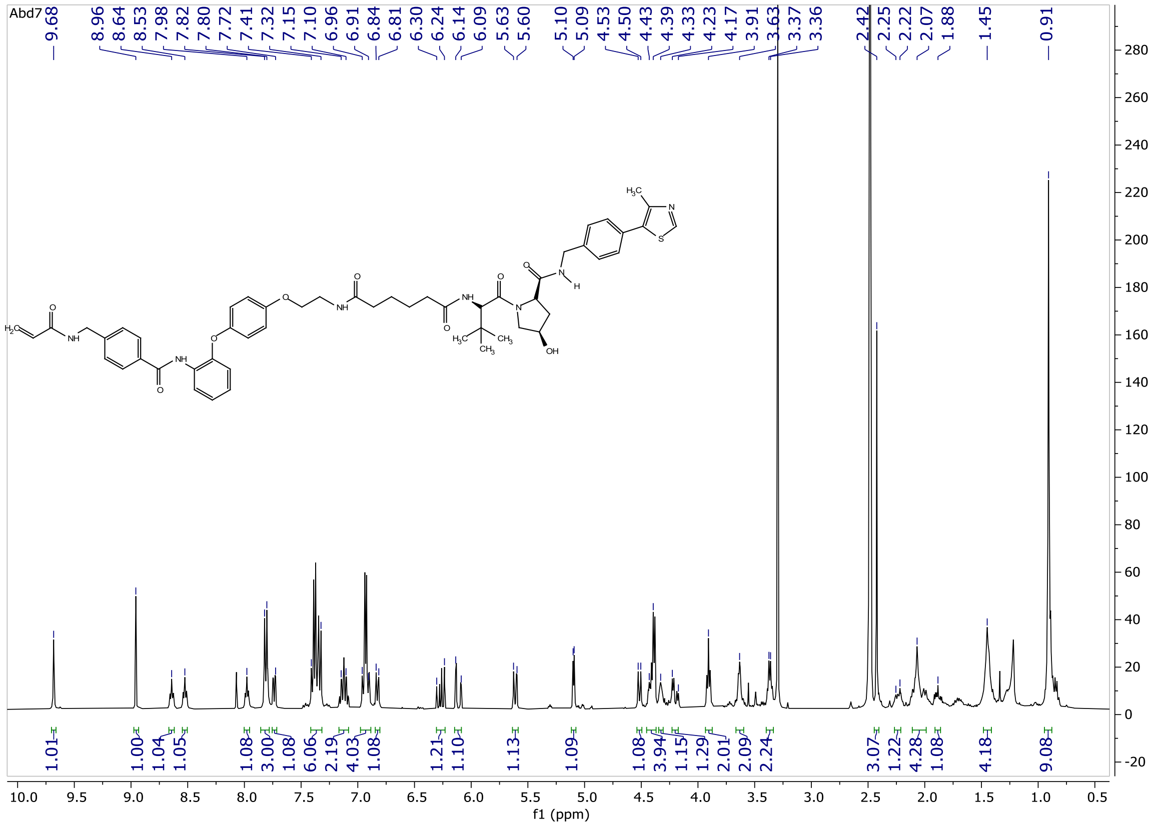

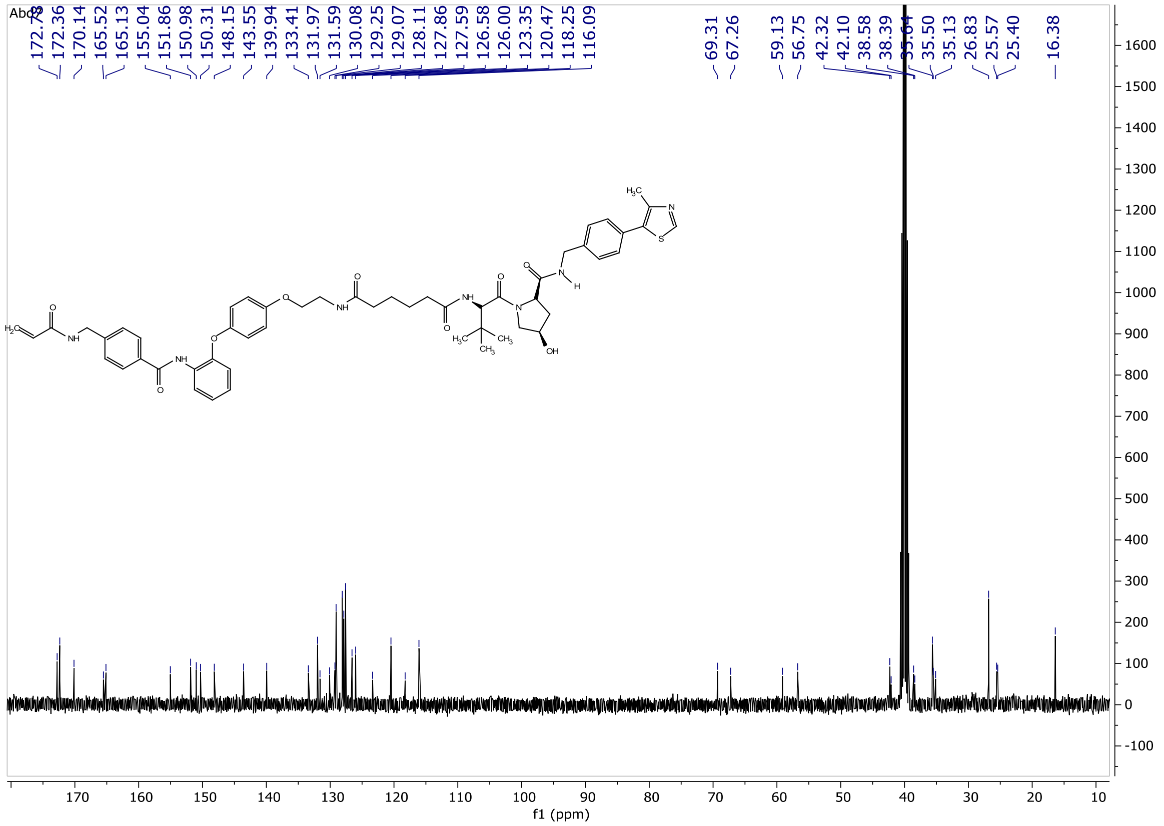

34i (Abd7)

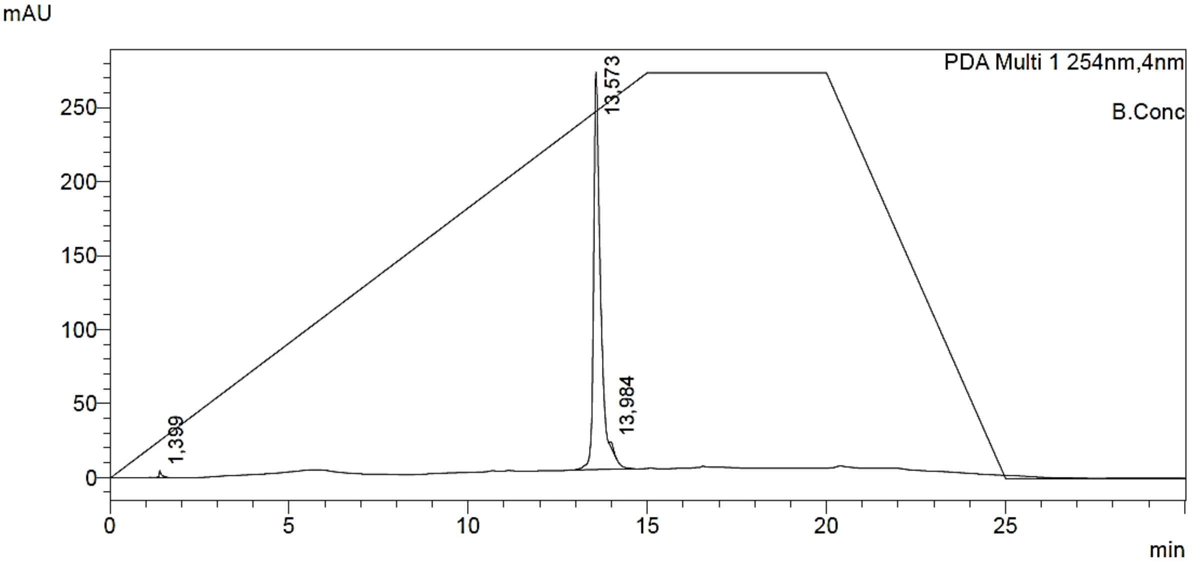

PDA Ch1 254nm

Peak#	Ret. Time	Area	Height	Area%
1	1,399	22778	4534	0,611
2	13,573	3685882	267964	98,834
3	13,984	20708	3417	0,555
Total		3729369	275916	100,000

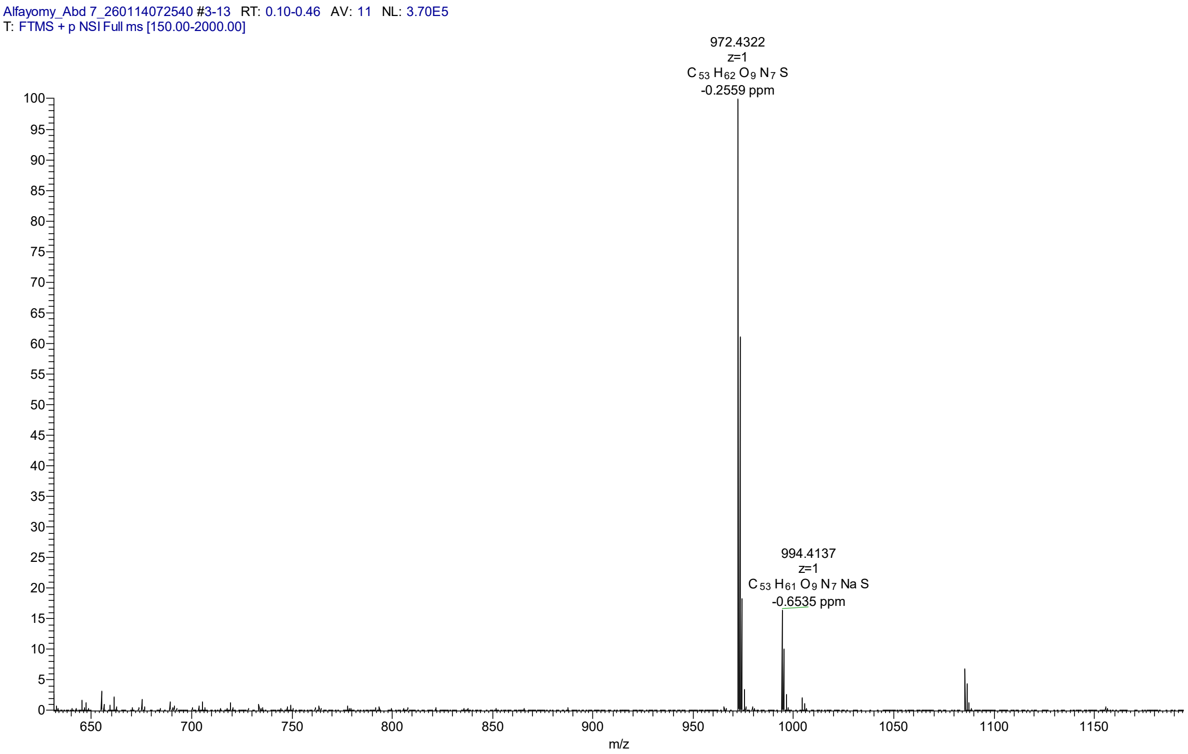

34j (Abd14)

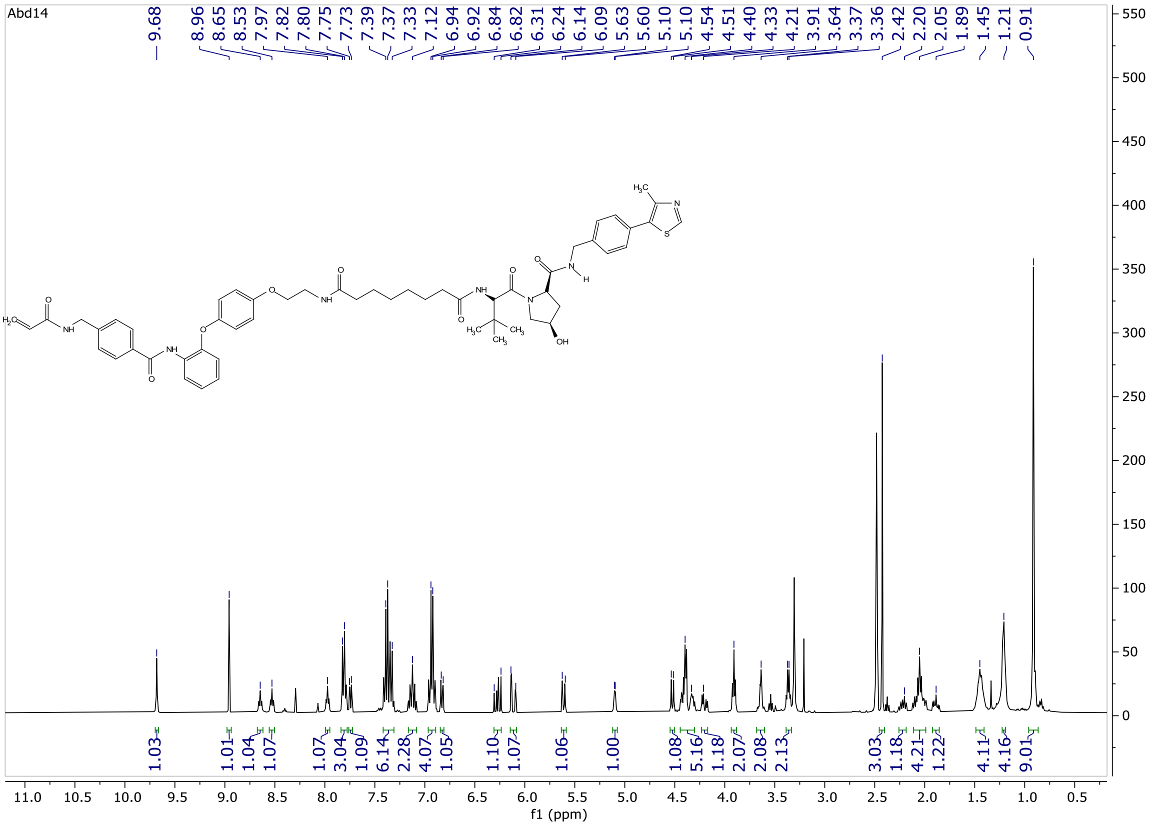
Abd14
9.68
8.96
8.65
8.53
7.97
7.82
7.80
7.75
7.73
7.39
7.37
7.33
7.12
6.94
6.92
6.84
6.82
6.31
6.24
6.14
6.09
5.63
5.60
5.10
5.10
4.54
4.51
4.40
4.33
4.21
3.91
3.64
3.37
3.36
2.42
2.20
2.05
1.89
1.45
1.21
0.91
1.03
1.01
1.04
1.07
1.07
3.04
1.09
6.14
2.28
4.07
1.05
1.10
1.07
1.06
1.00
1.08
5.16
1.18
2.07
2.08
2.13
3.03
1.18
4.21
1.22
4.11
4.16
9.01
f1 (ppm)

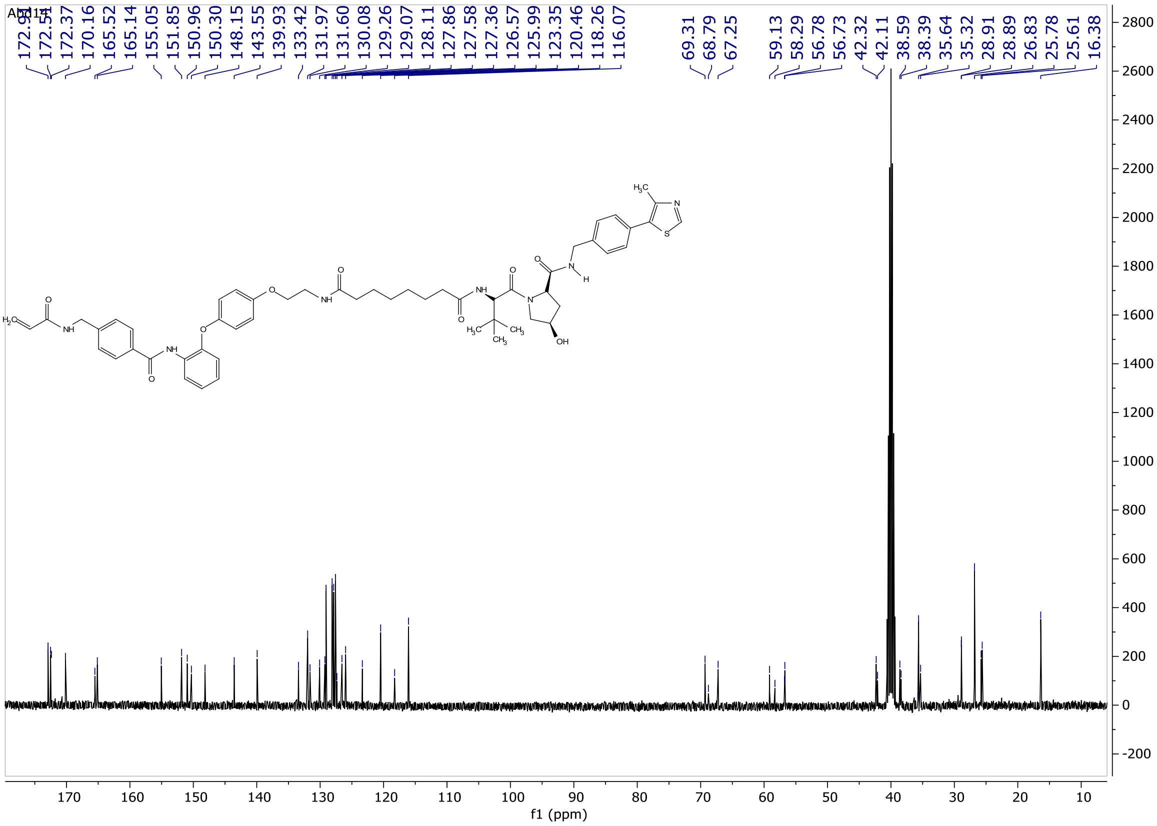
Abd14
172.37
170.16
165.52
165.14
155.05
151.85
150.96
150.30
148.15
143.55
139.93
133.42
131.97
131.60
130.08
129.26
129.07
128.11
127.86
127.58
127.36
126.57
125.99
123.35
120.46
118.26
116.07
69.31
68.79
67.25
59.13
58.29
56.78
56.73
42.32
42.11
38.59
38.39
35.64
35.32
28.91
28.89
26.83
25.78
25.61
16.38
f1 (ppm)

34j (Abd14)

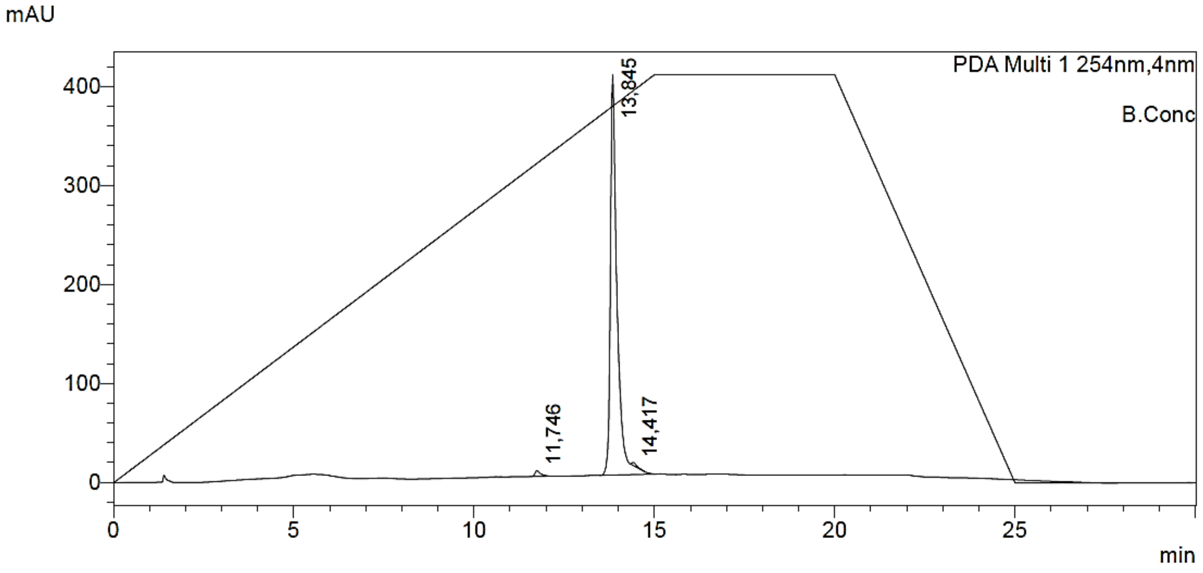

PDA Ch1 254nm

Peak#	Ret. Time	Area	Height	Area%
1	11,746	67130	5870	1,268
2	13,845	5198837	404386	98,235
3	14,417	26301	3184	0,497
Total		5292268	413440	100,000

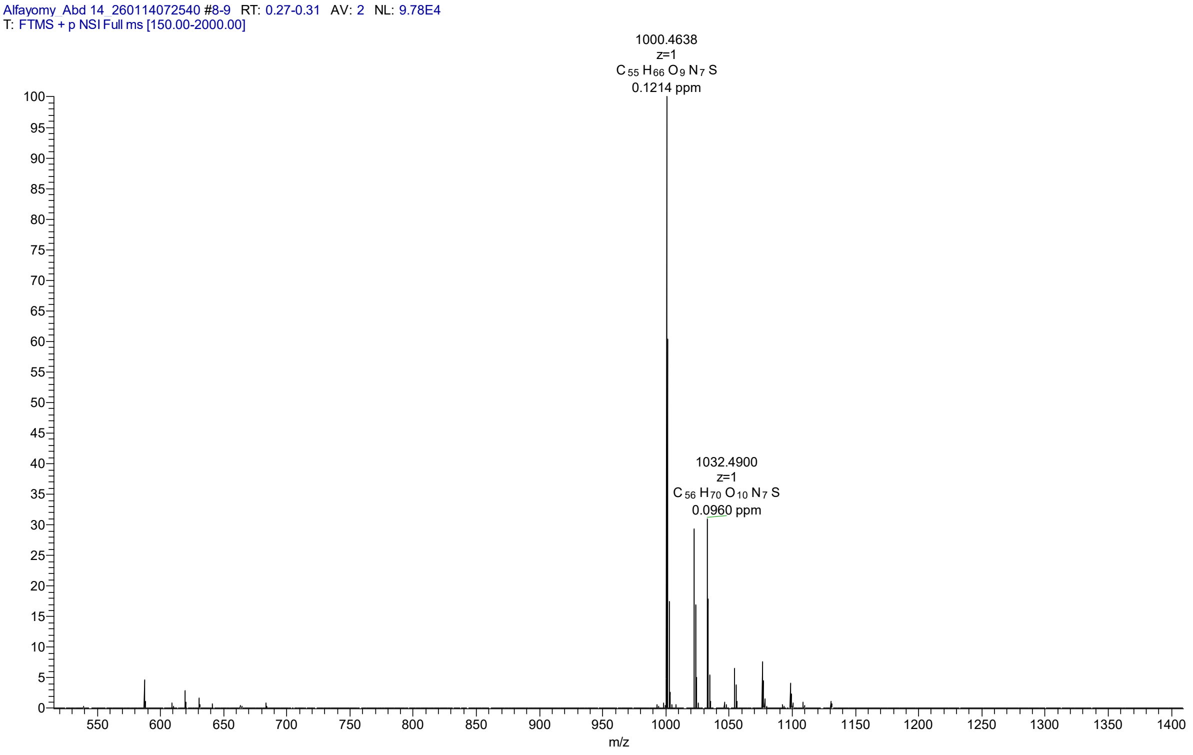

34k (Abd43)

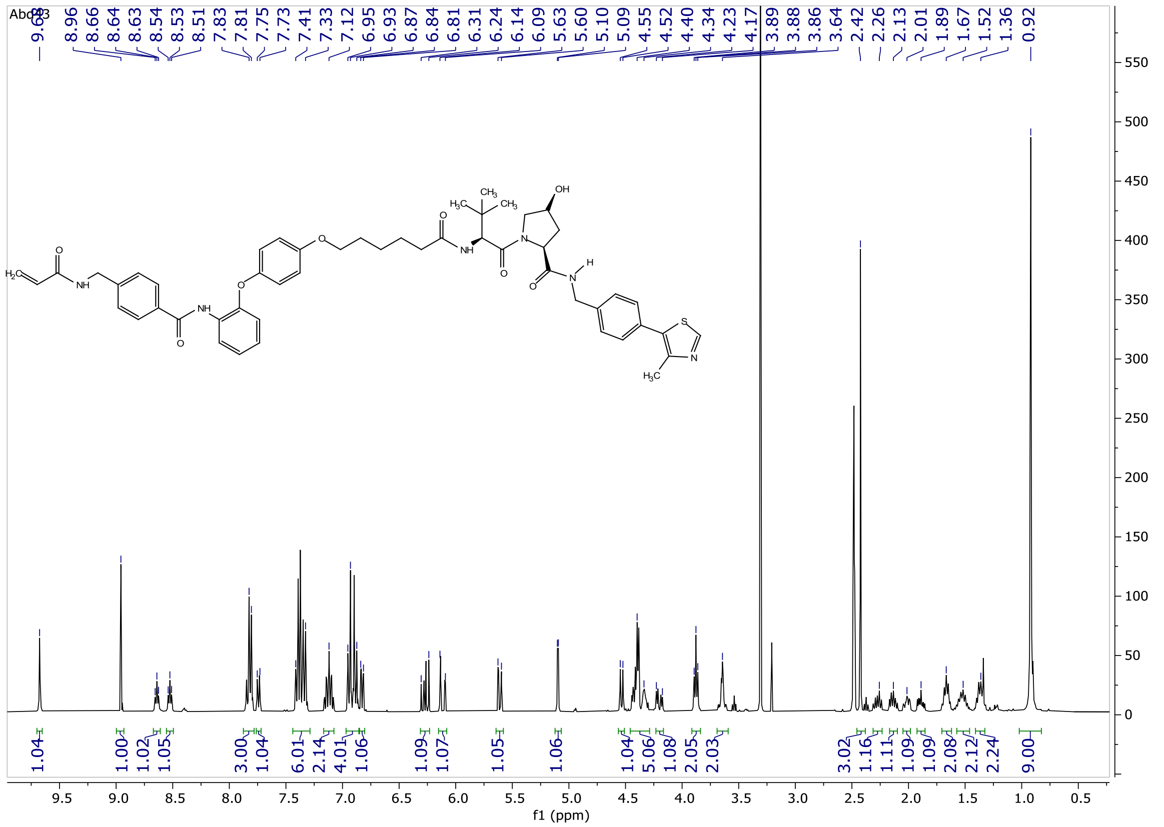

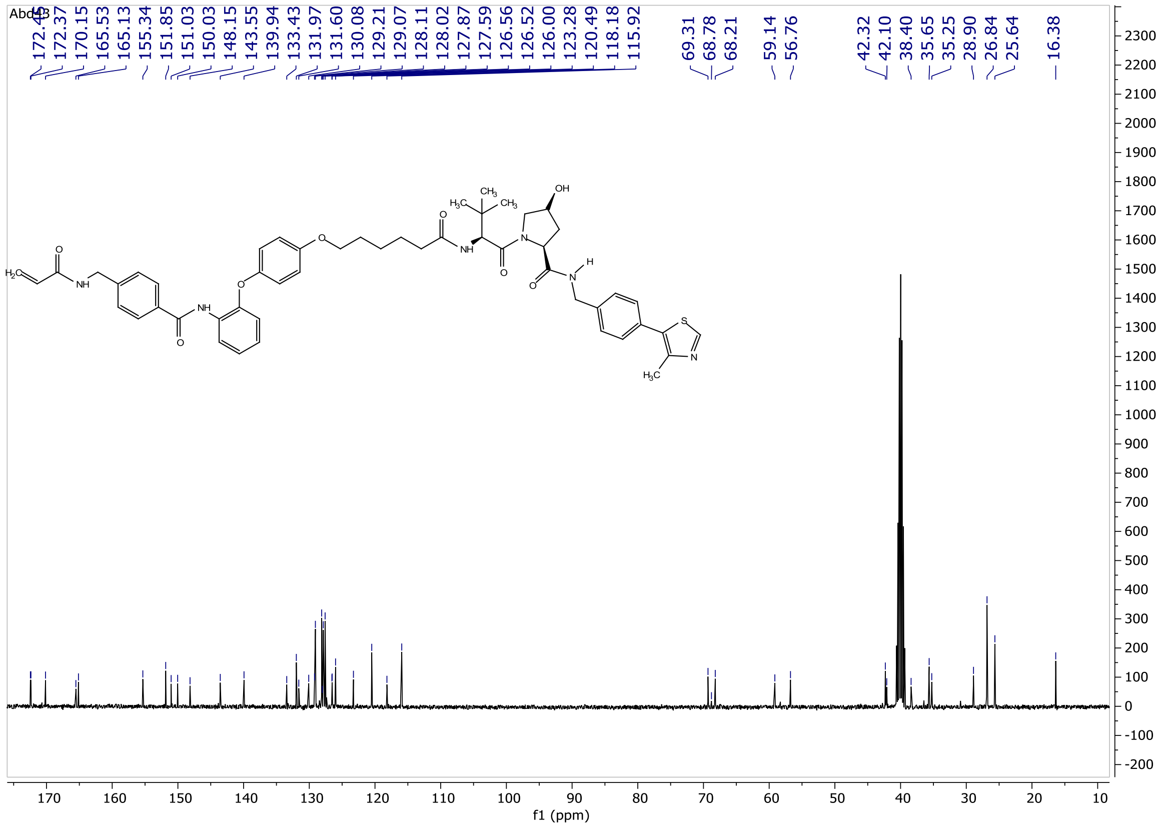

34k (Abd43)

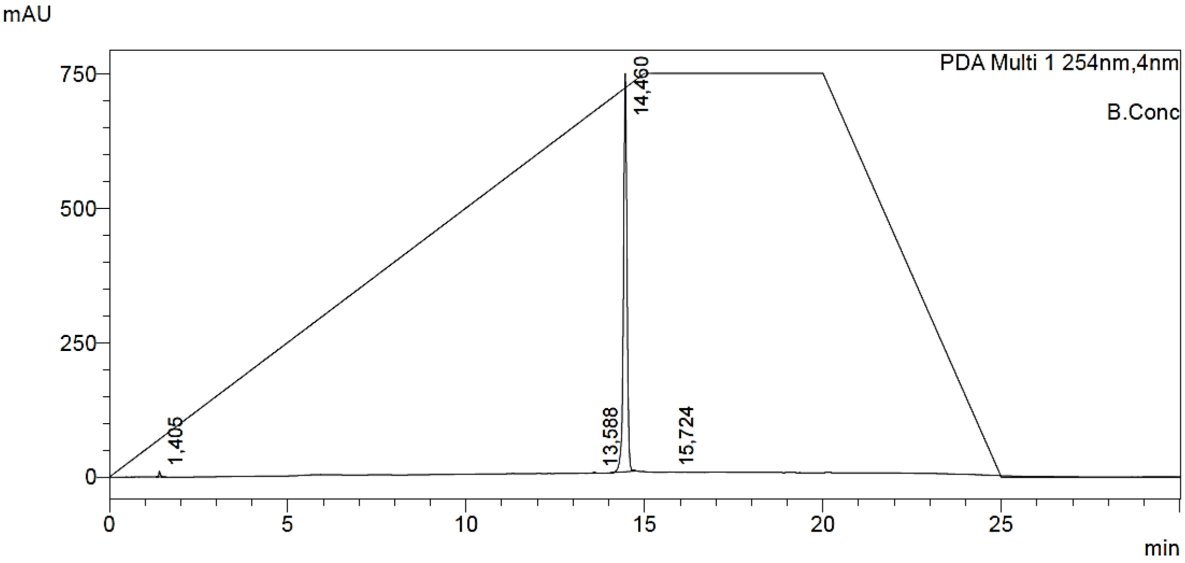

PDA Ch1 254nm

Peak#	Ret. Time	Area	Height	Area%
1	1,405	34292	10540	0,665
2	13,588	8779	1360	0,170
3	14,460	5107015	741613	99,002
4	15,724	8419	899	0,163
Total		5158505	754412	100,000

Alfayomy_Abd 43_260114094707 #23-27 RT: 0.82-0.97 AV: 5 NL: 1.06E5
T: FTMS + p NSI Full ms [150.00-2000.00]

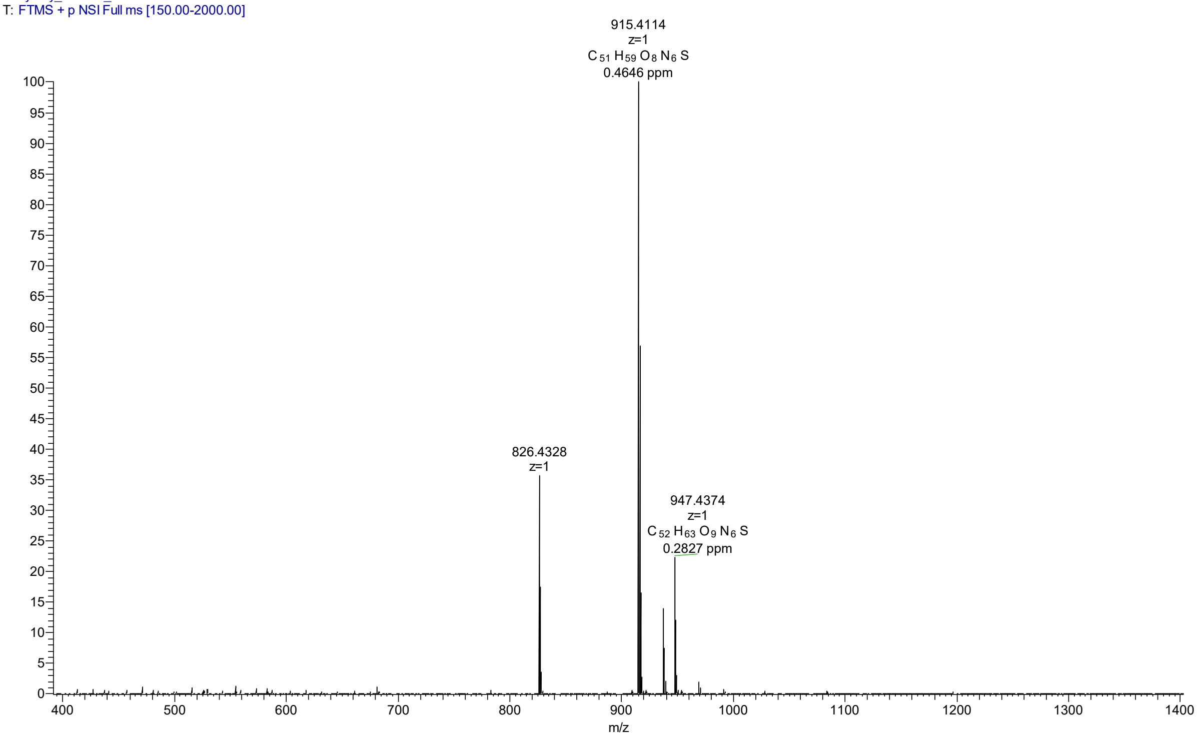

34l (Abd13)

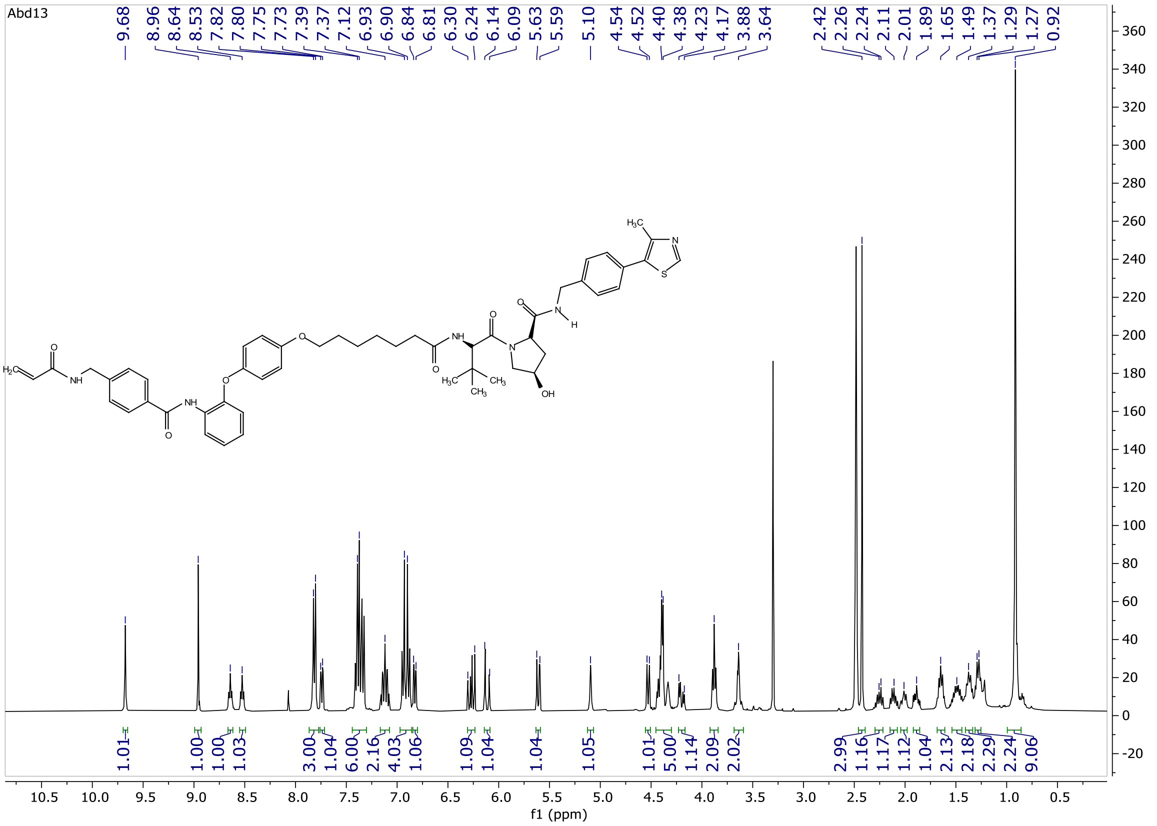
Abd13
9.68 8.96 8.64 8.53 7.82 7.80 7.75 7.73 7.39 7.37 7.12 6.93 6.90 6.84 6.81 6.30 6.24 6.14 6.09 5.63 5.59 5.10 4.54 4.52 4.40 4.38 4.23 4.17 3.88 3.64 2.42 2.26 2.24 2.11 2.01 1.89 1.65 1.49 1.37 1.29 1.27 0.92
1.01 1.00 1.00 1.03 3.00 1.04 6.00 2.16 4.03 1.06 1.09 1.04 1.04 1.05 1.01 5.00 1.14 2.09 2.02 2.99 1.16 1.17 1.12 1.04 2.13 2.18 2.29 2.24 9.06
f1 (ppm)

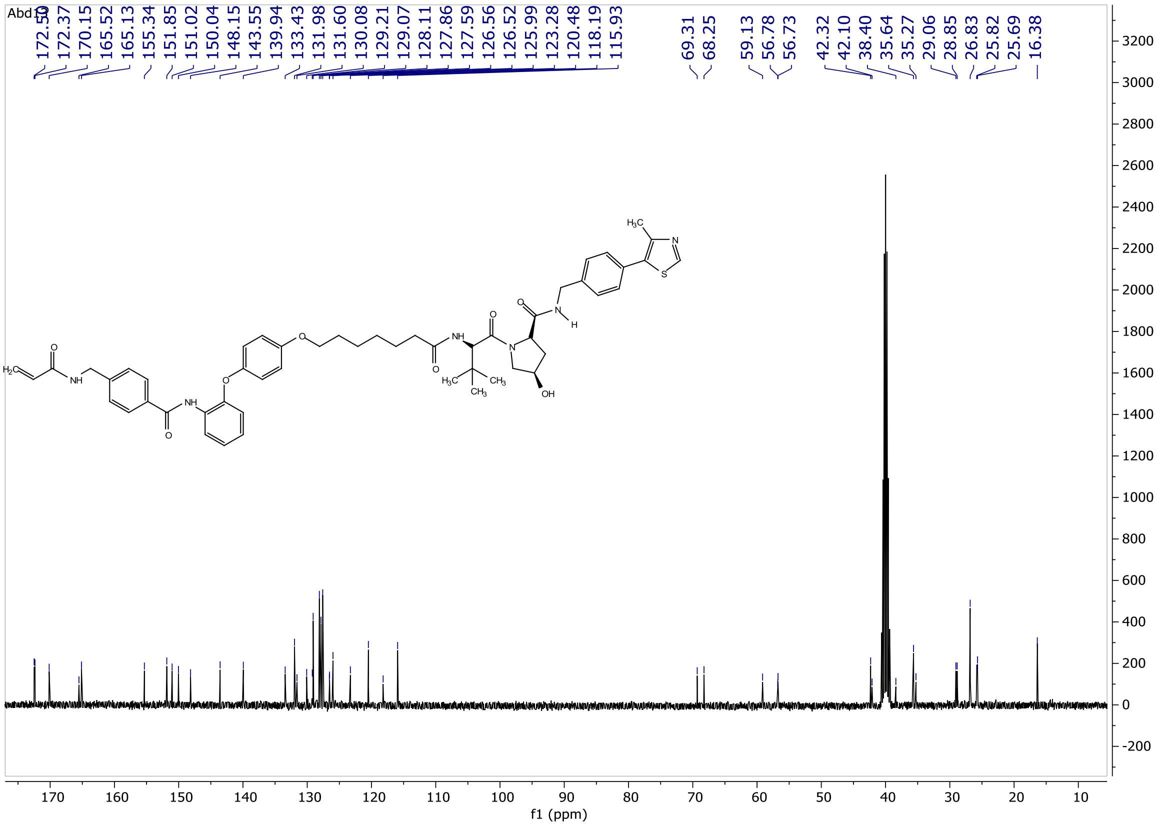
Abd13
172.50 172.37 170.15 165.52 165.13 155.34 151.85 151.02 150.04 148.15 143.55 139.94 133.43 131.98 131.60 130.08 129.21 129.07 128.11 127.86 127.59 126.56 126.52 125.99 123.28 120.48 118.19 115.93 69.31 68.25 59.13 56.78 56.73 42.32 42.10 38.40 35.64 35.27 29.06 28.85 26.83 25.82 25.69 16.38
f1 (ppm)

34l (Abd13)

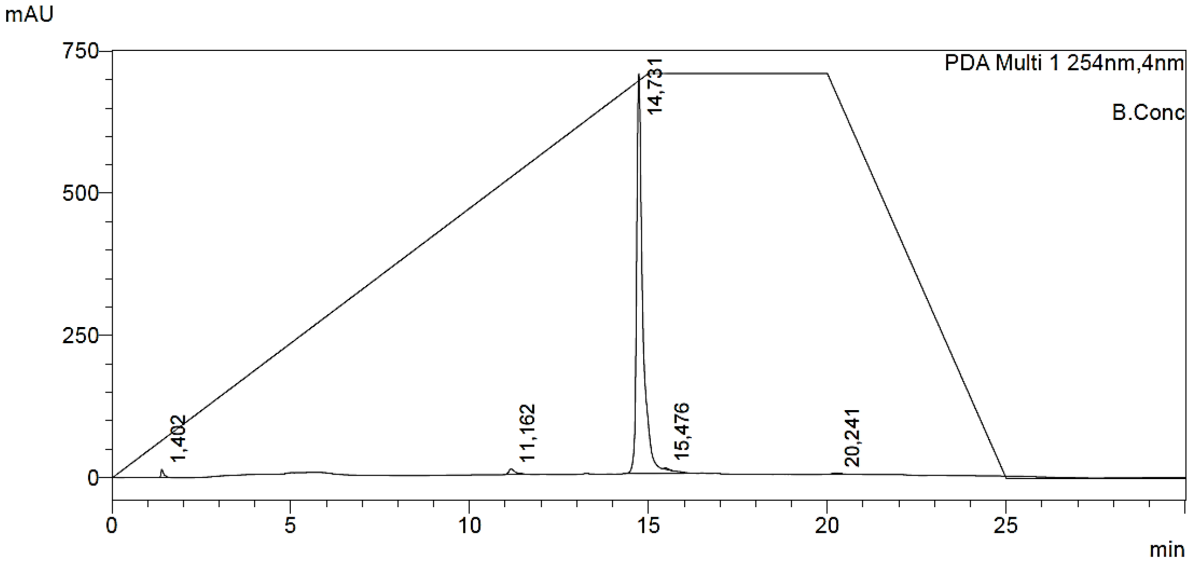

PDA Ch1 254nm

Peak#	Ret. Time	Area	Height	Area%
1	1,402	67717	13507	0,767
2	11,162	113177	9296	1,283
3	14,731	8587840	703602	97,328
4	15,476	22266	2559	0,252
5	20,241	32599	2188	0,369
Total		8823598	731153	100,000

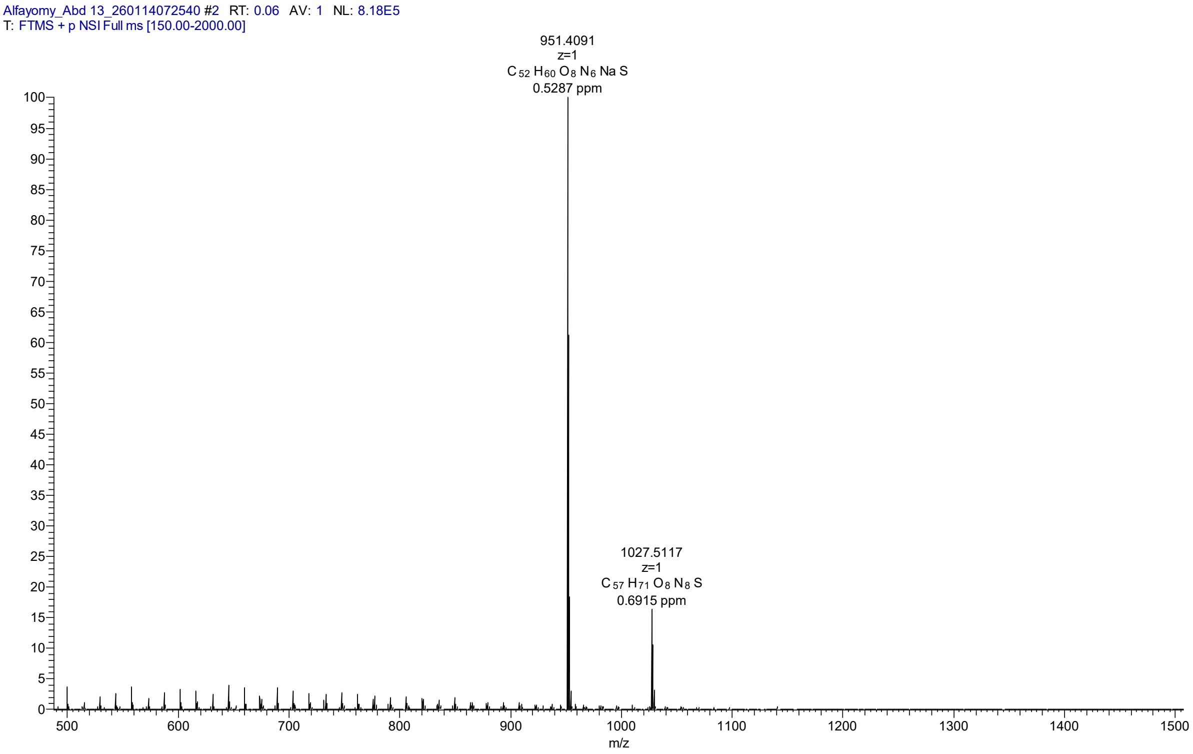

34m (Abd23)

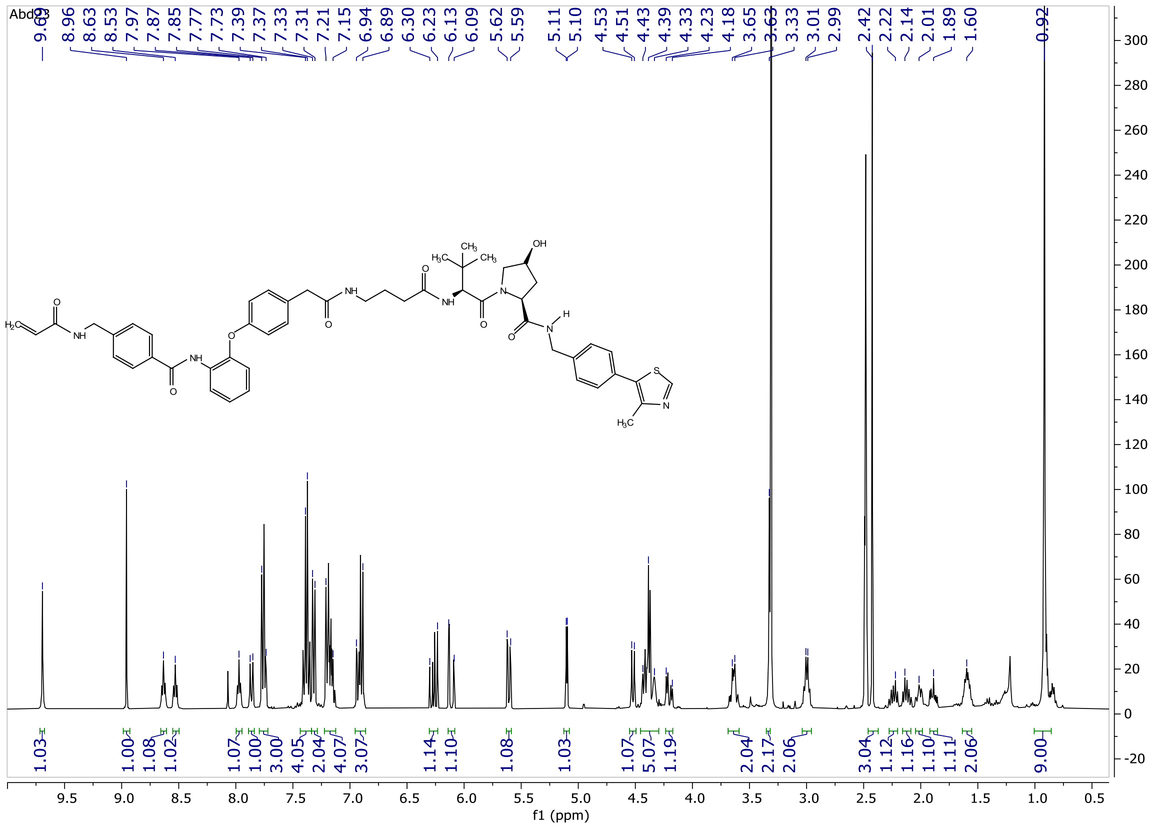

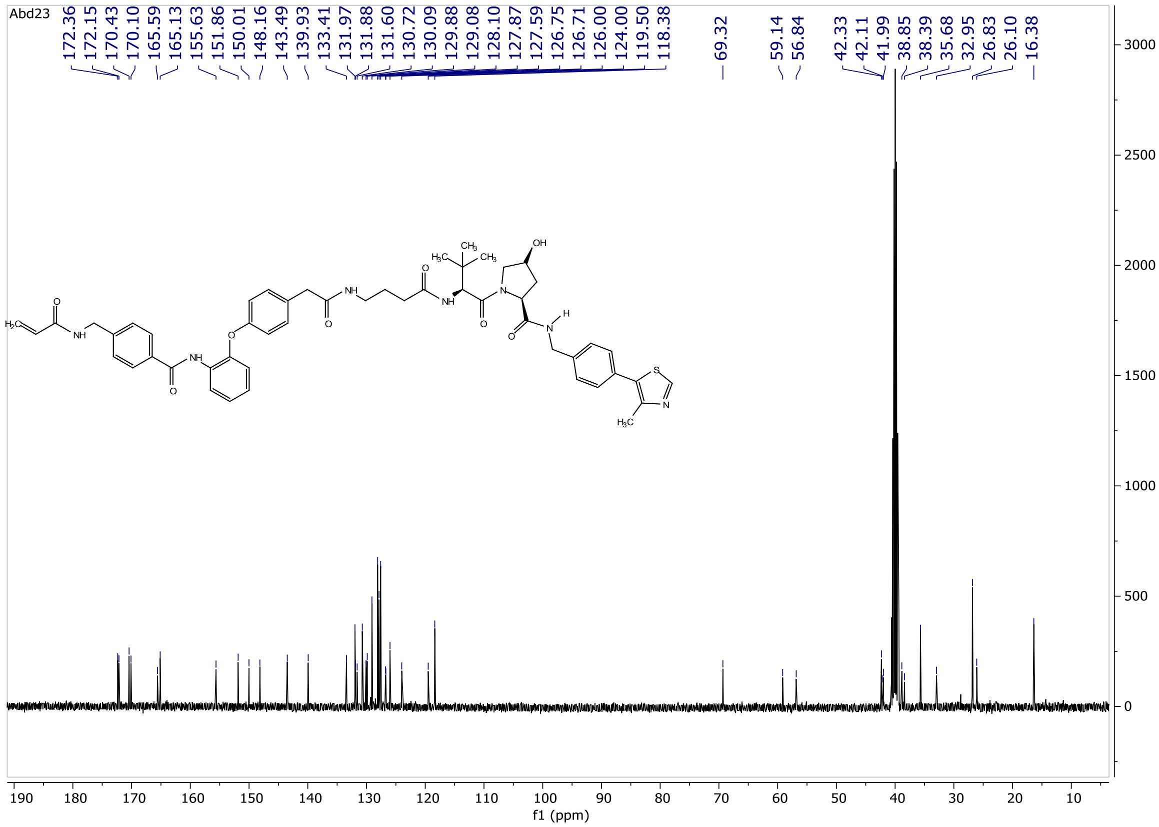

34m (Abd23)

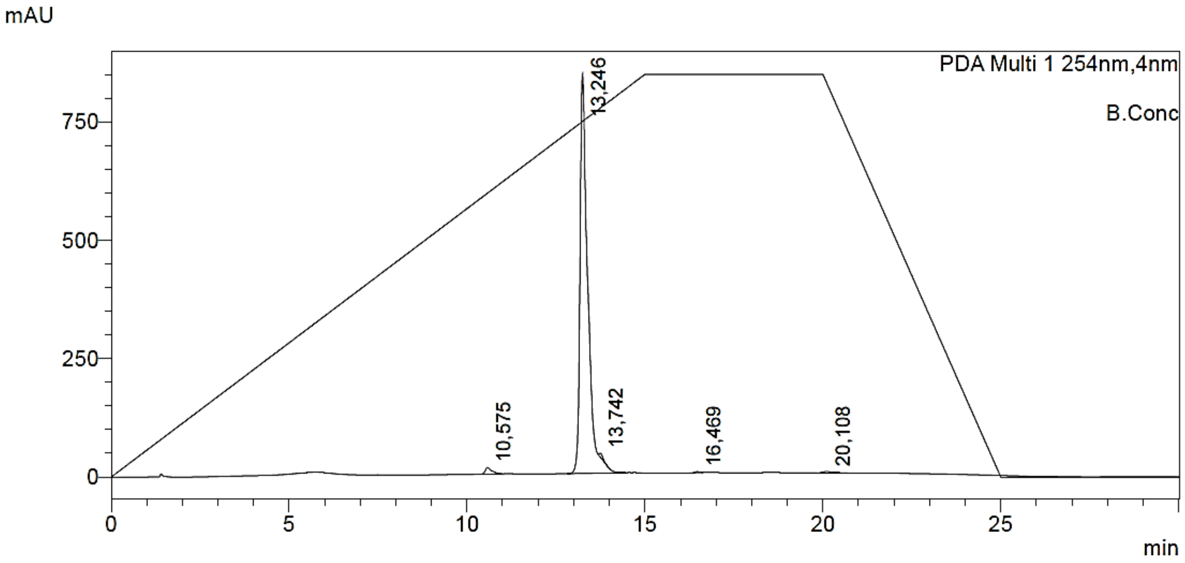

PDA Ch1 254nm

Peak#	Ret. Time	Area	Height	Area%
1	10,575	175742	13842	1,352
2	13,246	12714911	842613	97,785
3	13,742	49890	7360	0,384
4	16,469	14210	1959	0,109
5	20,108	48225	2634	0,371
Total		13002978	868408	100,000

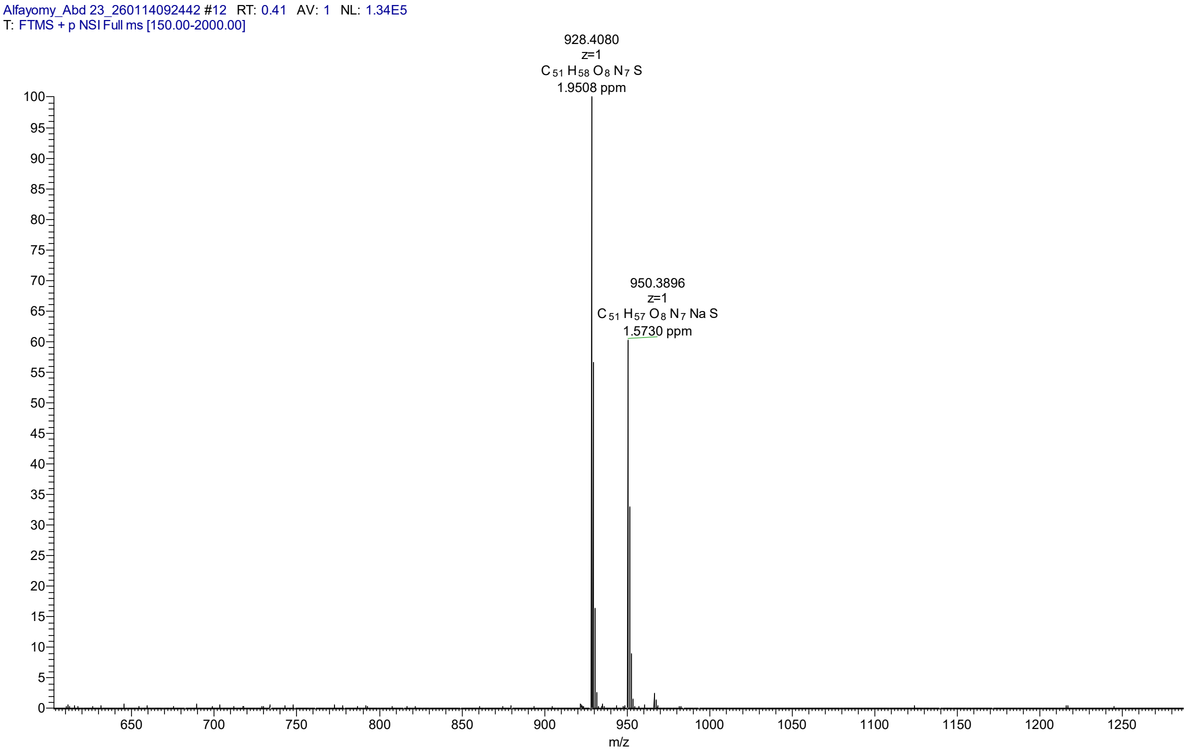

34n (Abd24)

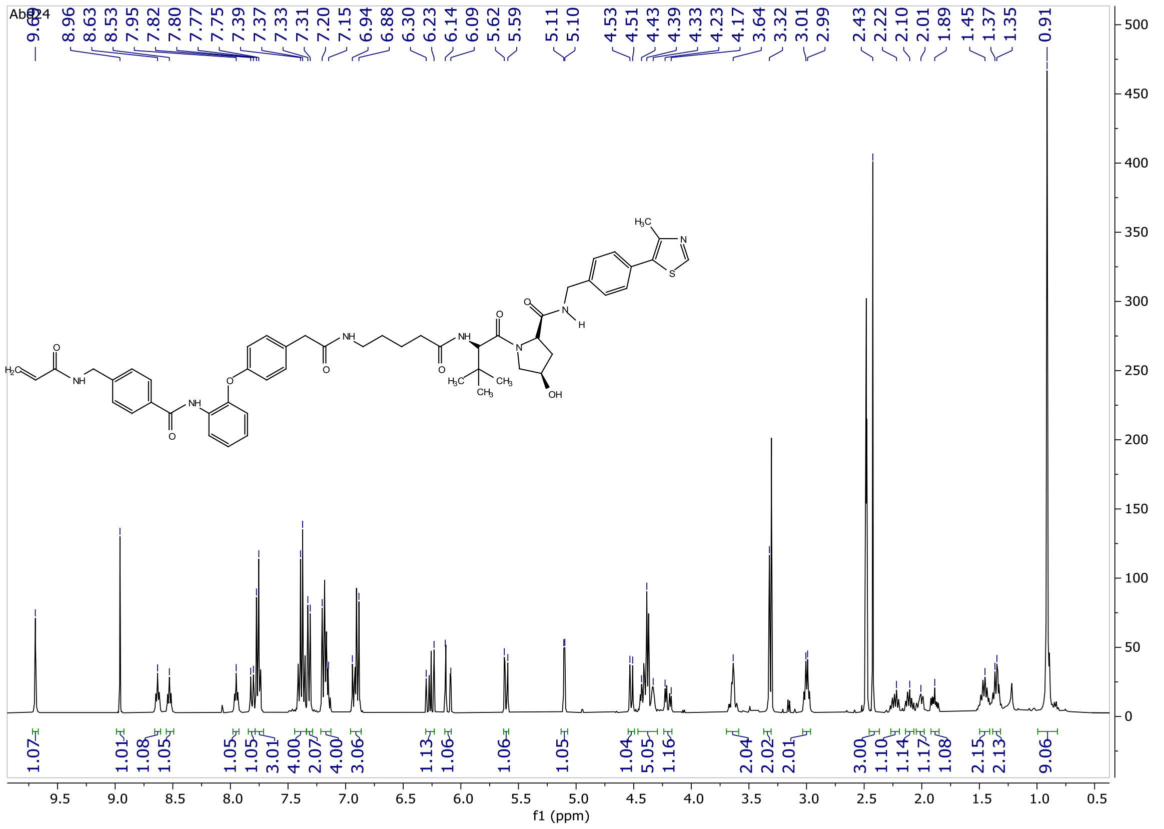

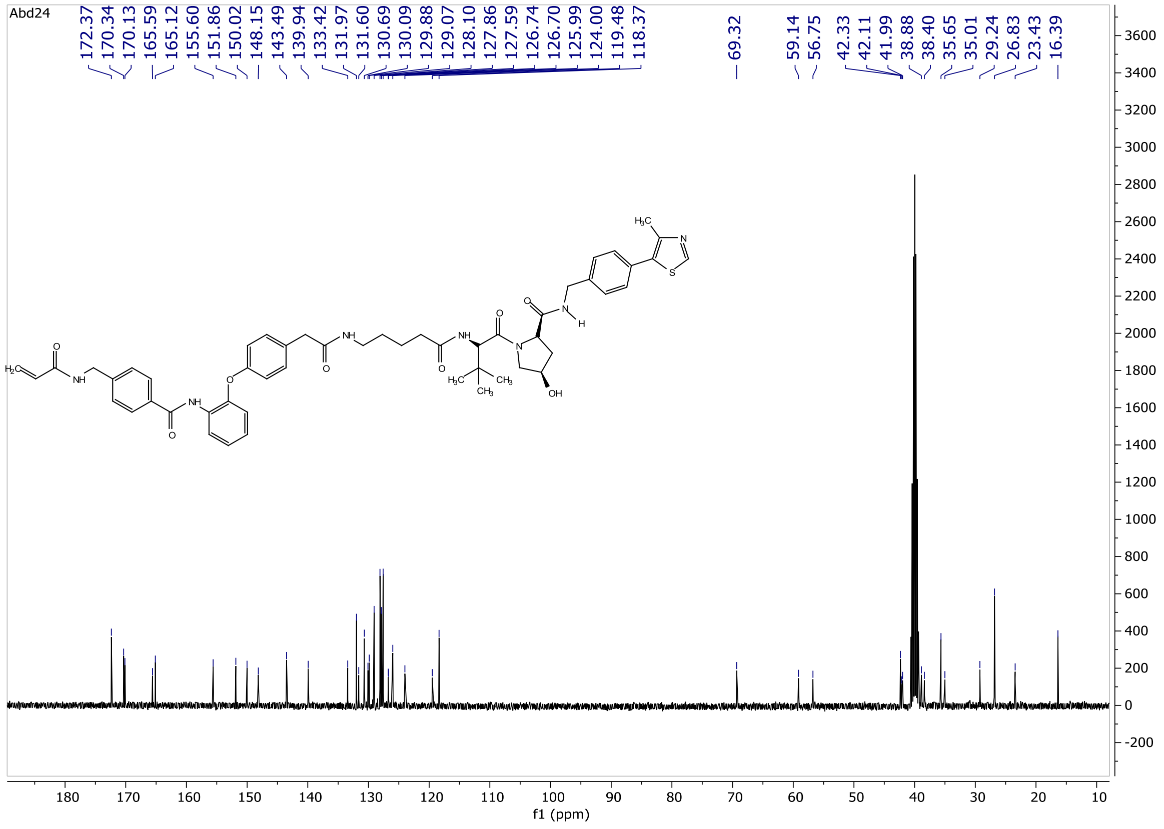

34n (Abd24)

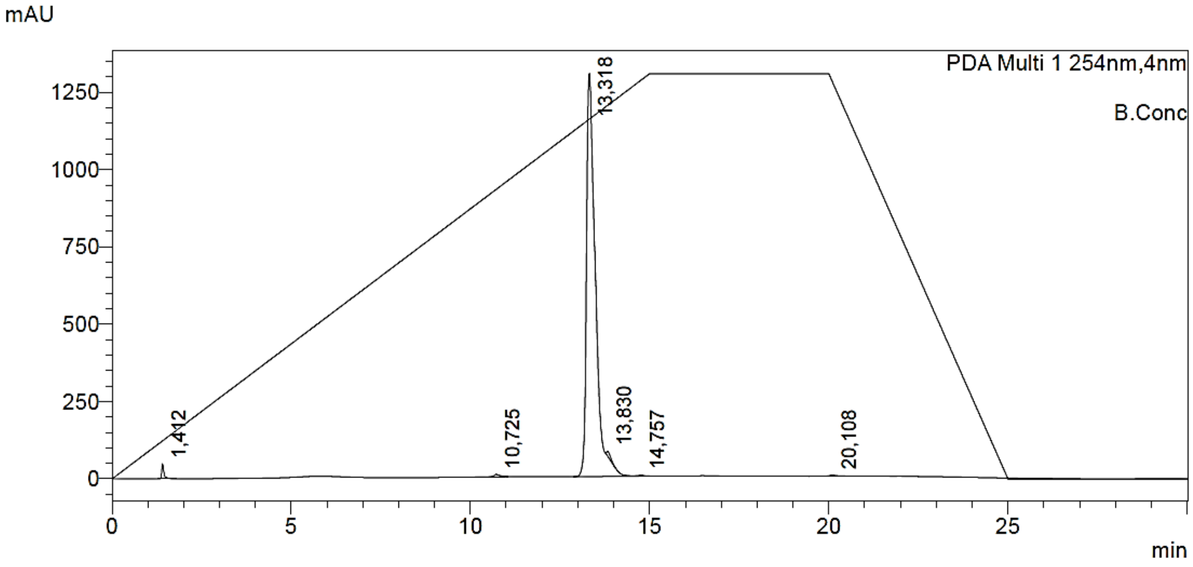

PDA Ch1 254nm

Peak#	Ret. Time	Area	Height	Area%
1	1,412	207679	46728	0,901
2	10,725	90880	7843	0,394
3	13,318	22563960	1302677	97,872
4	13,830	110588	14968	0,480
5	14,757	27590	2538	0,120
6	20,108	53915	2988	0,234
Total		23054612	1377743	100,000

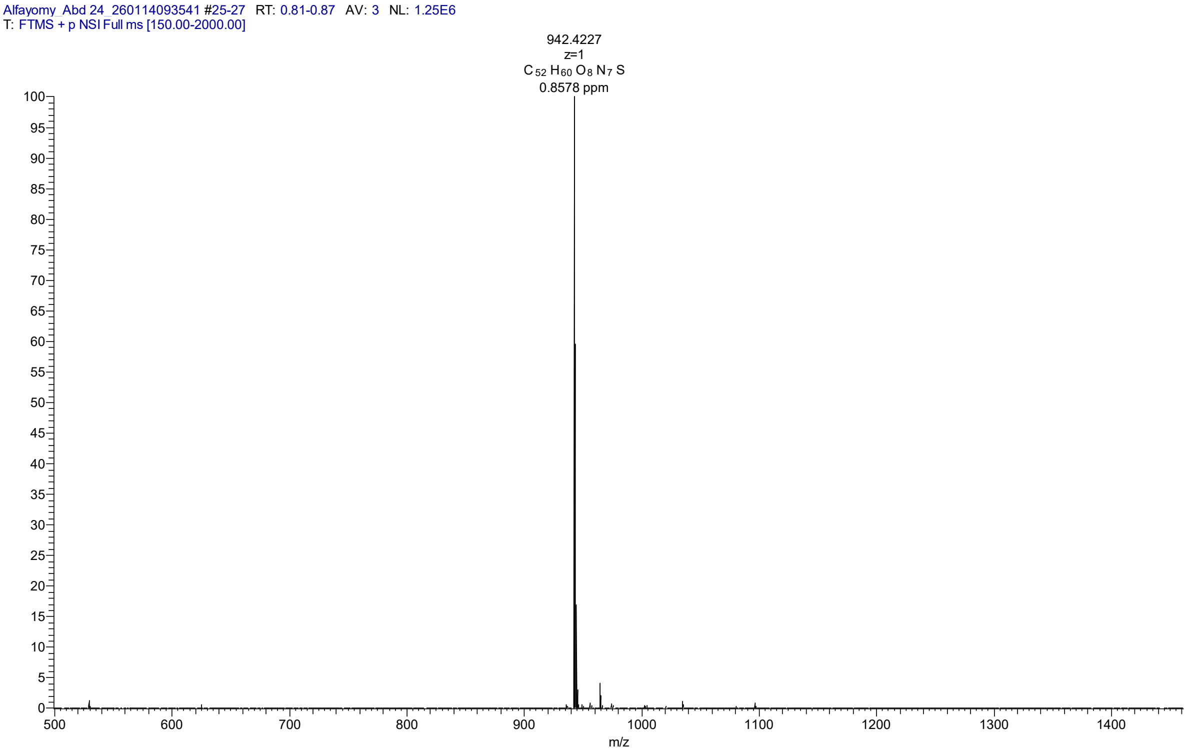

Figure S9. HPLC chromatograms, microsomal stability testing

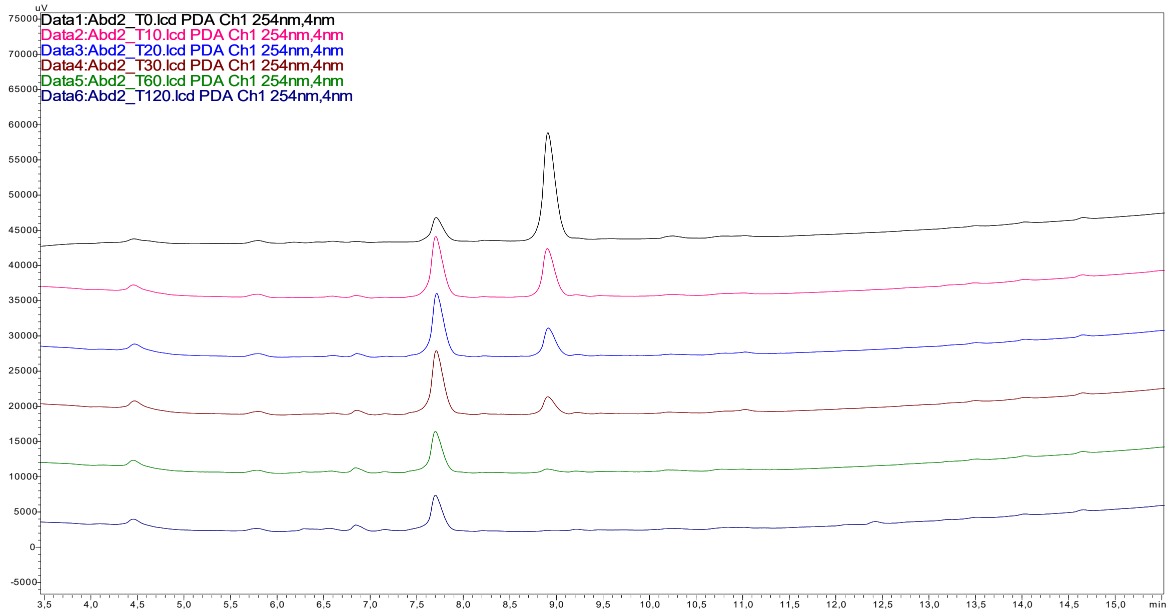

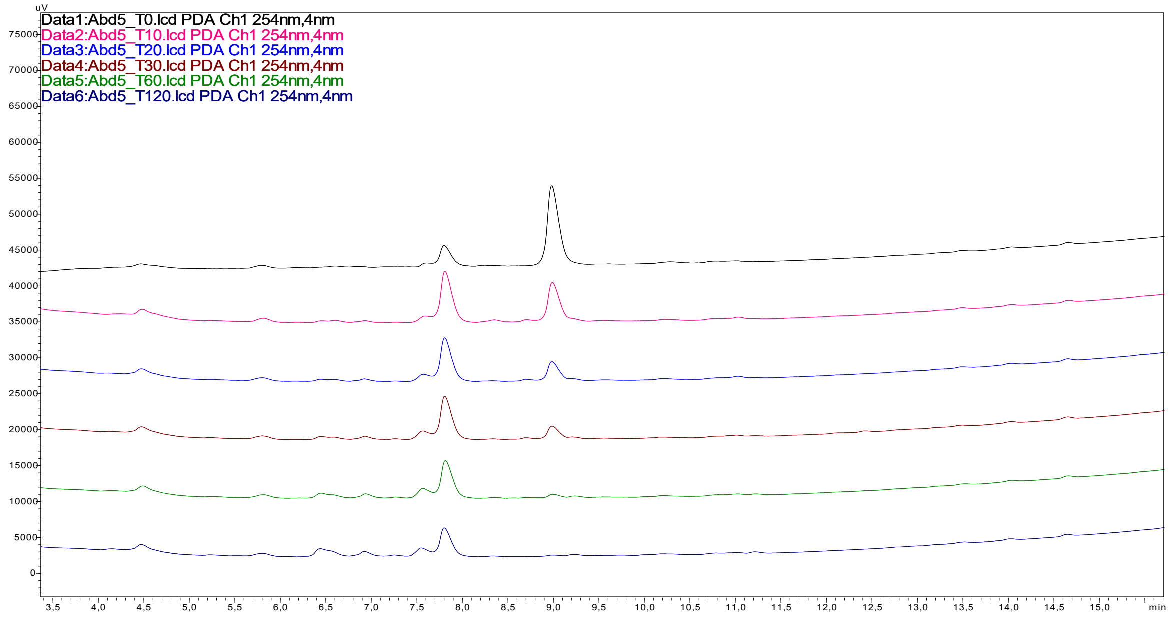

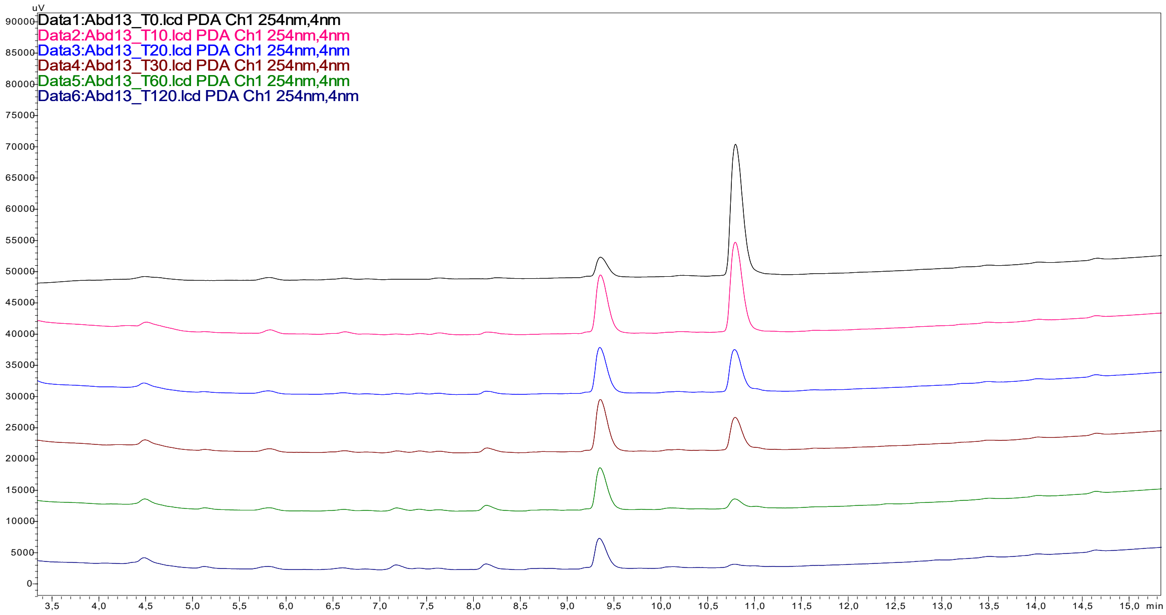
Data1:Abd13_T0.lcd PDA Ch1 254nm,4nm
Data2:Abd13_T10.lcd PDA Ch1 254nm,4nm
Data3:Abd13_T20.lcd PDA Ch1 254nm,4nm
Data4:Abd13_T30.lcd PDA Ch1 254nm,4nm
Data5:Abd13_T60.lcd PDA Ch1 254nm,4nm
Data6:Abd13_T120.lcd PDA Ch1 254nm,4nm
uV
min

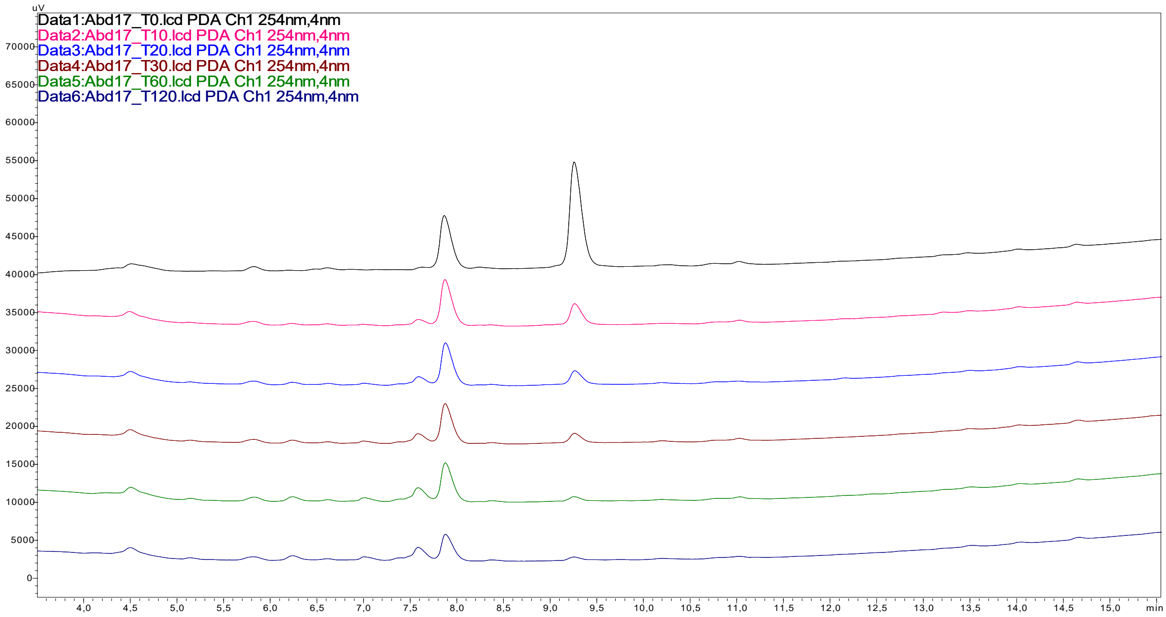
Data1:Abd17_T0.lcd PDA Ch1 254nm,4nm
Data2:Abd17_T10.lcd PDA Ch1 254nm,4nm
Data3:Abd17_T20.lcd PDA Ch1 254nm,4nm
Data4:Abd17_T30.lcd PDA Ch1 254nm,4nm
Data5:Abd17_T60.lcd PDA Ch1 254nm,4nm
Data6:Abd17_T120.lcd PDA Ch1 254nm,4nm
uV
min

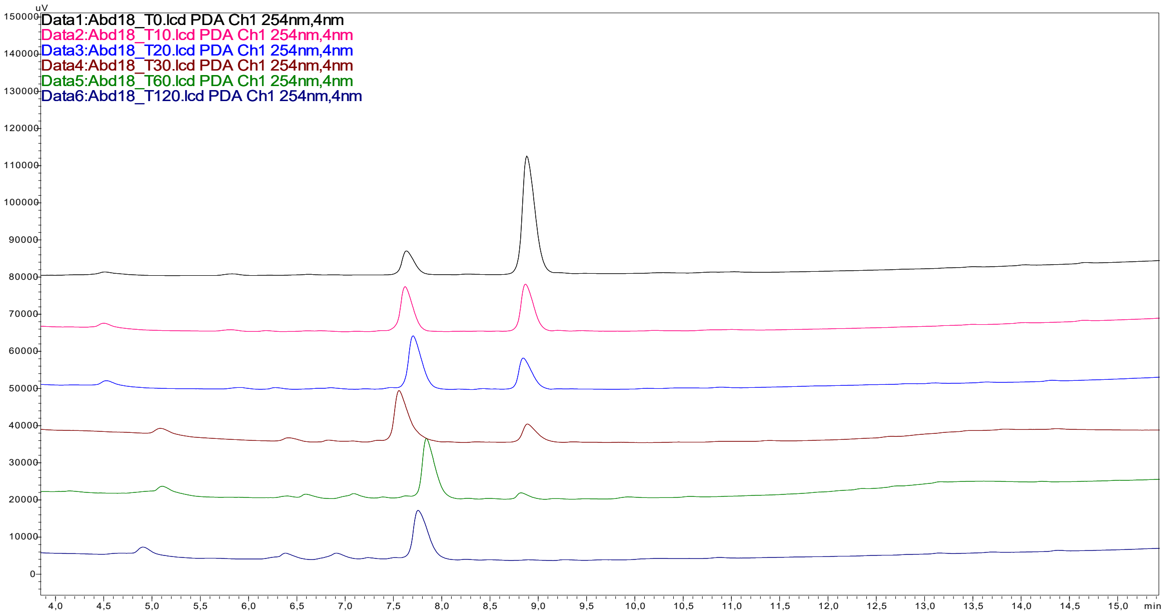
uV
Data1:Abd18_T0.lcd PDA Ch1 254nm,4nm
Data2:Abd18_T10.lcd PDA Ch1 254nm,4nm
Data3:Abd18_T20.lcd PDA Ch1 254nm,4nm
Data4:Abd18_T30.lcd PDA Ch1 254nm,4nm
Data5:Abd18_T60.lcd PDA Ch1 254nm,4nm
Data6:Abd18_T120.lcd PDA Ch1 254nm,4nm
min

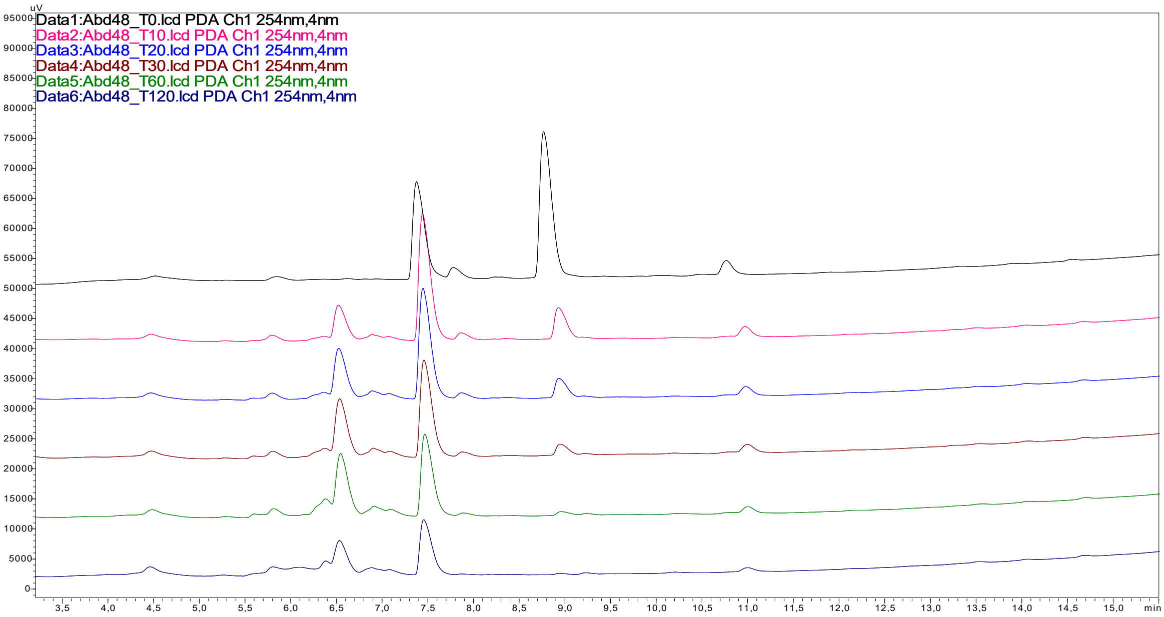
uV
Data1:Abd48_T0.lcd PDA Ch1 254nm,4nm
Data2:Abd48_T10.lcd PDA Ch1 254nm,4nm
Data3:Abd48_T20.lcd PDA Ch1 254nm,4nm
Data4:Abd48_T30.lcd PDA Ch1 254nm,4nm
Data5:Abd48_T60.lcd PDA Ch1 254nm,4nm
Data6:Abd48_T120.lcd PDA Ch1 254nm,4nm
min

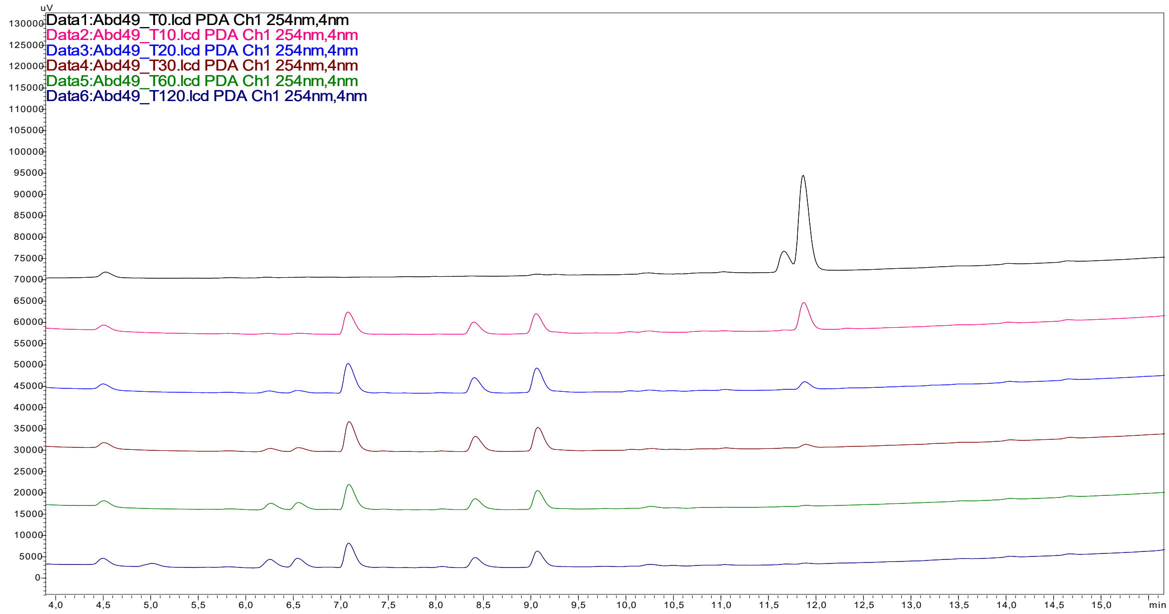
uV
Data1:Abd49_T0.lcd PDA Ch1 254nm,4nm
Data2:Abd49_T10.lcd PDA Ch1 254nm,4nm
Data3:Abd49_T20.lcd PDA Ch1 254nm,4nm
Data4:Abd49_T30.lcd PDA Ch1 254nm,4nm
Data5:Abd49_T60.lcd PDA Ch1 254nm,4nm
Data6:Abd49_T120.lcd PDA Ch1 254nm,4nm
min

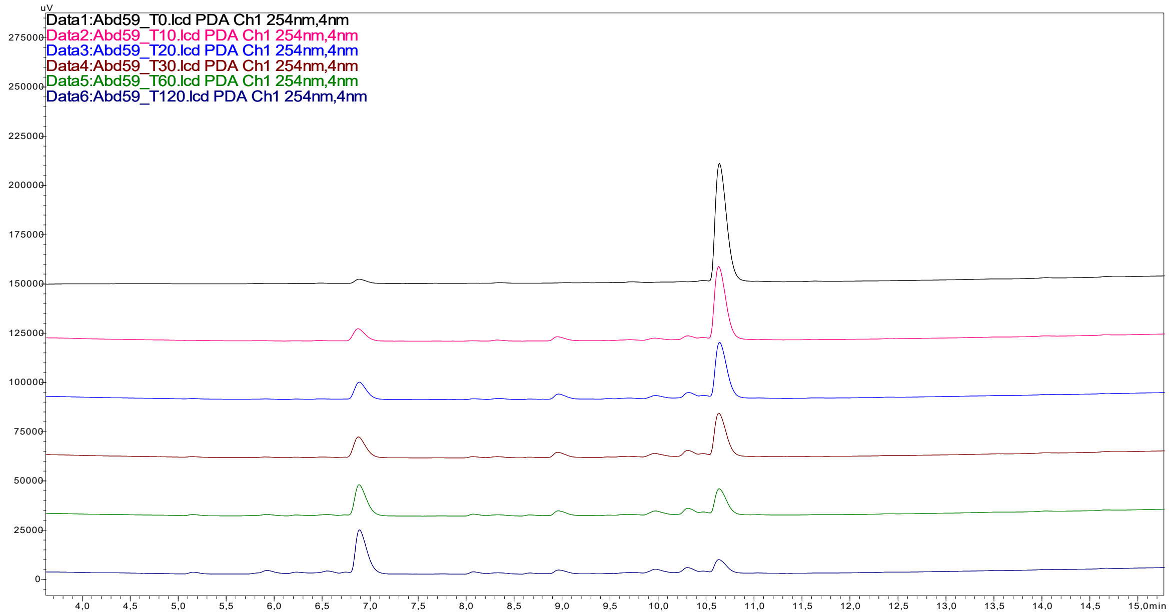
uV
Data1:Abd59_T0.lcd PDA Ch1 254nm,4nm
Data2:Abd59_T10.lcd PDA Ch1 254nm,4nm
Data3:Abd59_T20.lcd PDA Ch1 254nm,4nm
Data4:Abd59_T30.lcd PDA Ch1 254nm,4nm
Data5:Abd59_T60.lcd PDA Ch1 254nm,4nm
Data6:Abd59_T120.lcd PDA Ch1 254nm,4nm
min

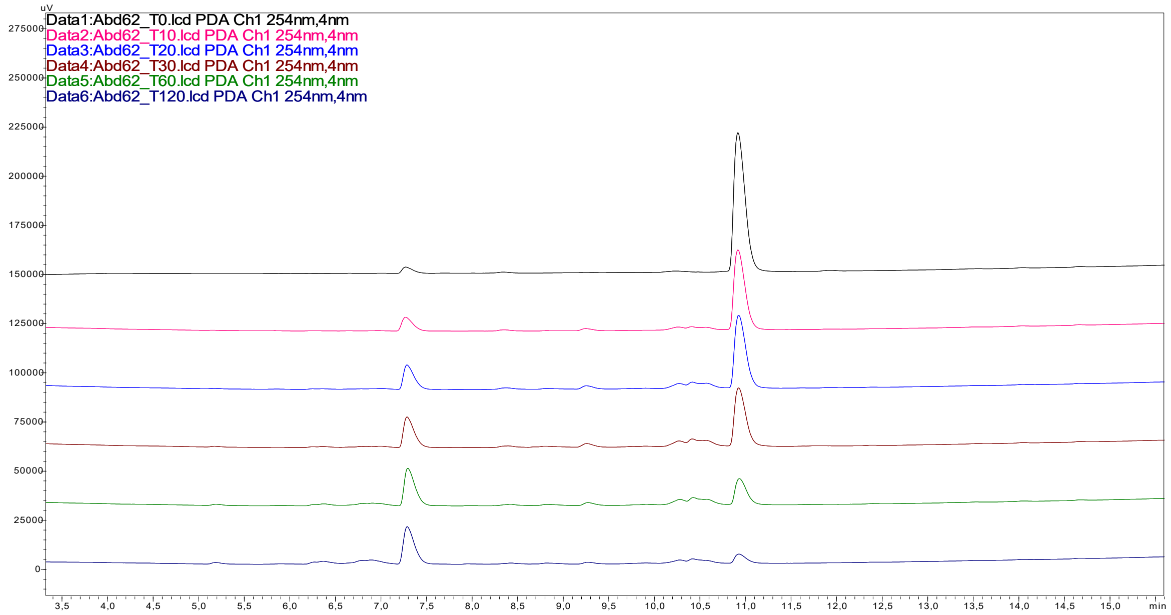
uV
Data1:Abd62_T0.lcd PDA Ch1 254nm,4nm
Data2:Abd62_T10.lcd PDA Ch1 254nm,4nm
Data3:Abd62_T20.lcd PDA Ch1 254nm,4nm
Data4:Abd62_T30.lcd PDA Ch1 254nm,4nm
Data5:Abd62_T60.lcd PDA Ch1 254nm,4nm
Data6:Abd62_T120.lcd PDA Ch1 254nm,4nm
min

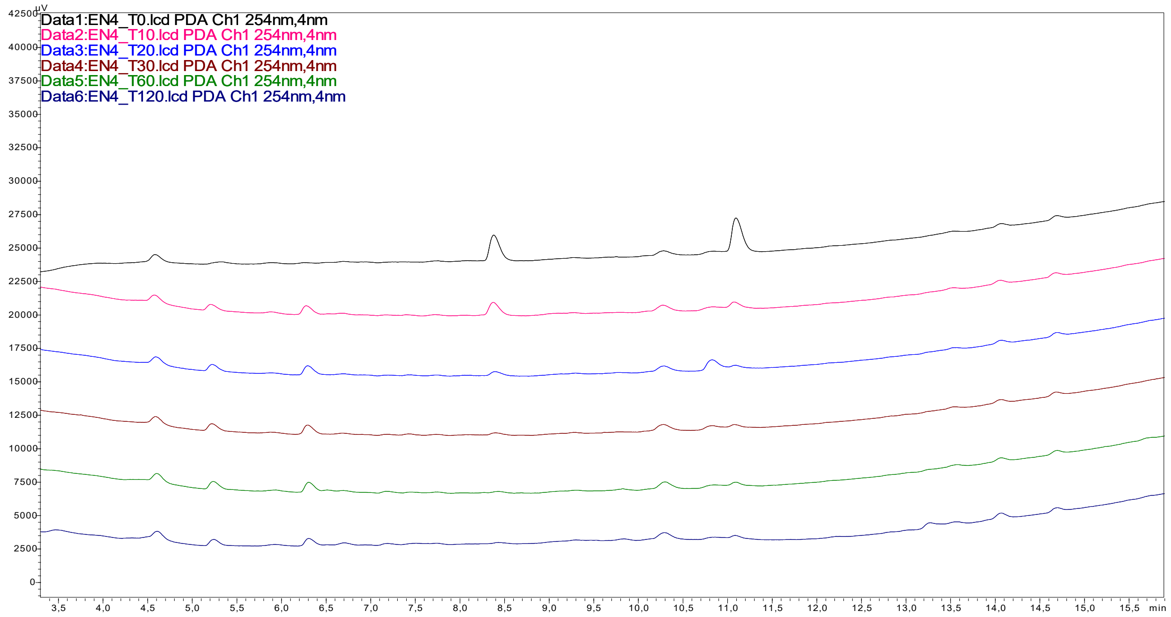
uV
Data1:EN4_T0.lcd PDA Ch1 254nm,4nm
Data2:EN4_T10.lcd PDA Ch1 254nm,4nm
Data3:EN4_T20.lcd PDA Ch1 254nm,4nm
Data4:EN4_T30.lcd PDA Ch1 254nm,4nm
Data5:EN4_T60.lcd PDA Ch1 254nm,4nm
Data6:EN4_T120.lcd PDA Ch1 254nm,4nm
min

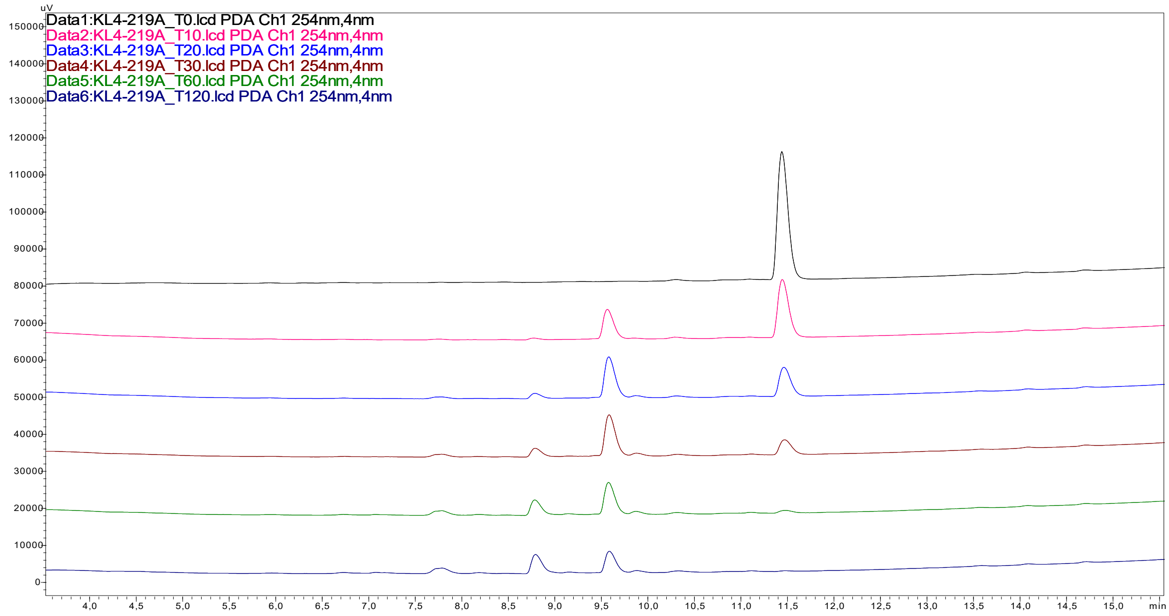
uV
Data1:KL4-219A_T0.lcd PDA Ch1 254nm,4nm
Data2:KL4-219A_T10.lcd PDA Ch1 254nm,4nm
Data3:KL4-219A_T20.lcd PDA Ch1 254nm,4nm
Data4:KL4-219A_T30.lcd PDA Ch1 254nm,4nm
Data5:KL4-219A_T60.lcd PDA Ch1 254nm,4nm
Data6:KL4-219A_T120.lcd PDA Ch1 254nm,4nm
min
